# Supplementary material for: IMPDH2 filaments protect from neurodegeneration in AMPD2 deficiency
Source: EMBO Rep. 2024 Jul 29;25(9):16. doi: 10.1038/s44319-024-00218-2 (PMC11387764; doi:10.1038/s44319-024-00218-2)
Supplement: Supplementary file 1 — Appendix [file 44319_2024_218_MOESM1_ESM.pdf]

Appendix

Table of content:

- **Appendix Figure S1:** Representative MRM chromatograms showing nucleotides from hippocampus of control and double Knockout (dKO) mice………………………**Page 2**
- **Appendix Figure S2:** Representative MRM chromatograms showing nucleotides from PCH9 NPCs expressing FLAG, IMPH2-WT and IMPH2-Y12A…………………**Page 3**

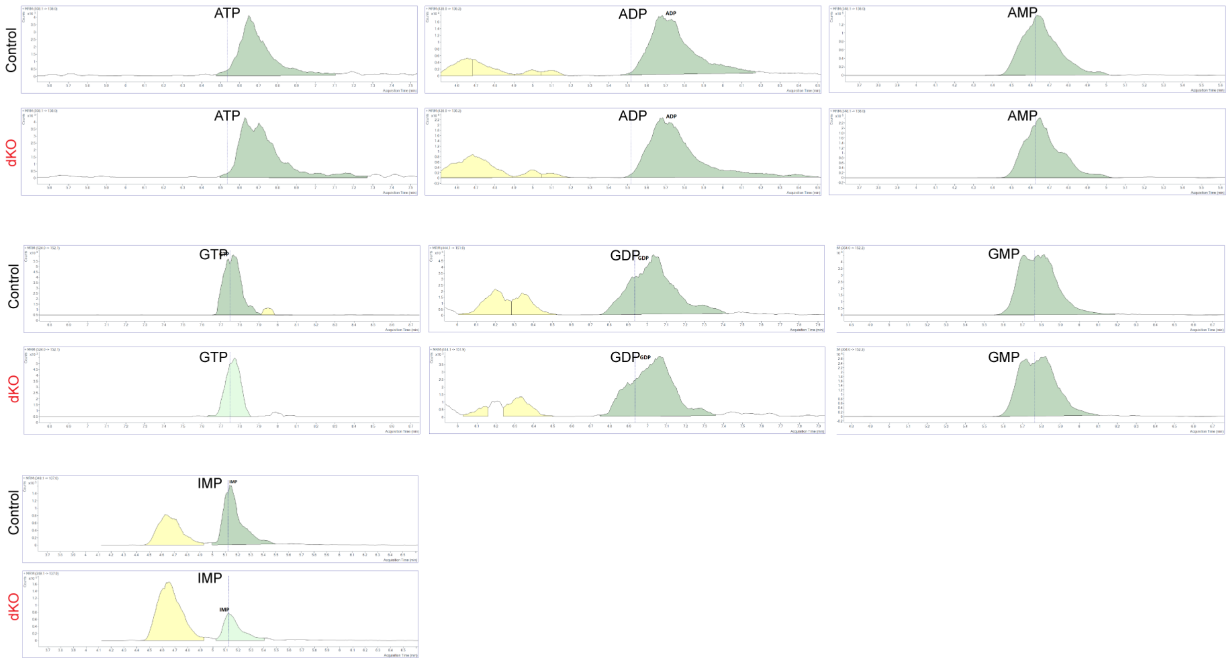

Appendix Figure S1: Representative MRM chromatograms showing nucleotides from hippocampus of control and double Knockout (dKO) mice.

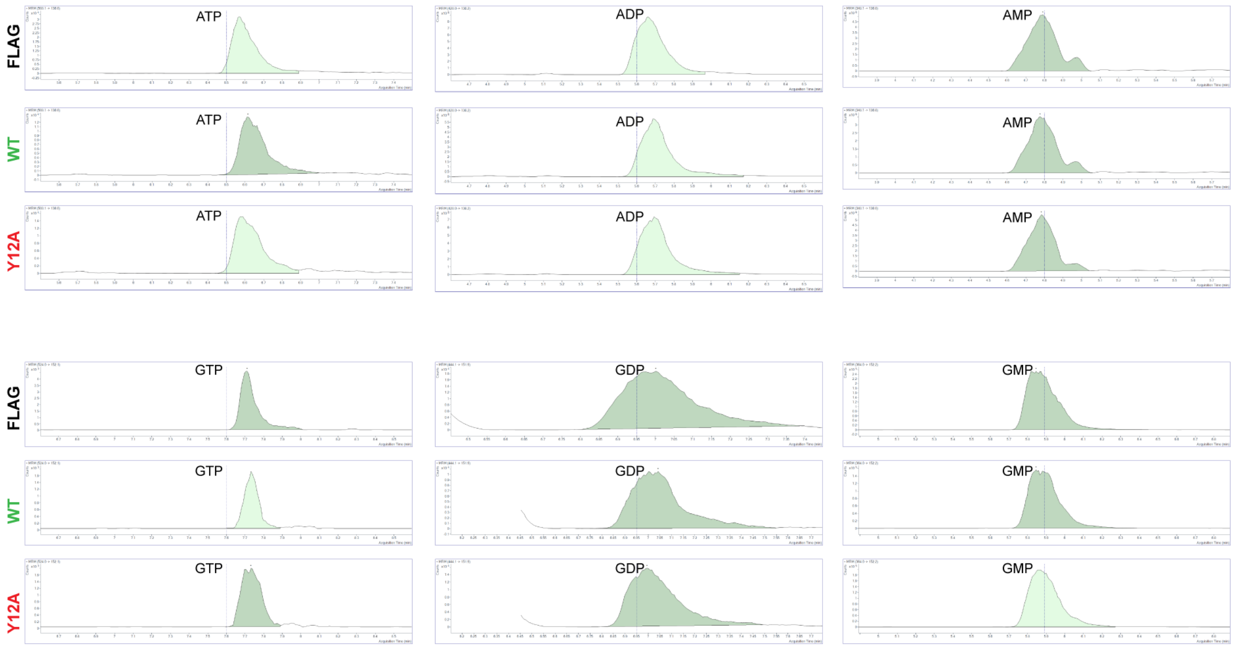

Appendix Figure S2: Representative MRM chromatograms showing nucleotides from PCH9 NPCs expressing FLAG, IMPH2-WT and IMPH2-Y12A
