# Supplementary figures and images for: IMPDH2 filaments protect from neurodegeneration in AMPD2 deficiency (part 1 of 2)
Source: EMBO Rep. 2024 Jul 29;25(9):16. doi: 10.1038/s44319-024-00218-2 (PMC11387764; doi:10.1038/s44319-024-00218-2)

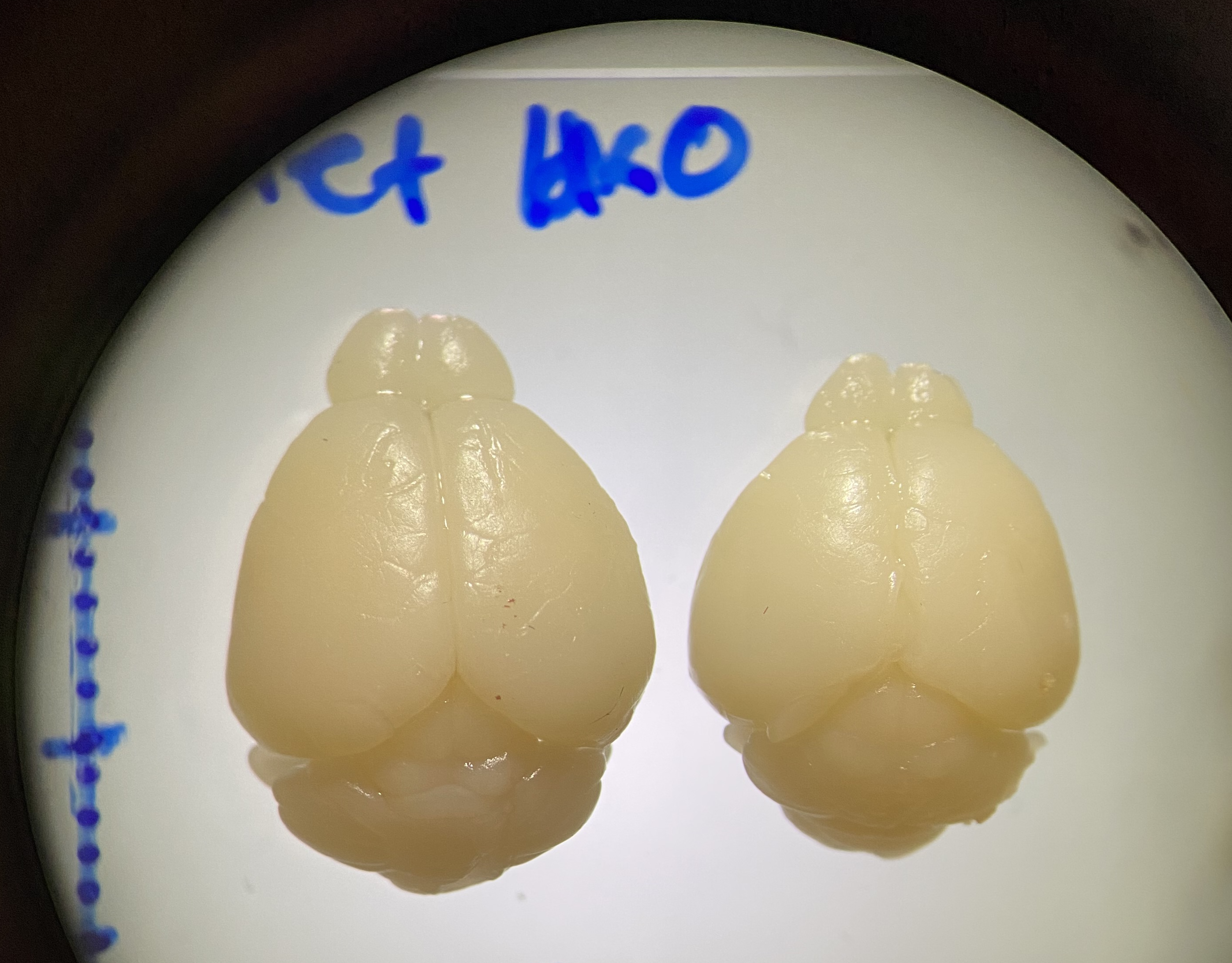

Supplement: Supplementary file 6 — Source data Fig. 1 [file 44319_2024_218_MOESM6_ESM.zip › Figure 1/1C/Untitled.tiff]

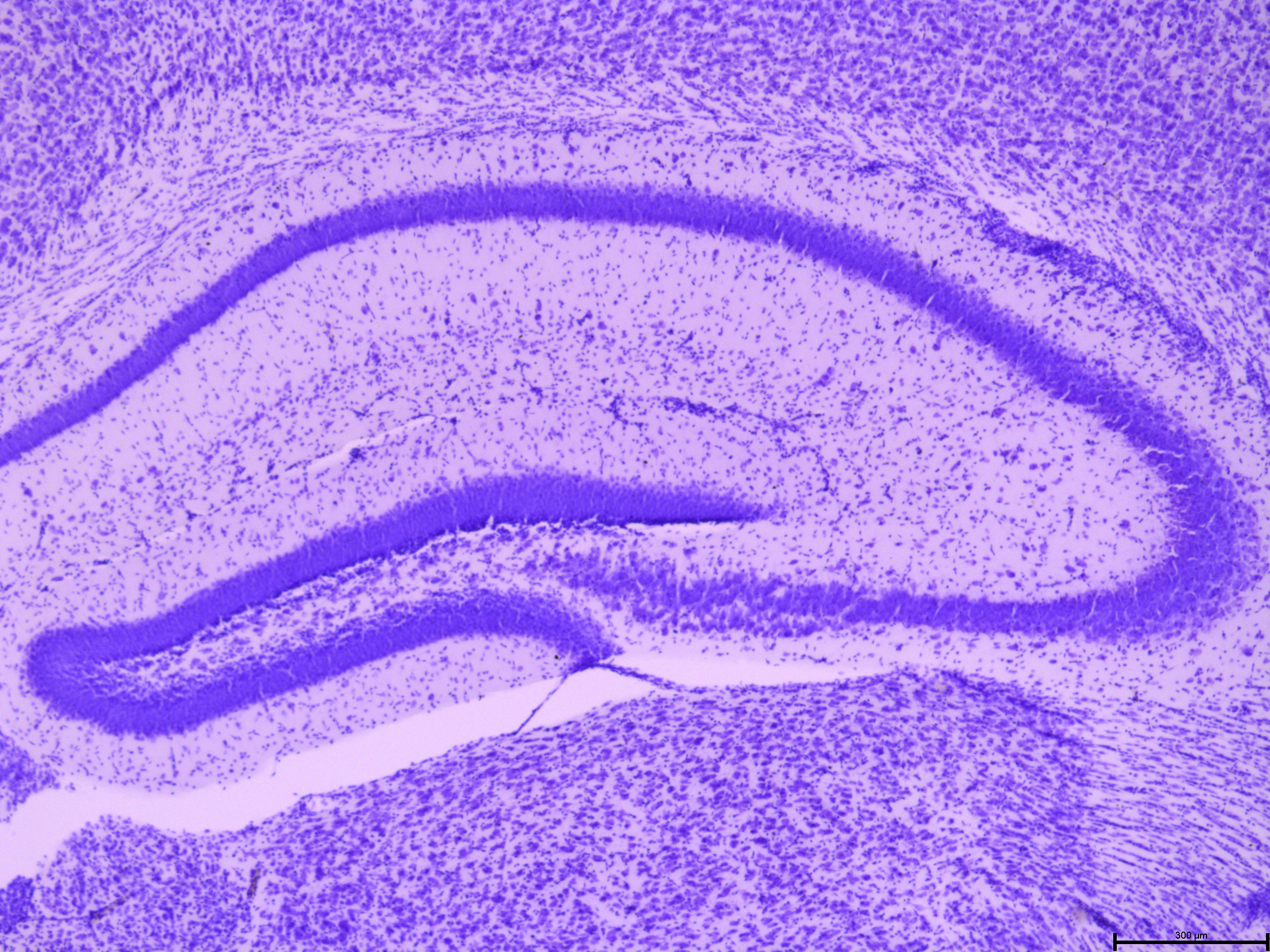

Supplement: Supplementary file 6 — Source data Fig. 1 [file 44319_2024_218_MOESM6_ESM.zip › Figure 1/1E/Ctrl/609 Het, Nissl_609 Het, Hip 5x-III_ch00.tif]

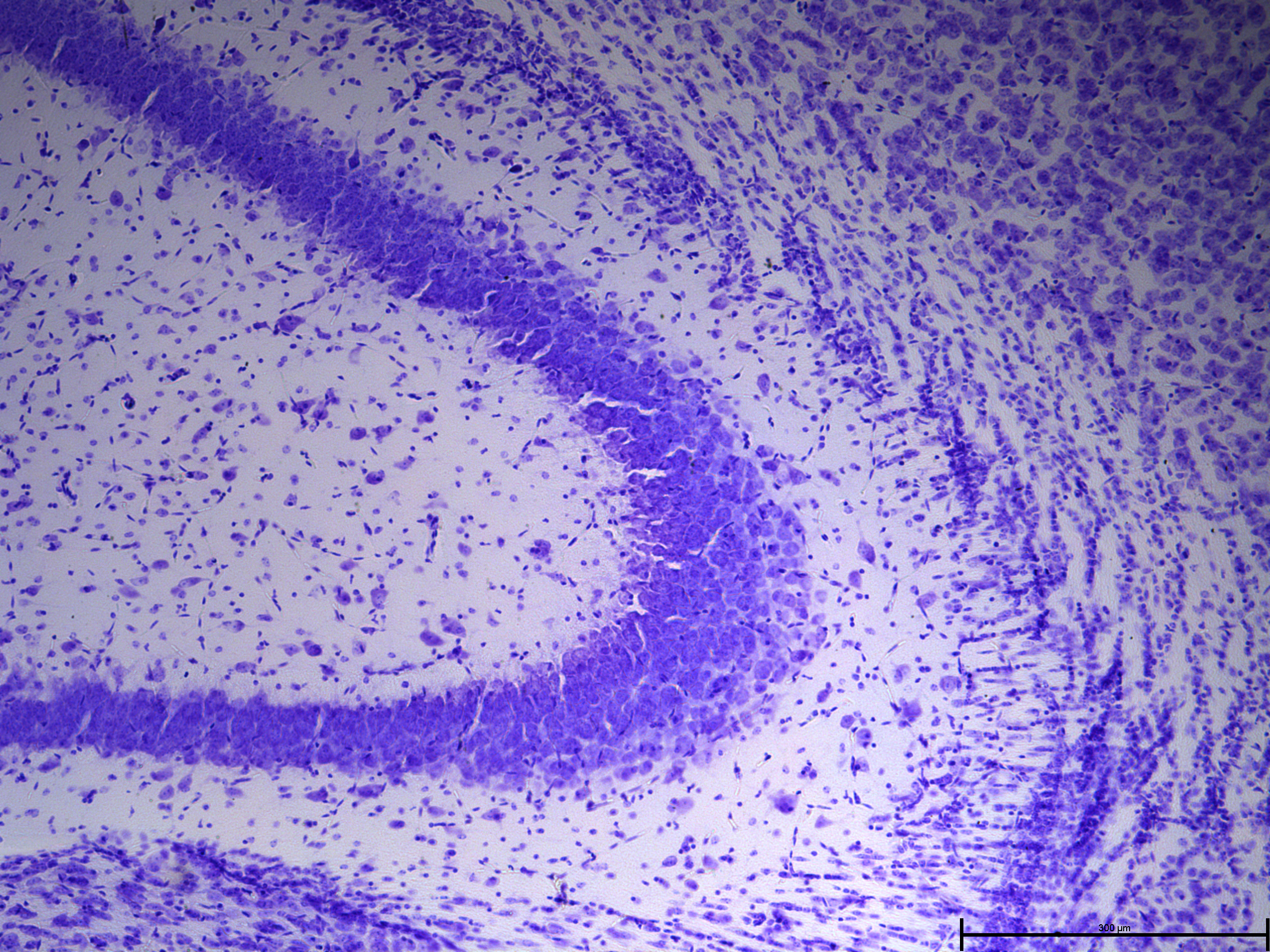

Supplement: Supplementary file 6 — Source data Fig. 1 [file 44319_2024_218_MOESM6_ESM.zip › Figure 1/1E/Ctrl/609 Het, Nissl_609 Het, CA3 10x-I_ch00.tif]

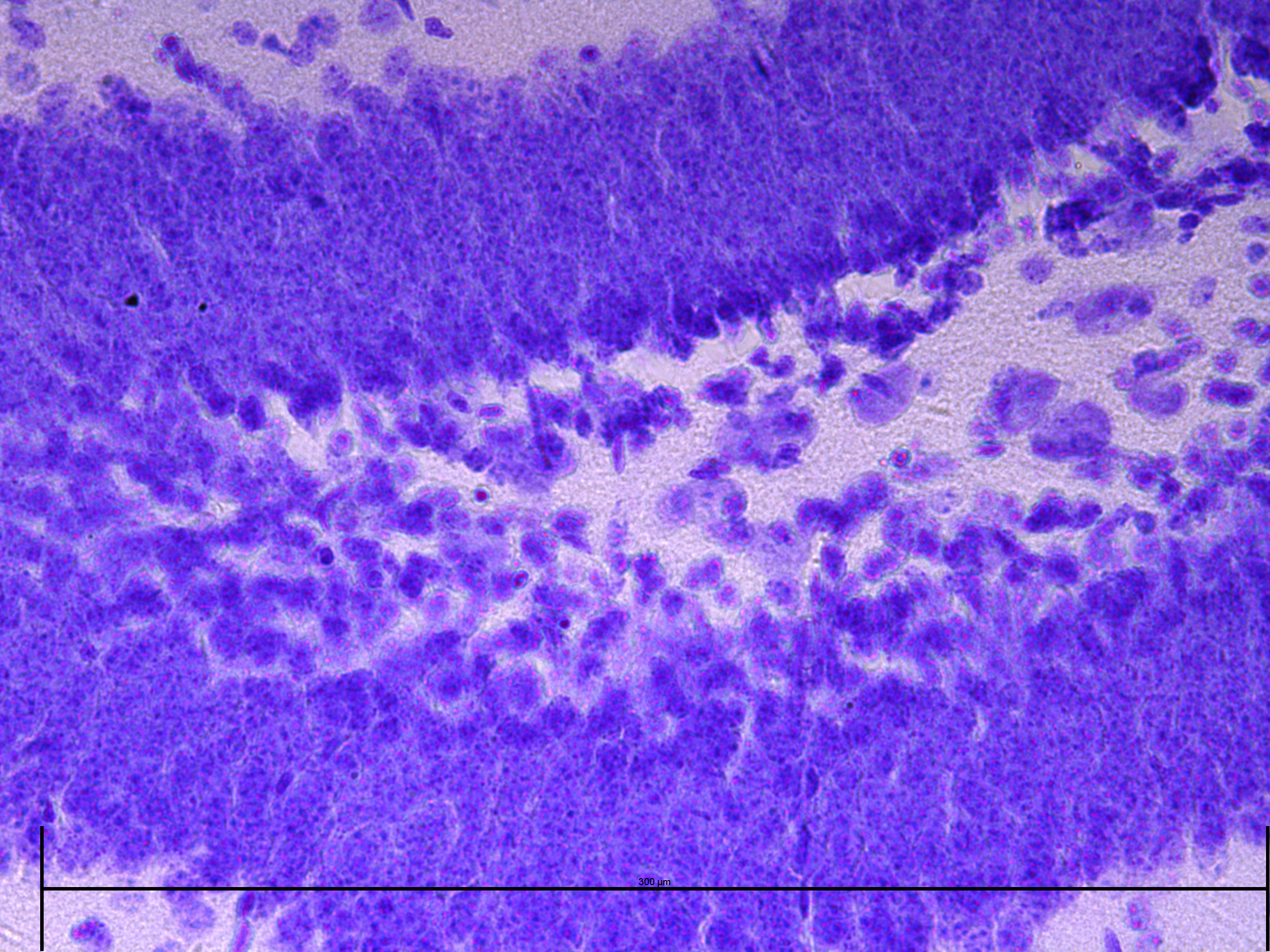

Supplement: Supplementary file 6 — Source data Fig. 1 [file 44319_2024_218_MOESM6_ESM.zip › Figure 1/1E/Ctrl/609 Het, Nissl_609 Het, DG 40x-III_ch00.tif]

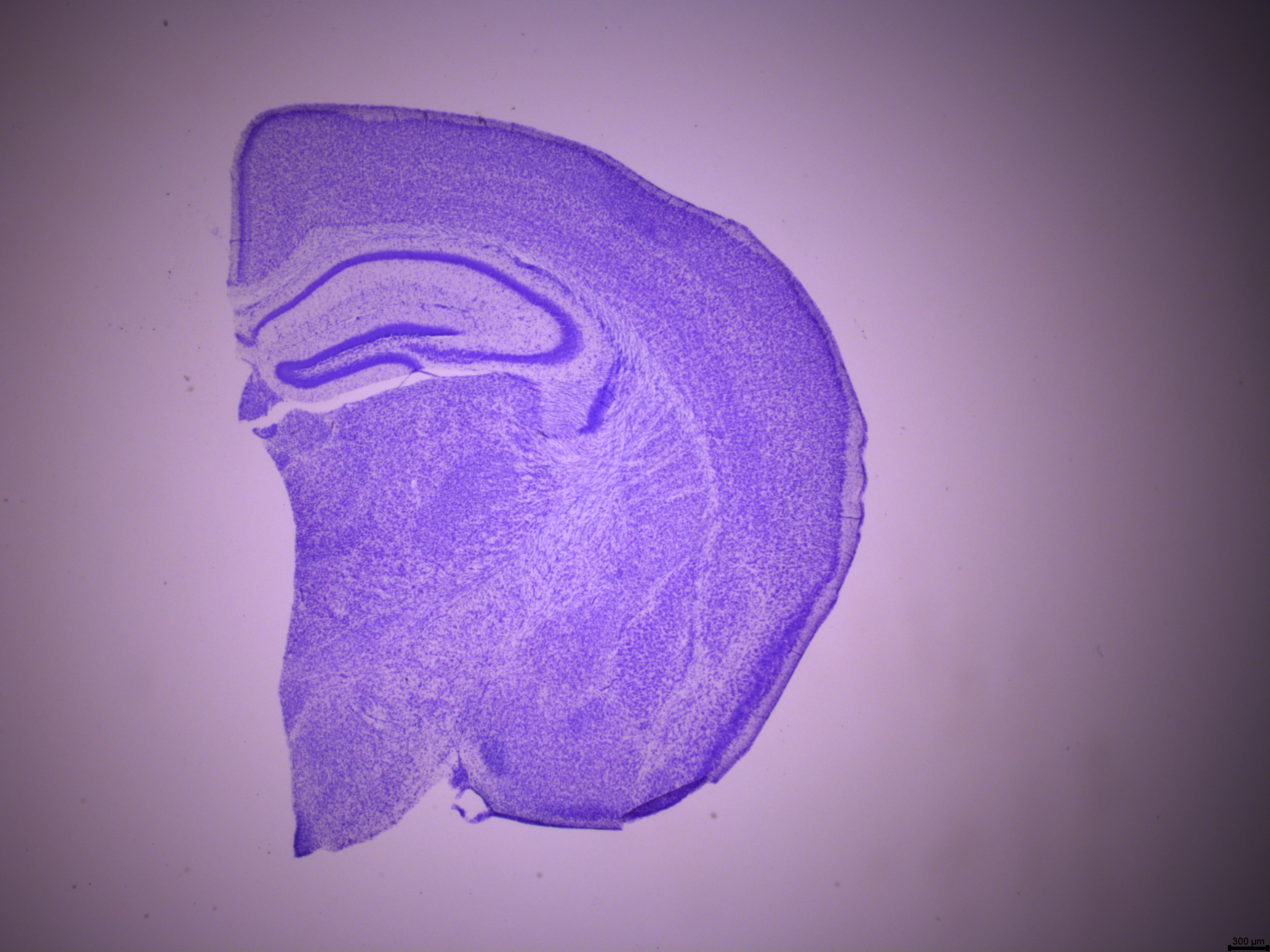

Supplement: Supplementary file 6 — Source data Fig. 1 [file 44319_2024_218_MOESM6_ESM.zip › Figure 1/1E/Ctrl/609 Het, Nissl_609 Het, 1,25x_ch00.tif]

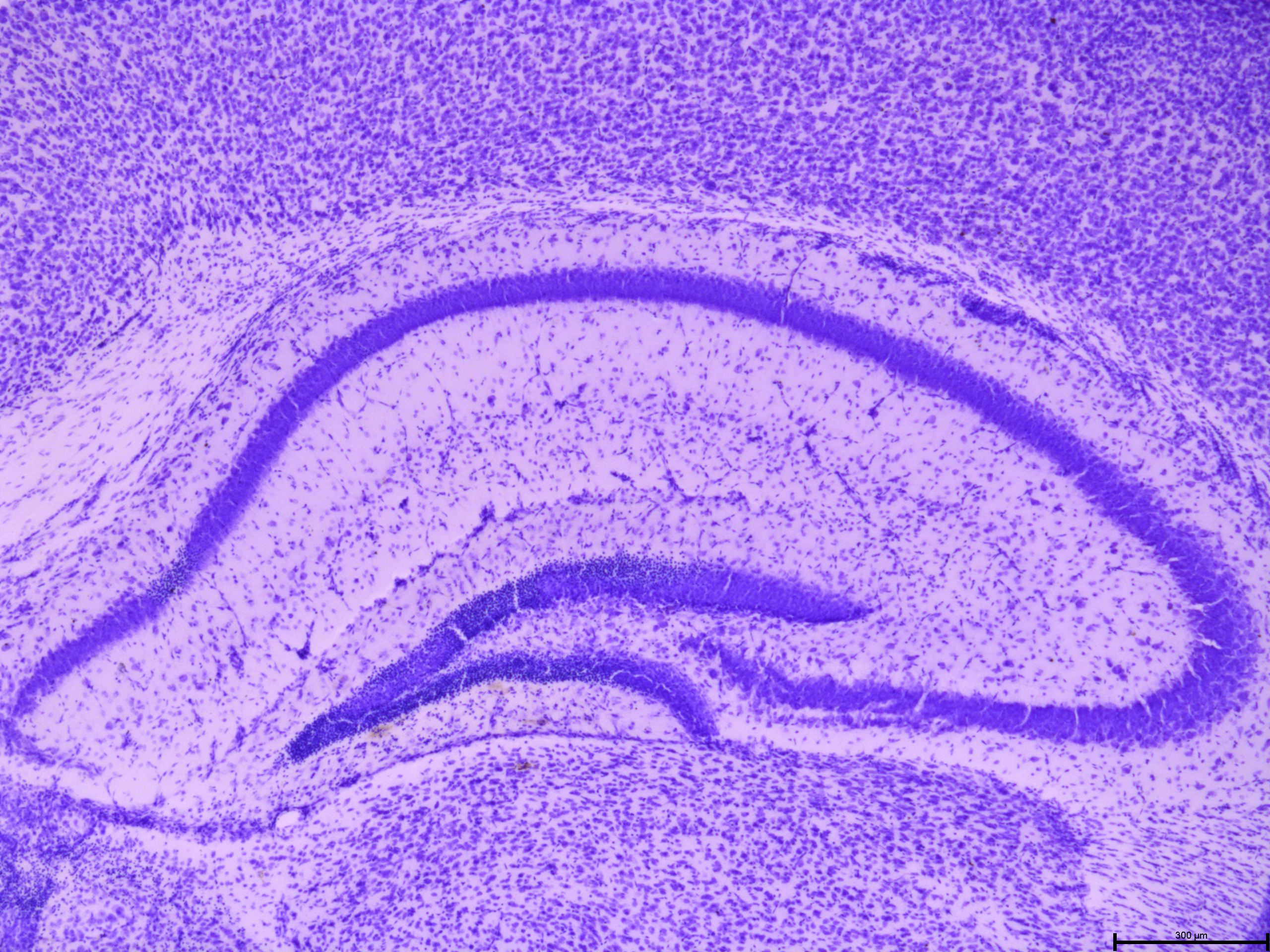

Supplement: Supplementary file 6 — Source data Fig. 1 [file 44319_2024_218_MOESM6_ESM.zip › Figure 1/1E/dKO/Nissl_614dKO, Hip 5x_ch00.tif]

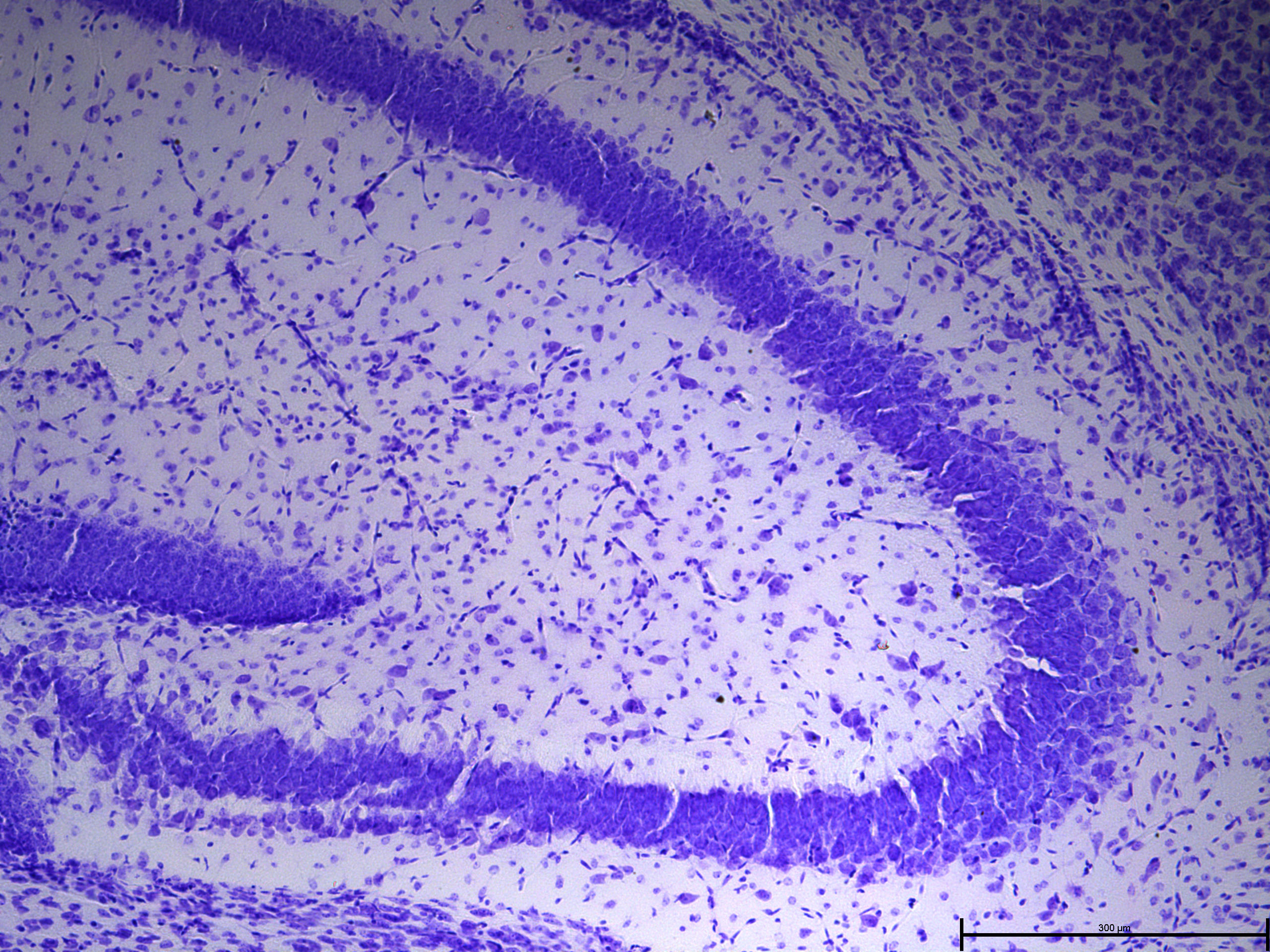

Supplement: Supplementary file 6 — Source data Fig. 1 [file 44319_2024_218_MOESM6_ESM.zip › Figure 1/1E/dKO/Nissl_614dKO, CA3 10x-V_ch00.tif]

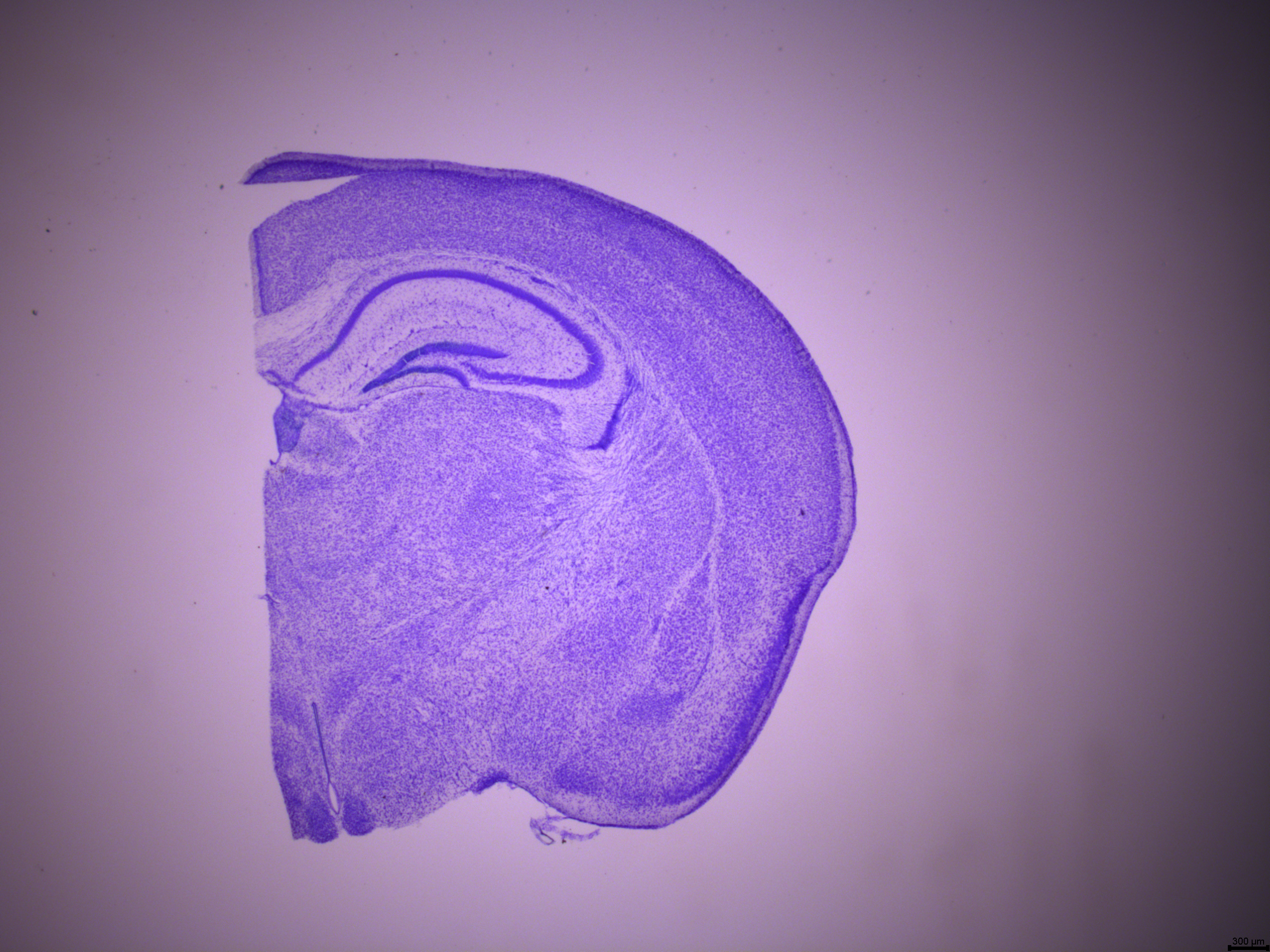

Supplement: Supplementary file 6 — Source data Fig. 1 [file 44319_2024_218_MOESM6_ESM.zip › Figure 1/1E/dKO/Nissl_614dKO, 1,25x_ch00.tif]

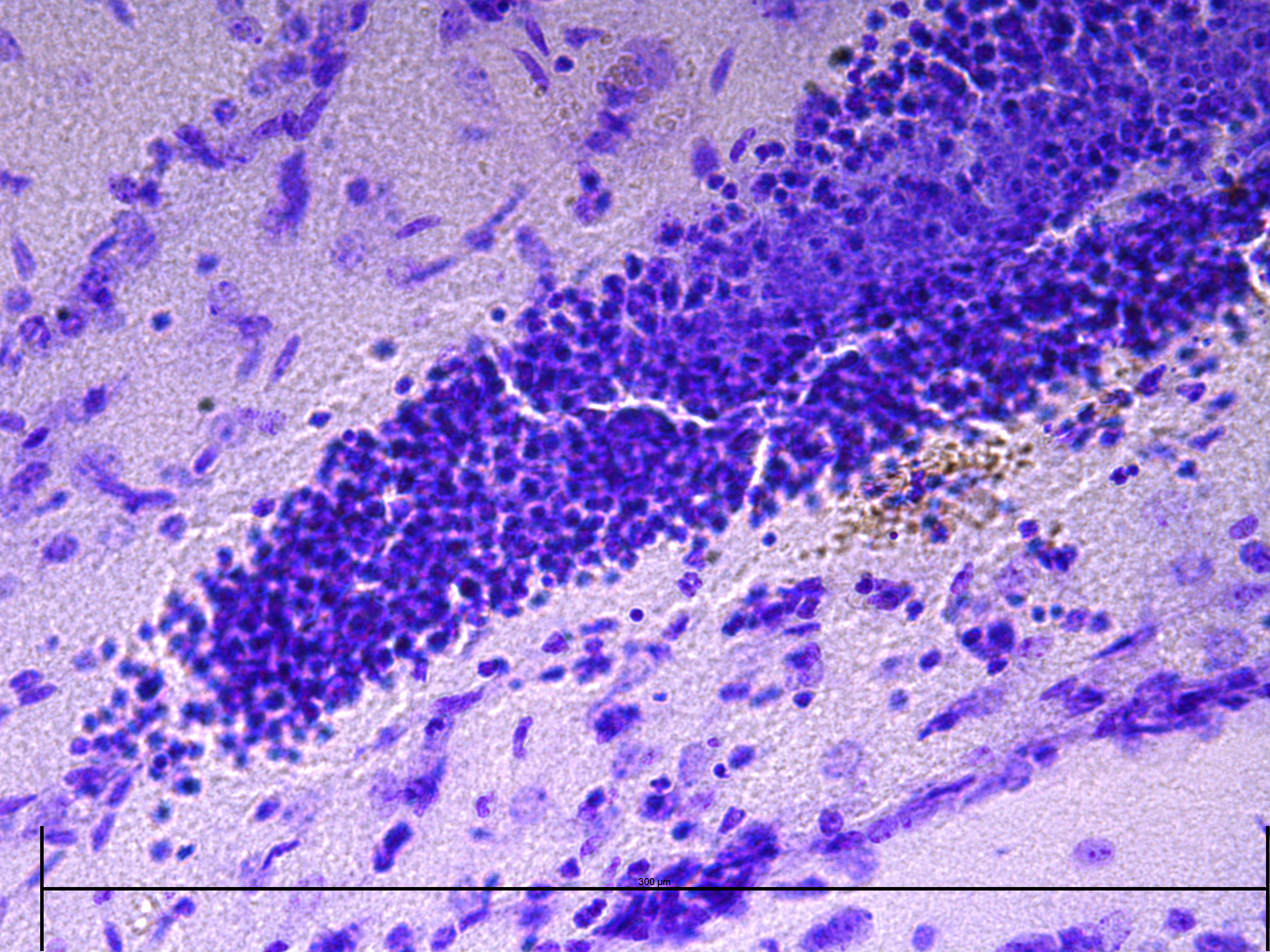

Supplement: Supplementary file 6 — Source data Fig. 1 [file 44319_2024_218_MOESM6_ESM.zip › Figure 1/1E/dKO/Nissl_614dKO, DG 40x-I_ch00.tif]

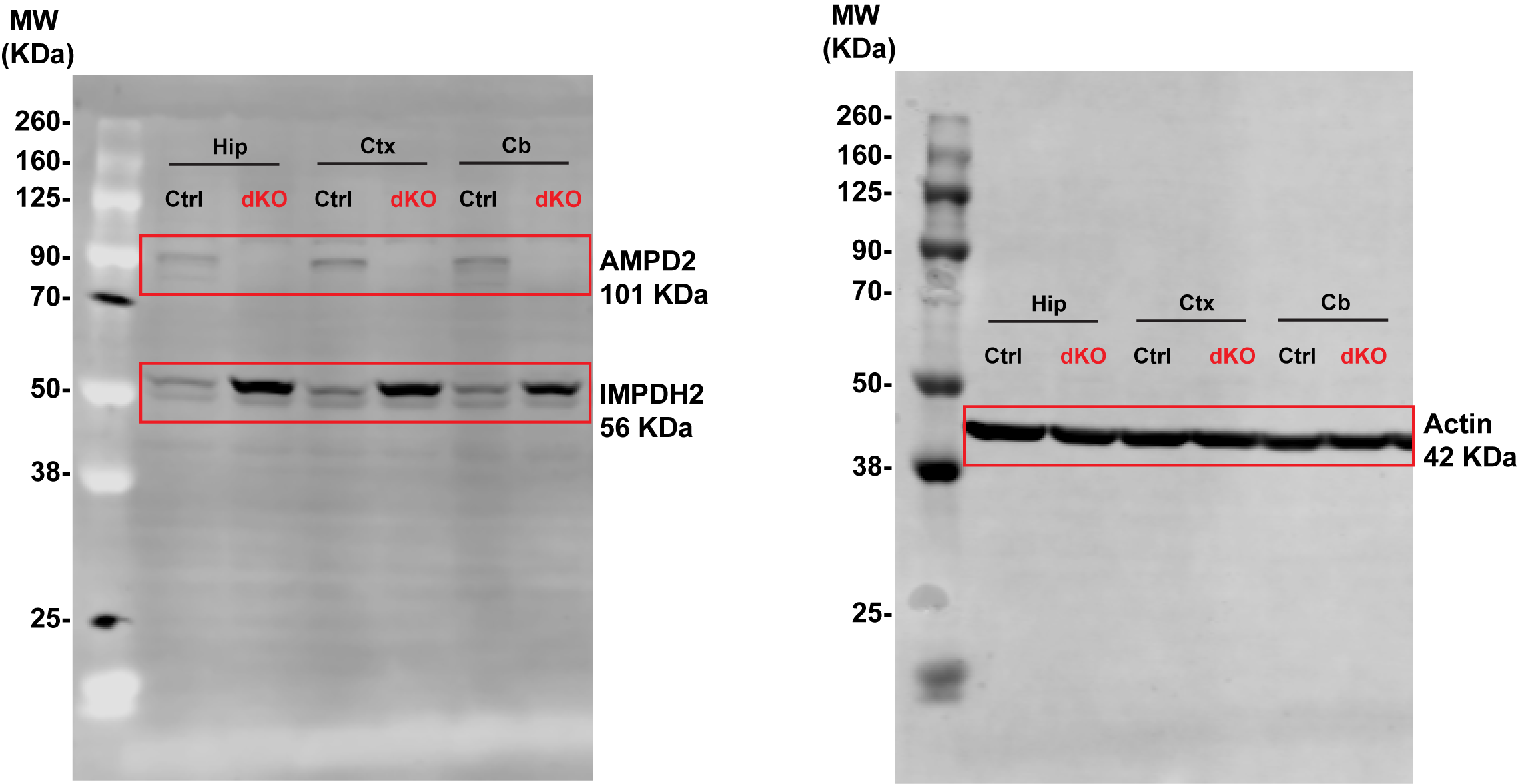

Supplement: Supplementary file 7 — Source data Fig. 2 [file 44319_2024_218_MOESM7_ESM.zip › Figure 2/2D/WB 2D.tif]

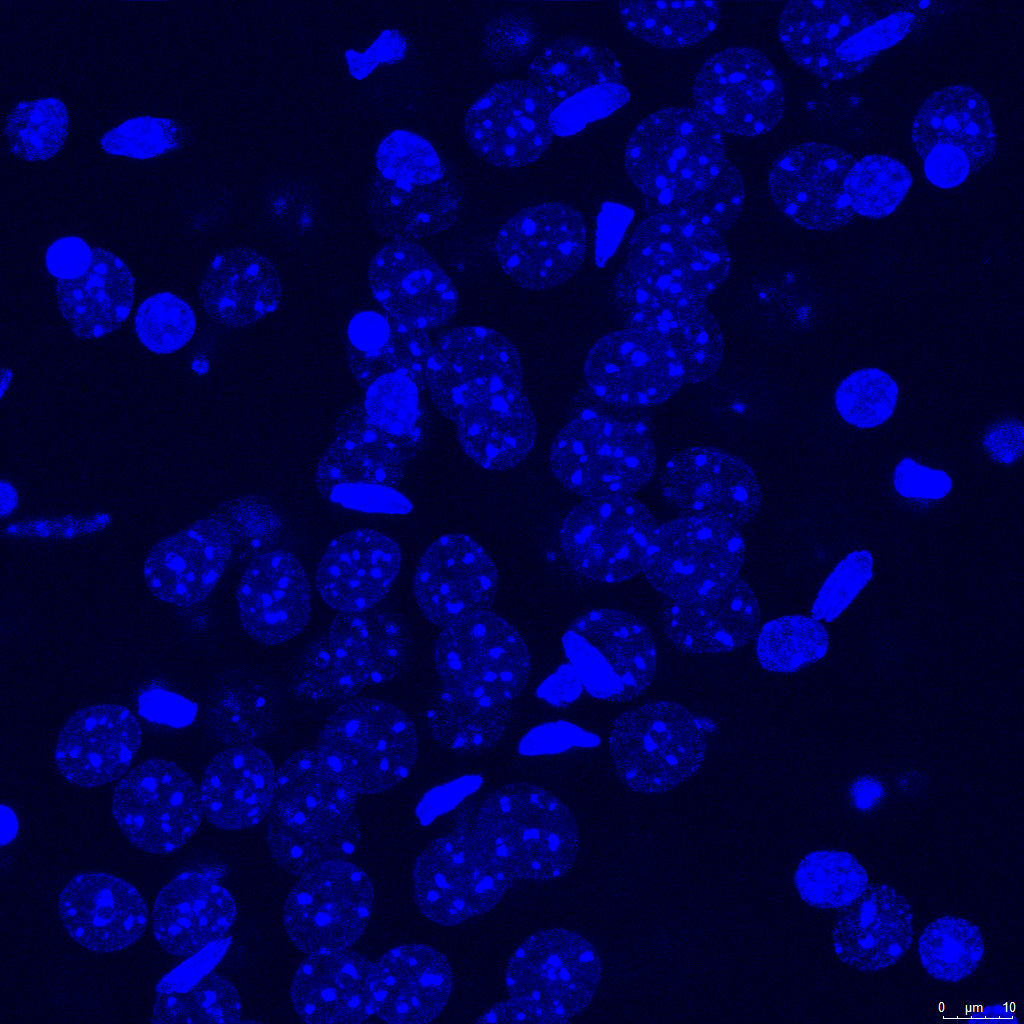

Supplement: Supplementary file 7 — Source data Fig. 2 [file 44319_2024_218_MOESM7_ESM.zip › Figure 2/2F/Hip/Het, S2 CA2-3, GFAP488-G, IMPDH555-R, NeuN633-W, ZS 40xZF2-I_ch00.tif]

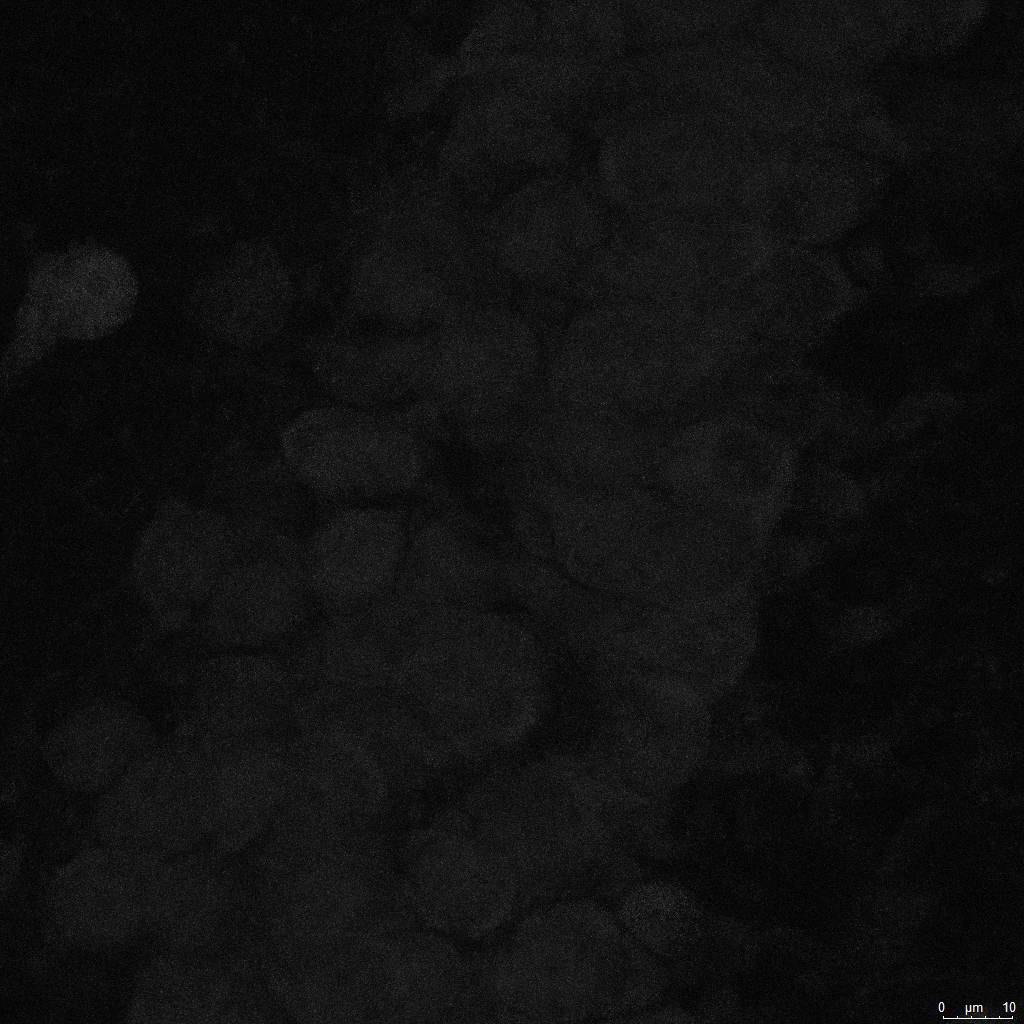

Supplement: Supplementary file 7 — Source data Fig. 2 [file 44319_2024_218_MOESM7_ESM.zip › Figure 2/2F/Hip/Het, S2 CA2-3, GFAP488-G, IMPDH555-R, NeuN633-W, ZS 40xZF2-I_ch03.tif]

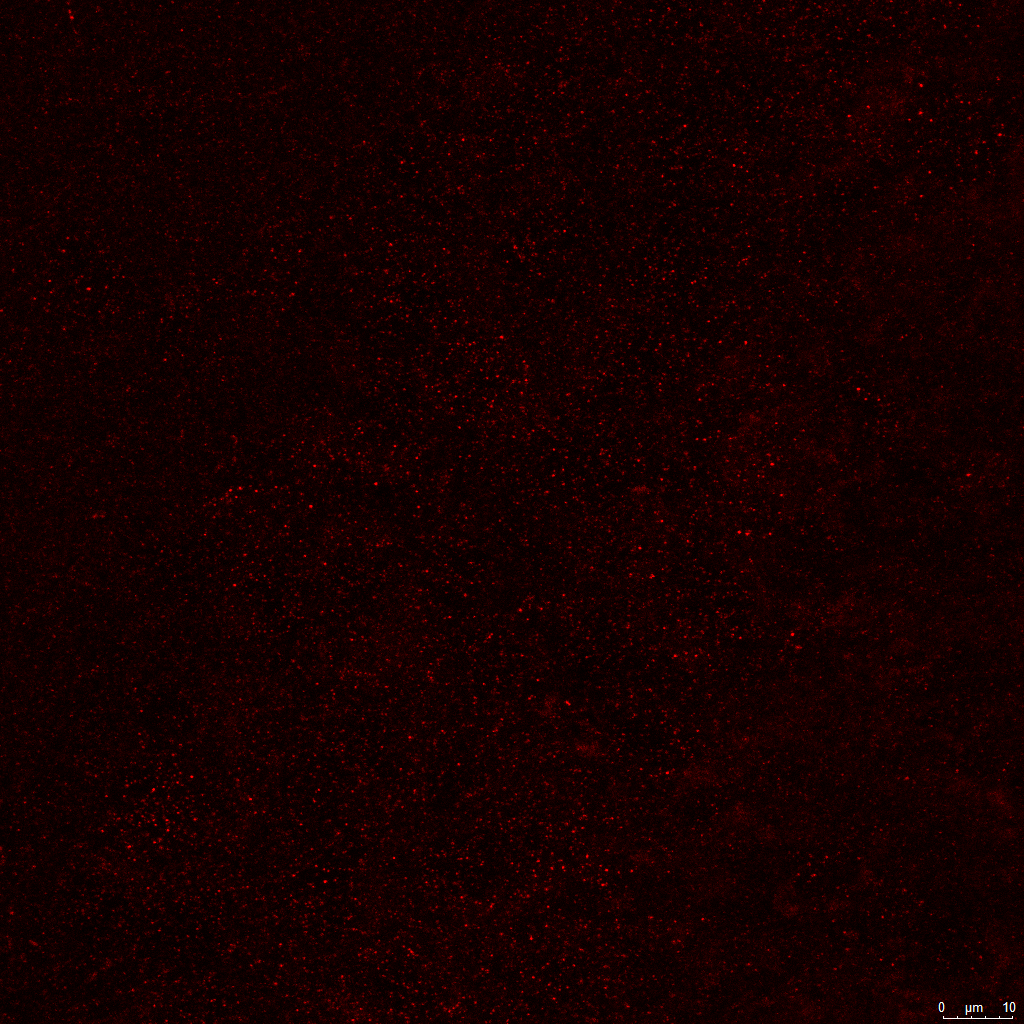

Supplement: Supplementary file 7 — Source data Fig. 2 [file 44319_2024_218_MOESM7_ESM.zip › Figure 2/2F/Hip/Het, S2 CA2-3, GFAP488-G, IMPDH555-R, NeuN633-W, ZS 40xZF2-I_ch02.tif]

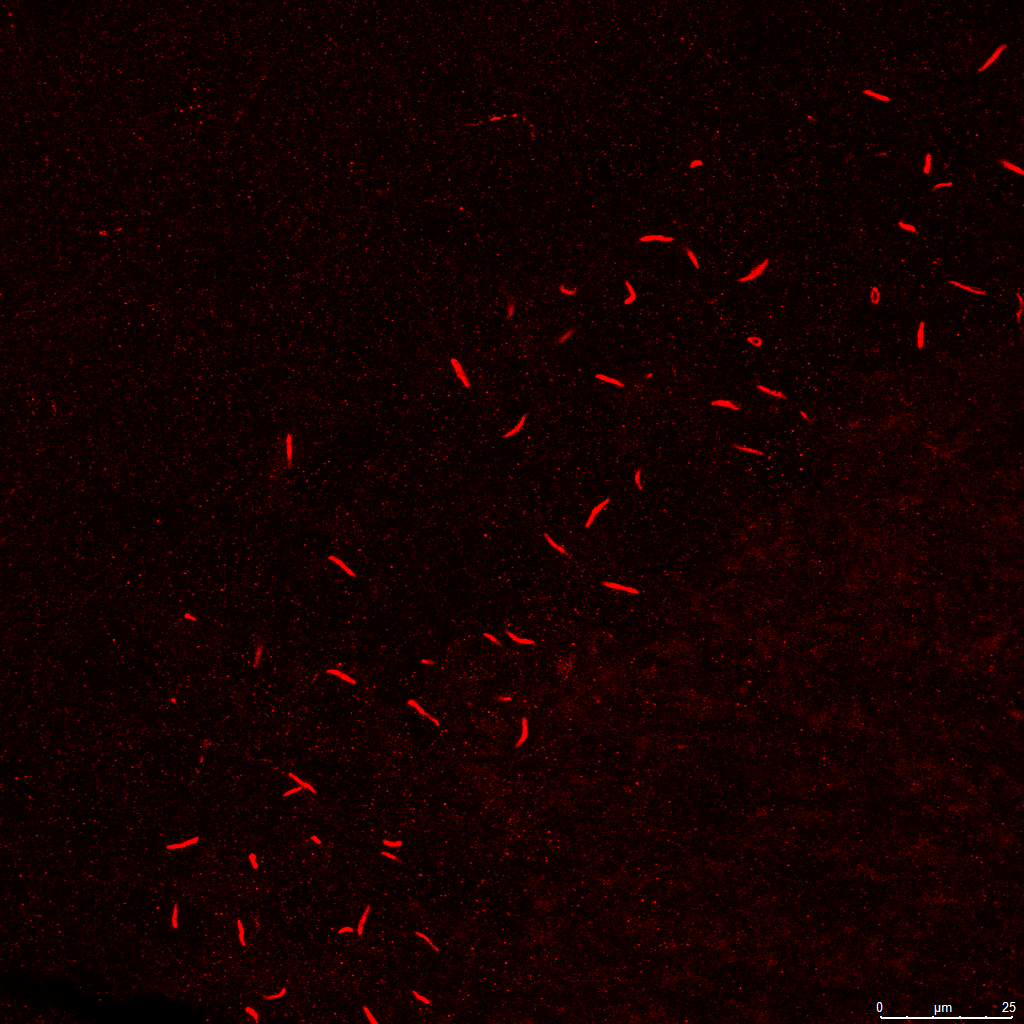

Supplement: Supplementary file 7 — Source data Fig. 2 [file 44319_2024_218_MOESM7_ESM.zip › Figure 2/2F/Hip/dKO, S2 CA2-3, GFAP488-G, IMPDH555-R, NeuN633-W, ZS 40xZF2-II_ch02.tif]

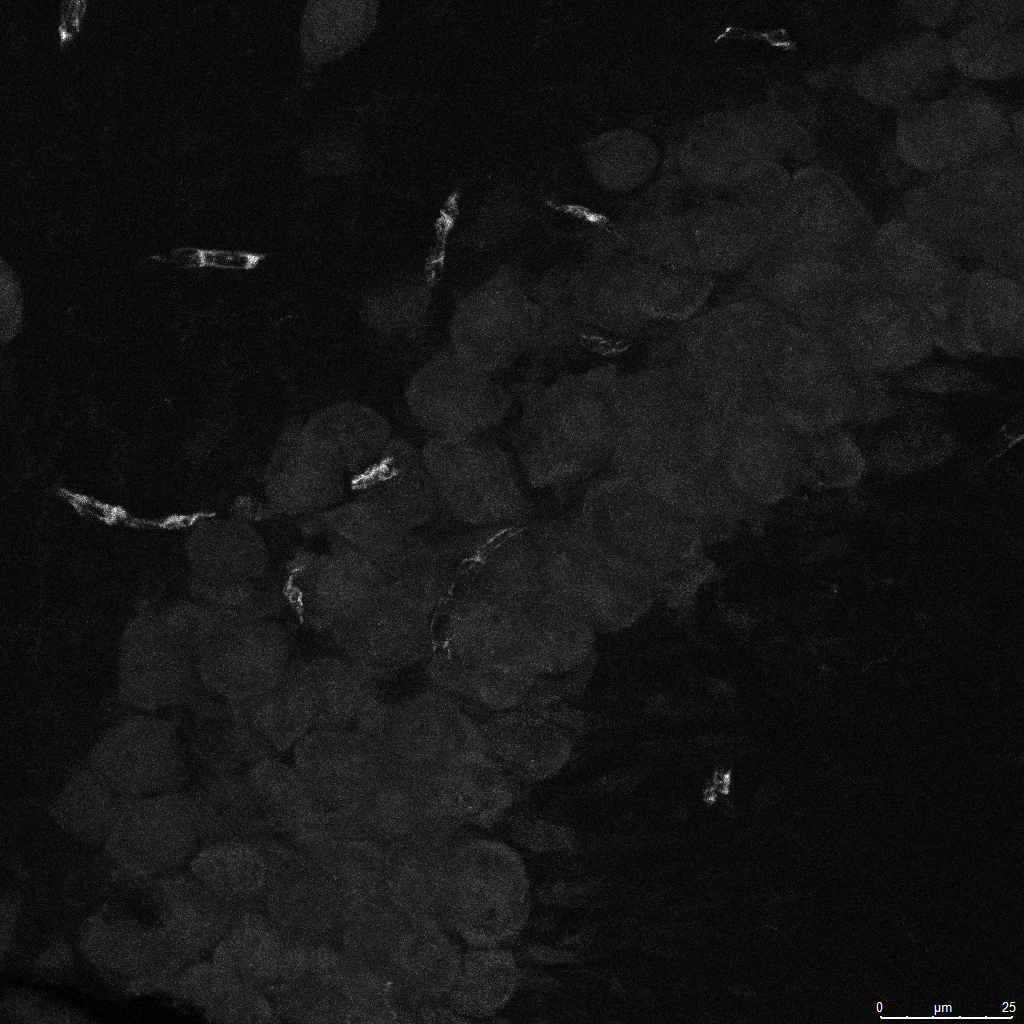

Supplement: Supplementary file 7 — Source data Fig. 2 [file 44319_2024_218_MOESM7_ESM.zip › Figure 2/2F/Hip/dKO, S2 CA2-3, GFAP488-G, IMPDH555-R, NeuN633-W, ZS 40xZF2-II_ch03.tif]

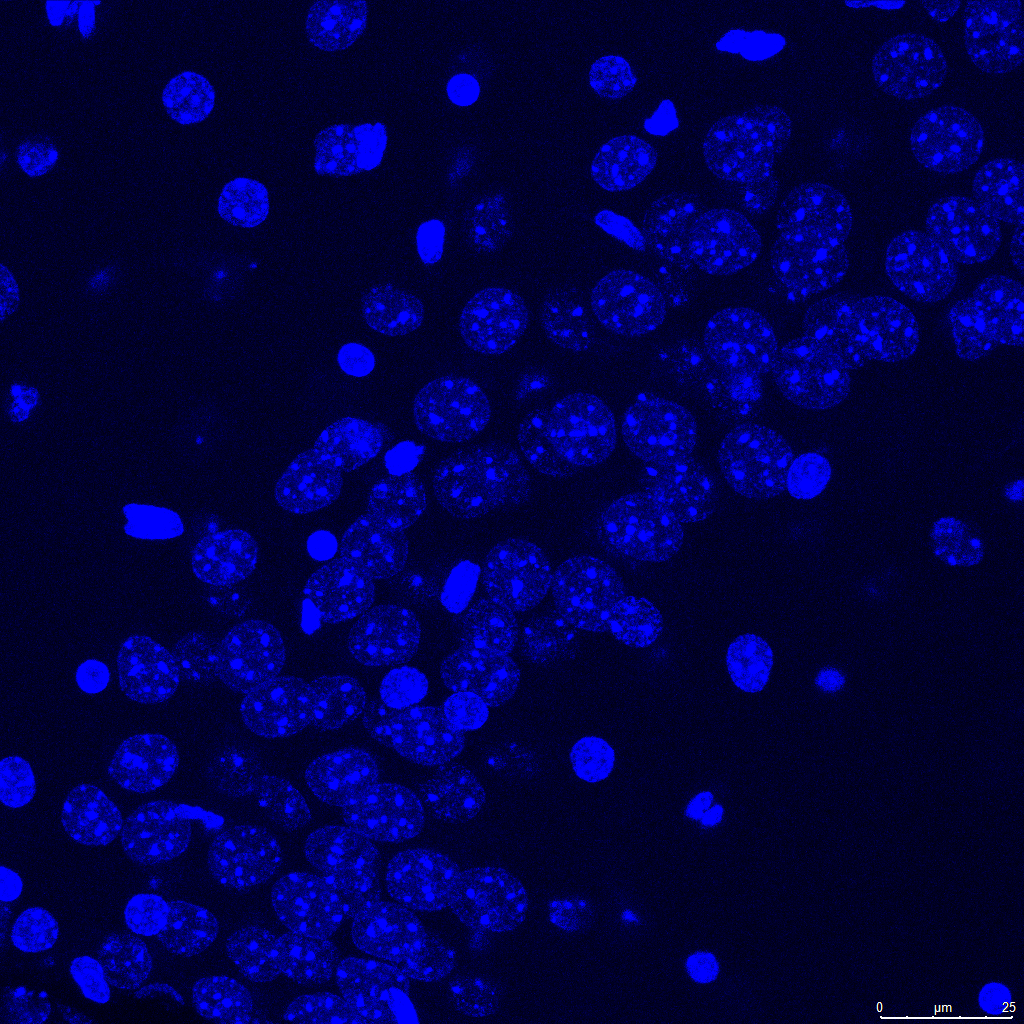

Supplement: Supplementary file 7 — Source data Fig. 2 [file 44319_2024_218_MOESM7_ESM.zip › Figure 2/2F/Hip/dKO, S2 CA2-3, GFAP488-G, IMPDH555-R, NeuN633-W, ZS 40xZF2-II_ch00.tif]

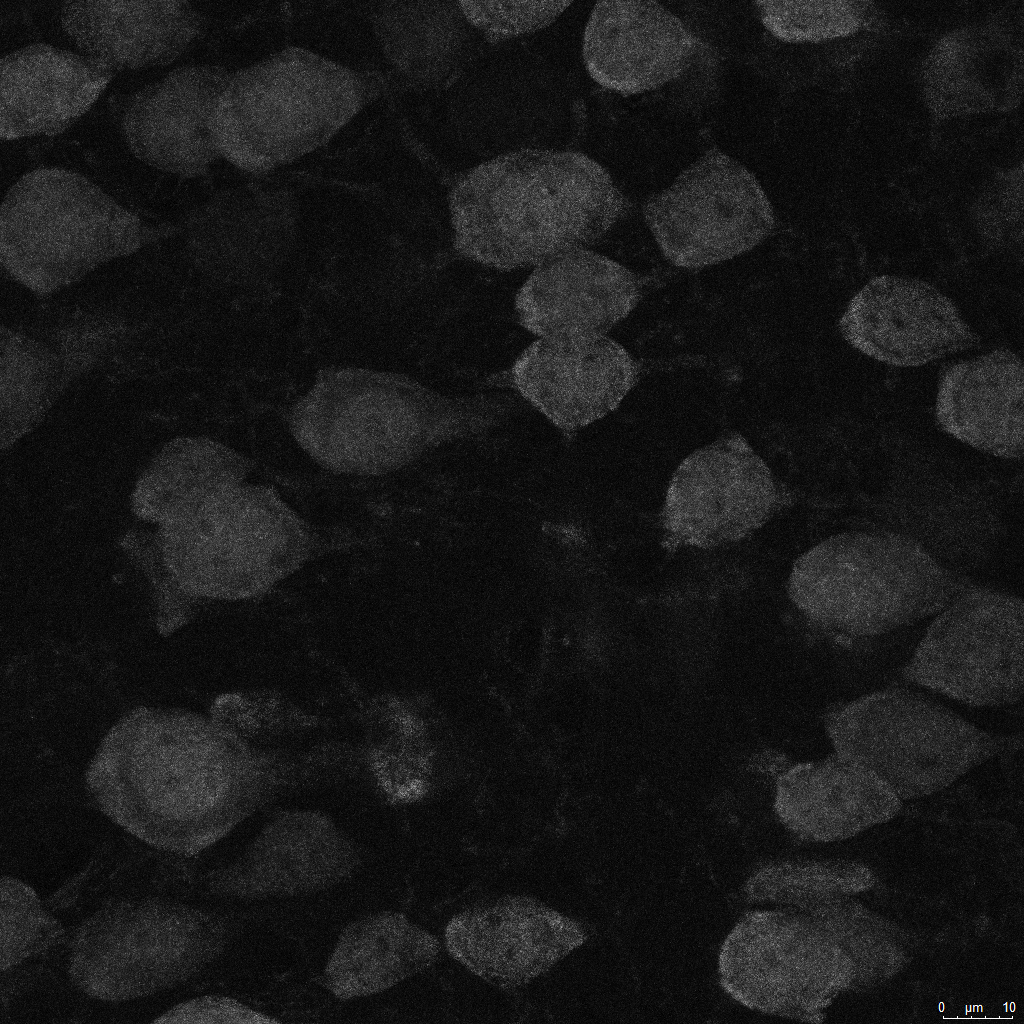

Supplement: Supplementary file 7 — Source data Fig. 2 [file 44319_2024_218_MOESM7_ESM.zip › Figure 2/2F/Ctx/dKO, S2 Cortex, GFAP488-G, IMPDH555-R, NeuN633-W, ZS 40xZF2-II_ch03.tif]

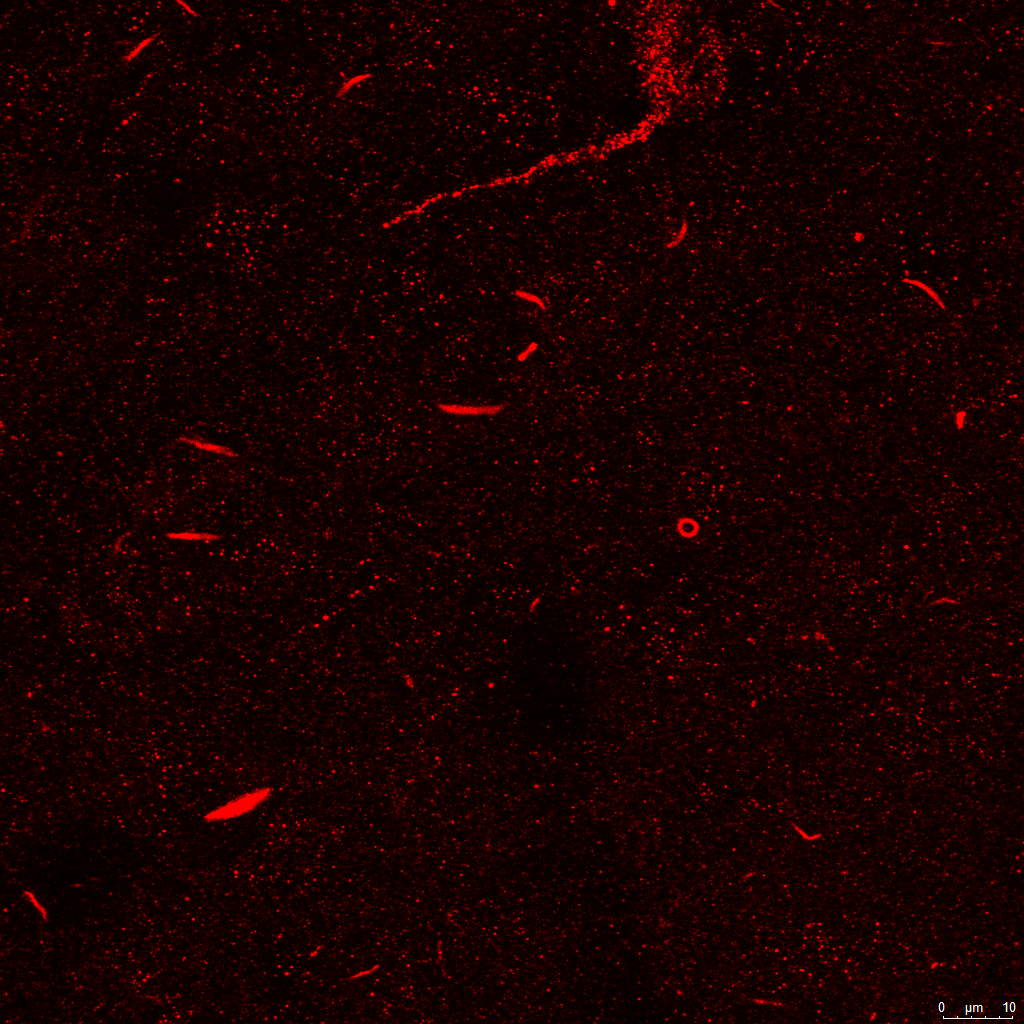

Supplement: Supplementary file 7 — Source data Fig. 2 [file 44319_2024_218_MOESM7_ESM.zip › Figure 2/2F/Ctx/dKO, S2 Cortex, GFAP488-G, IMPDH555-R, NeuN633-W, ZS 40xZF2-II_ch02.tif]

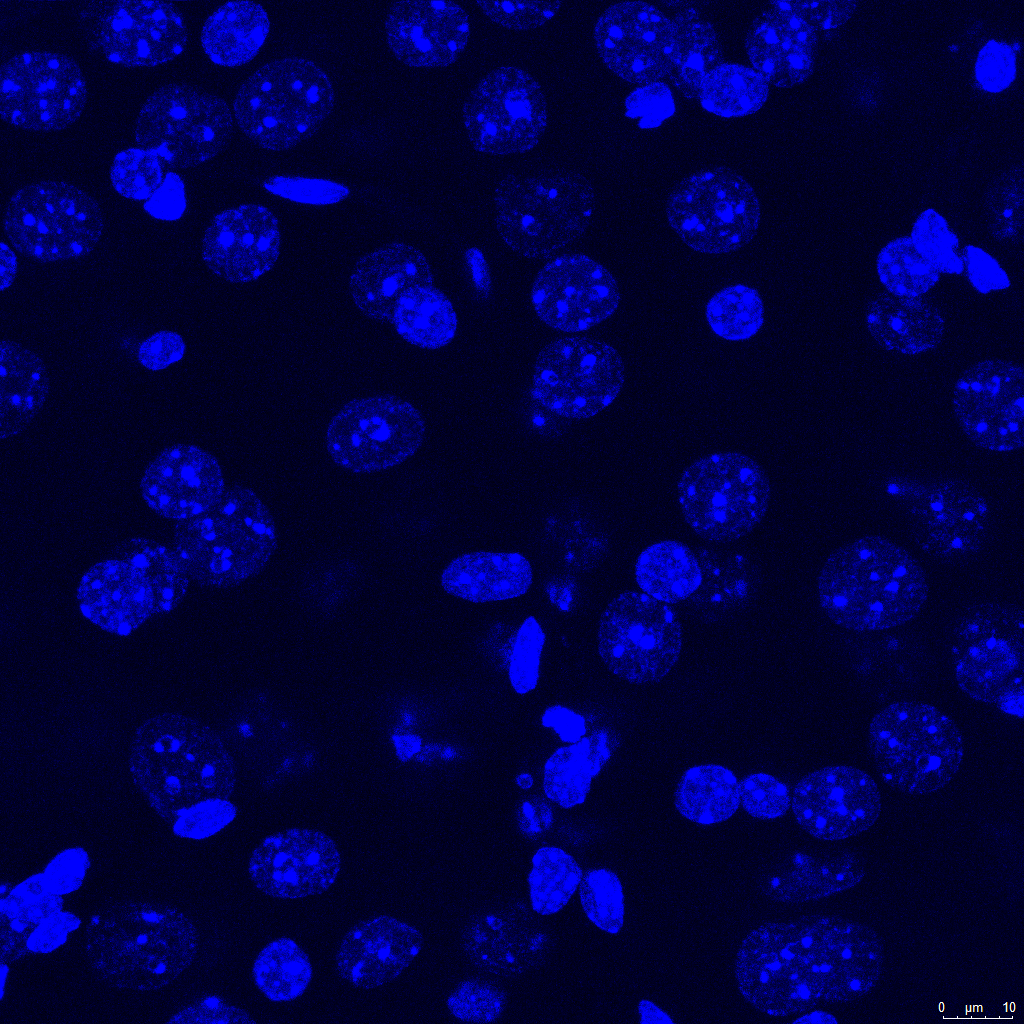

Supplement: Supplementary file 7 — Source data Fig. 2 [file 44319_2024_218_MOESM7_ESM.zip › Figure 2/2F/Ctx/dKO, S2 Cortex, GFAP488-G, IMPDH555-R, NeuN633-W, ZS 40xZF2-II_ch00.tif]

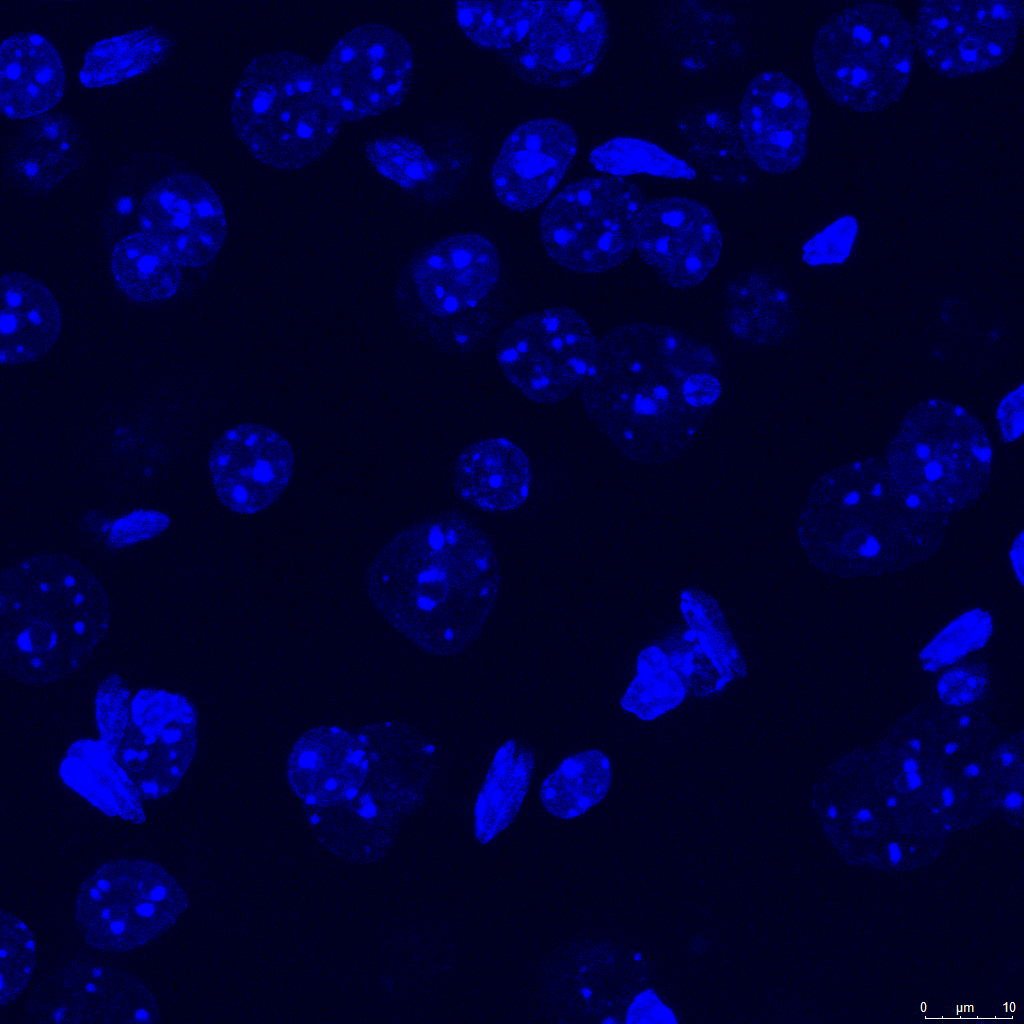

Supplement: Supplementary file 7 — Source data Fig. 2 [file 44319_2024_218_MOESM7_ESM.zip › Figure 2/2F/Ctx/Het, S2 Cortex, GFAP488-G, IMPDH555-R, NeuN633-W, ZS 40xZF2,5-I_ch00.tif]

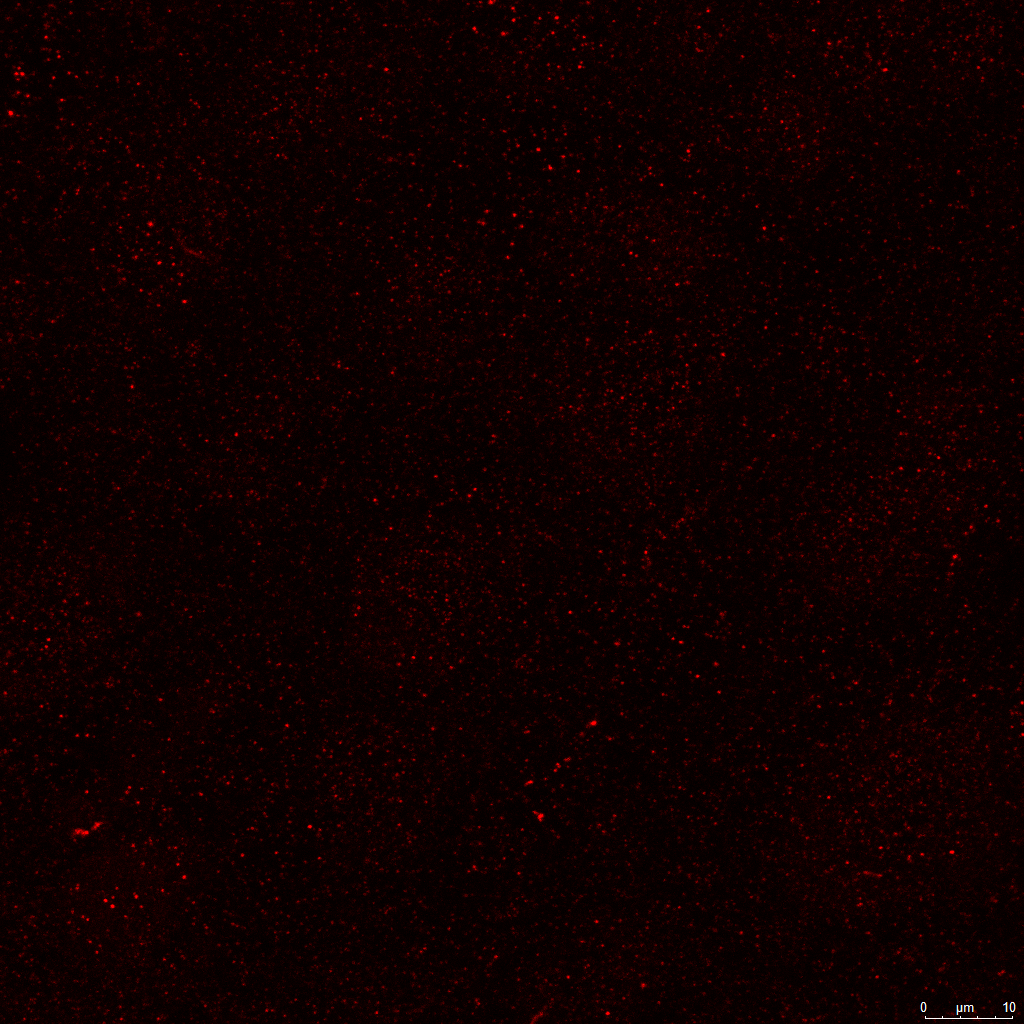

Supplement: Supplementary file 7 — Source data Fig. 2 [file 44319_2024_218_MOESM7_ESM.zip › Figure 2/2F/Ctx/Het, S2 Cortex, GFAP488-G, IMPDH555-R, NeuN633-W, ZS 40xZF2,5-I_ch02.tif]

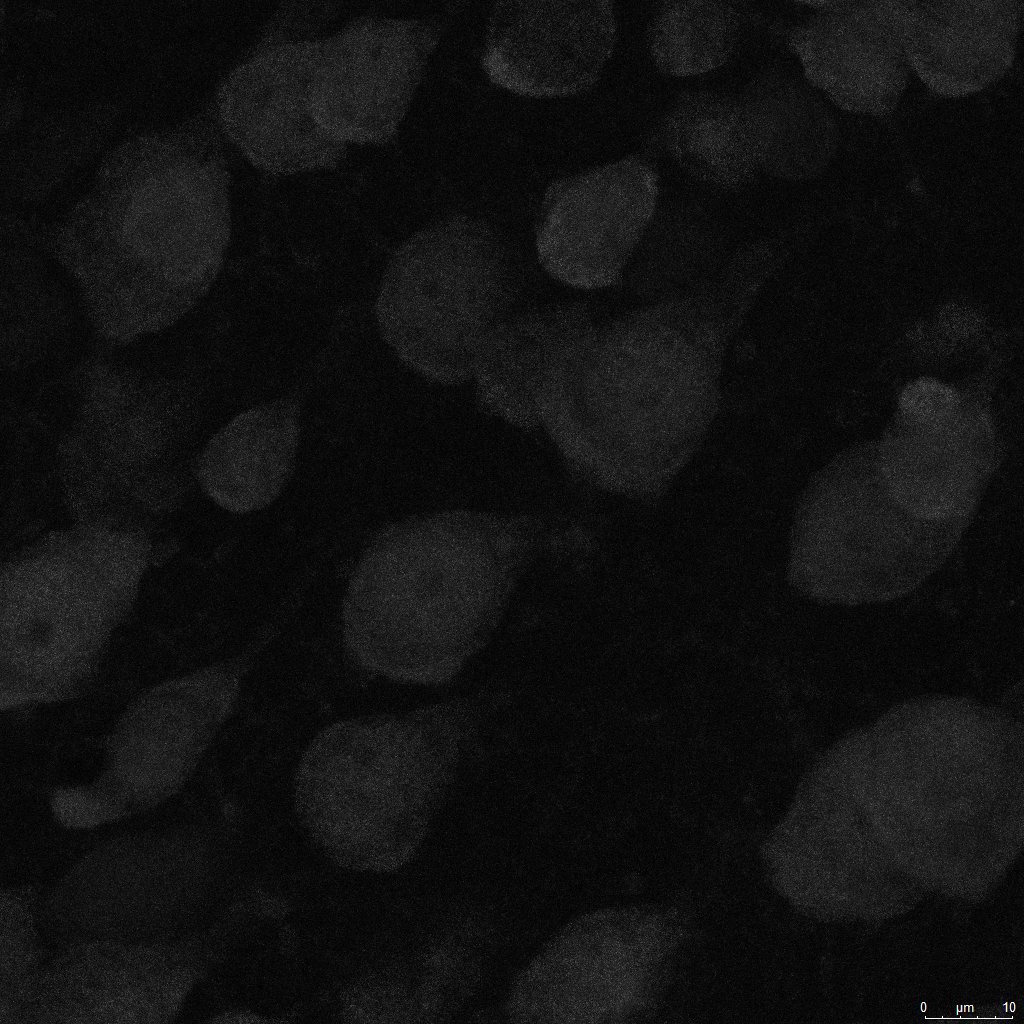

Supplement: Supplementary file 7 — Source data Fig. 2 [file 44319_2024_218_MOESM7_ESM.zip › Figure 2/2F/Ctx/Het, S2 Cortex, GFAP488-G, IMPDH555-R, NeuN633-W, ZS 40xZF2,5-I_ch03.tif]

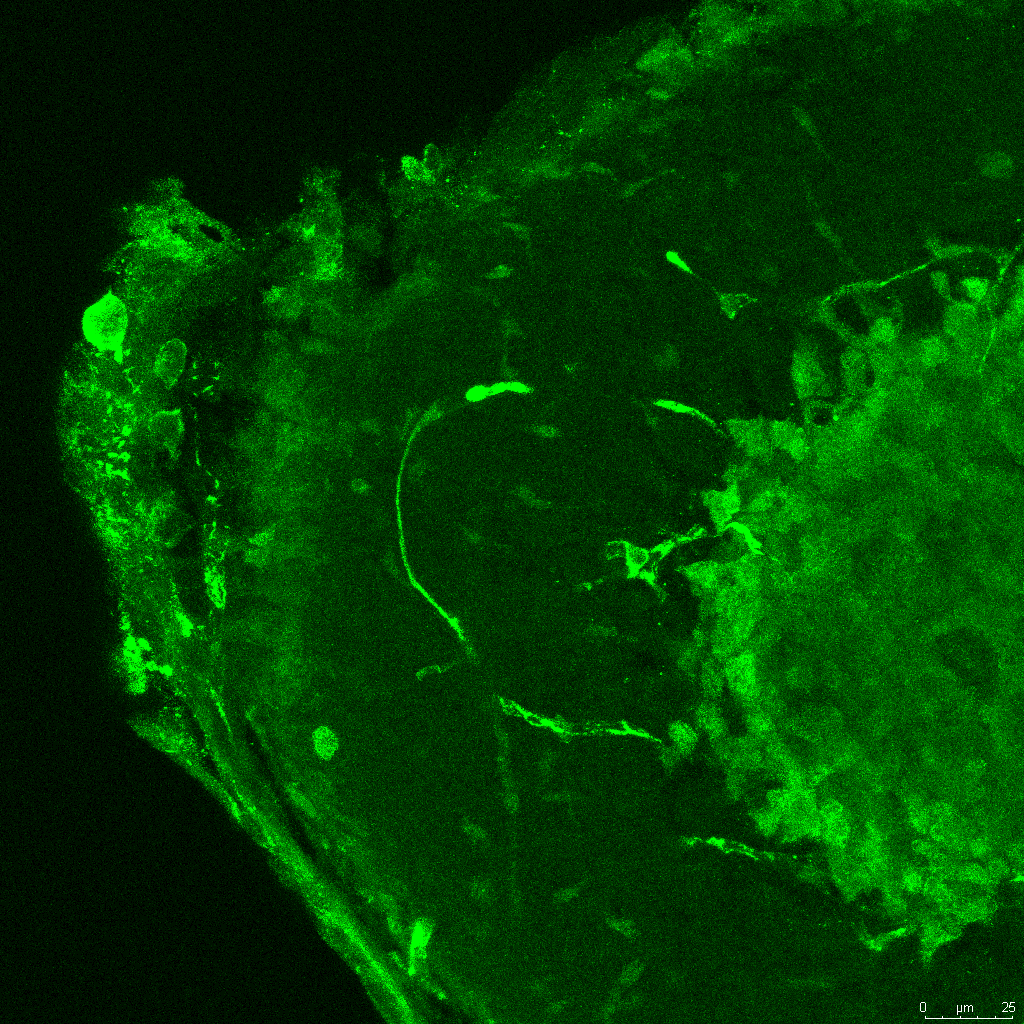

Supplement: Supplementary file 7 — Source data Fig. 2 [file 44319_2024_218_MOESM7_ESM.zip › Figure 2/2F/Cb/dKO, CB L3, NeuN488-G, IMPDH555-R, Ki633-W, ZS 40xZF1_ch01.tif]

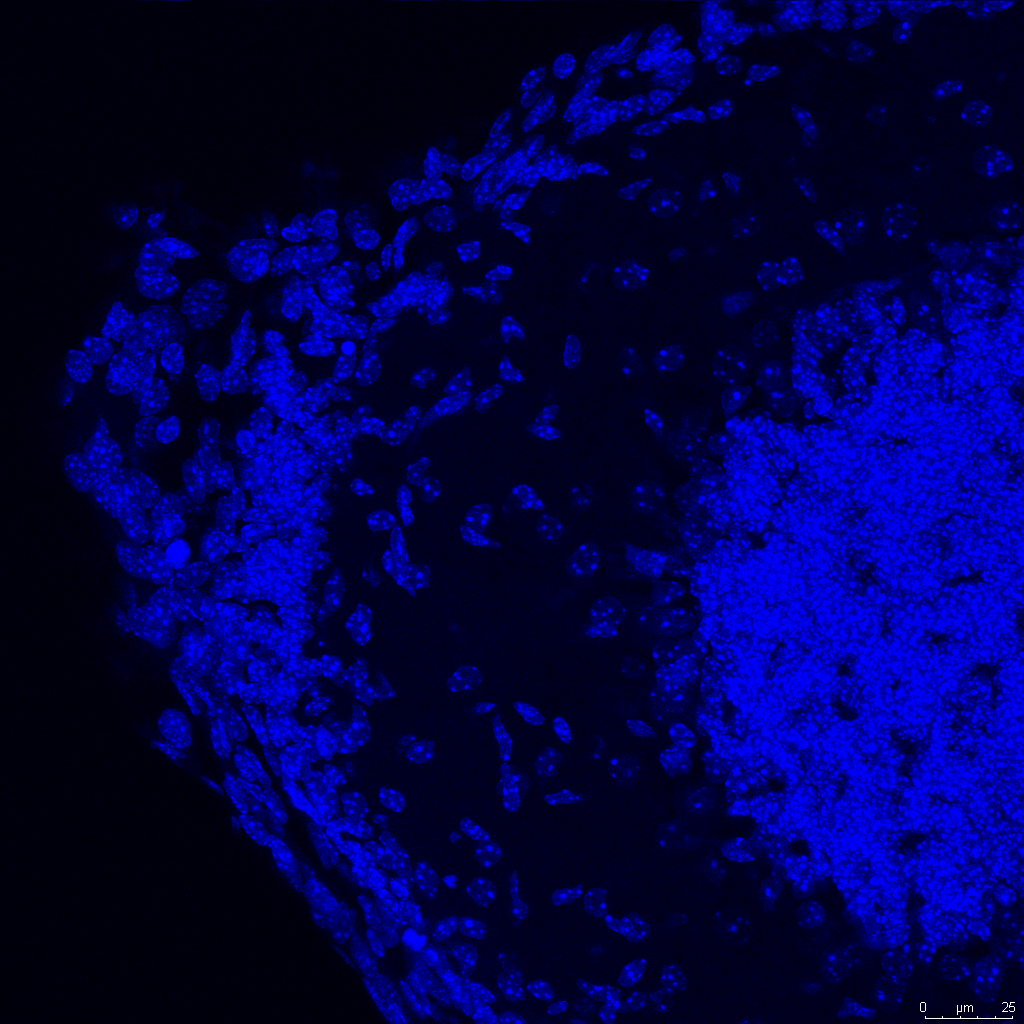

Supplement: Supplementary file 7 — Source data Fig. 2 [file 44319_2024_218_MOESM7_ESM.zip › Figure 2/2F/Cb/dKO, CB L3, NeuN488-G, IMPDH555-R, Ki633-W, ZS 40xZF1_ch00.tif]

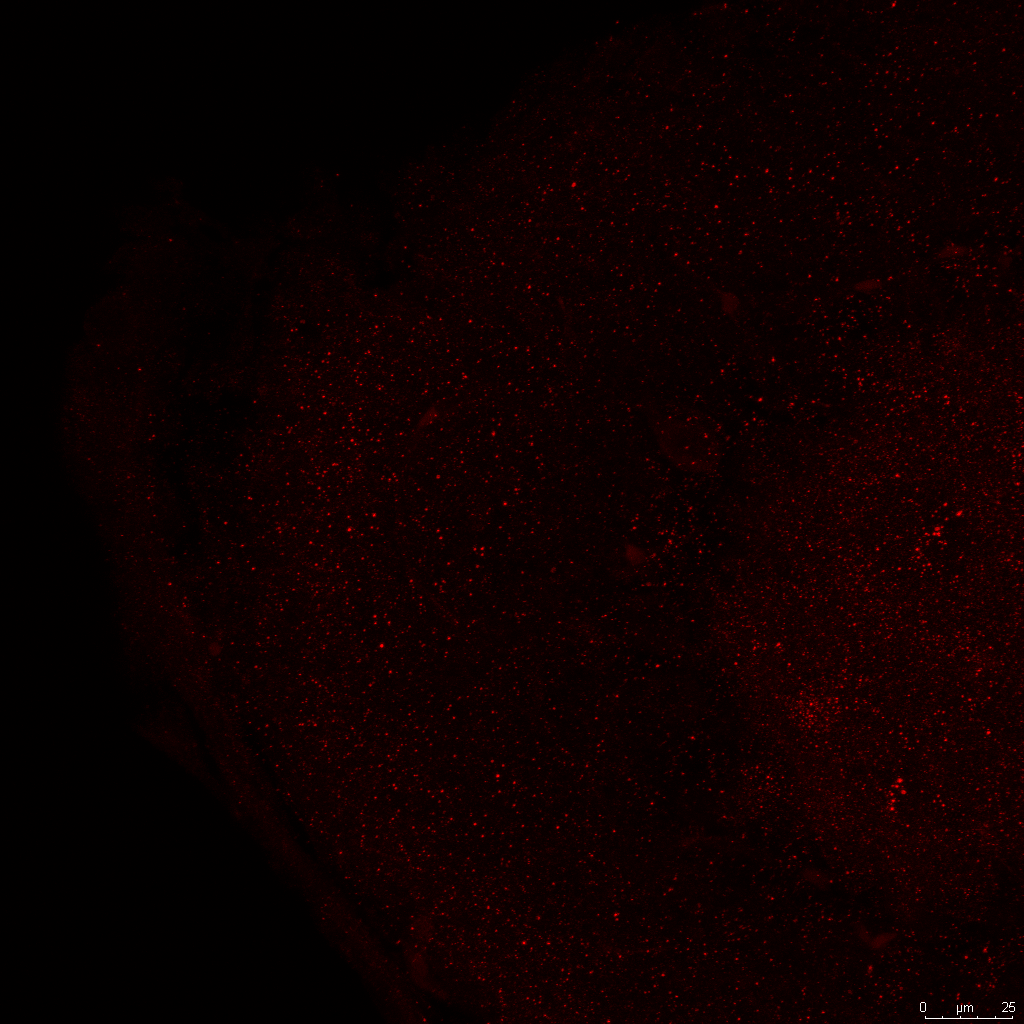

Supplement: Supplementary file 7 — Source data Fig. 2 [file 44319_2024_218_MOESM7_ESM.zip › Figure 2/2F/Cb/dKO, CB L3, NeuN488-G, IMPDH555-R, Ki633-W, ZS 40xZF1_ch02.tif]

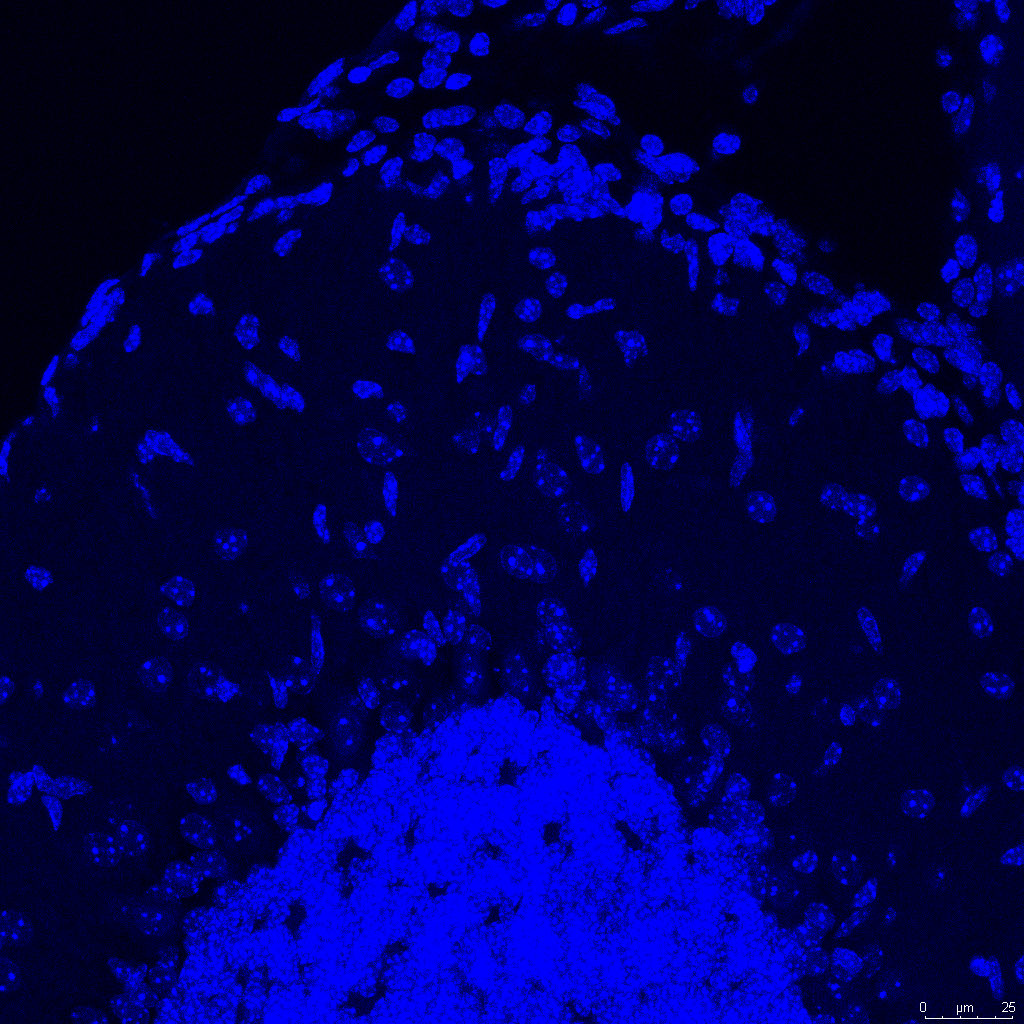

Supplement: Supplementary file 7 — Source data Fig. 2 [file 44319_2024_218_MOESM7_ESM.zip › Figure 2/2F/Cb/Het, CB No1 L3, NeuN488-G, IMPDH555-R, Ki633-W, ZS 40xZF1-II_ch00.tif]

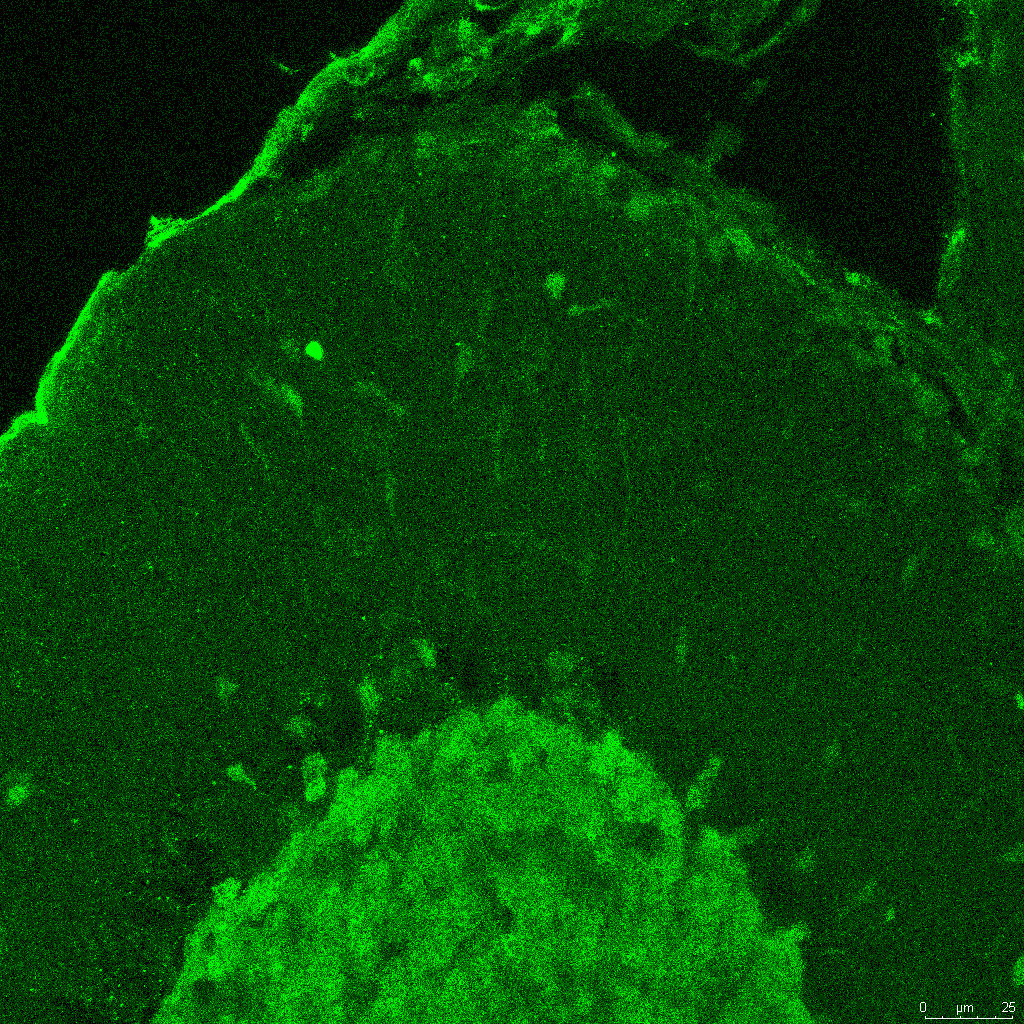

Supplement: Supplementary file 7 — Source data Fig. 2 [file 44319_2024_218_MOESM7_ESM.zip › Figure 2/2F/Cb/Het, CB No1 L3, NeuN488-G, IMPDH555-R, Ki633-W, ZS 40xZF1-II_ch01.tif]

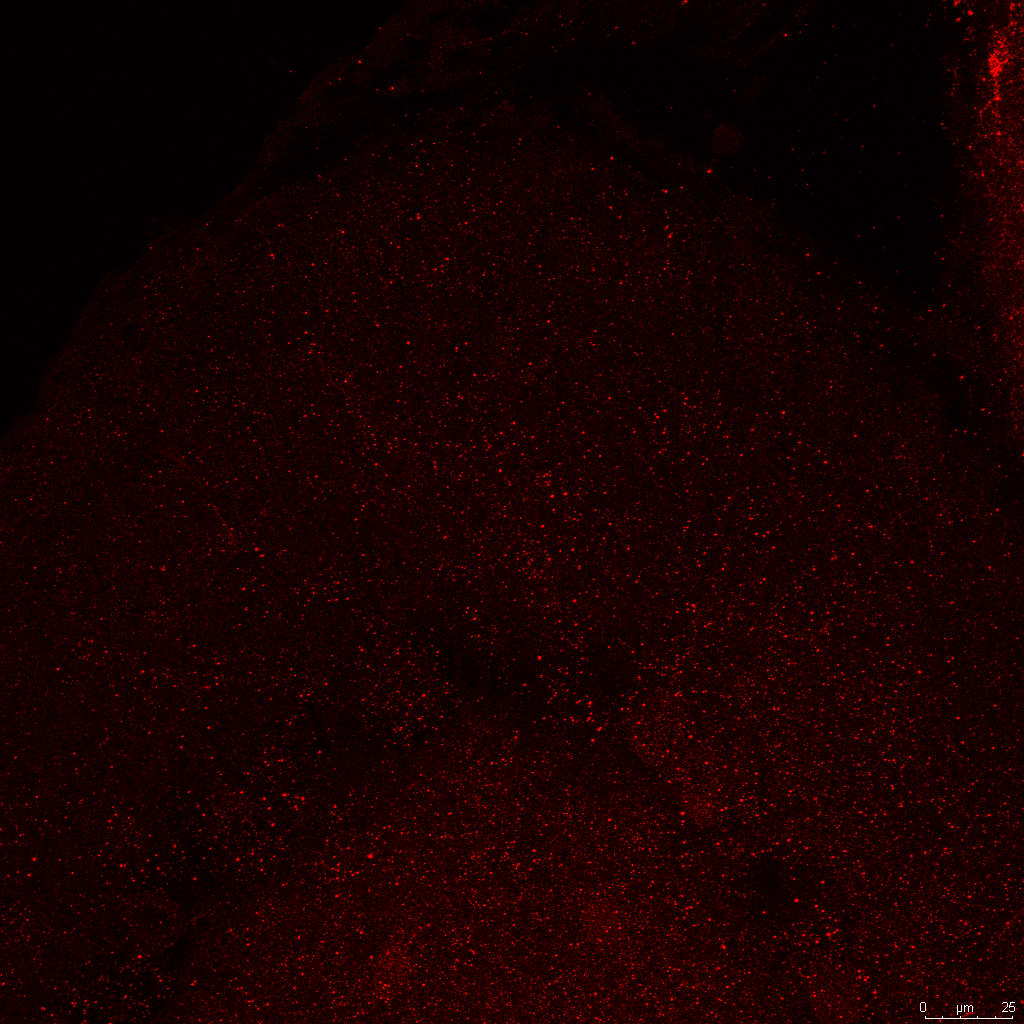

Supplement: Supplementary file 7 — Source data Fig. 2 [file 44319_2024_218_MOESM7_ESM.zip › Figure 2/2F/Cb/Het, CB No1 L3, NeuN488-G, IMPDH555-R, Ki633-W, ZS 40xZF1-II_ch02.tif]

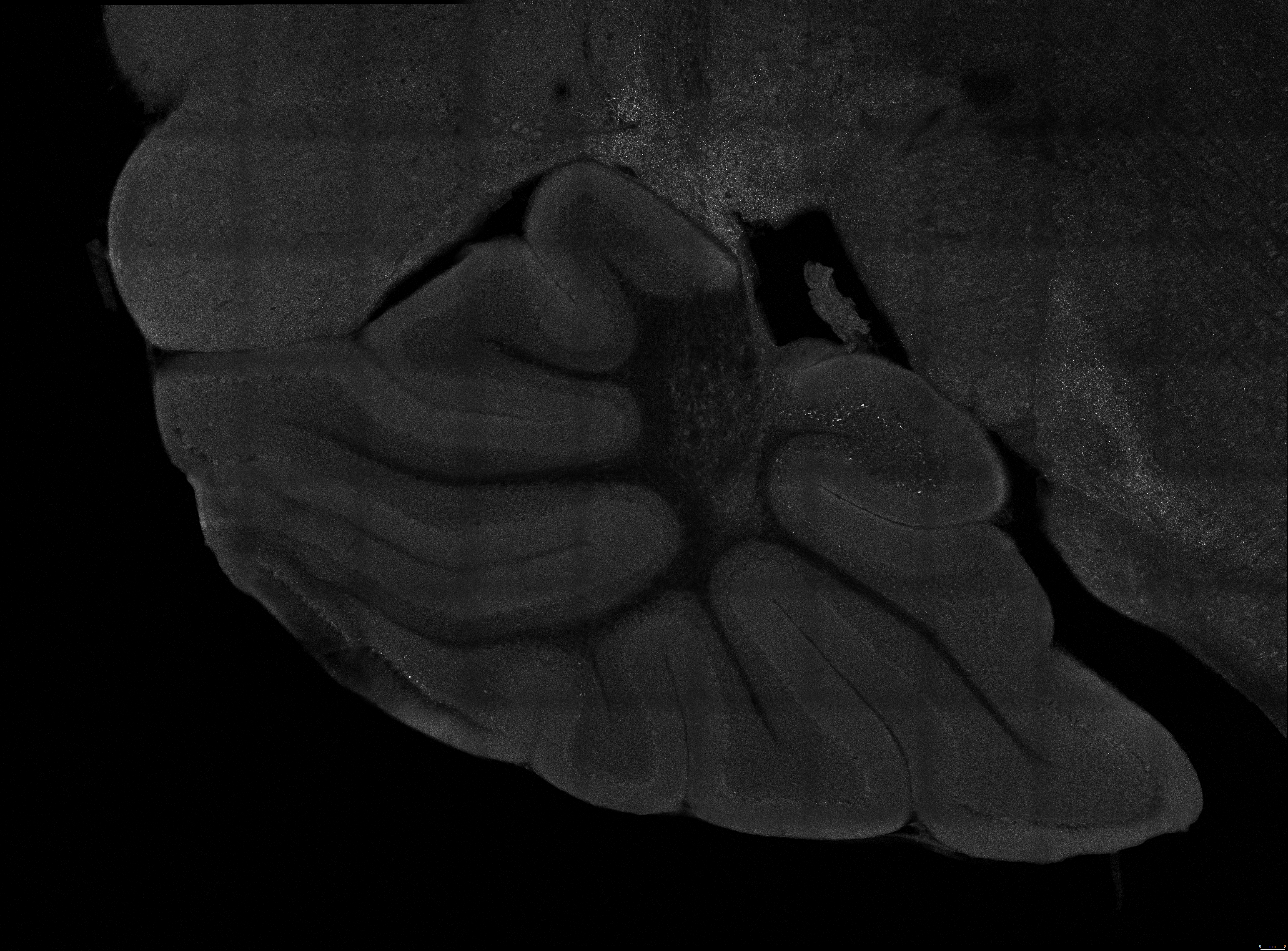

Supplement: Supplementary file 7 — Source data Fig. 2 [file 44319_2024_218_MOESM7_ESM.zip › Figure 2/2E/Ctrl/TileScan 002_598Het, Cb, CD68 488G, Casp7 555R, IMPDH2 633W, TS ZS 20X-1_ch02.tif]

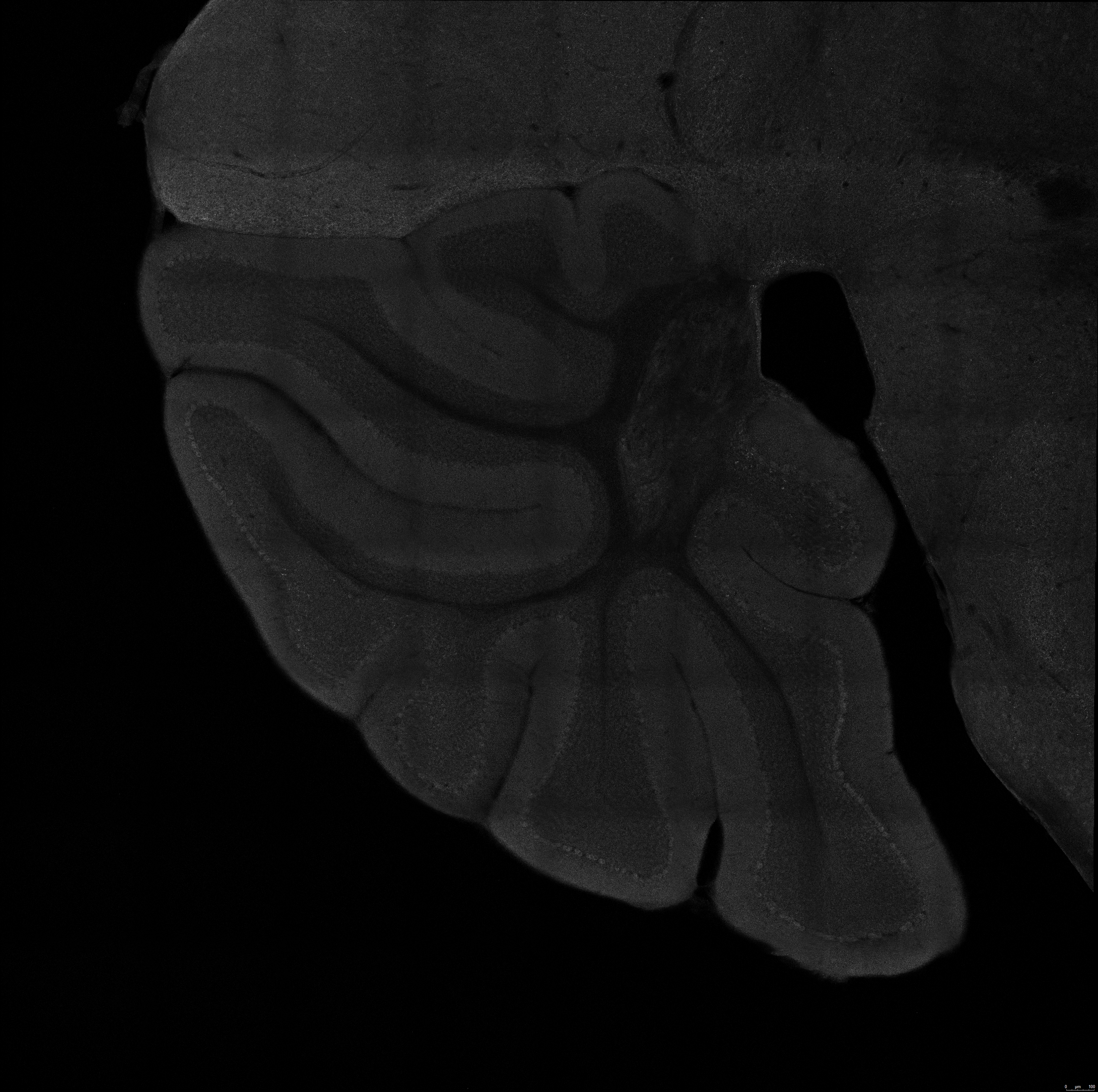

Supplement: Supplementary file 7 — Source data Fig. 2 [file 44319_2024_218_MOESM7_ESM.zip › Figure 2/2E/dKO/TileScan 001_614dKO, Cb, CD68 488G, Casp7 555R, IMPDH2 633W, TS ZS 20X-1_ch02.tif]

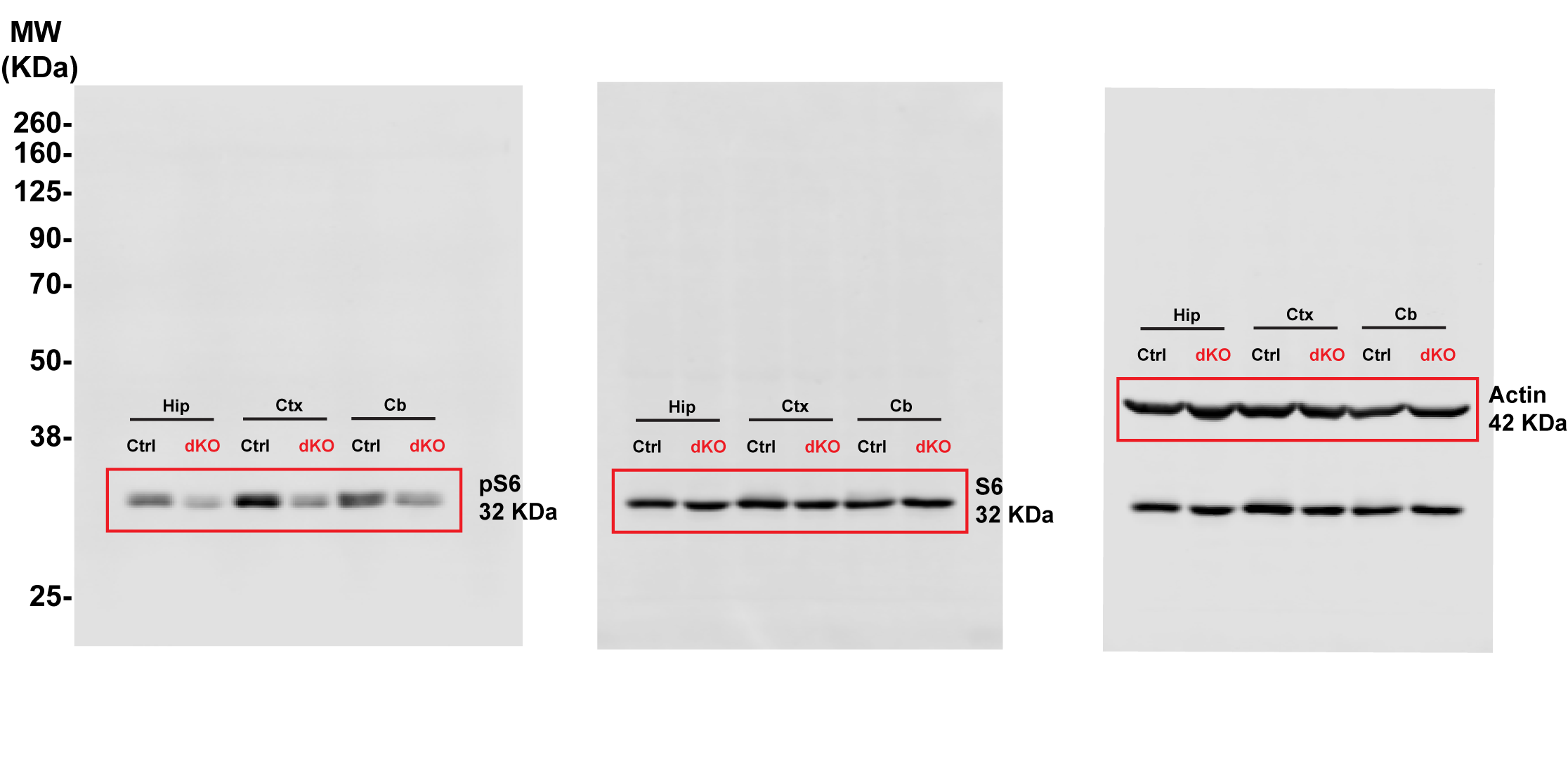

Supplement: Supplementary file 8 — Source data Fig. 3 [file 44319_2024_218_MOESM8_ESM.zip › Figure 3/3E/WB 3E.tif]

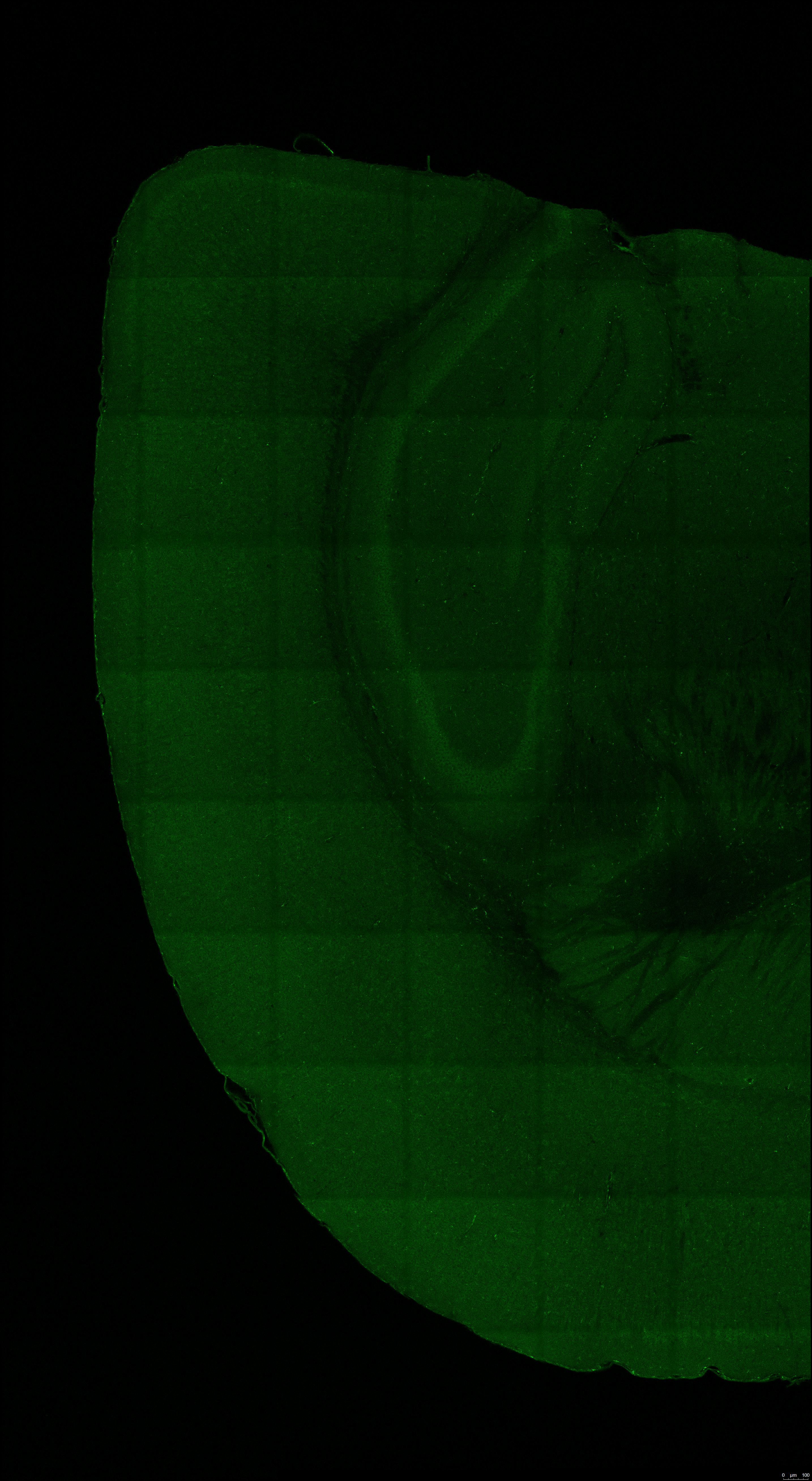

Supplement: Supplementary file 9 — Source data Fig. 4 [file 44319_2024_218_MOESM9_ESM.zip › Figure 4/4A/TileScan 002_Het, CD68 488G, IMPDH2 555R, NEUN 633W, TS ZS 20X ZFI-II_ch01.tif]

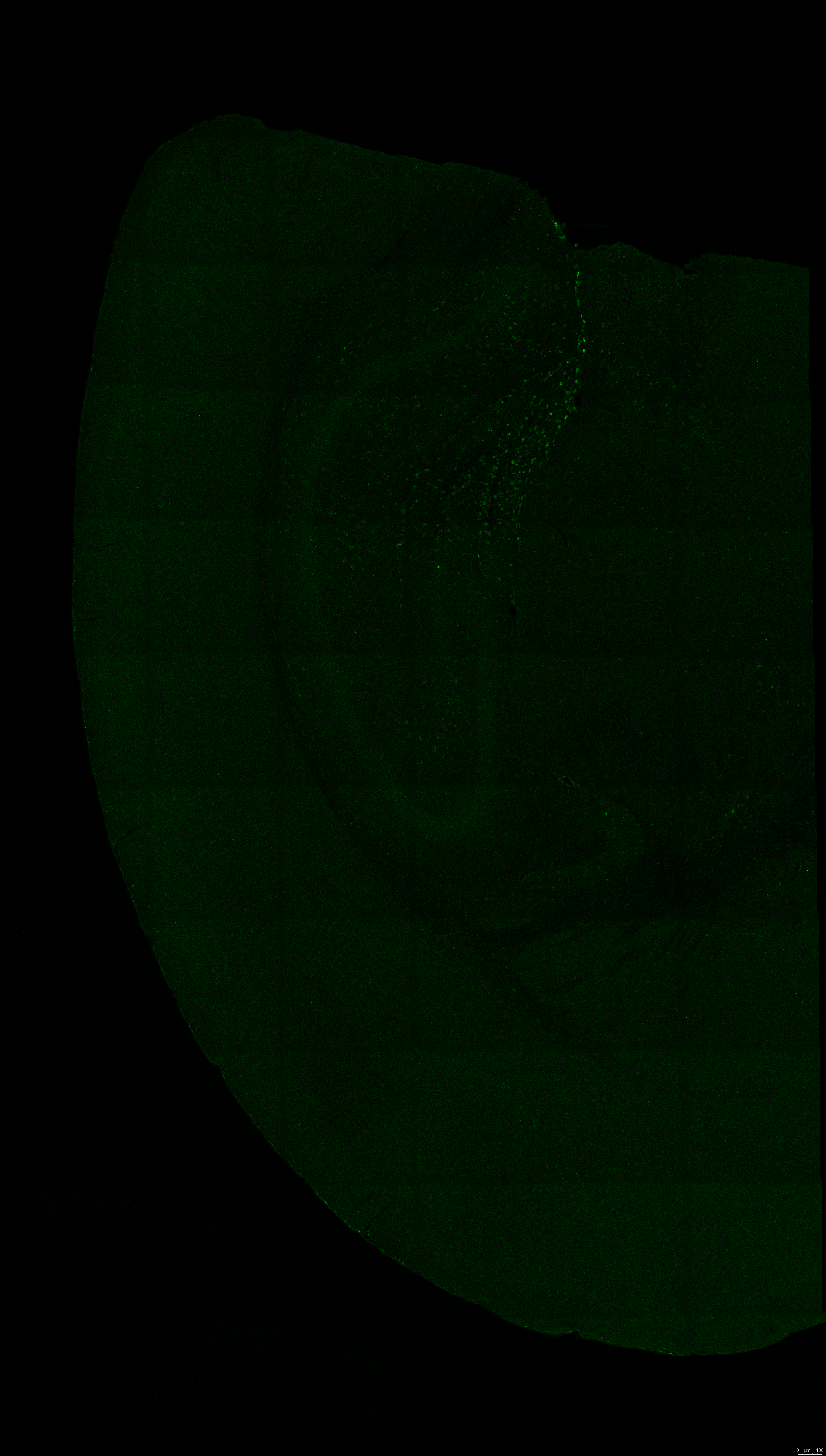

Supplement: Supplementary file 9 — Source data Fig. 4 [file 44319_2024_218_MOESM9_ESM.zip › Figure 4/4A/TileScan 001_dKO, CD68 488G, IMPDH2 555R, NEUN 633W, TS ZS 20X ZF1_ch01.tif]

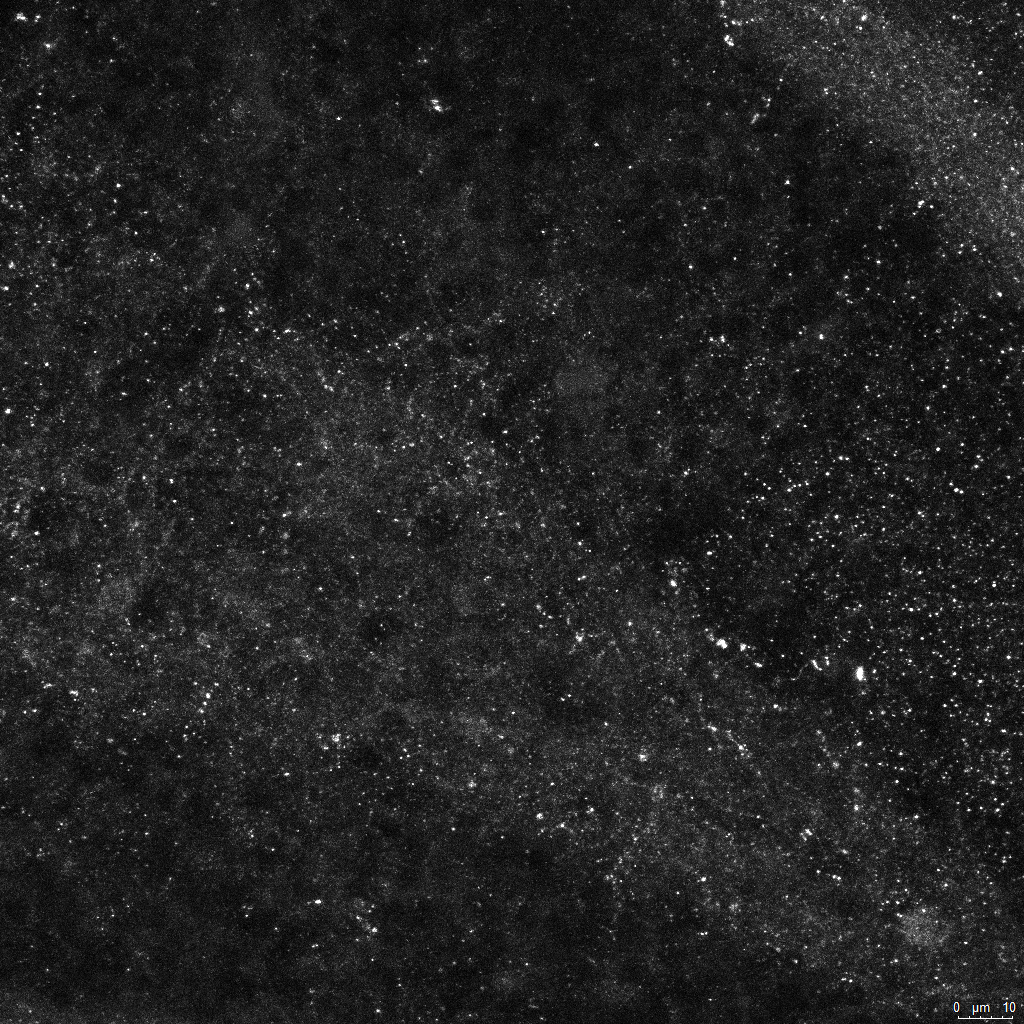

Supplement: Supplementary file 9 — Source data Fig. 4 [file 44319_2024_218_MOESM9_ESM.zip › Figure 4/4B/dKO DG/dKO, Hip, CD68 488G, Casp7 555R, IMPDH2 633W, ZS 63X-7_ch02.tif]

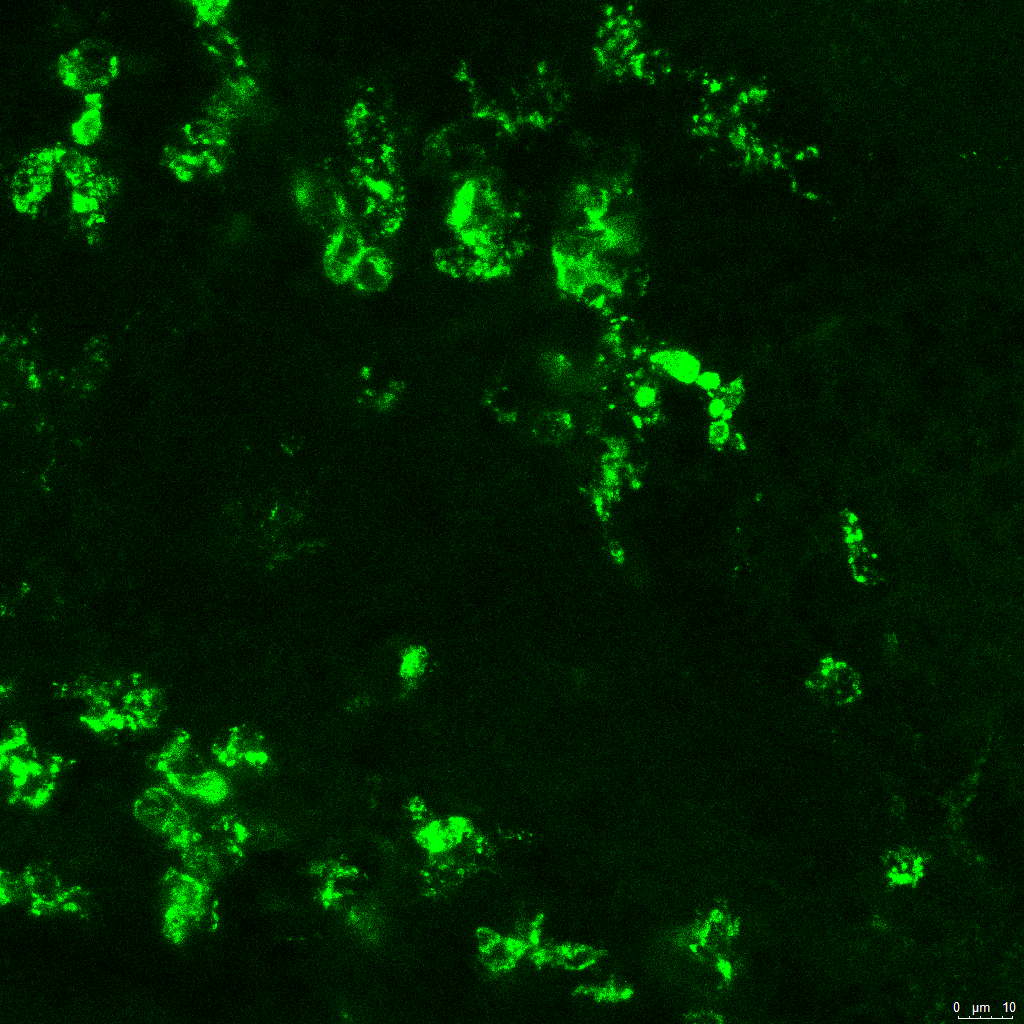

Supplement: Supplementary file 9 — Source data Fig. 4 [file 44319_2024_218_MOESM9_ESM.zip › Figure 4/4B/dKO DG/dKO, Hip, CD68 488G, Casp7 555R, IMPDH2 633W, ZS 63X-7_ch01.tif]

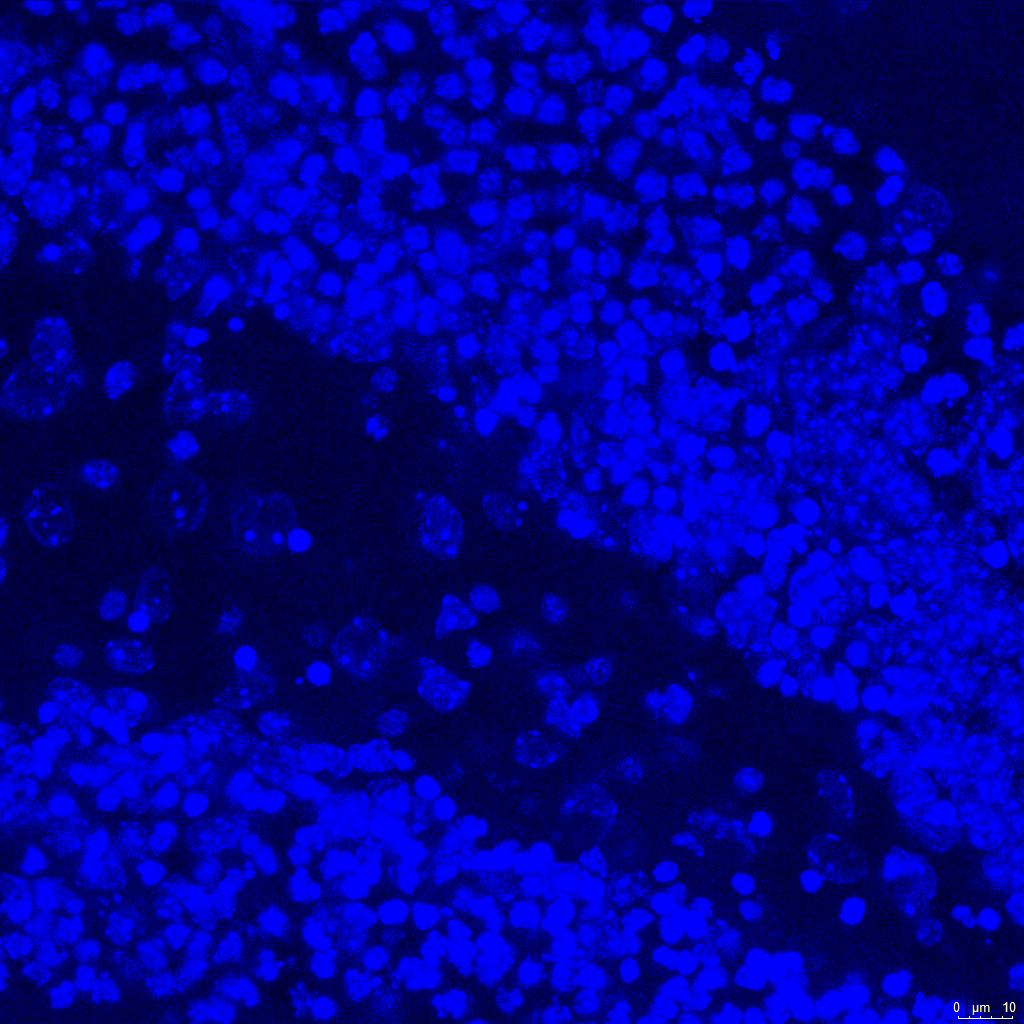

Supplement: Supplementary file 9 — Source data Fig. 4 [file 44319_2024_218_MOESM9_ESM.zip › Figure 4/4B/dKO DG/dKO, Hip, CD68 488G, Casp7 555R, IMPDH2 633W, ZS 63X-7_ch00.tif]

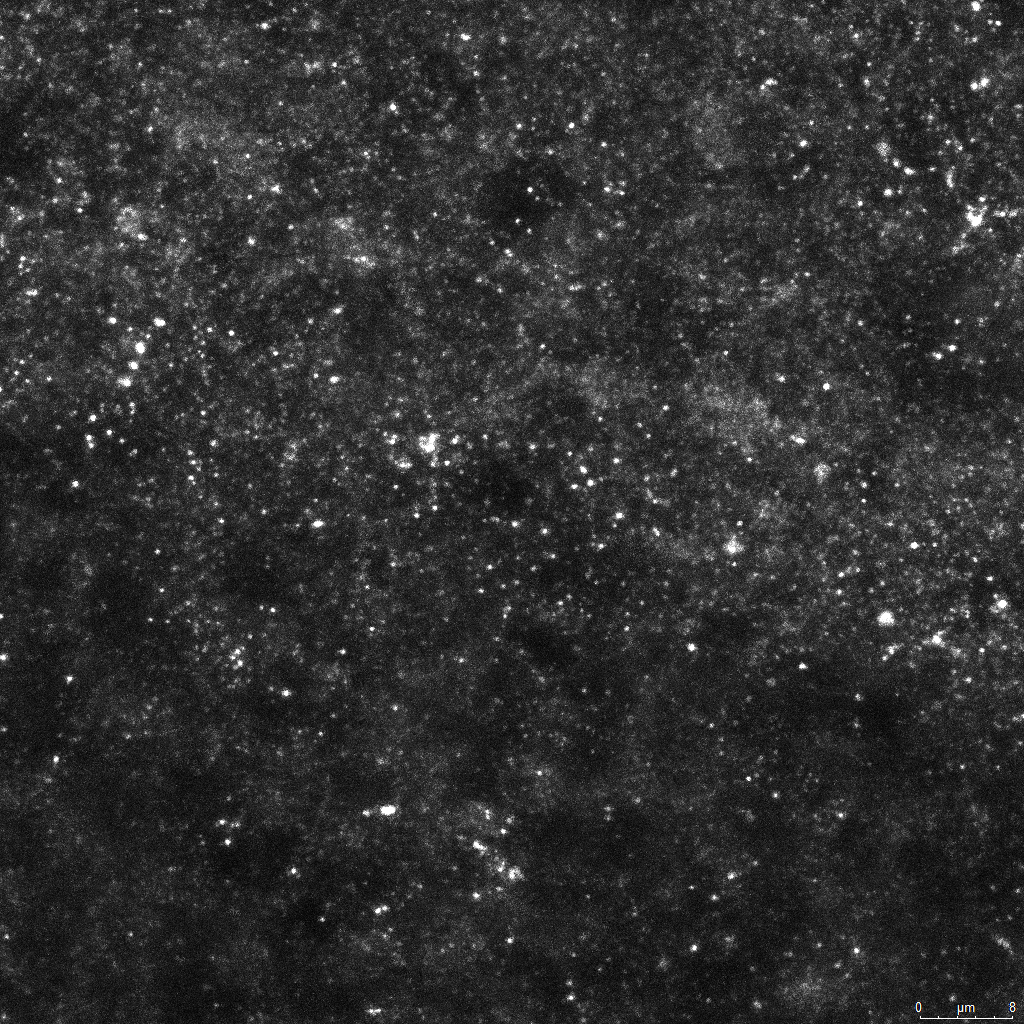

Supplement: Supplementary file 9 — Source data Fig. 4 [file 44319_2024_218_MOESM9_ESM.zip › Figure 4/4B/dKO DG/dKO, Hip, CD68 488G, Casp7 555R, IMPDH2 633W, ZS 63X-9_ch02.tif]

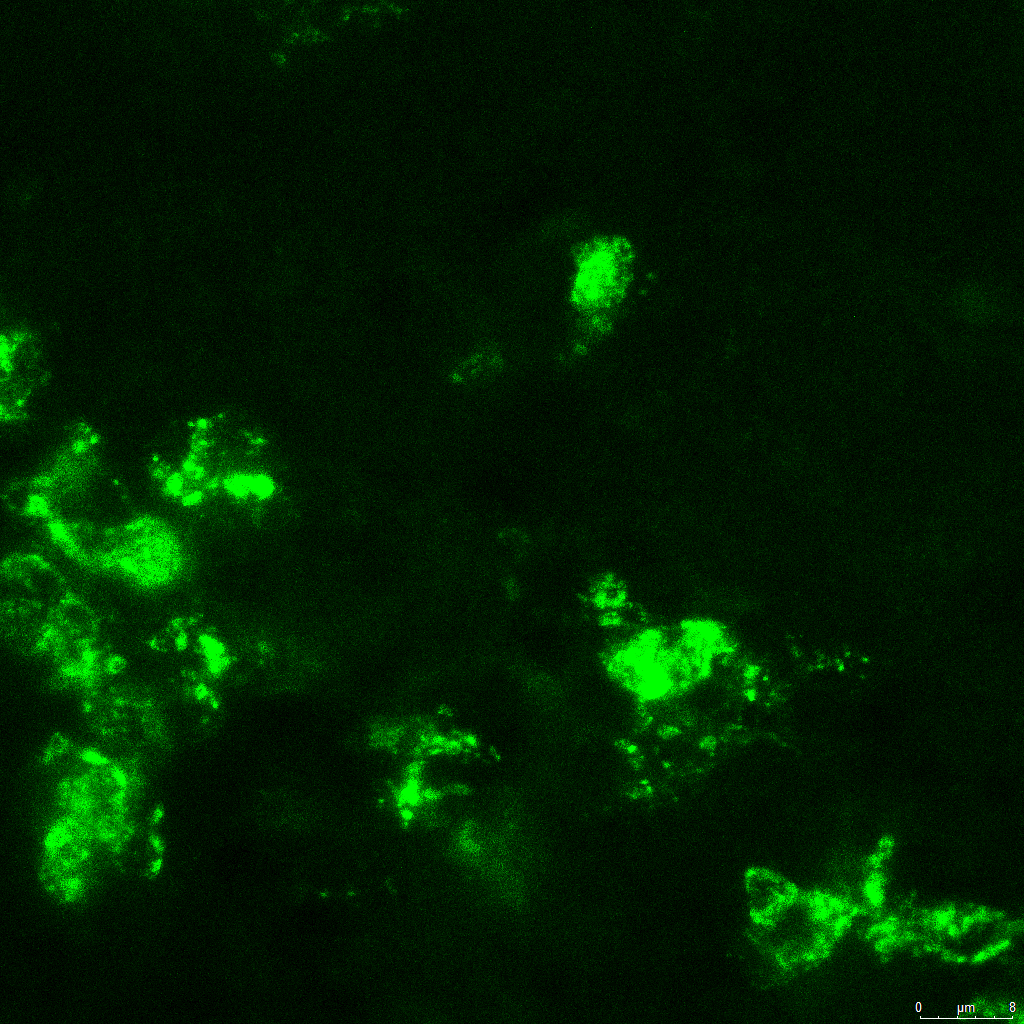

Supplement: Supplementary file 9 — Source data Fig. 4 [file 44319_2024_218_MOESM9_ESM.zip › Figure 4/4B/dKO DG/dKO, Hip, CD68 488G, Casp7 555R, IMPDH2 633W, ZS 63X-9_ch01.tif]

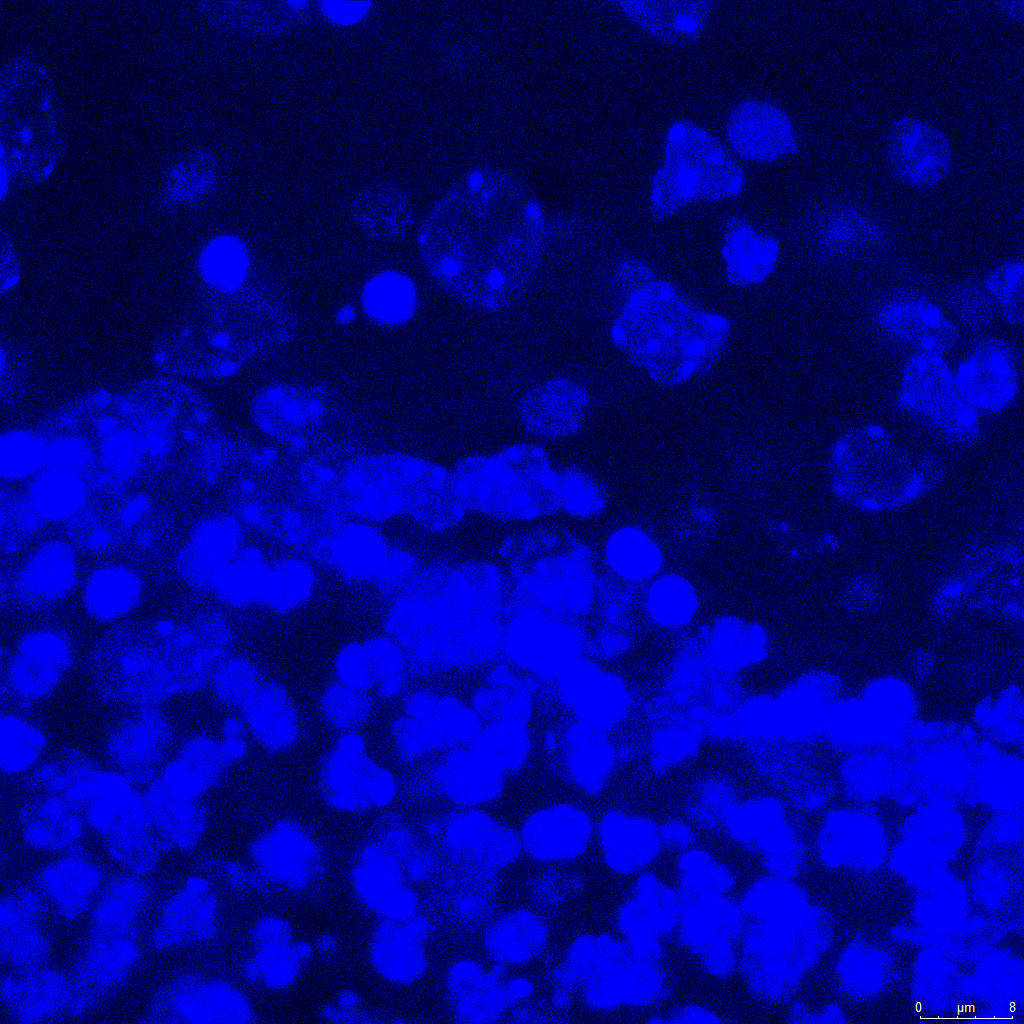

Supplement: Supplementary file 9 — Source data Fig. 4 [file 44319_2024_218_MOESM9_ESM.zip › Figure 4/4B/dKO DG/dKO, Hip, CD68 488G, Casp7 555R, IMPDH2 633W, ZS 63X-9_ch00.tif]

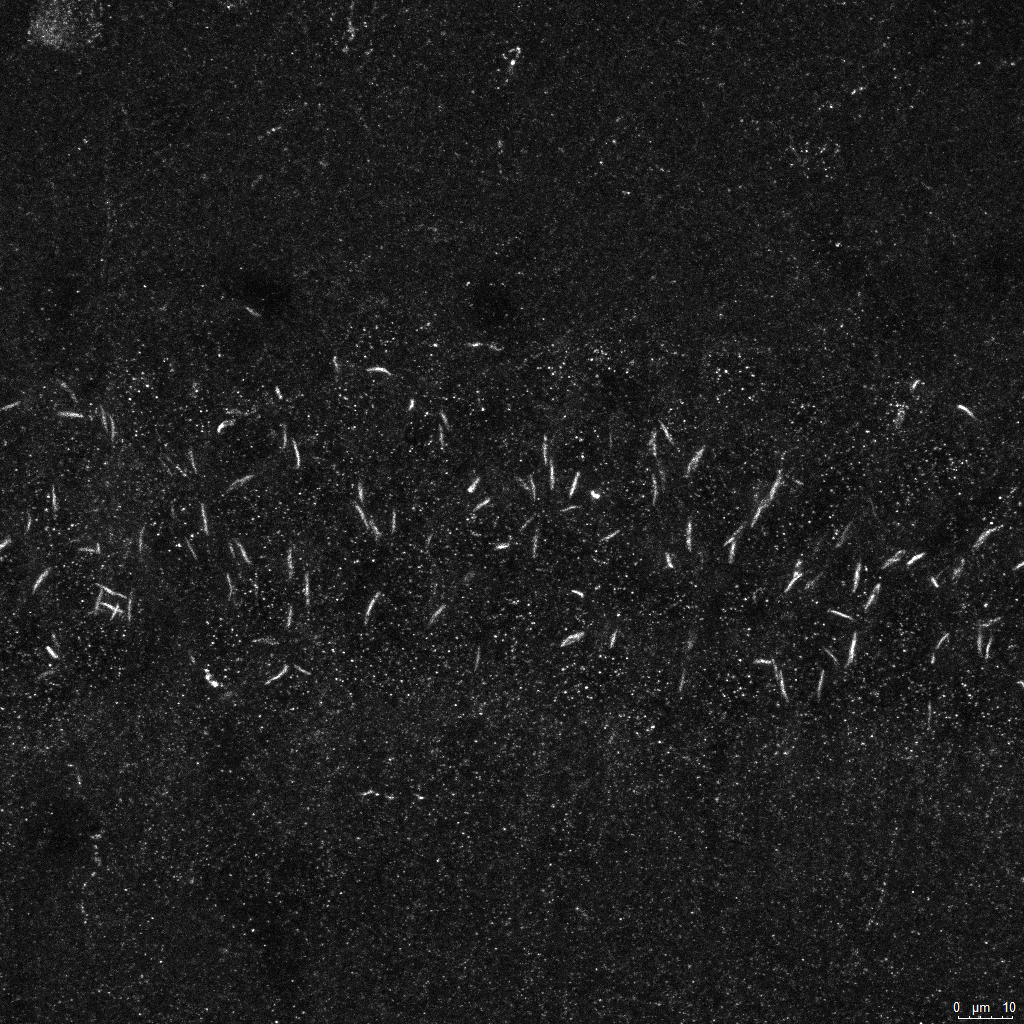

Supplement: Supplementary file 9 — Source data Fig. 4 [file 44319_2024_218_MOESM9_ESM.zip › Figure 4/4B/dKO CA1/dKO, Hip, CD68 488G, Casp7 555R, IMPDH2 633W, ZS 63X-1_ch02.tif]

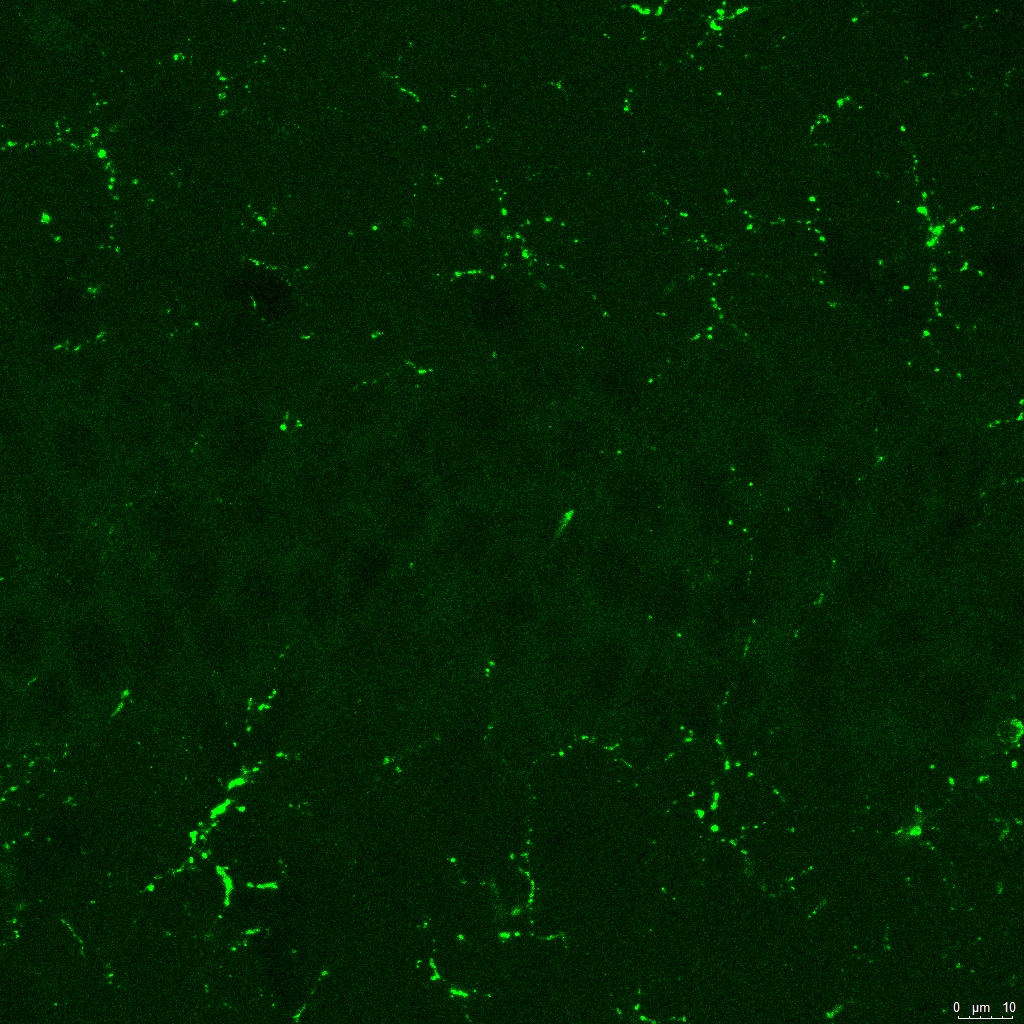

Supplement: Supplementary file 9 — Source data Fig. 4 [file 44319_2024_218_MOESM9_ESM.zip › Figure 4/4B/dKO CA1/dKO, Hip, CD68 488G, Casp7 555R, IMPDH2 633W, ZS 63X-1_ch01.tif]

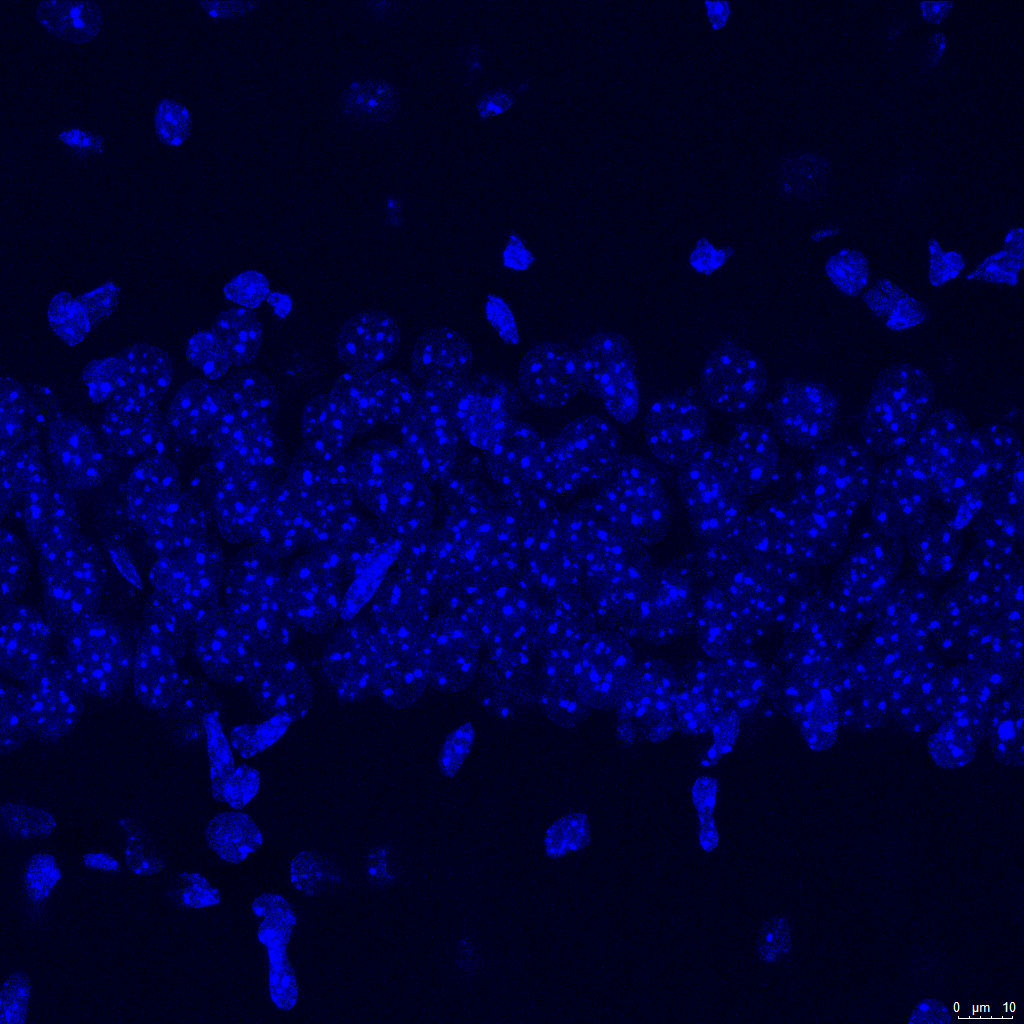

Supplement: Supplementary file 9 — Source data Fig. 4 [file 44319_2024_218_MOESM9_ESM.zip › Figure 4/4B/dKO CA1/dKO, Hip, CD68 488G, Casp7 555R, IMPDH2 633W, ZS 63X-1_ch00.tif]

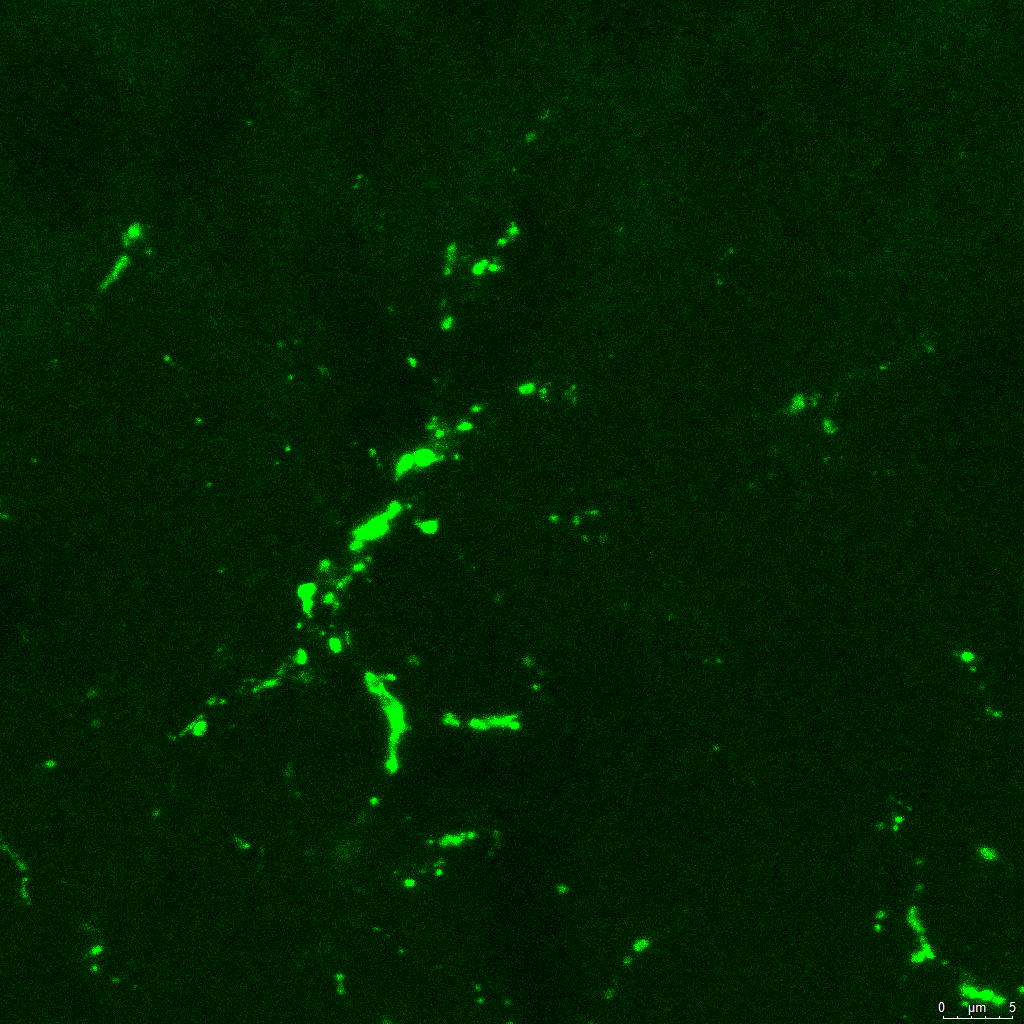

Supplement: Supplementary file 9 — Source data Fig. 4 [file 44319_2024_218_MOESM9_ESM.zip › Figure 4/4B/dKO CA1/dKO, Hip, CD68 488G, Casp7 555R, IMPDH2 633W, ZS 63X-2_ch01.tif]

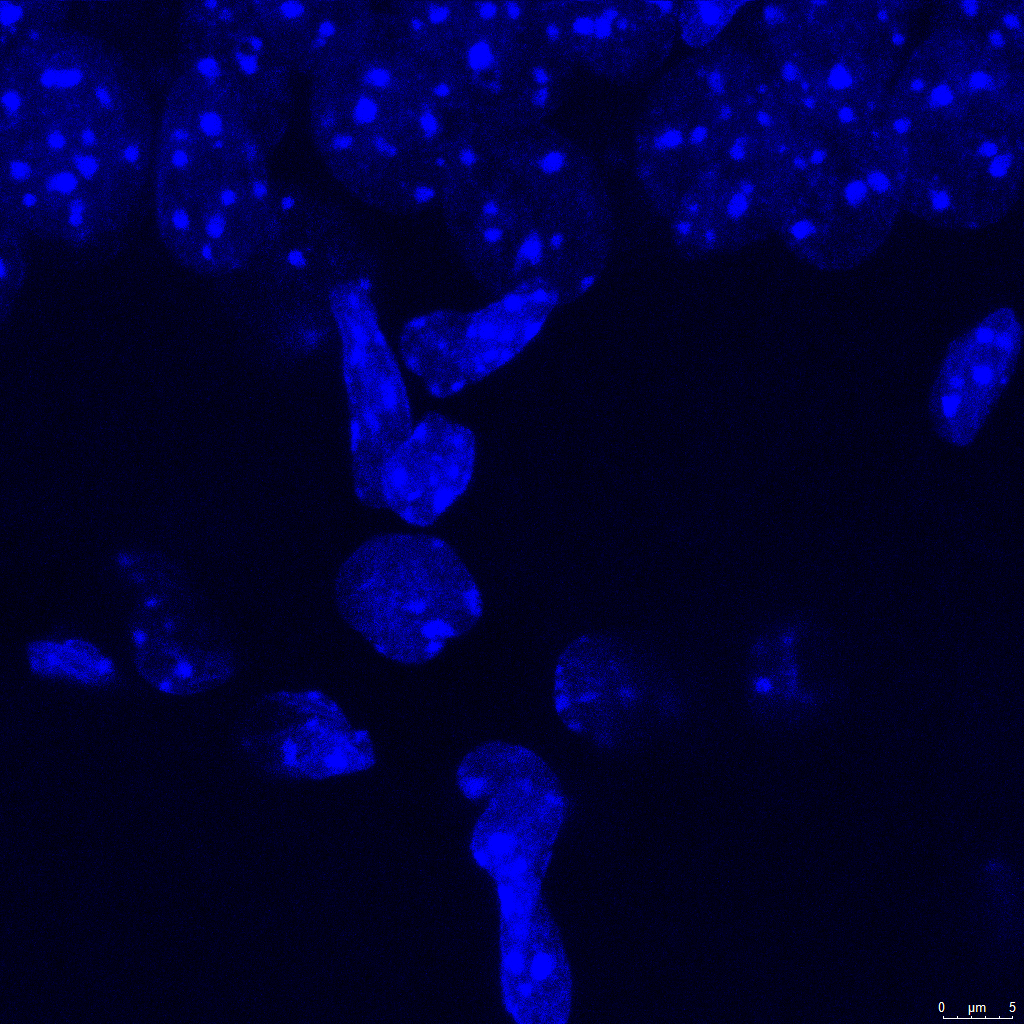

Supplement: Supplementary file 9 — Source data Fig. 4 [file 44319_2024_218_MOESM9_ESM.zip › Figure 4/4B/dKO CA1/dKO, Hip, CD68 488G, Casp7 555R, IMPDH2 633W, ZS 63X-2_ch00.tif]

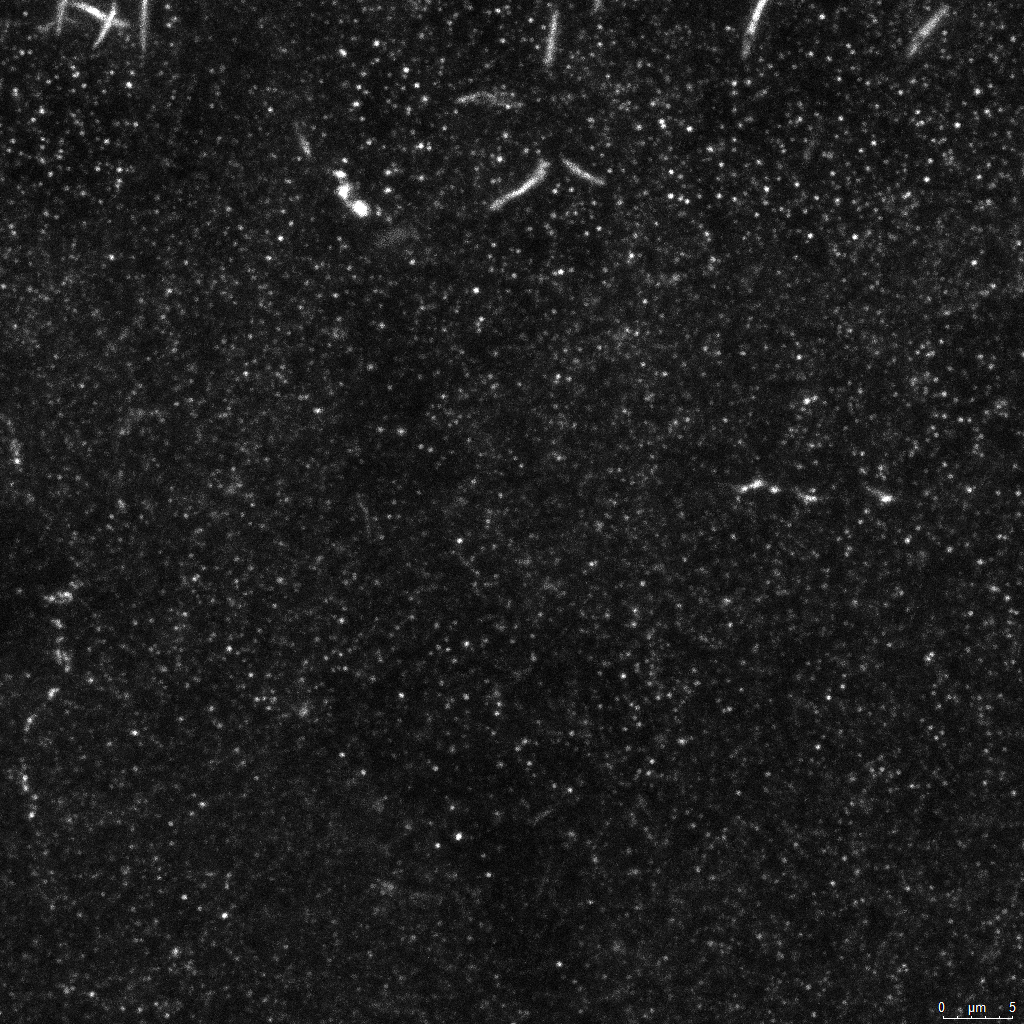

Supplement: Supplementary file 9 — Source data Fig. 4 [file 44319_2024_218_MOESM9_ESM.zip › Figure 4/4B/dKO CA1/dKO, Hip, CD68 488G, Casp7 555R, IMPDH2 633W, ZS 63X-2_ch02.tif]

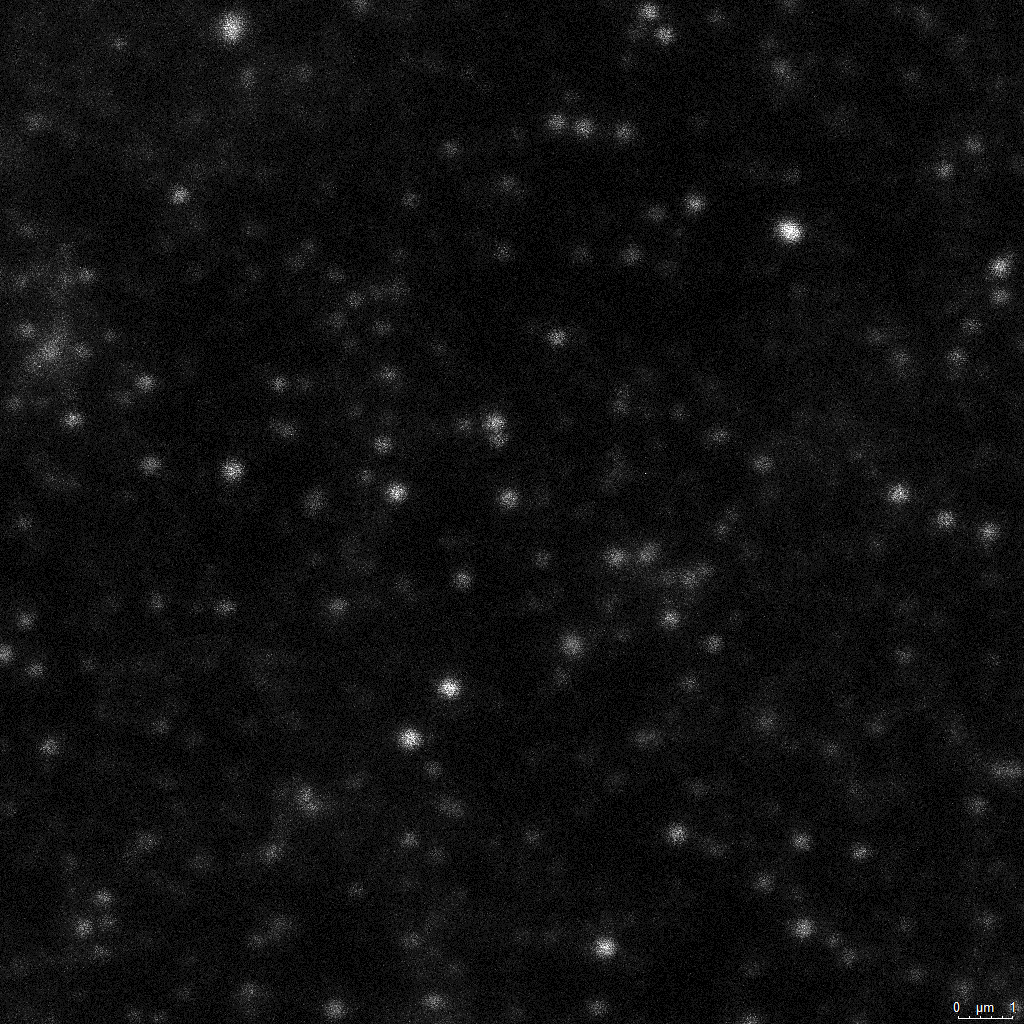

Supplement: Supplementary file 9 — Source data Fig. 4 [file 44319_2024_218_MOESM9_ESM.zip › Figure 4/4C/p10/dKO 898_dKO p10 898, DG, IMPDH2 488W, NEUN 568G, ZS 63X-2E_ch01.tif]

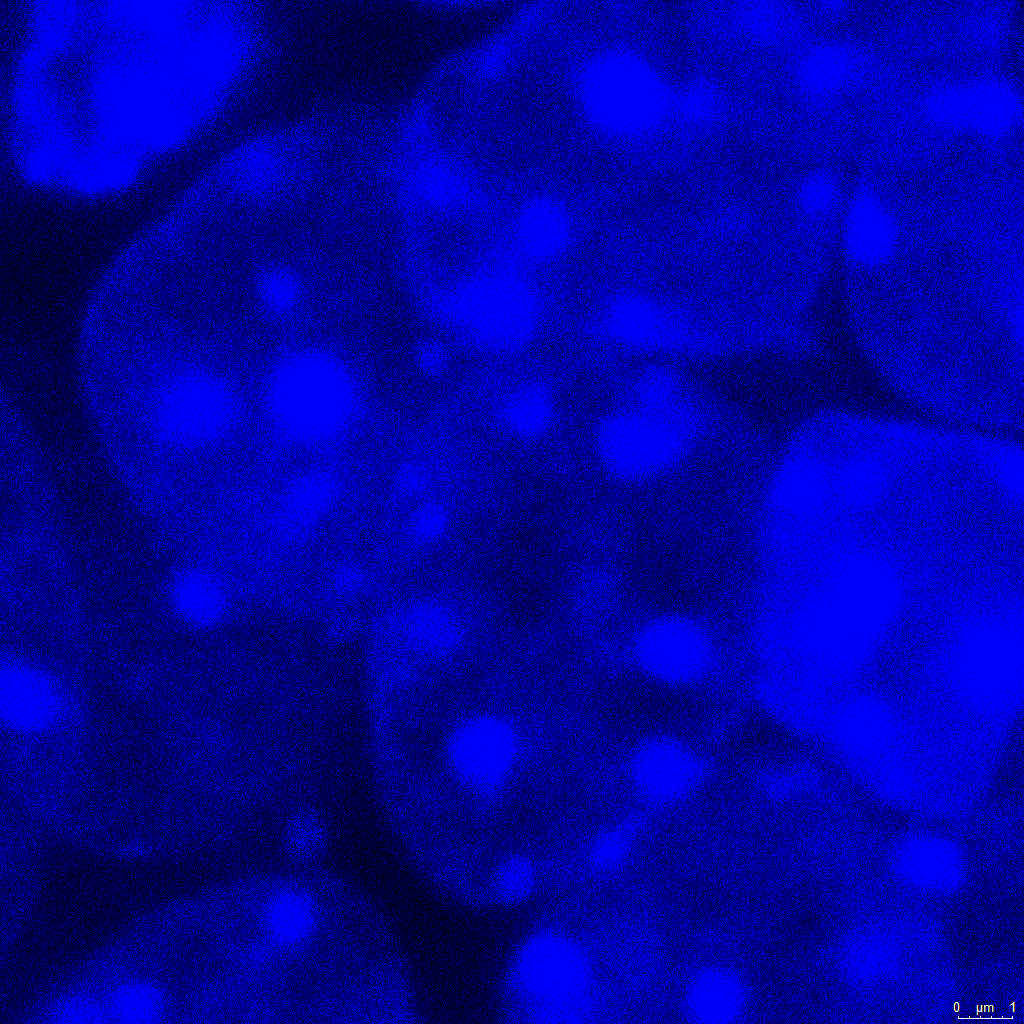

Supplement: Supplementary file 9 — Source data Fig. 4 [file 44319_2024_218_MOESM9_ESM.zip › Figure 4/4C/p10/dKO 898_dKO p10 898, DG, IMPDH2 488W, NEUN 568G, ZS 63X-2E_ch00.tif]

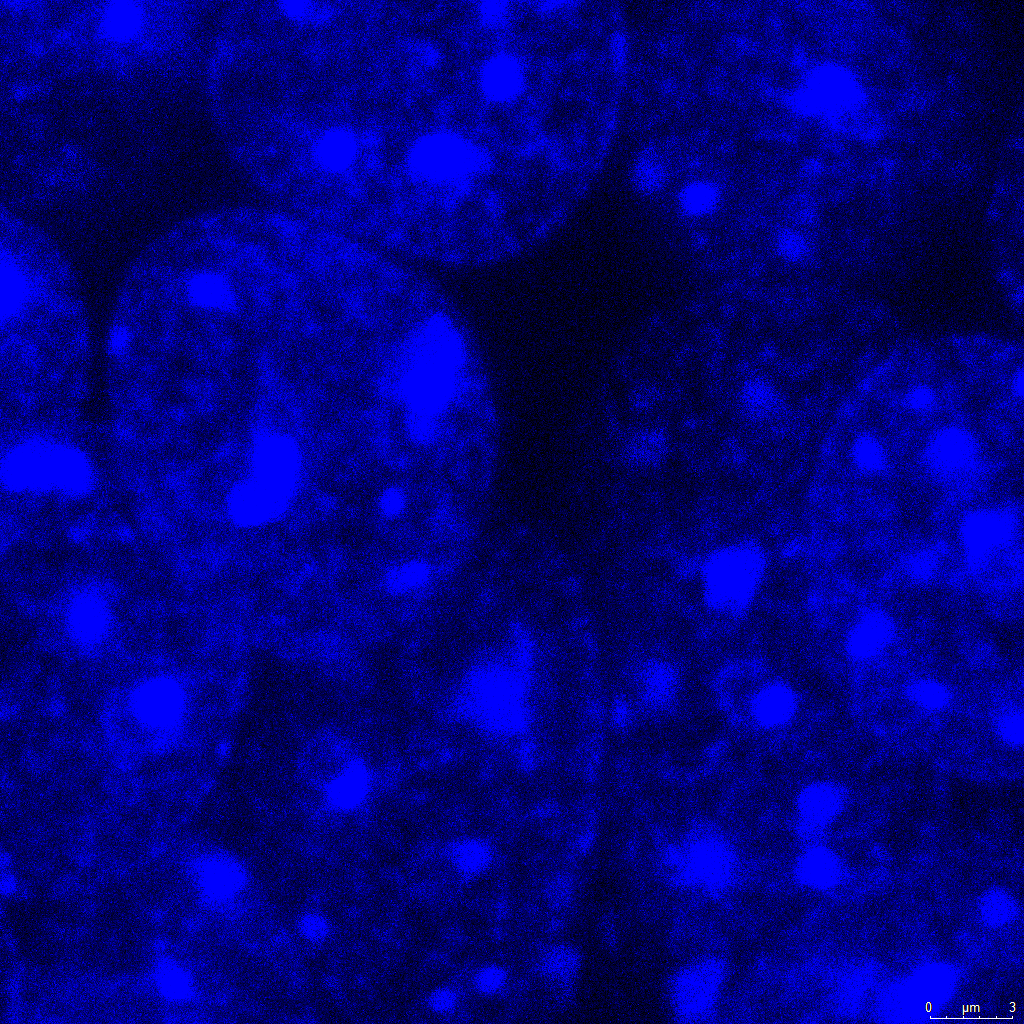

Supplement: Supplementary file 9 — Source data Fig. 4 [file 44319_2024_218_MOESM9_ESM.zip › Figure 4/4C/p10/dKO 898_dKO p10 898, CA1, IMPDH2 488W, NEUN 568G, ZS 63X-1D_ch00.tif]

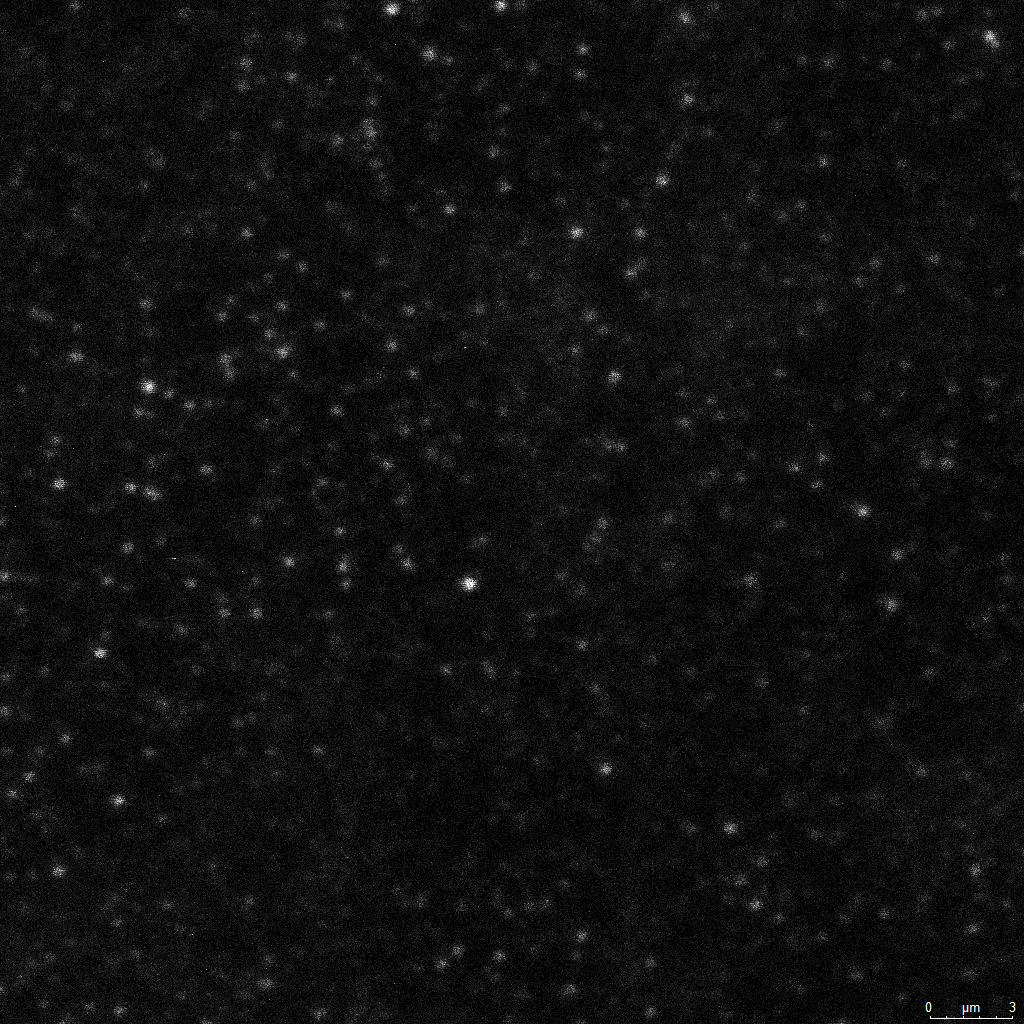

Supplement: Supplementary file 9 — Source data Fig. 4 [file 44319_2024_218_MOESM9_ESM.zip › Figure 4/4C/p10/dKO 898_dKO p10 898, CA1, IMPDH2 488W, NEUN 568G, ZS 63X-1D_ch01.tif]

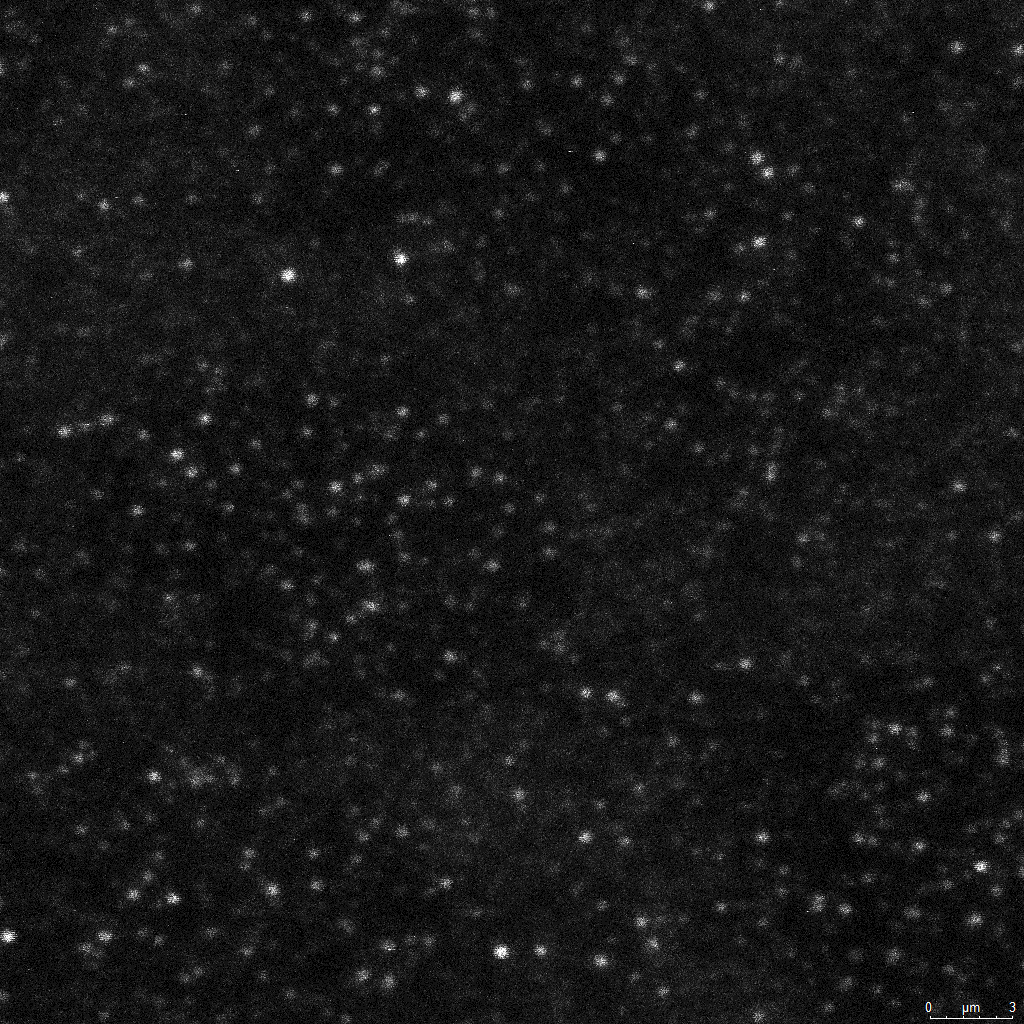

Supplement: Supplementary file 9 — Source data Fig. 4 [file 44319_2024_218_MOESM9_ESM.zip › Figure 4/4C/p10/dKO 898_dKO p10 898, CA3, IMPDH2 488W, NEUN 568G, ZS 63X-2D_ch01.tif]

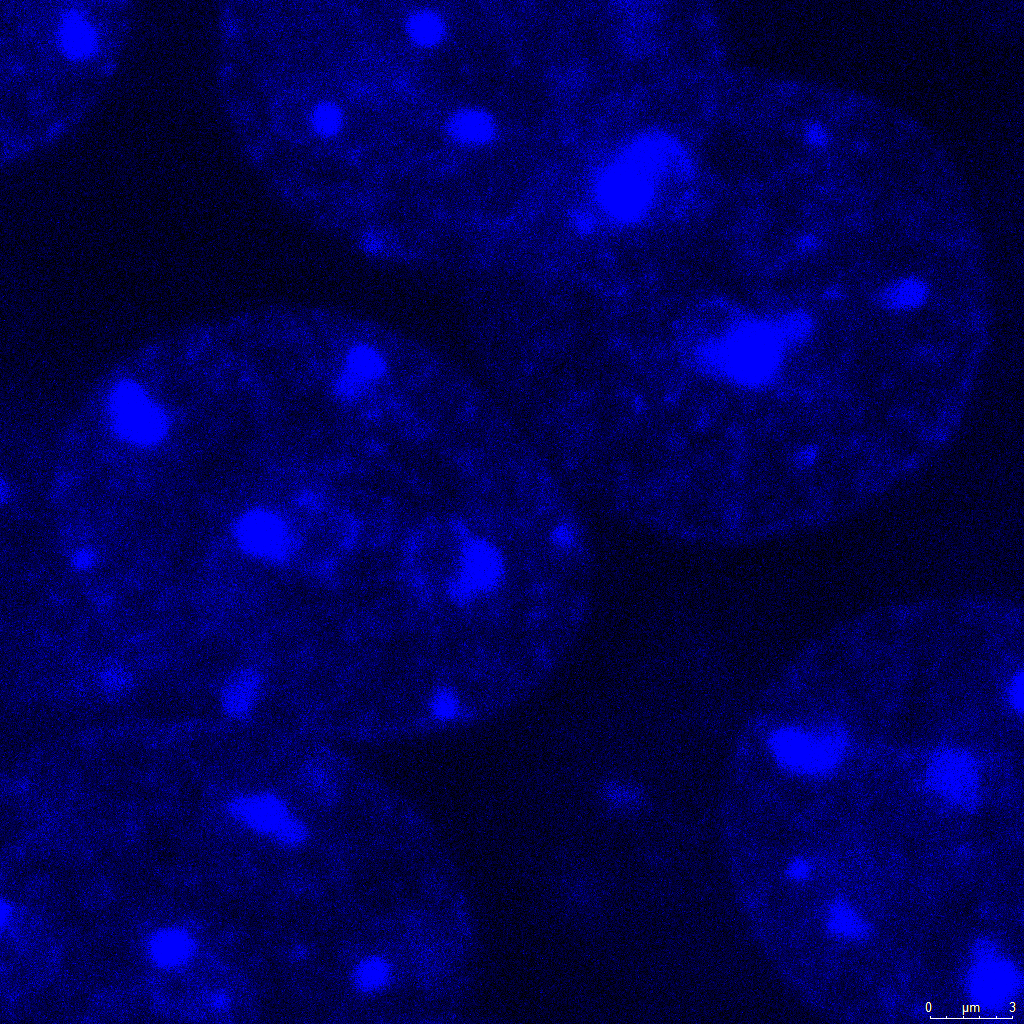

Supplement: Supplementary file 9 — Source data Fig. 4 [file 44319_2024_218_MOESM9_ESM.zip › Figure 4/4C/p10/dKO 898_dKO p10 898, CA3, IMPDH2 488W, NEUN 568G, ZS 63X-2D_ch00.tif]

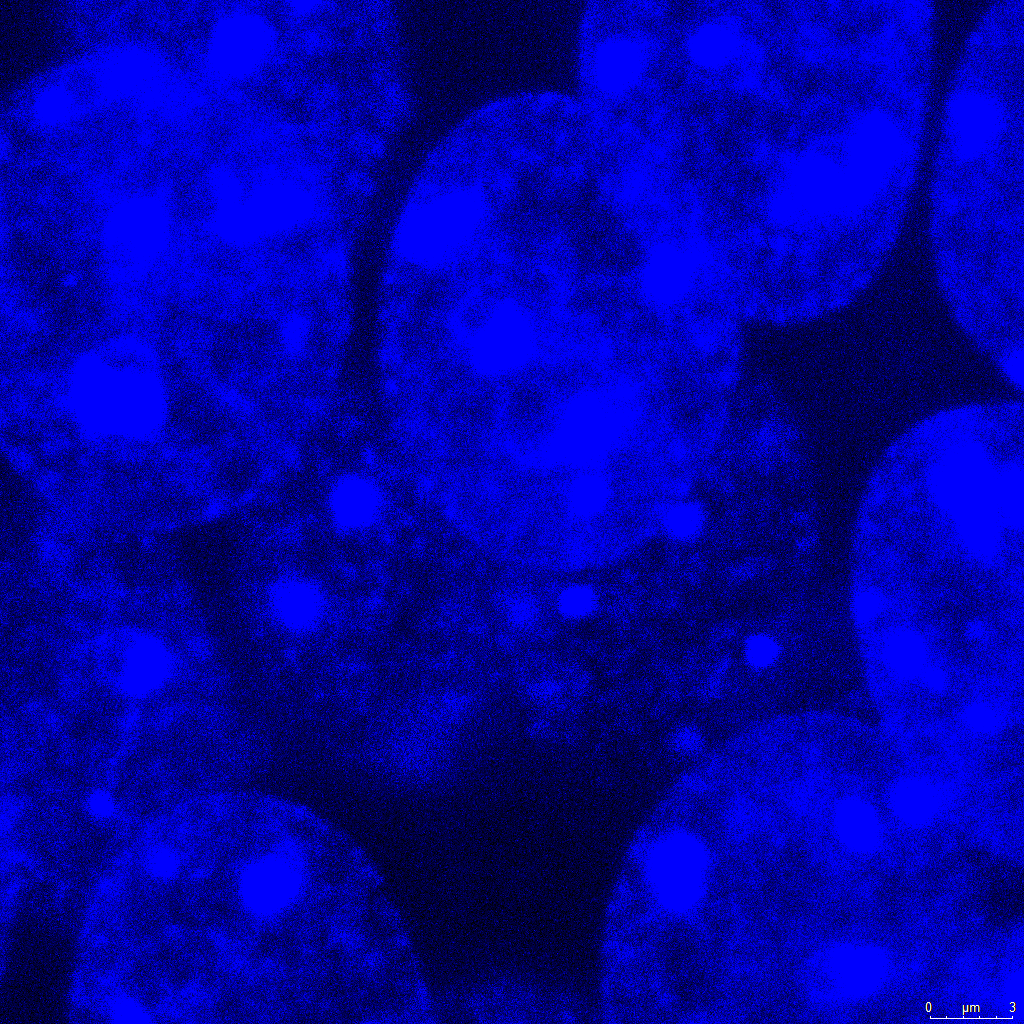

Supplement: Supplementary file 9 — Source data Fig. 4 [file 44319_2024_218_MOESM9_ESM.zip › Figure 4/4C/p20/p20 dKO 900_dKO p20 900, CA1, IMPDH2 488W, NEUN 568G, ZS 63X-1E_ch00.tif]

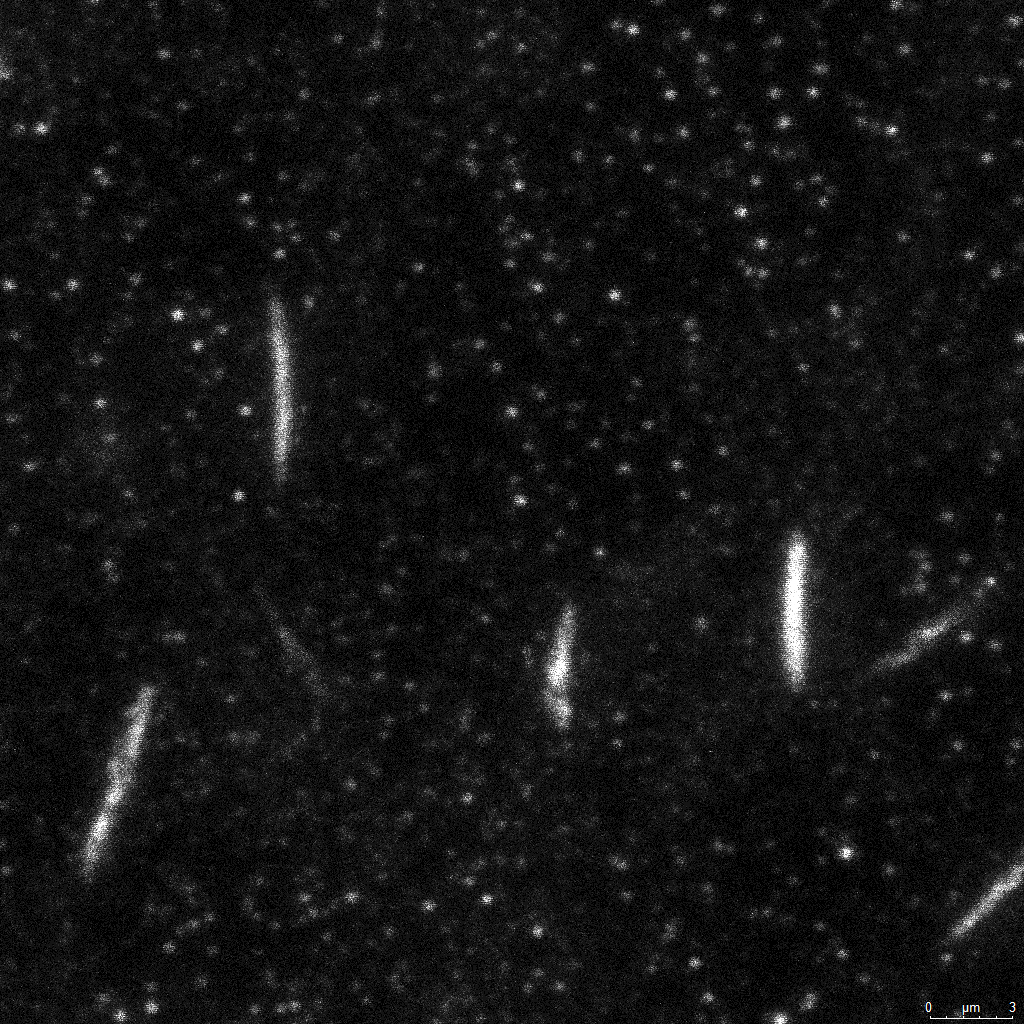

Supplement: Supplementary file 9 — Source data Fig. 4 [file 44319_2024_218_MOESM9_ESM.zip › Figure 4/4C/p20/p20 dKO 900_dKO p20 900, CA1, IMPDH2 488W, NEUN 568G, ZS 63X-1E_ch01.tif]

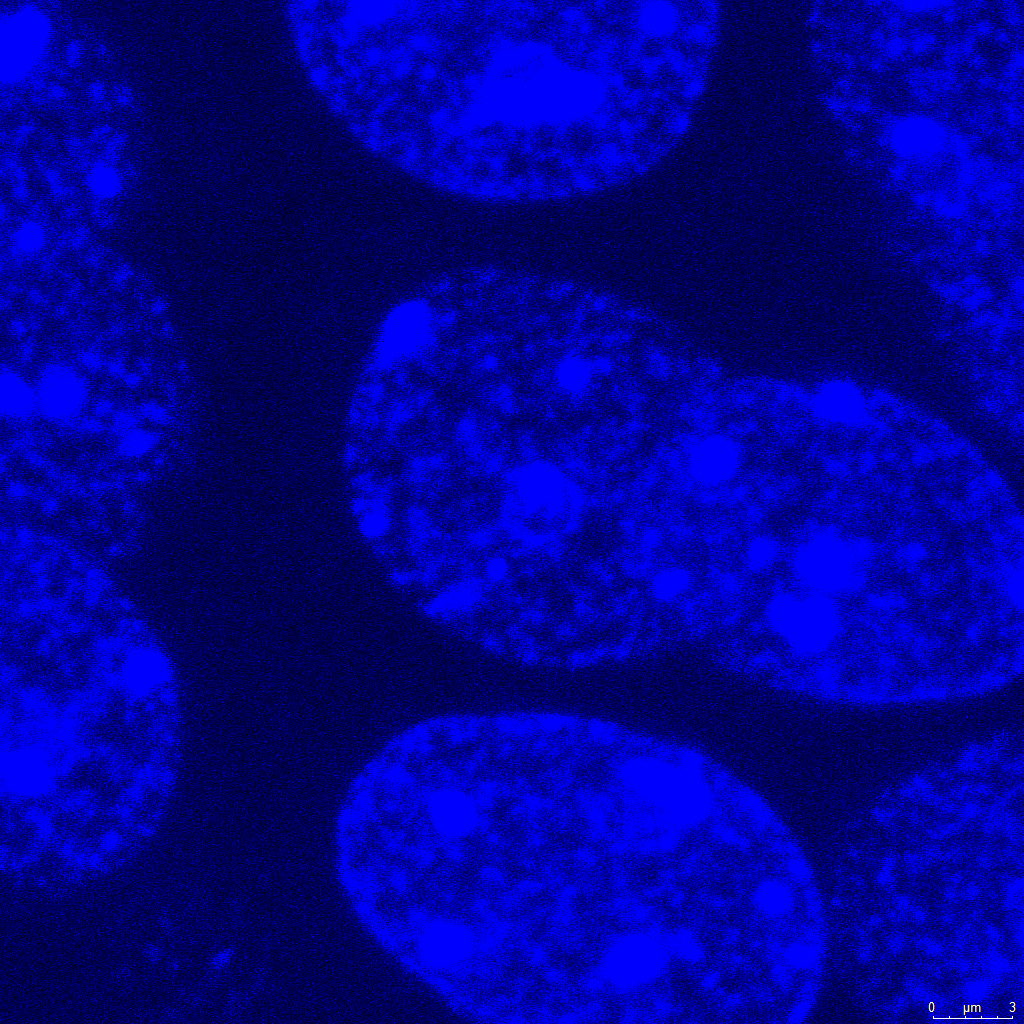

Supplement: Supplementary file 9 — Source data Fig. 4 [file 44319_2024_218_MOESM9_ESM.zip › Figure 4/4C/p20/p20 dKO 900_dKO p20 900, CA3, IMPDH2 488W, NEUN 568G, ZS 63X-1C_ch00.tif]

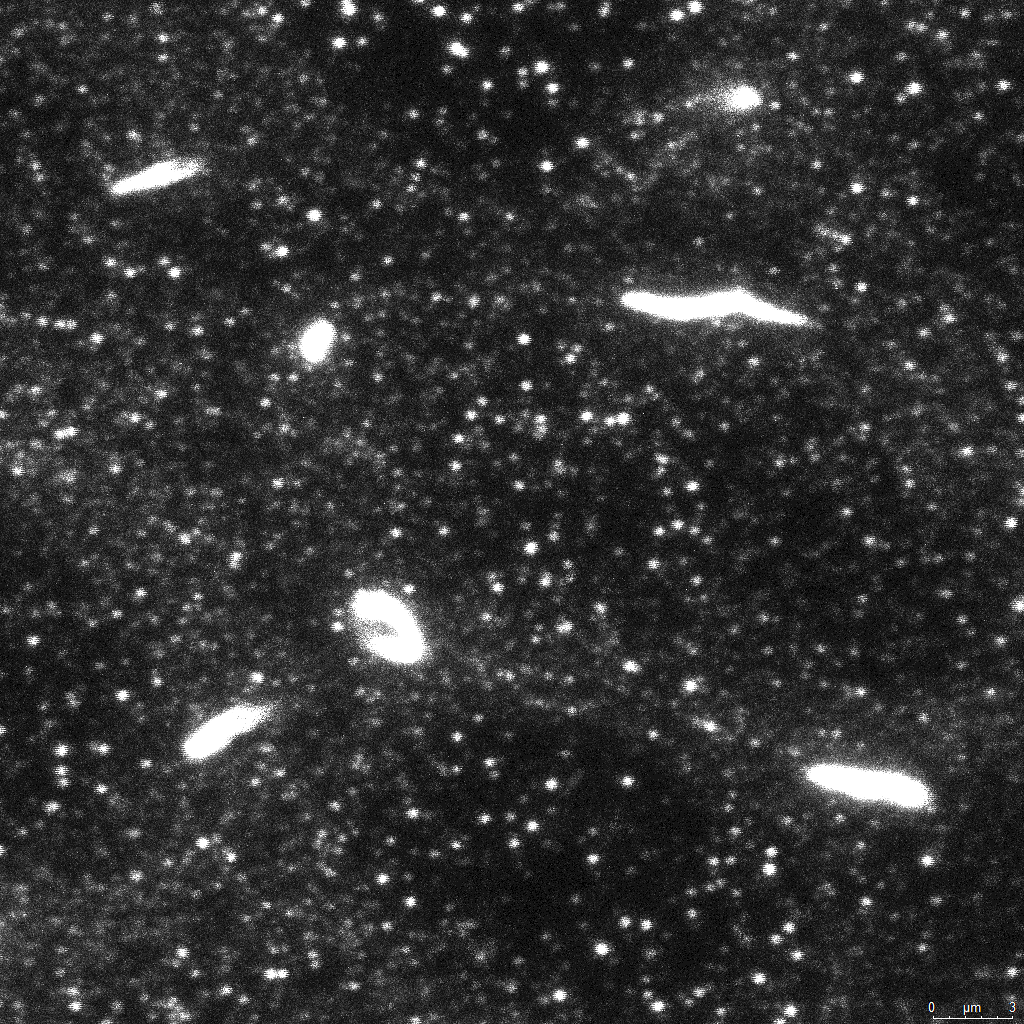

Supplement: Supplementary file 9 — Source data Fig. 4 [file 44319_2024_218_MOESM9_ESM.zip › Figure 4/4C/p20/p20 dKO 900_dKO p20 900, CA3, IMPDH2 488W, NEUN 568G, ZS 63X-1C_ch01.tif]

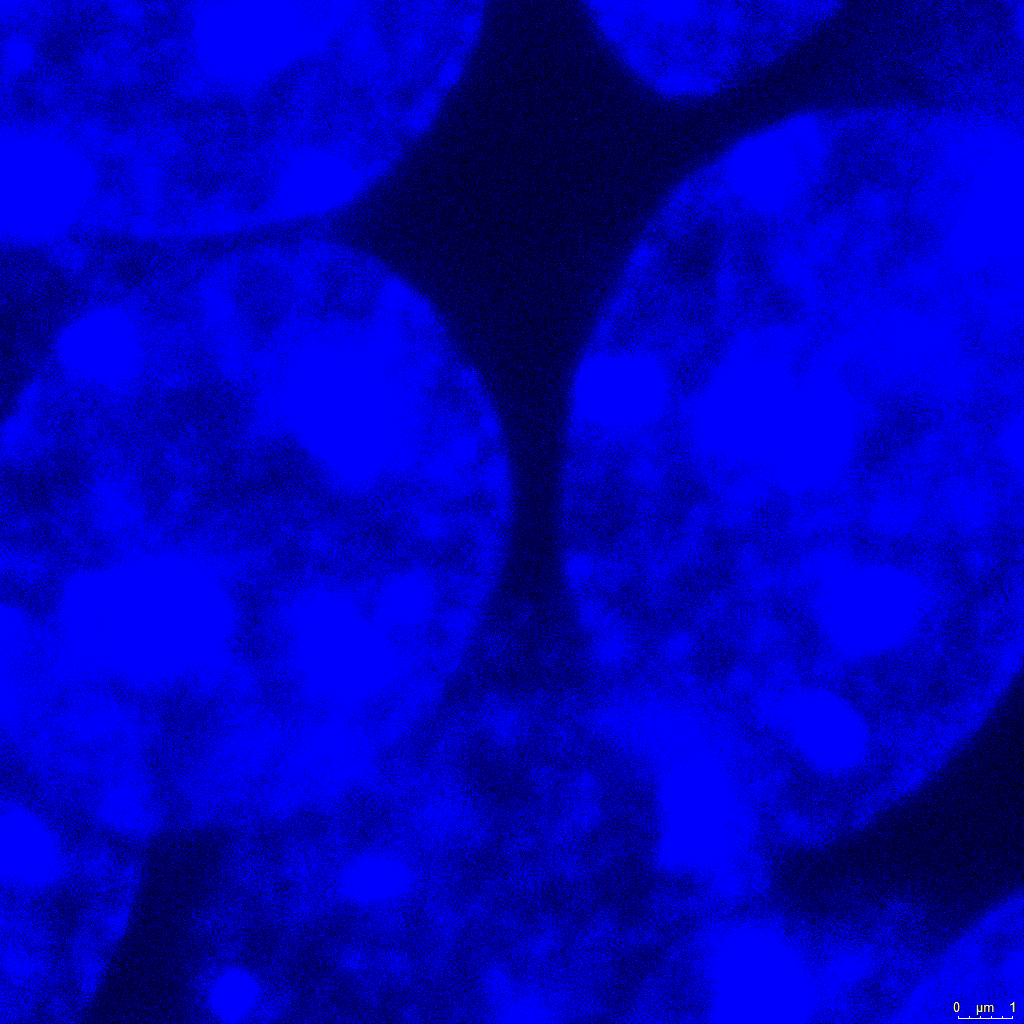

Supplement: Supplementary file 9 — Source data Fig. 4 [file 44319_2024_218_MOESM9_ESM.zip › Figure 4/4C/p20/p20 dKO 900_dKO p20 900, DG, IMPDH2 488W, NEUN 568G, ZS 63X-1E_ch00.tif]

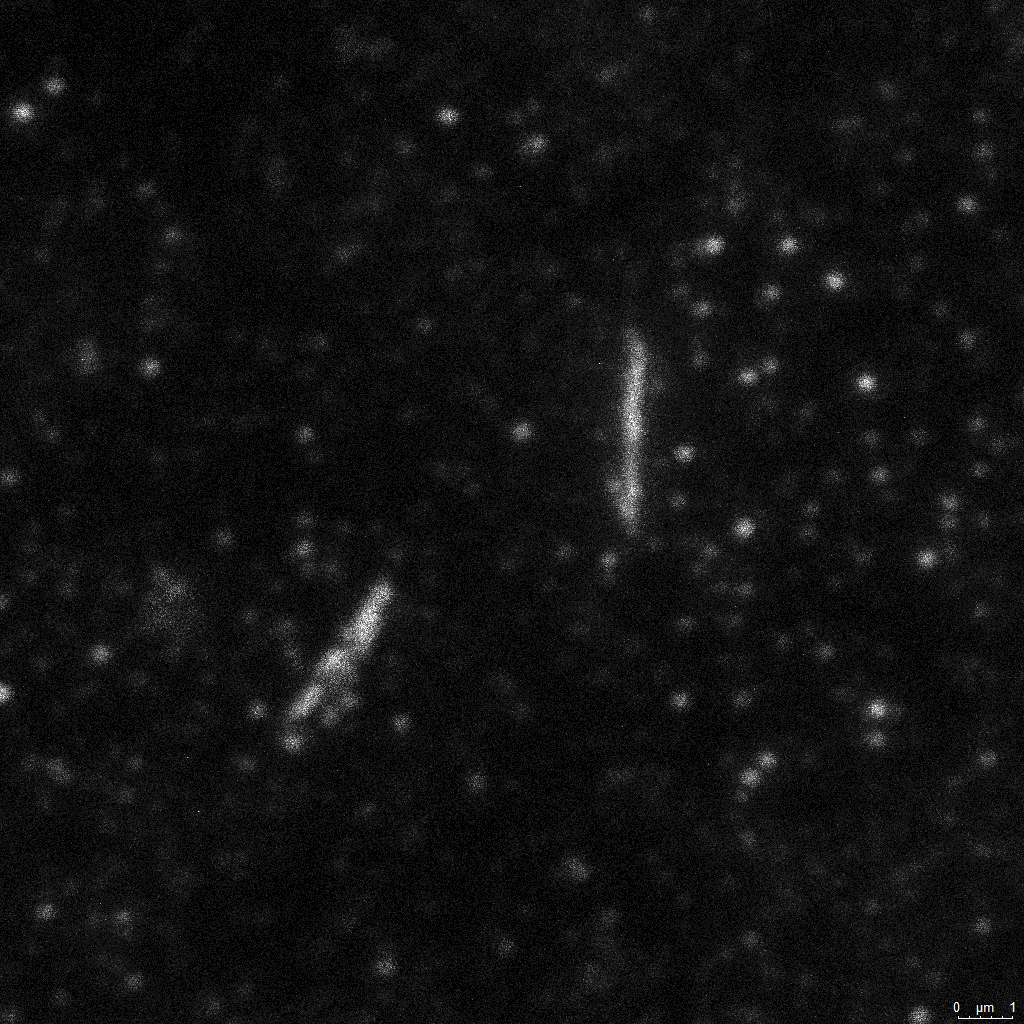

Supplement: Supplementary file 9 — Source data Fig. 4 [file 44319_2024_218_MOESM9_ESM.zip › Figure 4/4C/p20/p20 dKO 900_dKO p20 900, DG, IMPDH2 488W, NEUN 568G, ZS 63X-1E_ch01.tif]

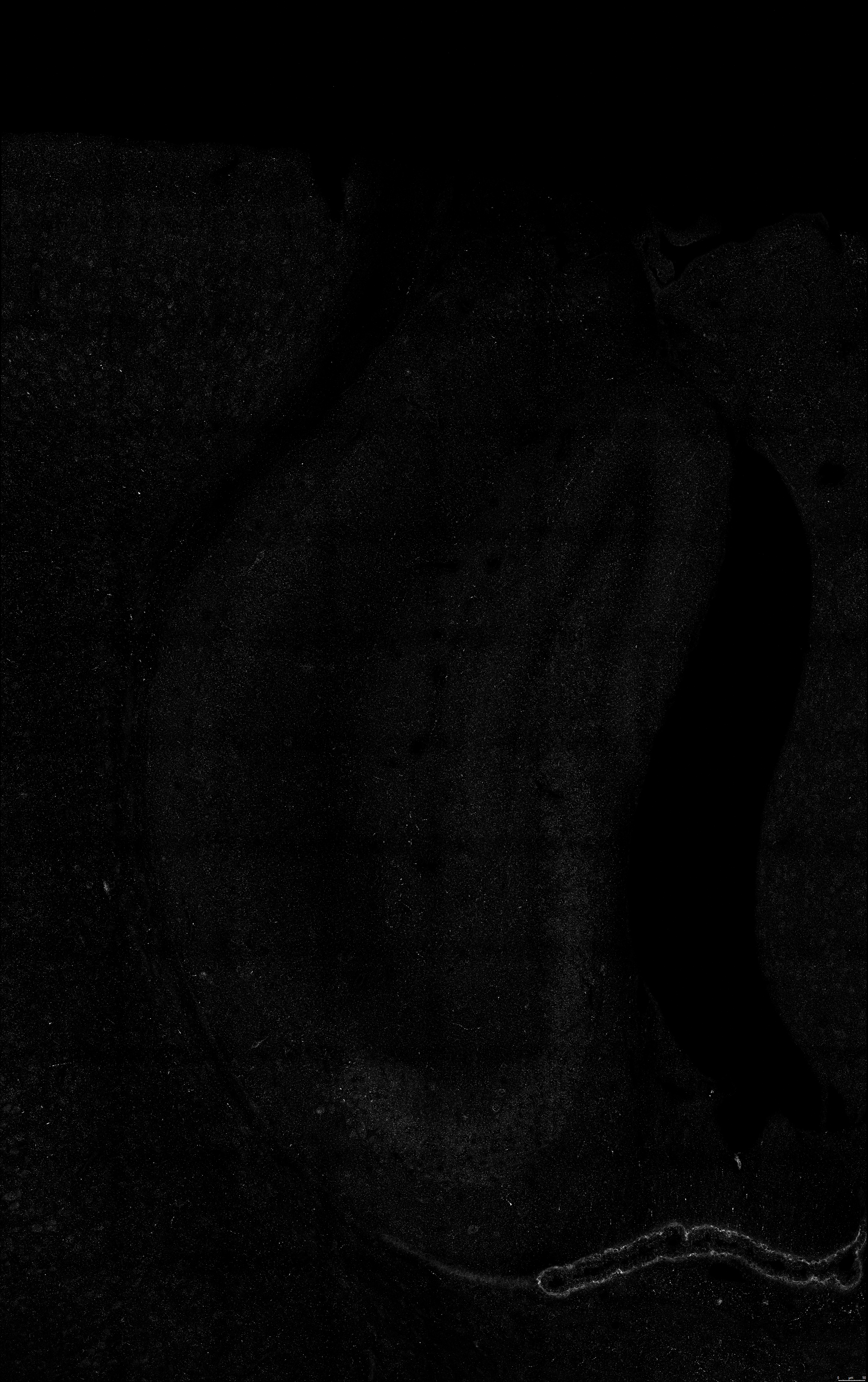

Supplement: Supplementary file 9 — Source data Fig. 4 [file 44319_2024_218_MOESM9_ESM.zip › Figure 4/4C/p15/TileScan 001_TileScan_001_dKO p15 896, IMPDH2 488W, NEUN 568G, ZS 63X-1A_ch01.tif]

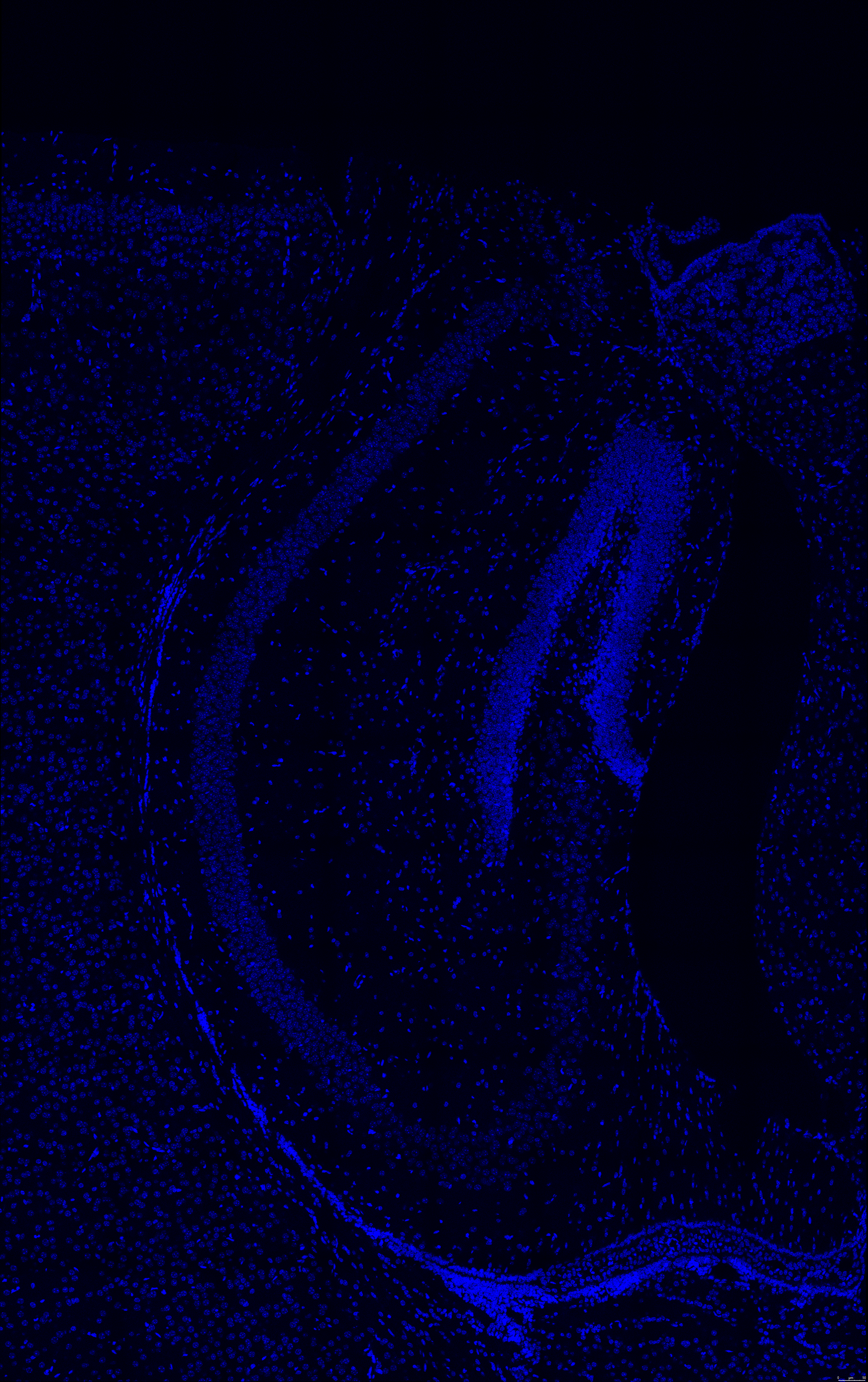

Supplement: Supplementary file 9 — Source data Fig. 4 [file 44319_2024_218_MOESM9_ESM.zip › Figure 4/4C/p15/TileScan 001_TileScan_001_dKO p15 896, IMPDH2 488W, NEUN 568G, ZS 63X-1A_ch00.tif]

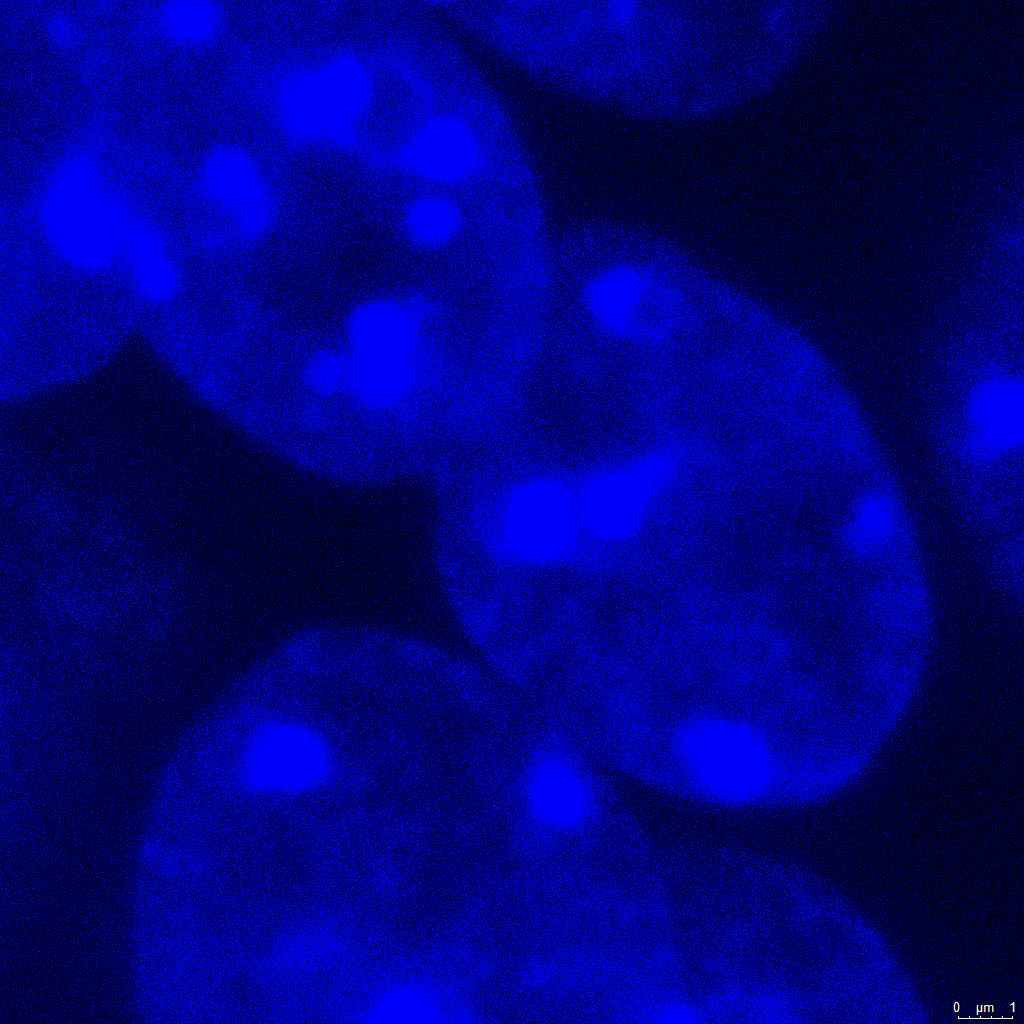

Supplement: Supplementary file 9 — Source data Fig. 4 [file 44319_2024_218_MOESM9_ESM.zip › Figure 4/4C/p15/dKO 896_dKO p15 896, DG, IMPDH2 488W, NEUN 568G, ZS 63X-1F_ch00.tif]

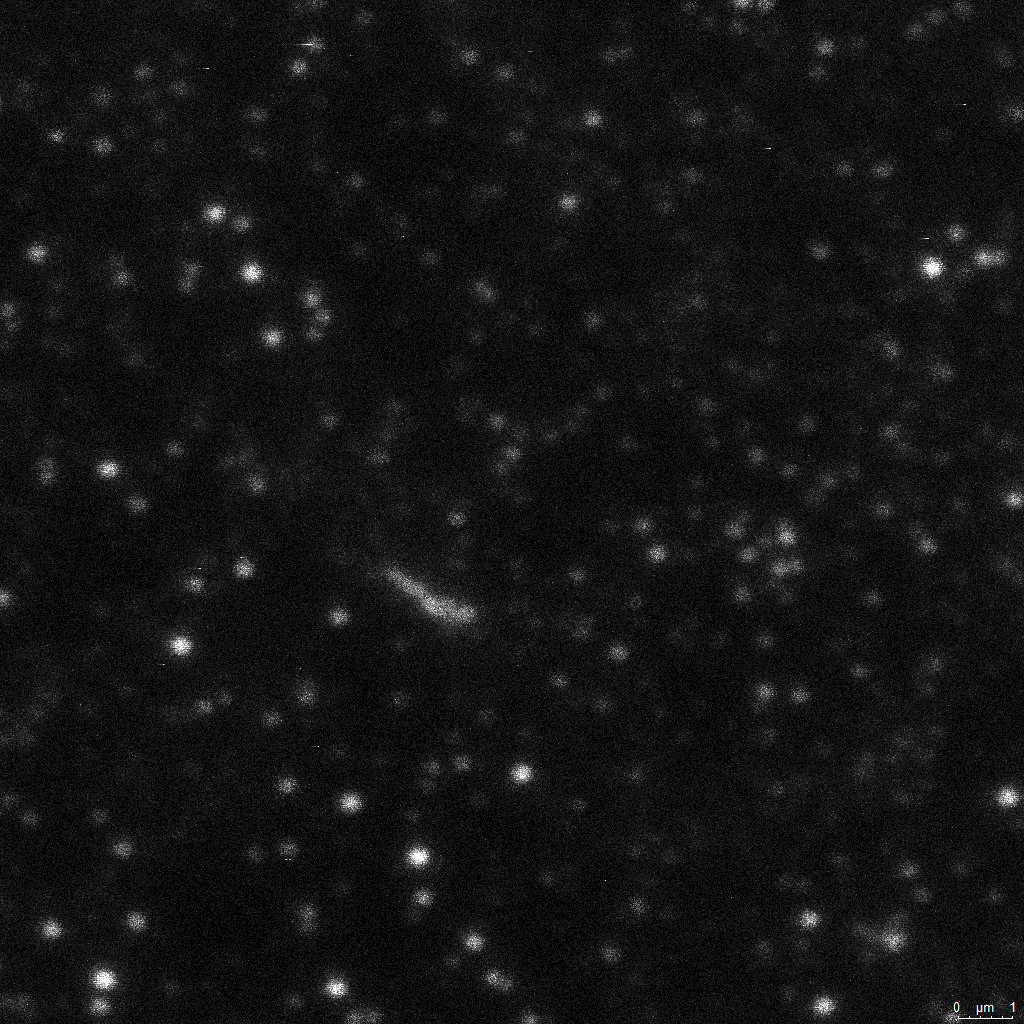

Supplement: Supplementary file 9 — Source data Fig. 4 [file 44319_2024_218_MOESM9_ESM.zip › Figure 4/4C/p15/dKO 896_dKO p15 896, DG, IMPDH2 488W, NEUN 568G, ZS 63X-1F_ch01.tif]

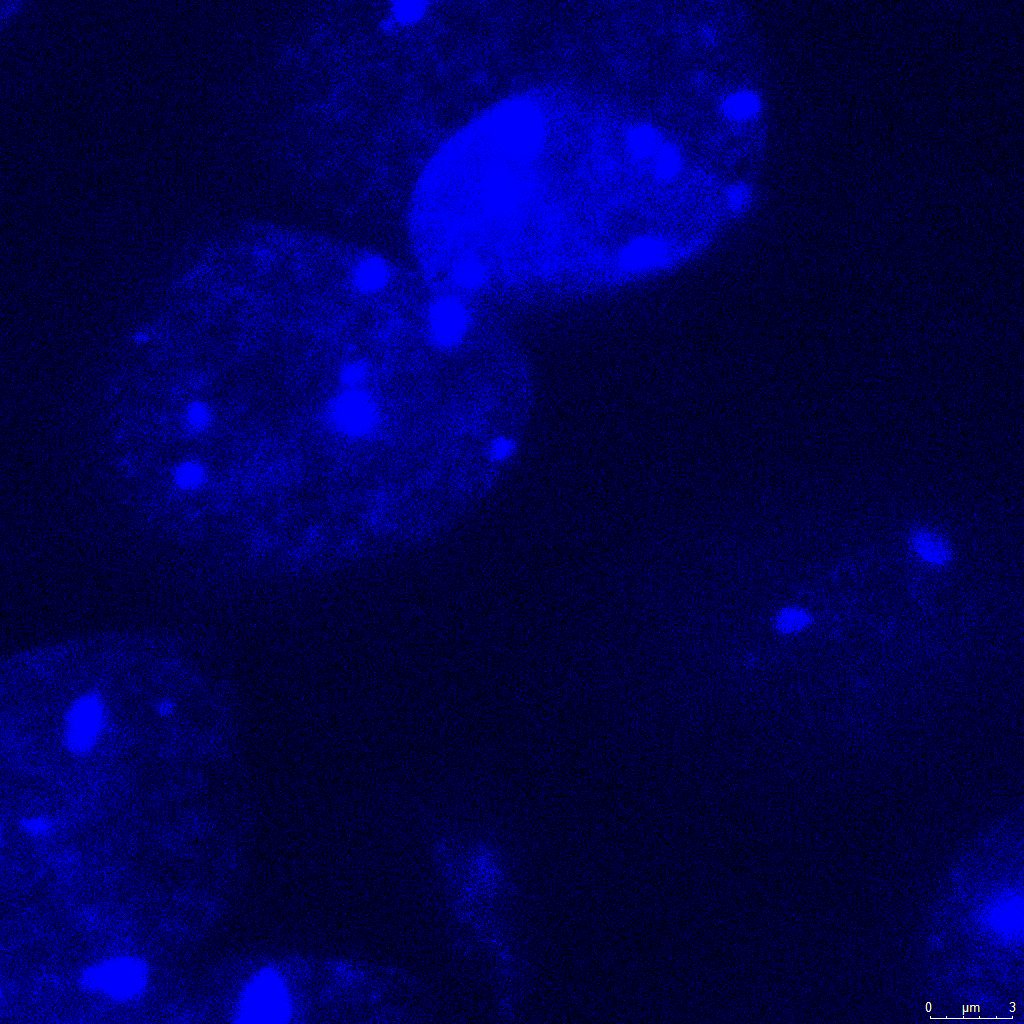

Supplement: Supplementary file 9 — Source data Fig. 4 [file 44319_2024_218_MOESM9_ESM.zip › Figure 4/4C/p15/dKO 896_dKO p15 896, CA3, IMPDH2 488W, NEUN 568G, ZS 63X-2D_ch00.tif]

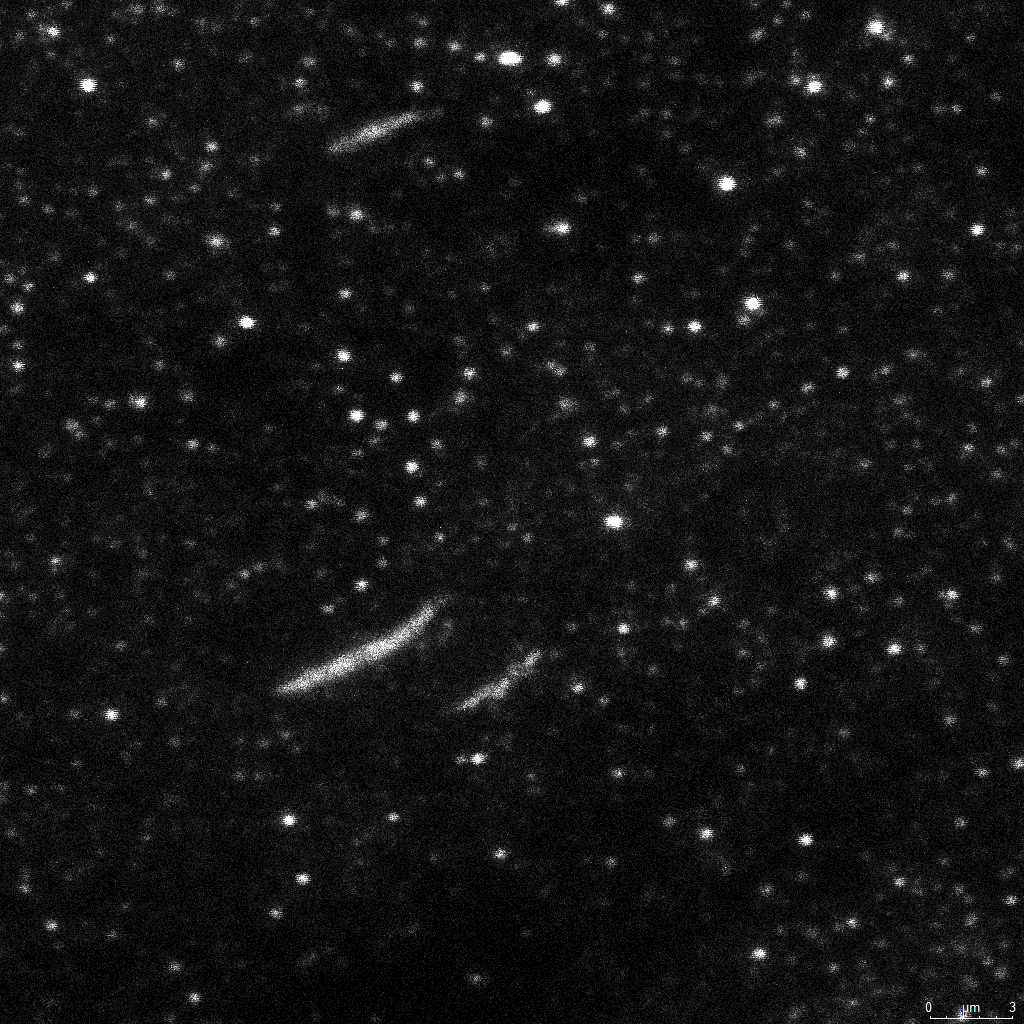

Supplement: Supplementary file 9 — Source data Fig. 4 [file 44319_2024_218_MOESM9_ESM.zip › Figure 4/4C/p15/dKO 896_dKO p15 896, CA3, IMPDH2 488W, NEUN 568G, ZS 63X-2D_ch01.tif]

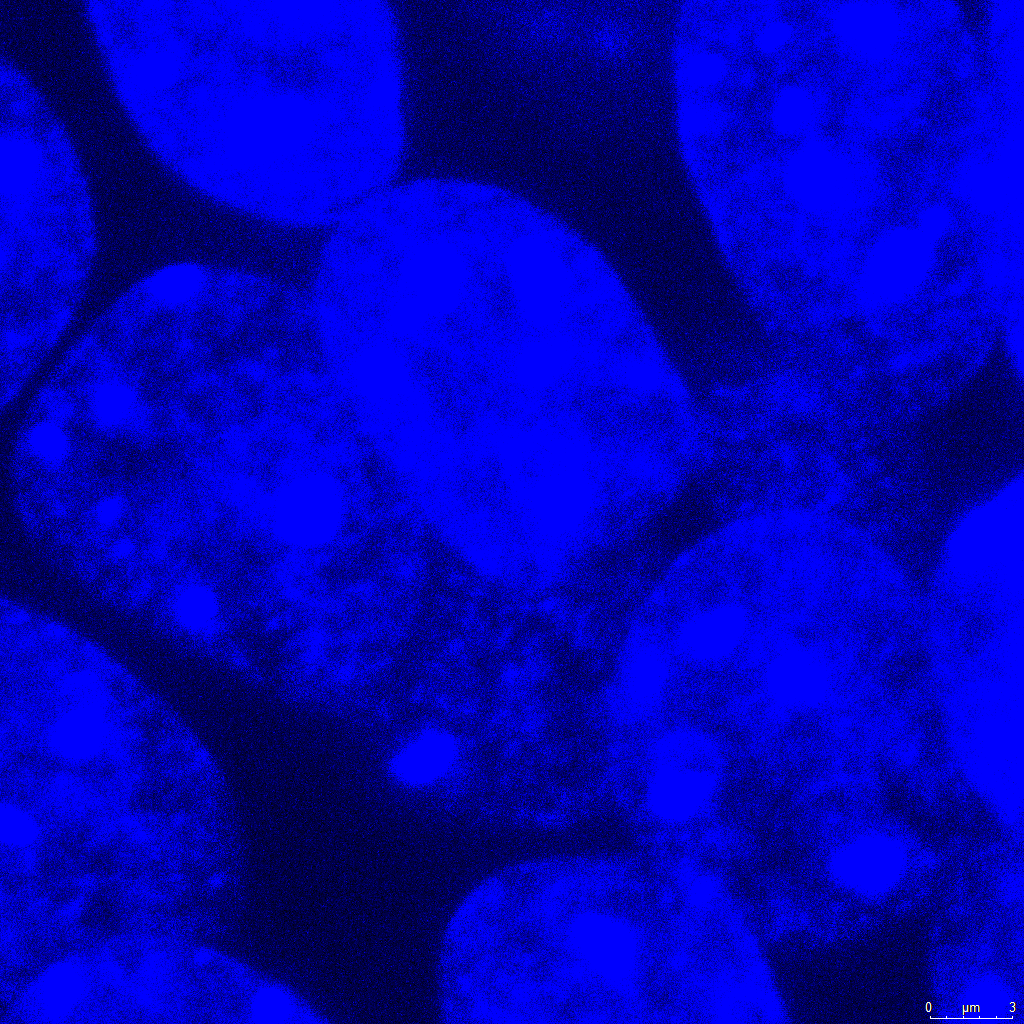

Supplement: Supplementary file 9 — Source data Fig. 4 [file 44319_2024_218_MOESM9_ESM.zip › Figure 4/4C/p15/dKO 896_dKO p15 896, CA1, IMPDH2 488W, NEUN 568G, ZS 63X-2D_ch00.tif]

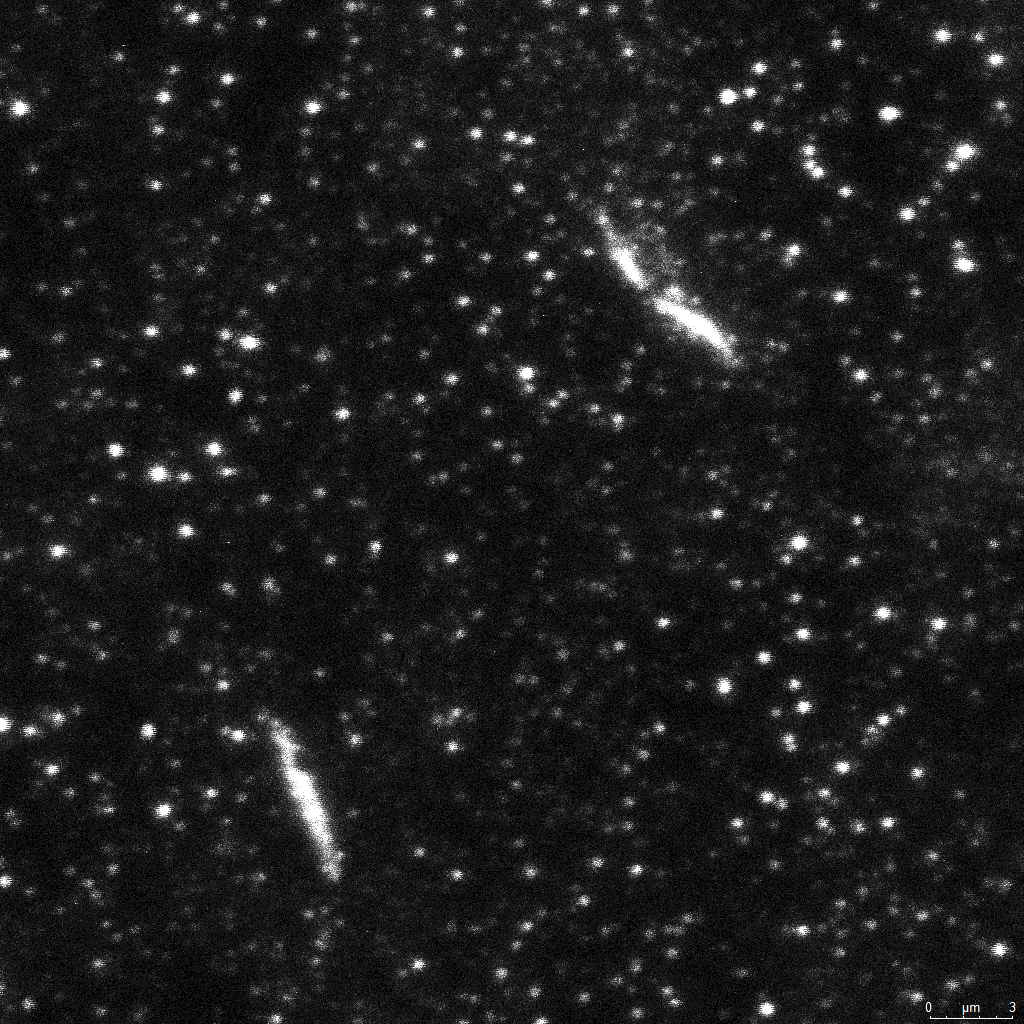

Supplement: Supplementary file 9 — Source data Fig. 4 [file 44319_2024_218_MOESM9_ESM.zip › Figure 4/4C/p15/dKO 896_dKO p15 896, CA1, IMPDH2 488W, NEUN 568G, ZS 63X-2D_ch01.tif]

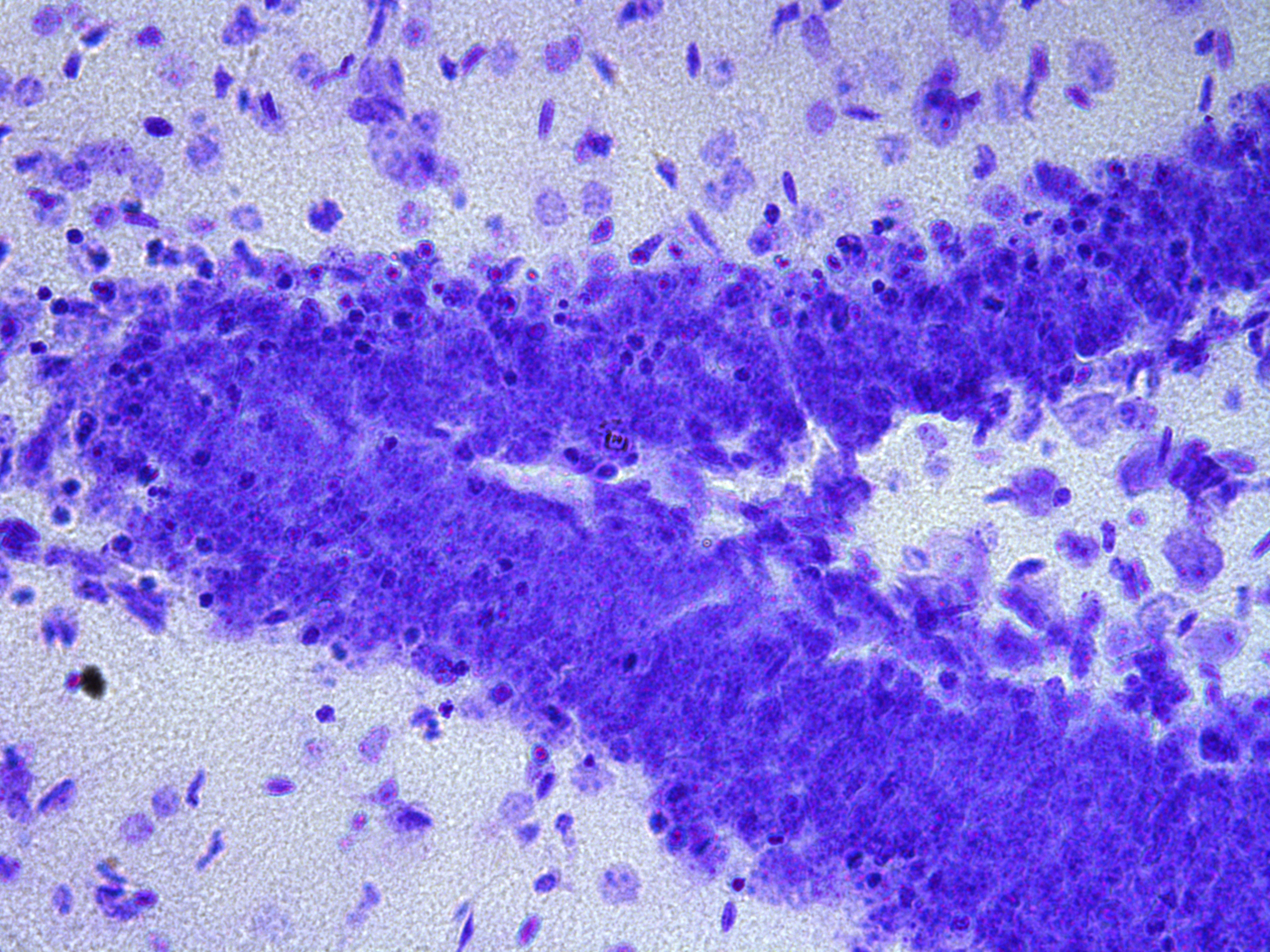

Supplement: Supplementary file 10 — Source data Fig. 5A-B [file 44319_2024_218_MOESM10_ESM.zip › Figure 5 A-B/5A/cdKO 5w/cdKO DG, NISSL_Ant_Hip_5w_CKO_BF_20X_Region_40X_Pygnotic_Cells_Rep_2.12.21.lif - 914_KFC_Ant_Hip_DG_crest_40X (RGB).tif]

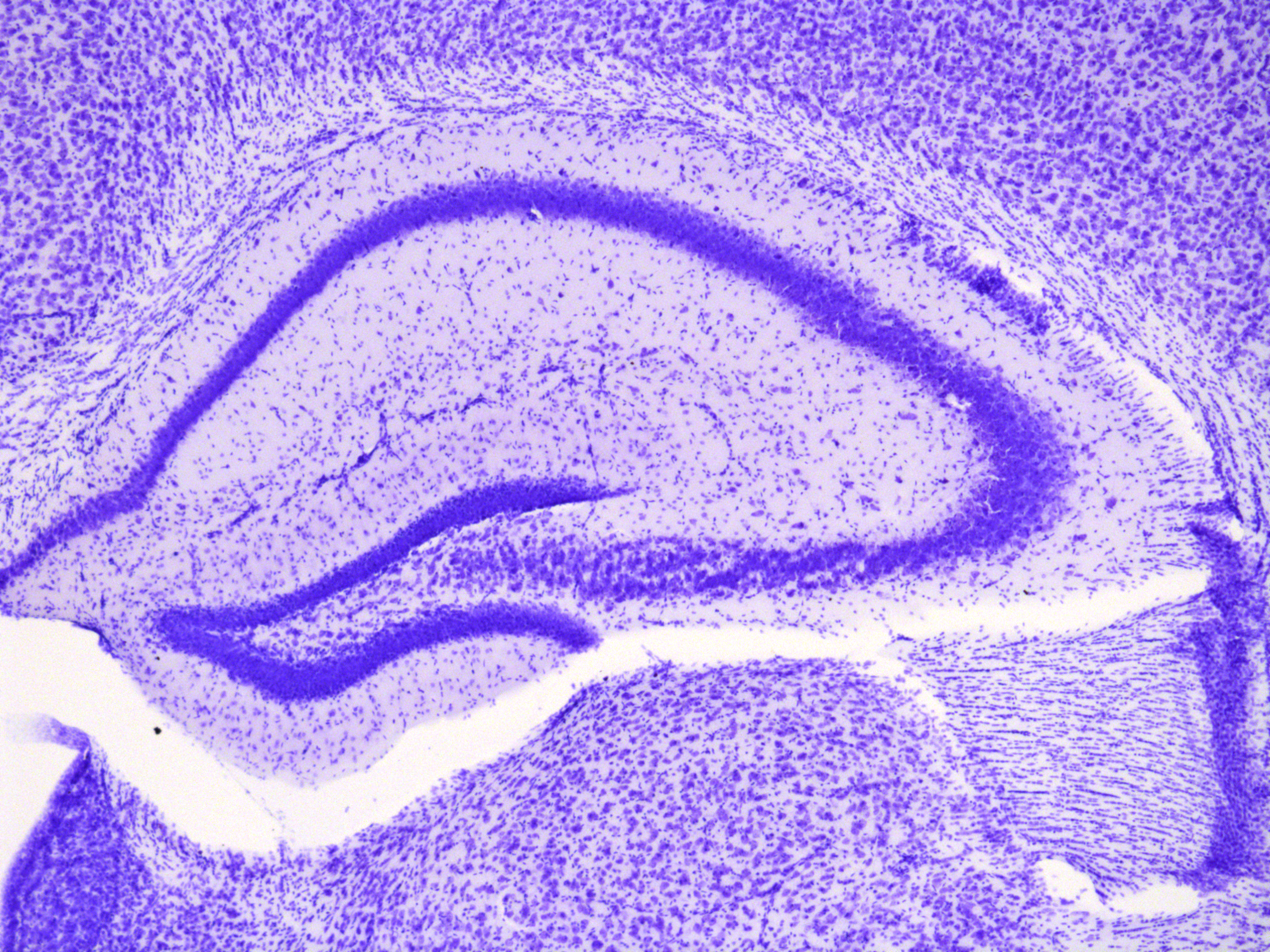

Supplement: Supplementary file 10 — Source data Fig. 5A-B [file 44319_2024_218_MOESM10_ESM.zip › Figure 5 A-B/5A/cdKO 5w/cdKO Hip, NISSL_5w_D2D3CKO_BF_5X_Overview_2.12.21.lif - 914_KFC_Ant_Hip_5x (RGB)-1.tif]

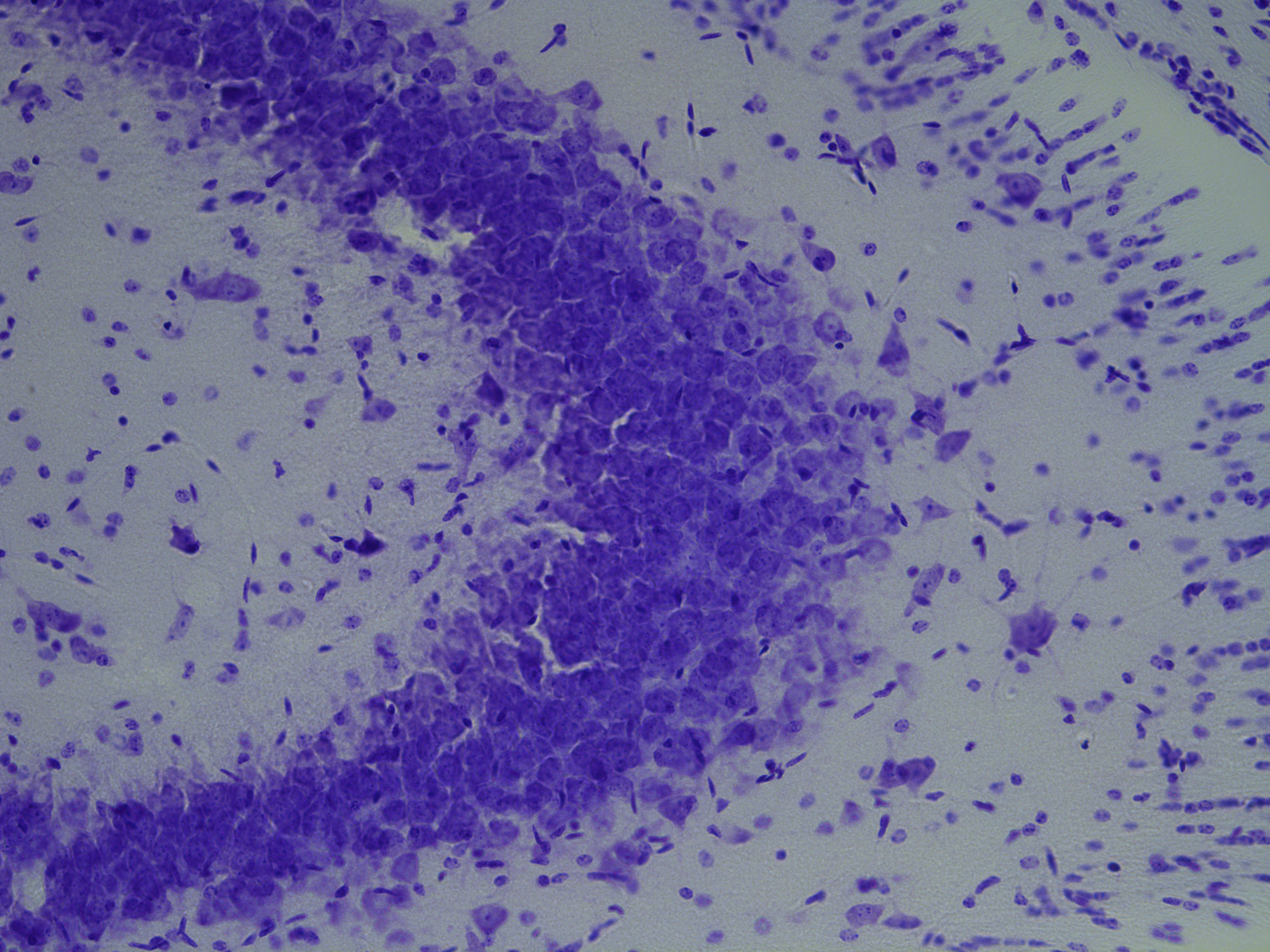

Supplement: Supplementary file 10 — Source data Fig. 5A-B [file 44319_2024_218_MOESM10_ESM.zip › Figure 5 A-B/5A/cdKO 5w/cdKO CA3,NISSL_Ant_Hip_5w_CKO_BF_20X_Region_40X_Pygnotic_Cells_Rep_2.12.21.lif - 914_KFC_Ant_Hip_DG_CA3_20X (RGB).tif]

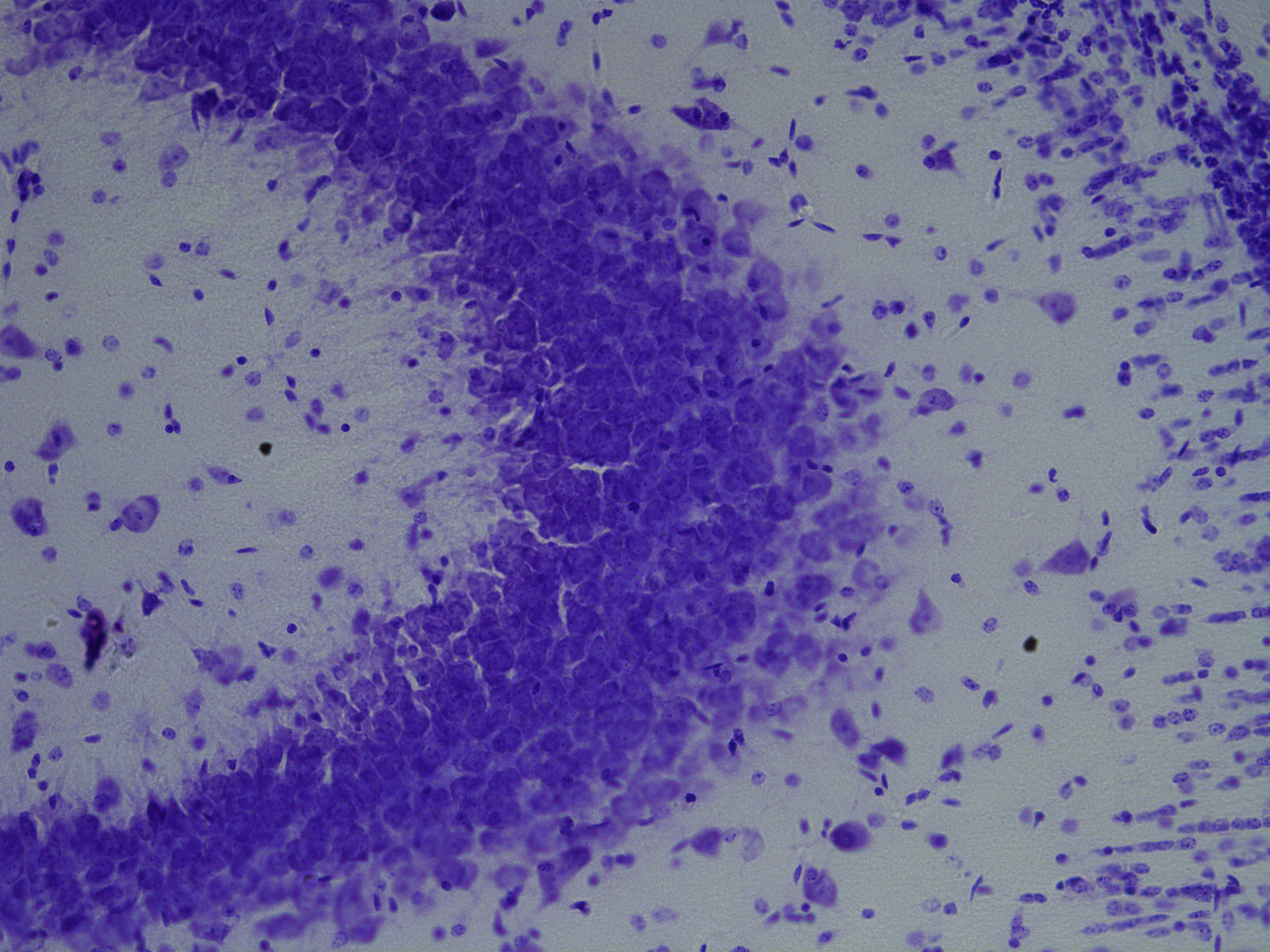

Supplement: Supplementary file 10 — Source data Fig. 5A-B [file 44319_2024_218_MOESM10_ESM.zip › Figure 5 A-B/5A/Ctrl 5w/Ctrl CA3, NISSL_Ant_Hip_5w_CKO_BF_20X_Region_40X_Pygnotic_Cells_Rep_2.12.21.lif - 913_KFC_Ant_Hip_DG_CA3_20X (RGB).tif]

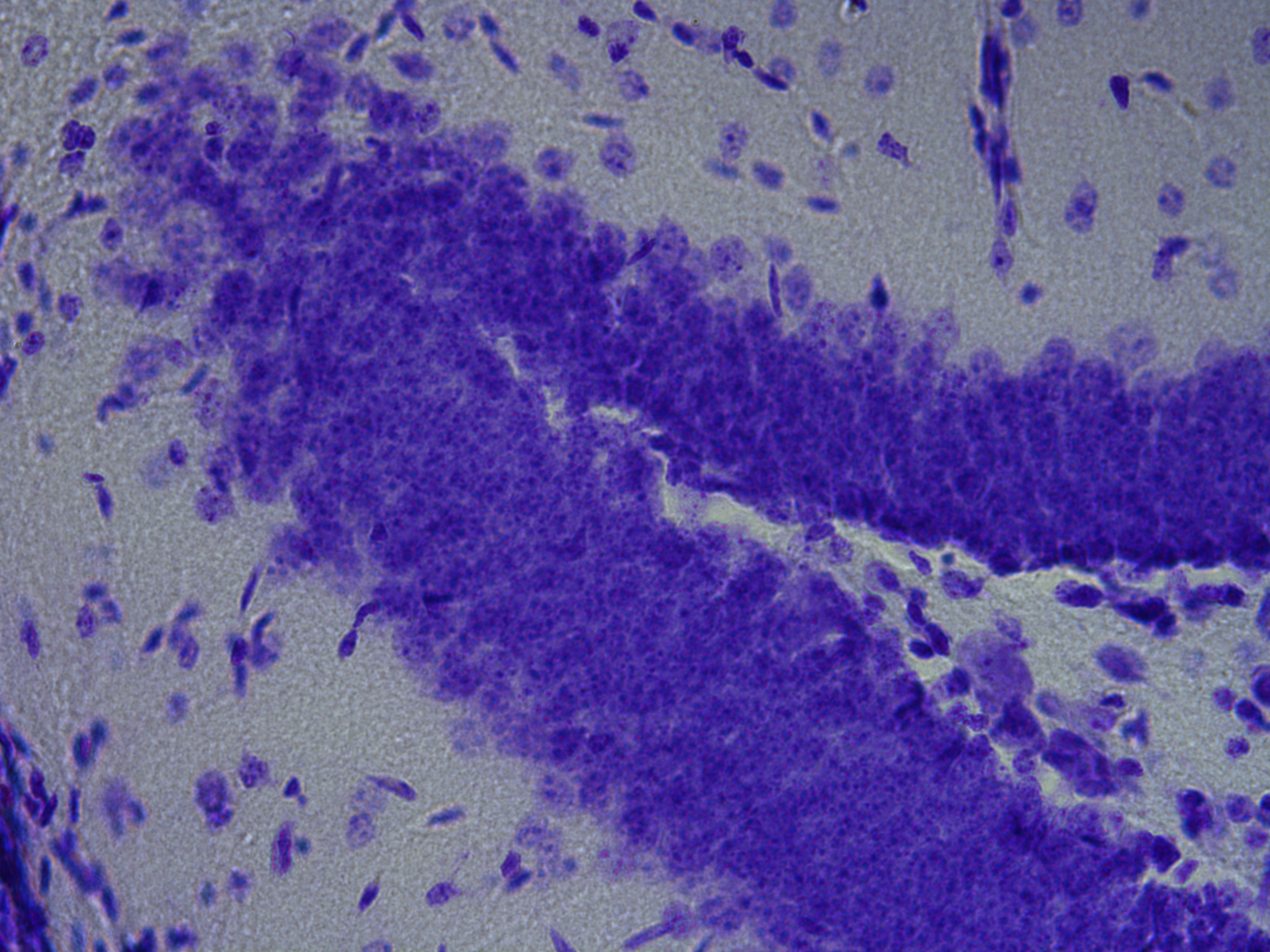

Supplement: Supplementary file 10 — Source data Fig. 5A-B [file 44319_2024_218_MOESM10_ESM.zip › Figure 5 A-B/5A/Ctrl 5w/Ctrl DG, NISSL_Ant_Hip_5w_CKO_BF_20X_Region_40X_Pygnotic_Cells_Rep_2.12.21.lif - 913_KF_Ant_Hip_DG_crest_40X (RGB).tif]

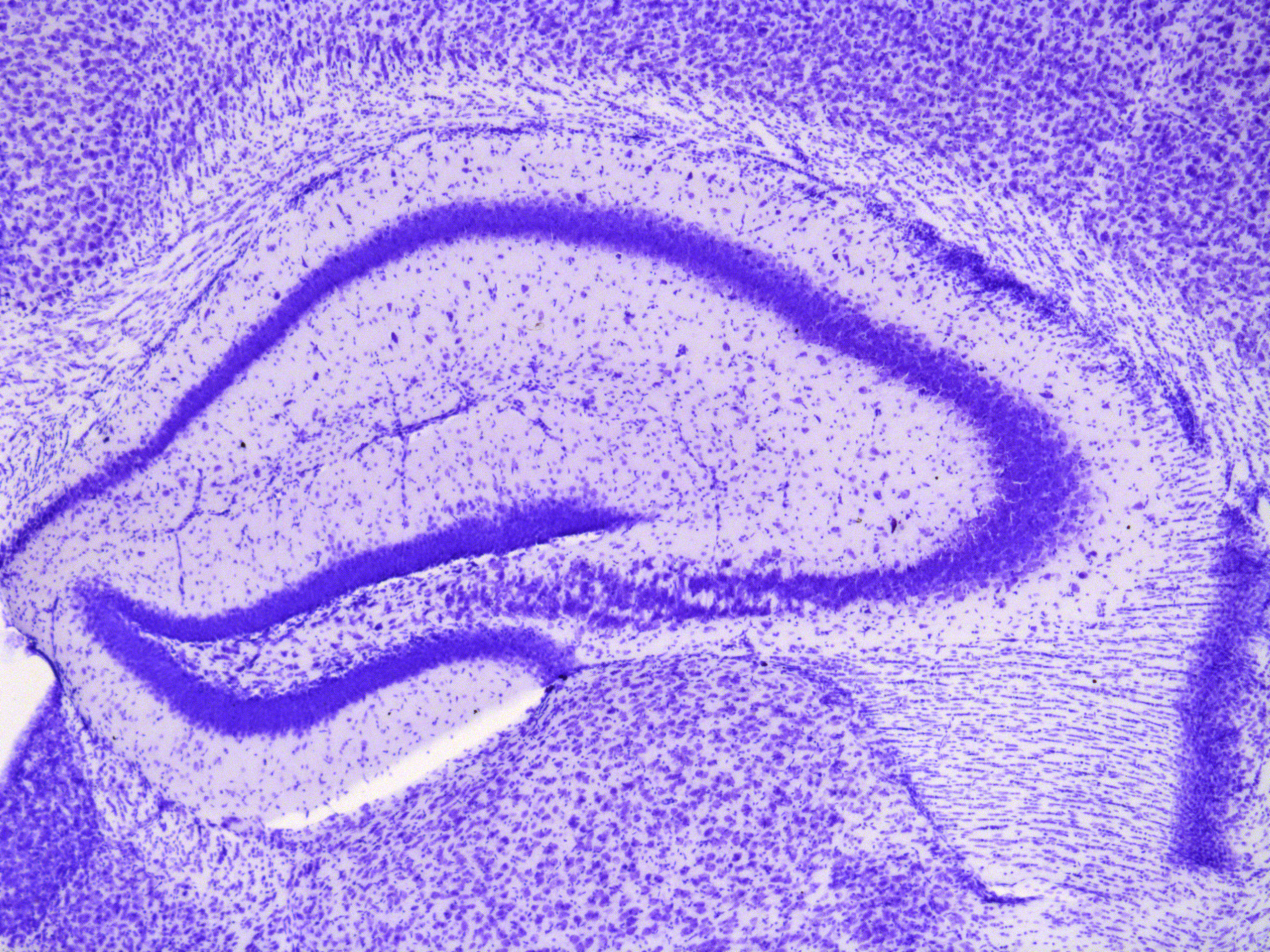

Supplement: Supplementary file 10 — Source data Fig. 5A-B [file 44319_2024_218_MOESM10_ESM.zip › Figure 5 A-B/5A/Ctrl 5w/Ctrl Hip, NISSL_5w_D2D3CKO_BF_5X_Overview_2.12.21.lif - 913_Cont_Ant_Hip_5x (RGB)-1.tif]

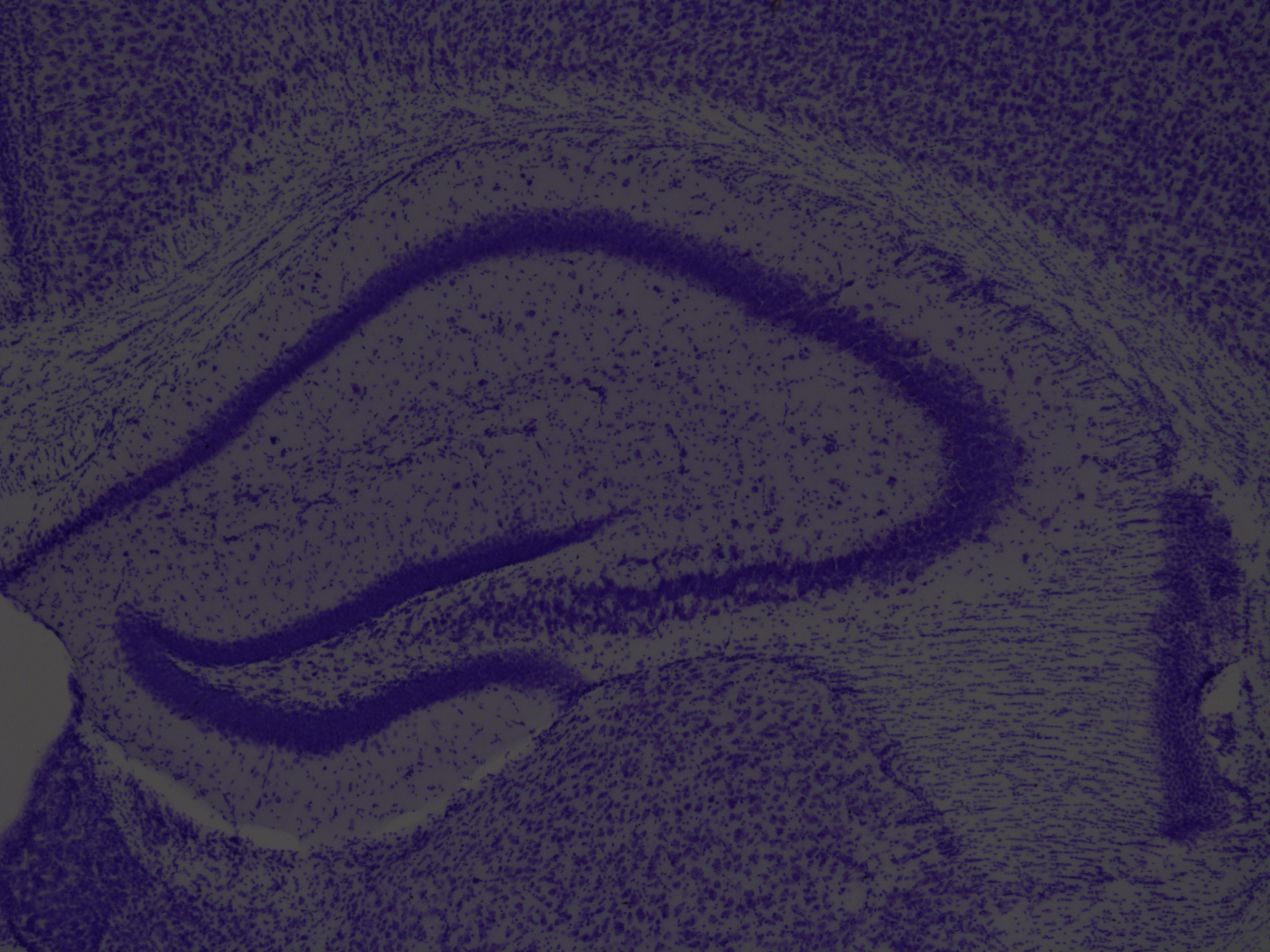

Supplement: Supplementary file 10 — Source data Fig. 5A-B [file 44319_2024_218_MOESM10_ESM.zip › Figure 5 A-B/5B/Ctrl/Ctrl Hip, NISSL_2mo_D2D3CKO_BF_5X_Overview_1.29.21.lif - 884_HFC_Ant_Hip_5x (RGB).tif]

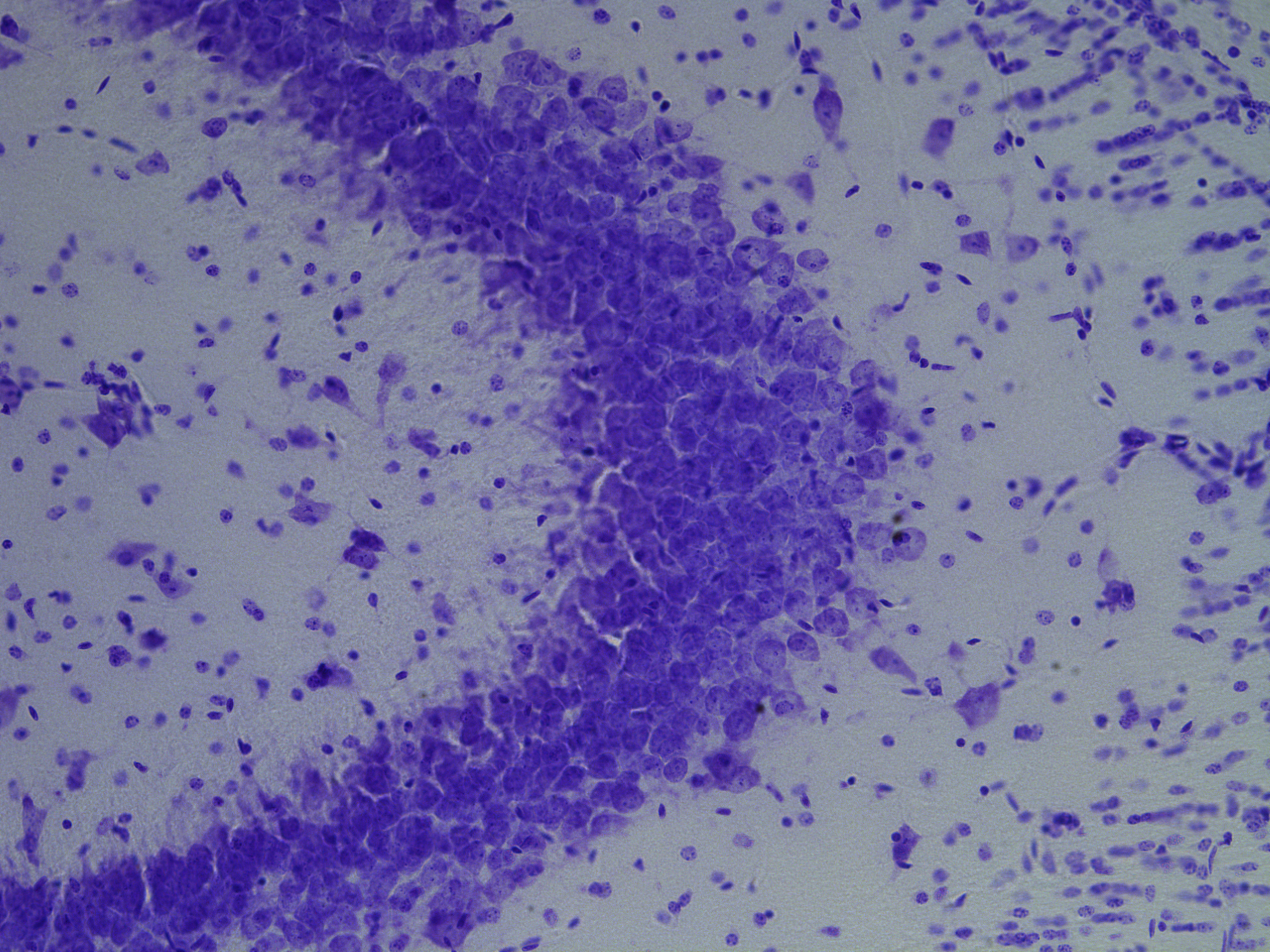

Supplement: Supplementary file 10 — Source data Fig. 5A-B [file 44319_2024_218_MOESM10_ESM.zip › Figure 5 A-B/5B/Ctrl/Ctrl CA3, NISSL_Ant_Hip_2mo_CKO_BF_20X_Region_40X_Pygnotic_Cells_Rep_1.29.21.lif - 884_HFC_Ant_Hip_DG_CA3_20X (RGB).tif]

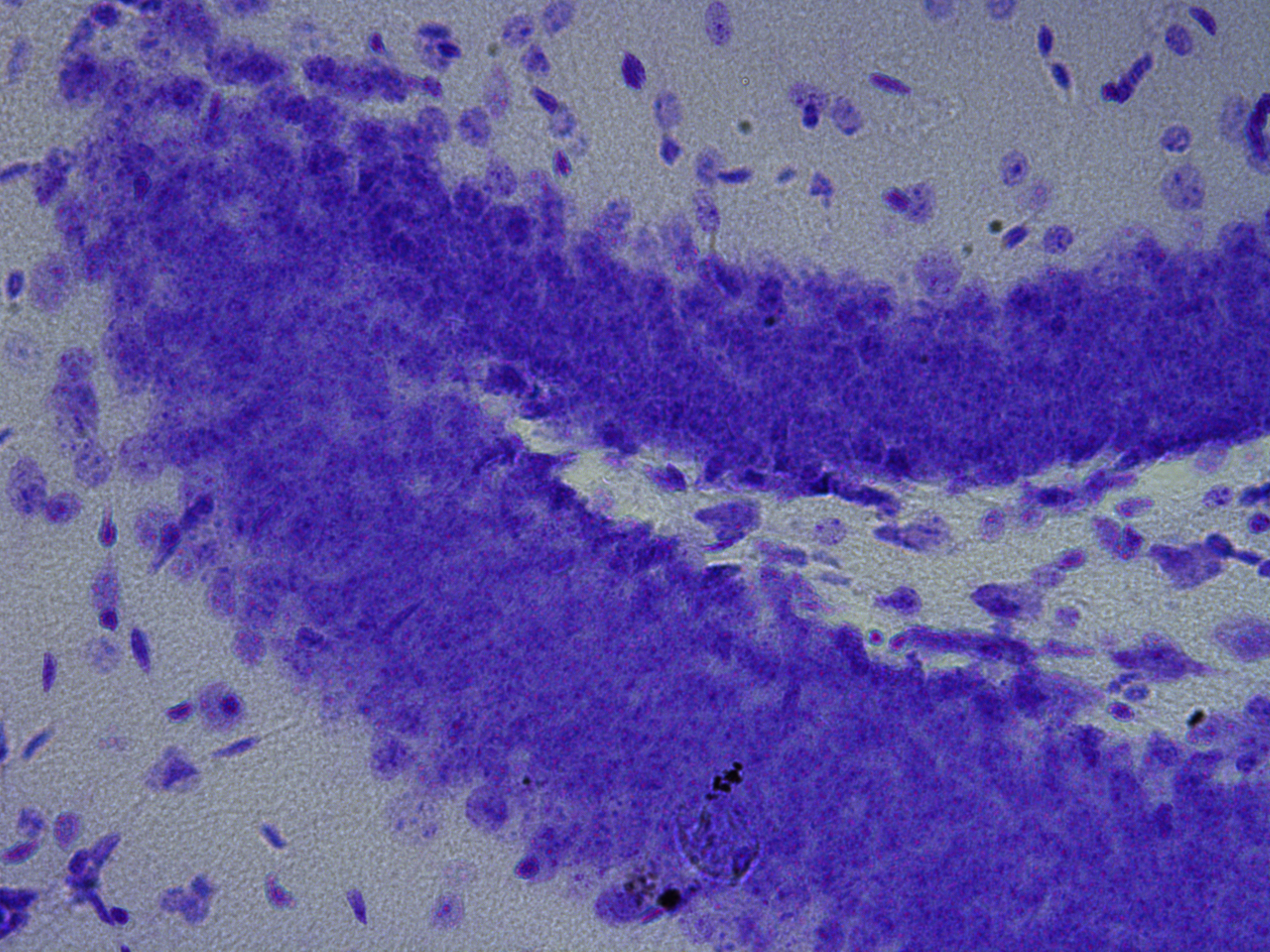

Supplement: Supplementary file 10 — Source data Fig. 5A-B [file 44319_2024_218_MOESM10_ESM.zip › Figure 5 A-B/5B/Ctrl/Ctrl DG, NISSL_Ant_Hip_2mo_CKO_BF_20X_Region_40X_Pygnotic_Cells_Rep_1.29.21.lif - 884_HFC_Ant_Hip_DG_crest_40X (RGB).tif]

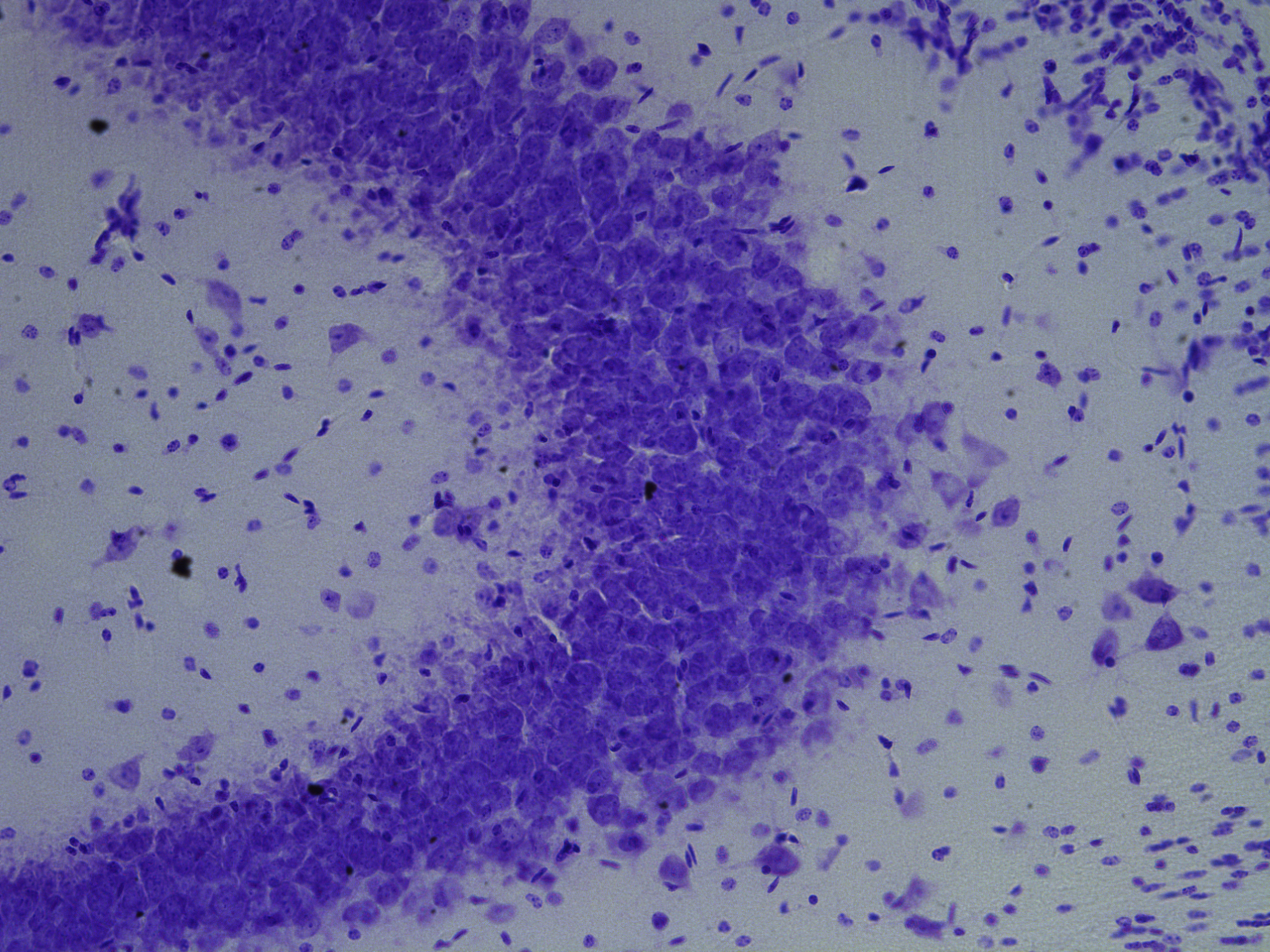

Supplement: Supplementary file 10 — Source data Fig. 5A-B [file 44319_2024_218_MOESM10_ESM.zip › Figure 5 A-B/5B/cdKO/cdKO CA3, NISSL_Ant_Hip_2mo_CKO_BF_20X_Region_40X_Pygnotic_Cells_Rep_1.29.21.lif - 880_KFC_Ant_Hip_DG_CA3_20X (RGB).tif]

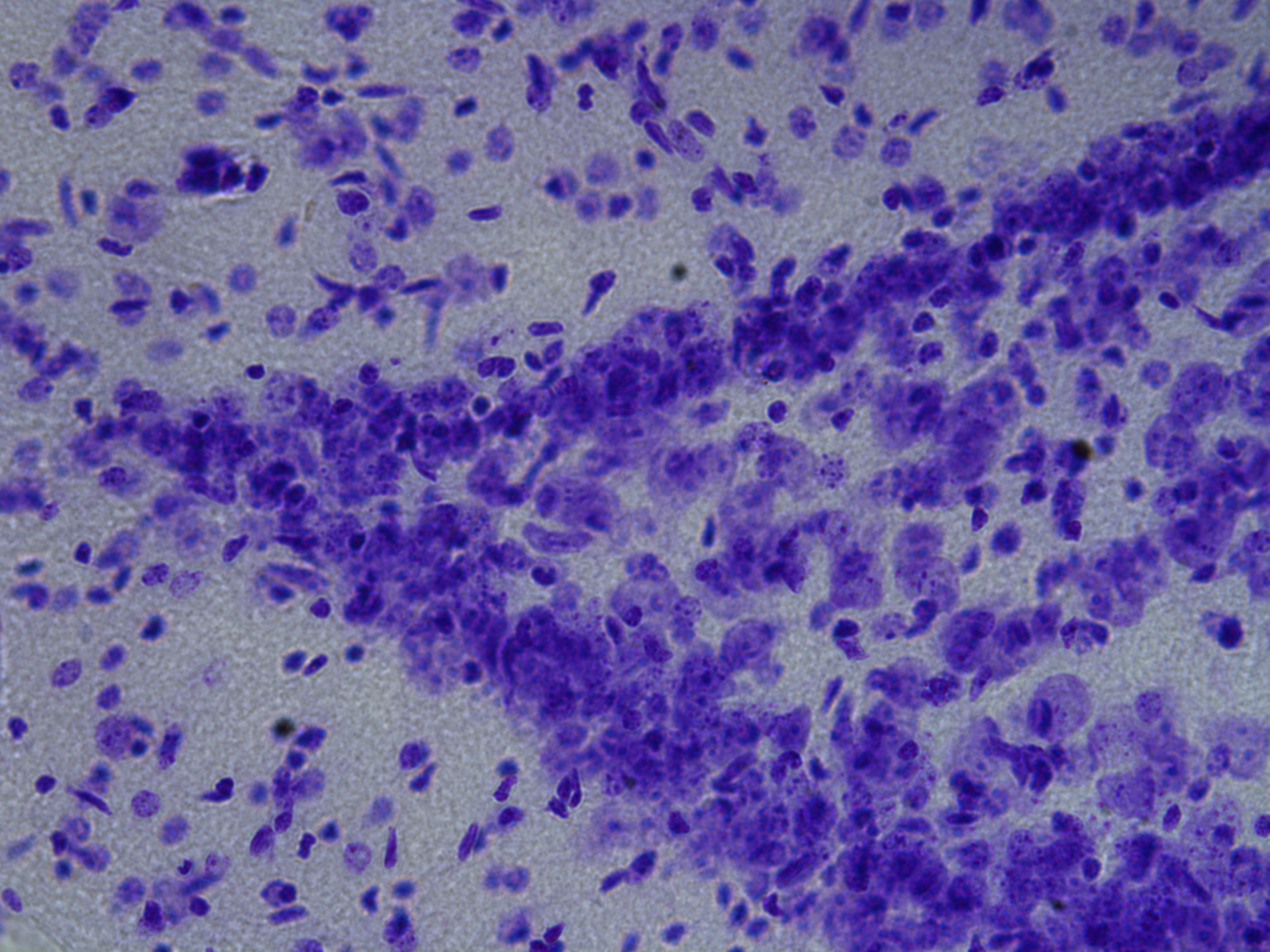

Supplement: Supplementary file 10 — Source data Fig. 5A-B [file 44319_2024_218_MOESM10_ESM.zip › Figure 5 A-B/5B/cdKO/cdKO DG, NISSL_Ant_Hip_2mo_CKO_BF_20X_Region_40X_Pygnotic_Cells_Rep_1.29.21.lif - 880_KFC_Ant_Hip_DG_crest_40X (RGB).tif]

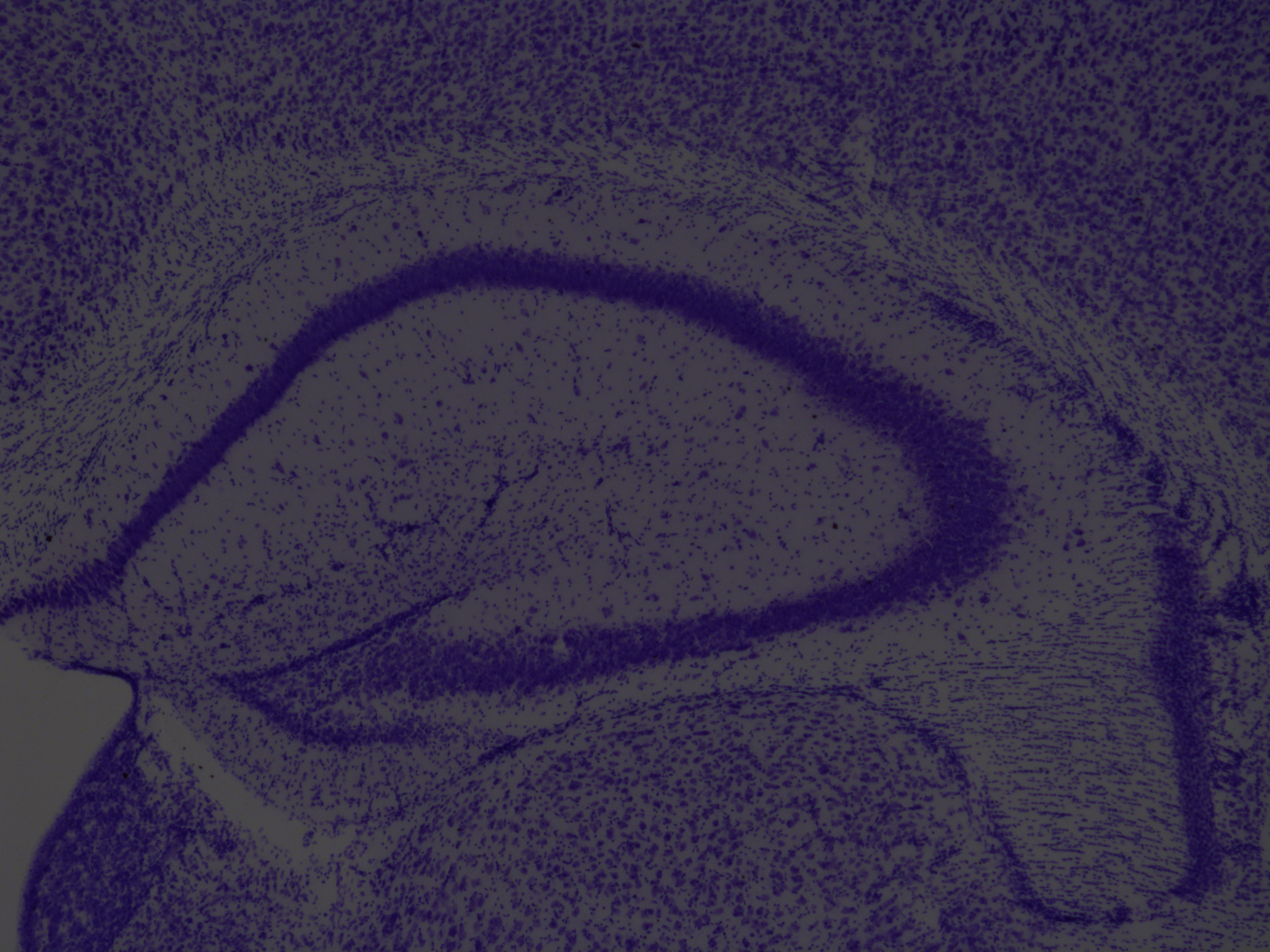

Supplement: Supplementary file 10 — Source data Fig. 5A-B [file 44319_2024_218_MOESM10_ESM.zip › Figure 5 A-B/5B/cdKO/cdKO Hip, NISSL_2mo_D2D3CKO_BF_5X_Overview_1.29.21.lif - 880_KFC_Ant_Hip_5x (RGB).tif]

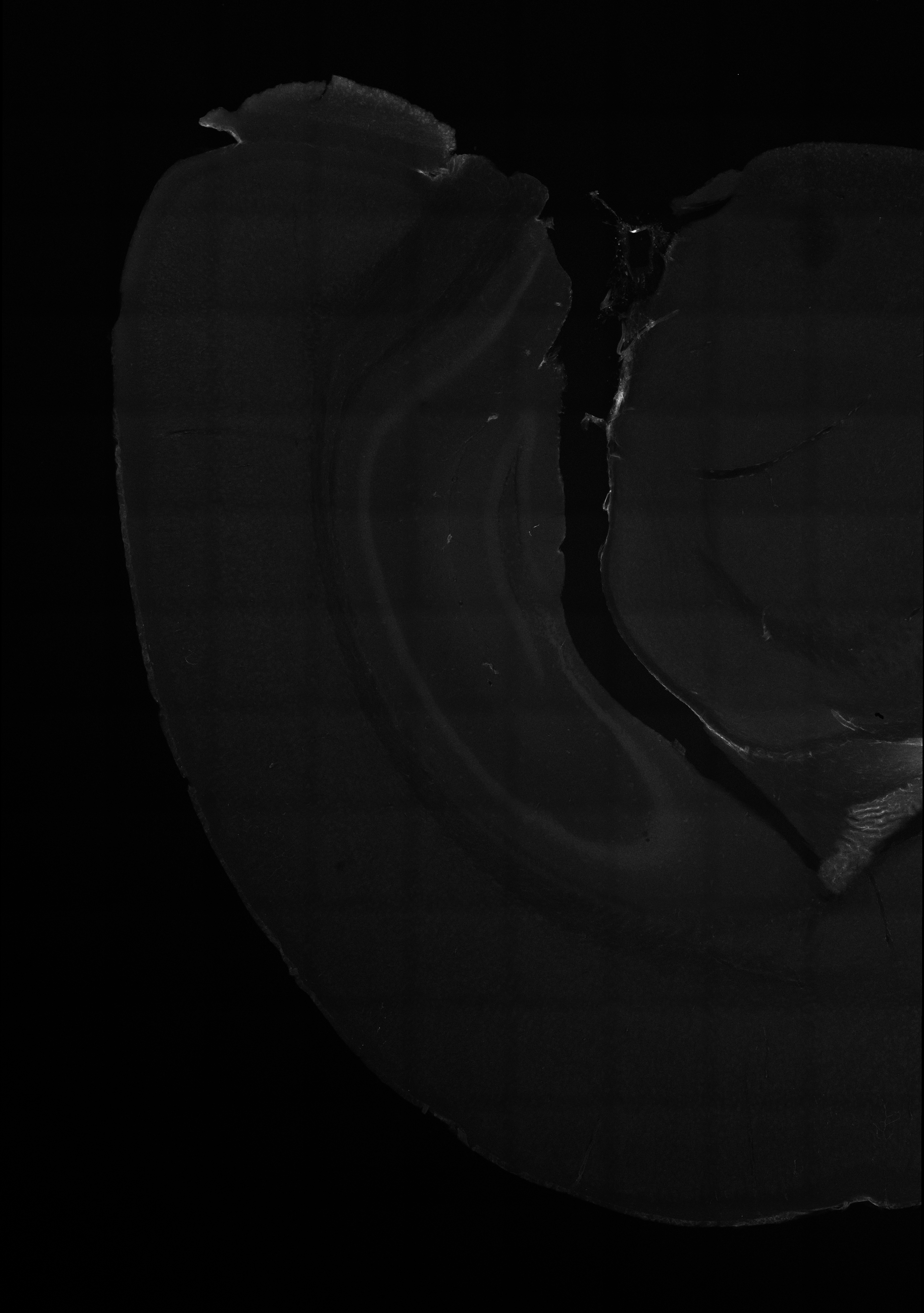

Supplement: Supplementary file 11 — Source data Fig. 5C-E [file 44319_2024_218_MOESM11_ESM.zip › Figure 5 C-D/5C/Ctrl/C4-5w_Cont_20X_TS_5.5.21.lif - Post_Hip_5w_Cont:TileScan_001_Merging_Processed001.tif]

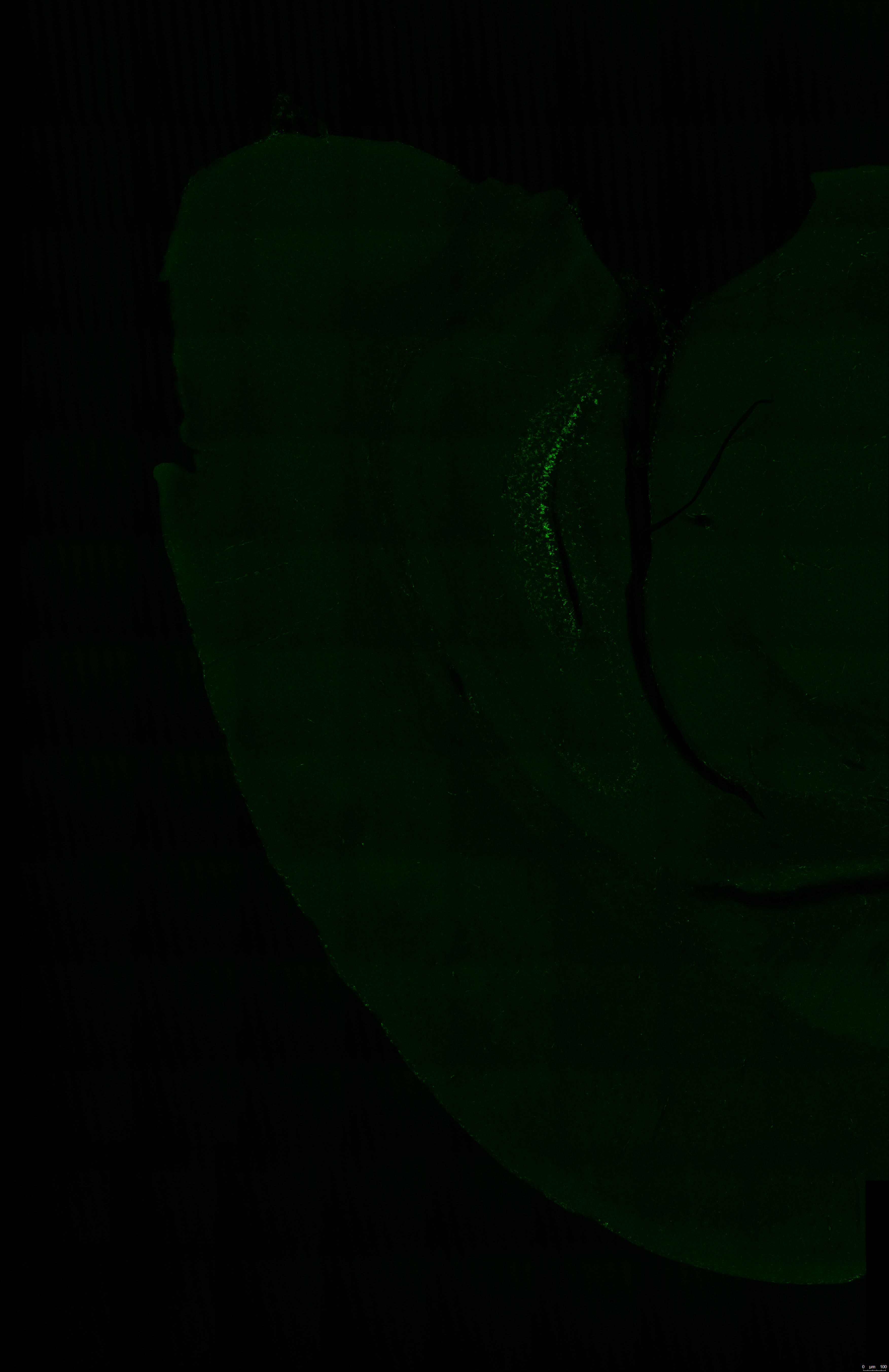

Supplement: Supplementary file 11 — Source data Fig. 5C-E [file 44319_2024_218_MOESM11_ESM.zip › Figure 5 C-D/5C/cdKO/Post_Hip_5w_CKO_TileScan_001_Merging_Processed001_ch01.tif]

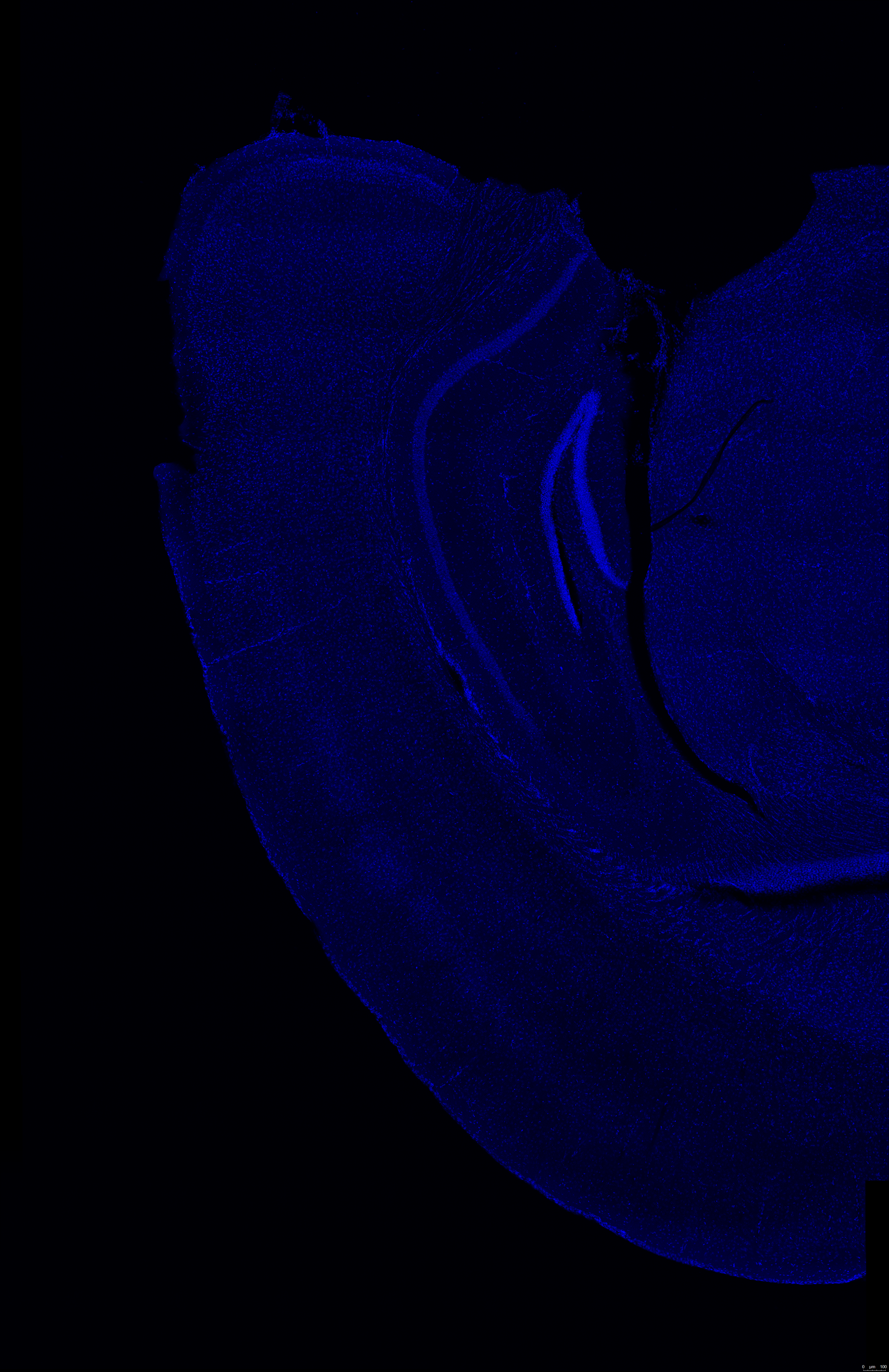

Supplement: Supplementary file 11 — Source data Fig. 5C-E [file 44319_2024_218_MOESM11_ESM.zip › Figure 5 C-D/5C/cdKO/Post_Hip_5w_CKO_TileScan_001_Merging_Processed001_ch00.tif]

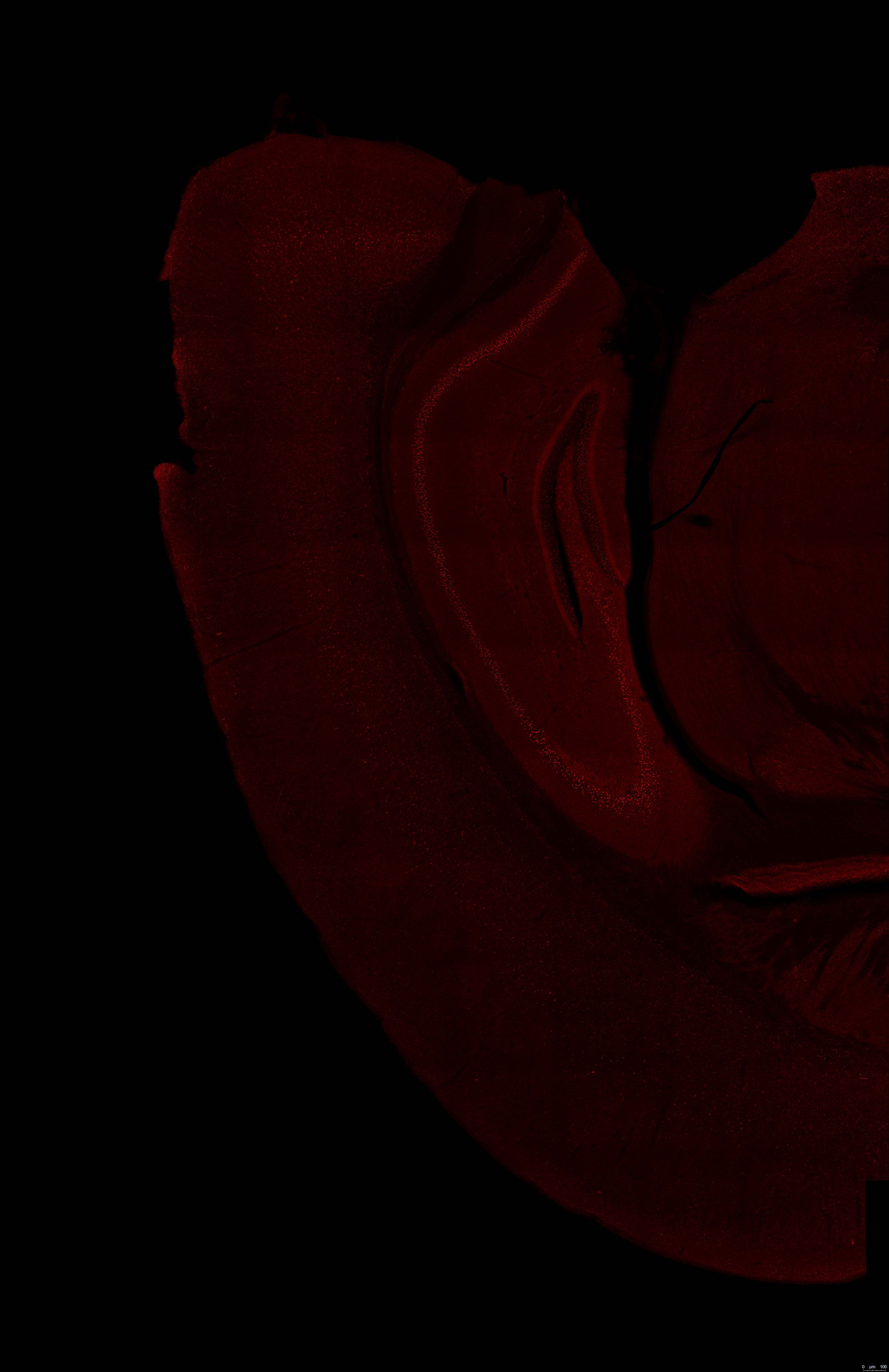

Supplement: Supplementary file 11 — Source data Fig. 5C-E [file 44319_2024_218_MOESM11_ESM.zip › Figure 5 C-D/5C/cdKO/Post_Hip_5w_CKO_TileScan_001_Merging_Processed001_ch02.tif]

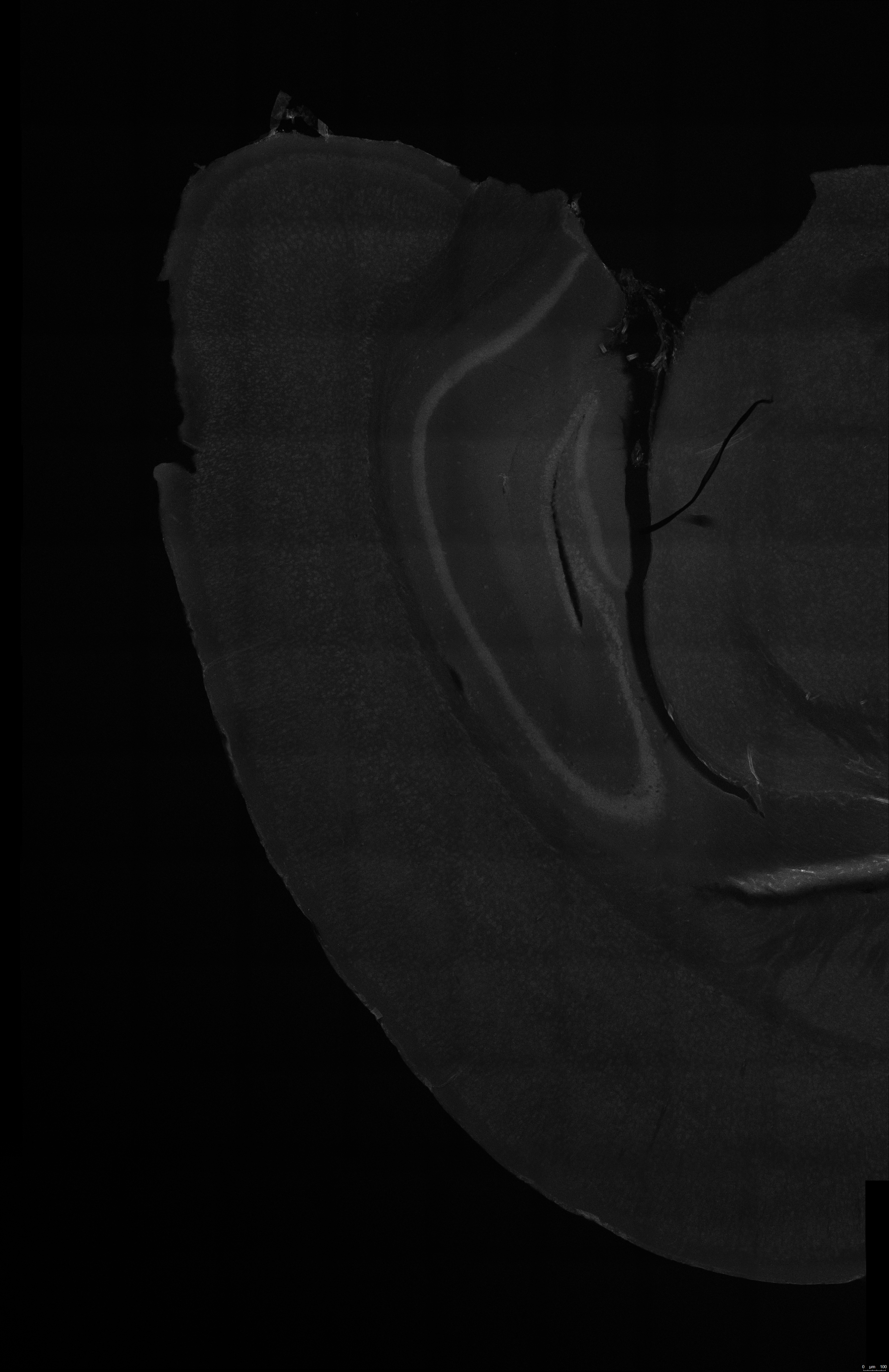

Supplement: Supplementary file 11 — Source data Fig. 5C-E [file 44319_2024_218_MOESM11_ESM.zip › Figure 5 C-D/5C/cdKO/Post_Hip_5w_CKO_TileScan_001_Merging_Processed001_ch03.tif]

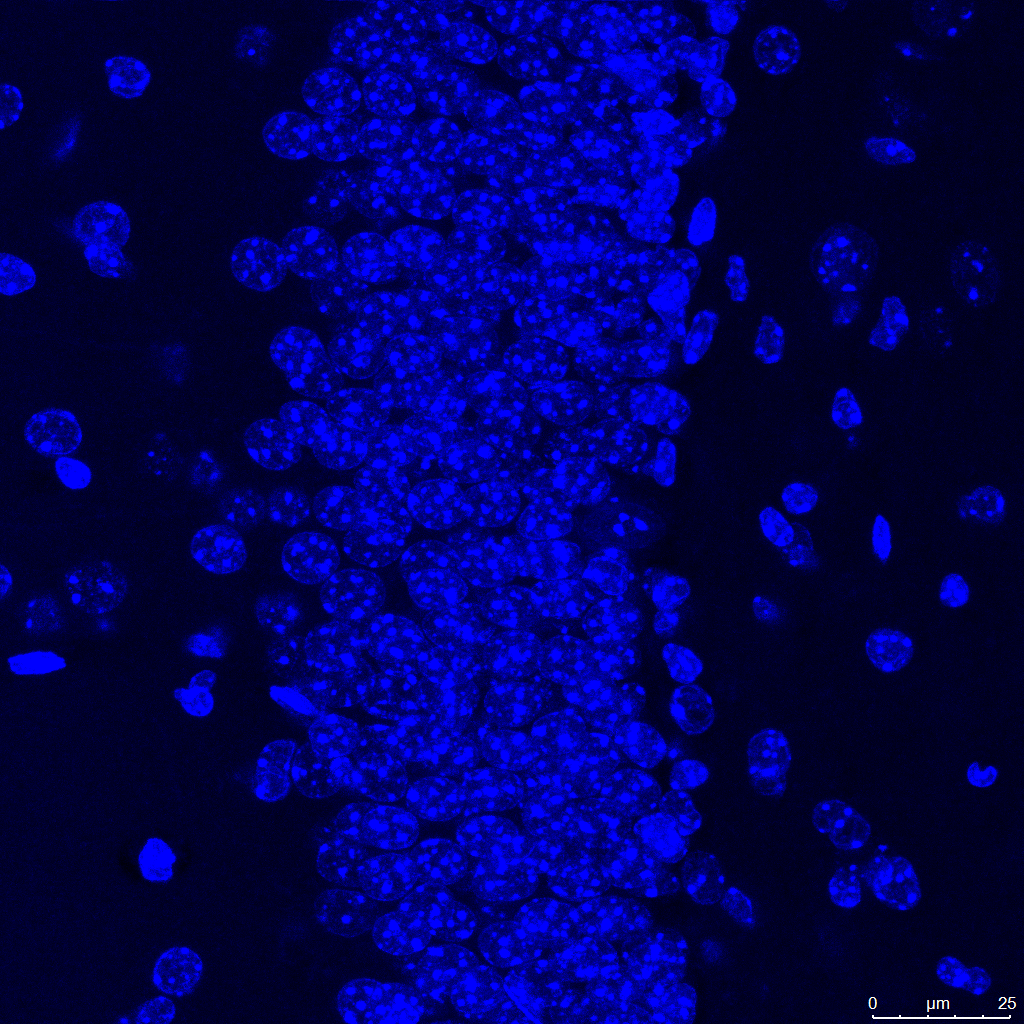

Supplement: Supplementary file 11 — Source data Fig. 5C-E [file 44319_2024_218_MOESM11_ESM.zip › Figure 5 C-D/5C/Ctrl/DG/Post_Cont_Hip_5w_Hip_IMPDH2_555_CD68_488_Casp7_633_5.4.21_KF_5w_DG_SB_Processed001_ch00.tif]

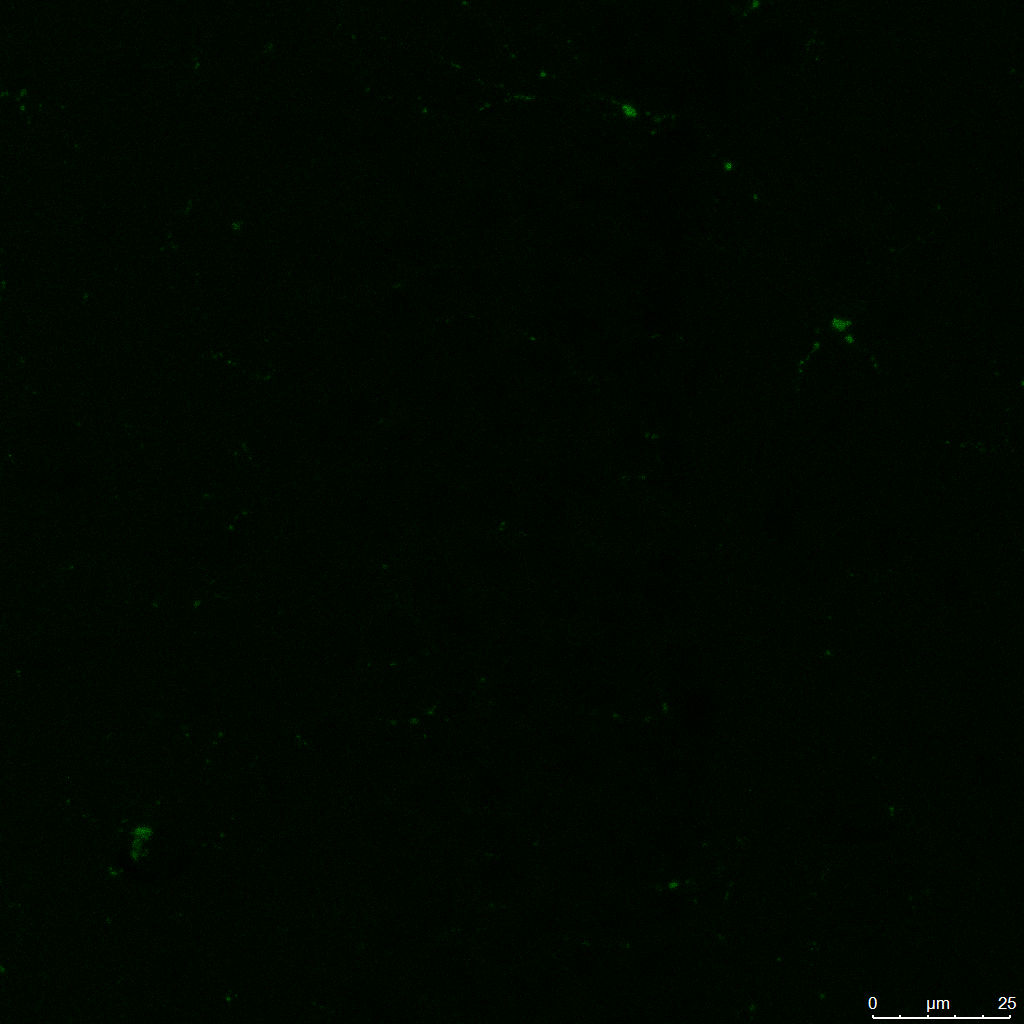

Supplement: Supplementary file 11 — Source data Fig. 5C-E [file 44319_2024_218_MOESM11_ESM.zip › Figure 5 C-D/5C/Ctrl/DG/Post_Cont_Hip_5w_Hip_IMPDH2_555_CD68_488_Casp7_633_5.4.21_KF_5w_DG_SB_Processed001_ch01.tif]

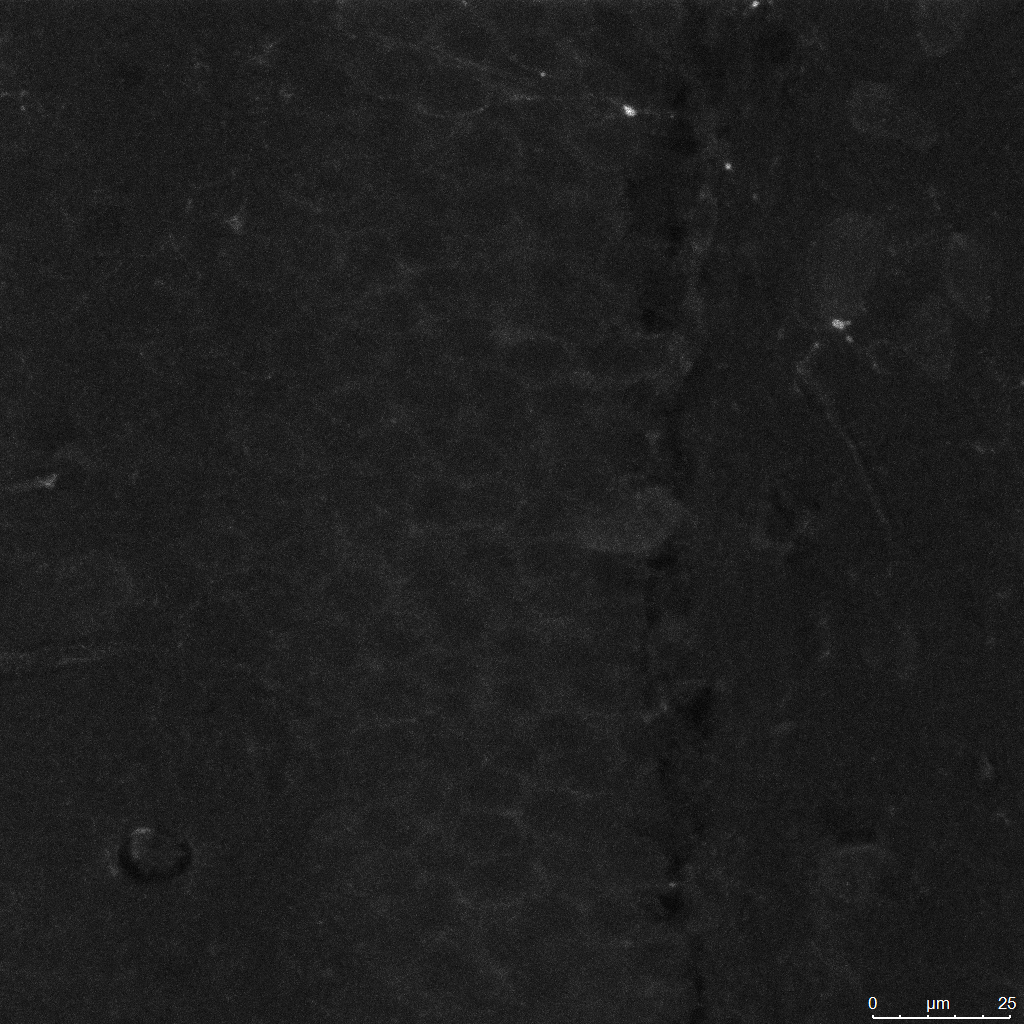

Supplement: Supplementary file 11 — Source data Fig. 5C-E [file 44319_2024_218_MOESM11_ESM.zip › Figure 5 C-D/5C/Ctrl/DG/Post_Cont_Hip_5w_Hip_IMPDH2_555_CD68_488_Casp7_633_5.4.21_KF_5w_DG_SB_Processed001_ch03.tif]

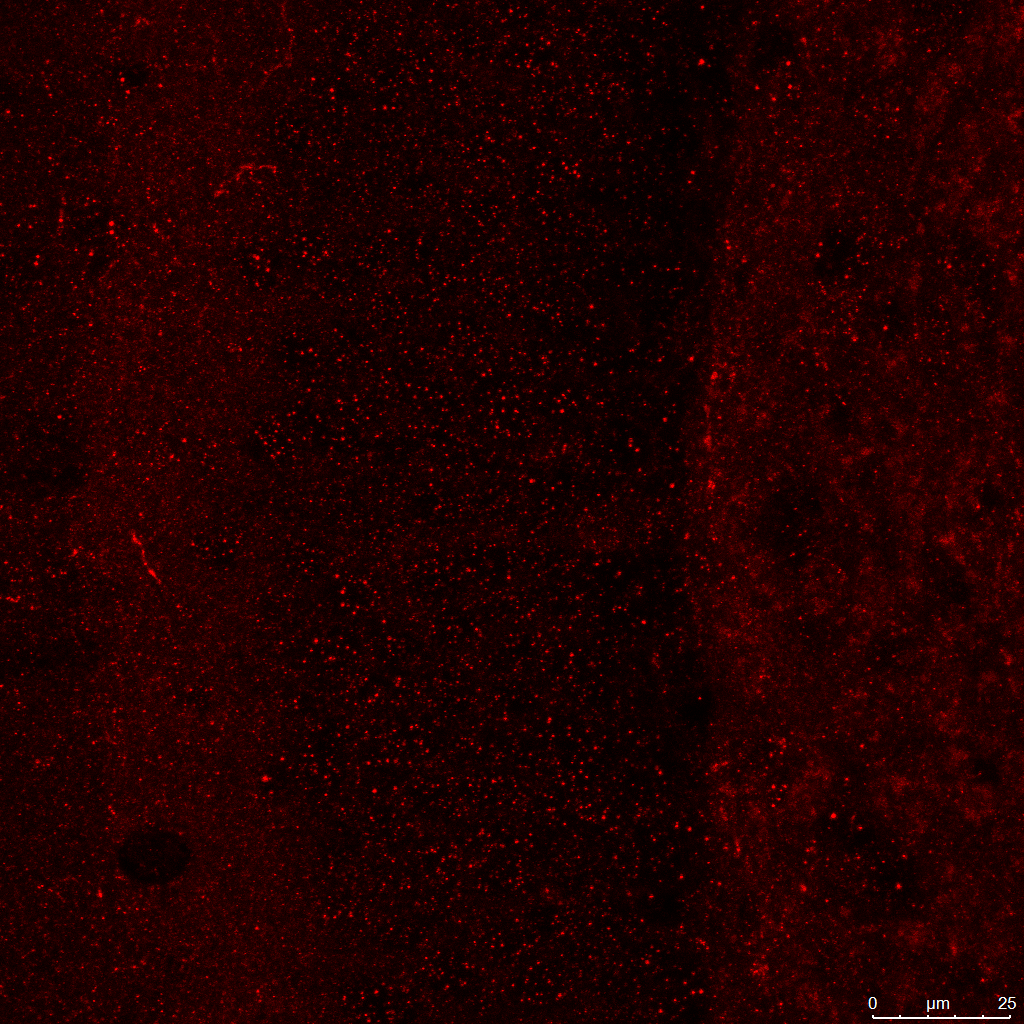

Supplement: Supplementary file 11 — Source data Fig. 5C-E [file 44319_2024_218_MOESM11_ESM.zip › Figure 5 C-D/5C/Ctrl/DG/Post_Cont_Hip_5w_Hip_IMPDH2_555_CD68_488_Casp7_633_5.4.21_KF_5w_DG_SB_Processed001_ch02.tif]

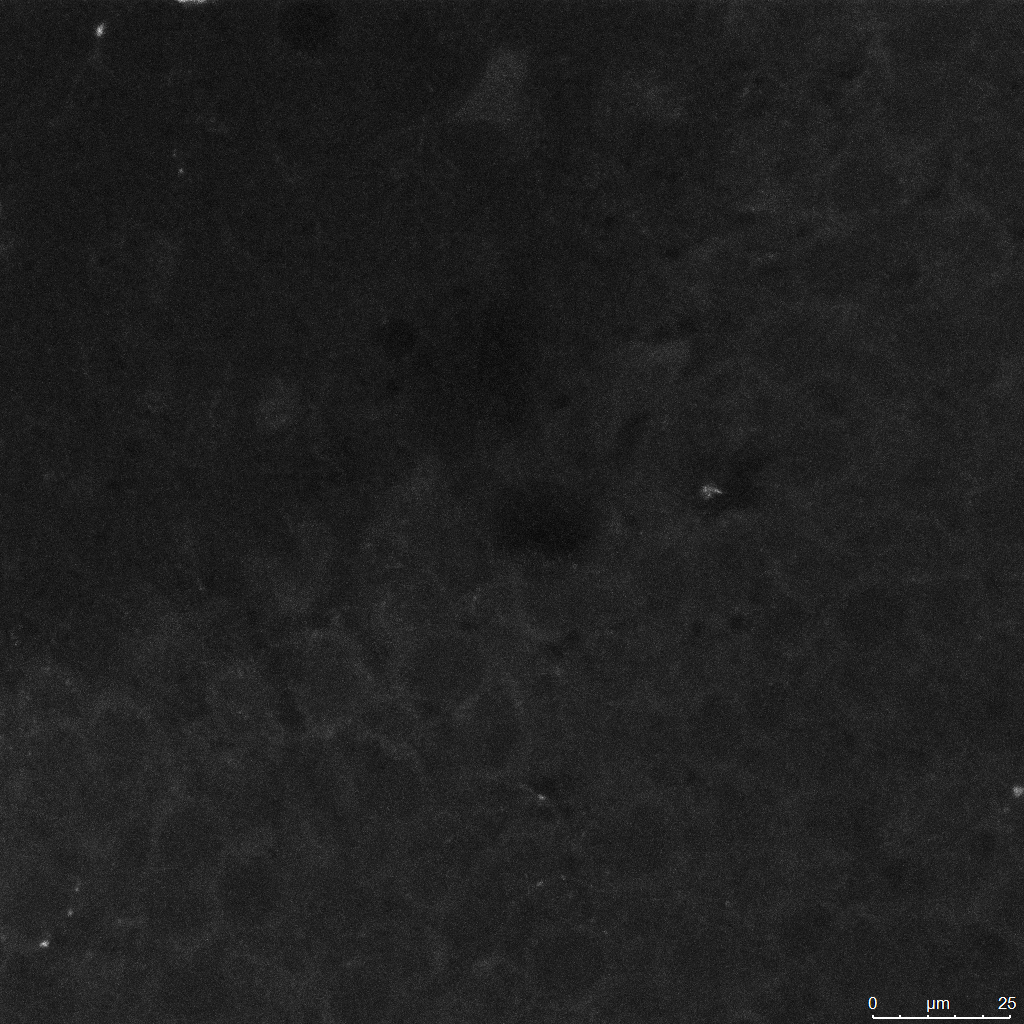

Supplement: Supplementary file 11 — Source data Fig. 5C-E [file 44319_2024_218_MOESM11_ESM.zip › Figure 5 C-D/5C/Ctrl/CA3/Post_Cont_Hip_5w_Hip_IMPDH2_555_CD68_488_Casp7_633_5.4.21_KF_5w_Hip_CA3_Processed001_ch03.tif]

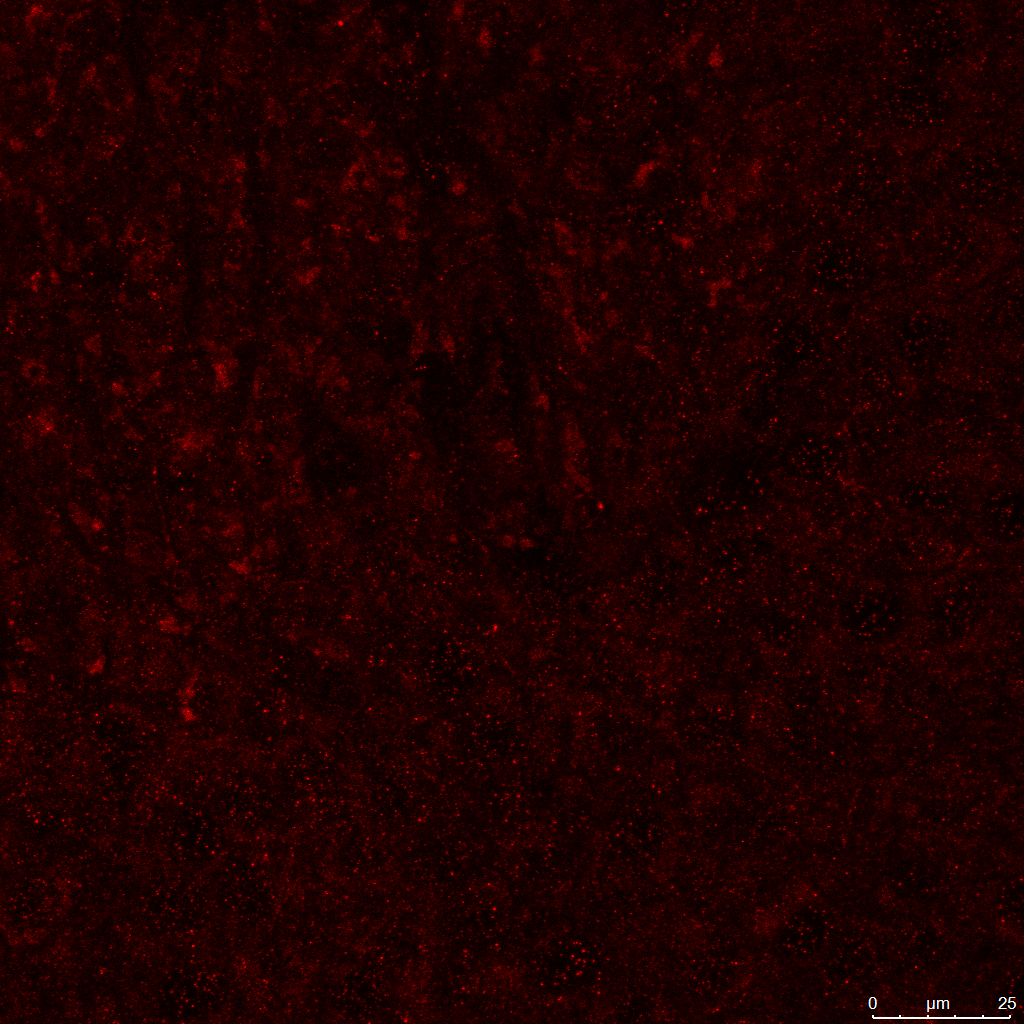

Supplement: Supplementary file 11 — Source data Fig. 5C-E [file 44319_2024_218_MOESM11_ESM.zip › Figure 5 C-D/5C/Ctrl/CA3/Post_Cont_Hip_5w_Hip_IMPDH2_555_CD68_488_Casp7_633_5.4.21_KF_5w_Hip_CA3_Processed001_ch02.tif]

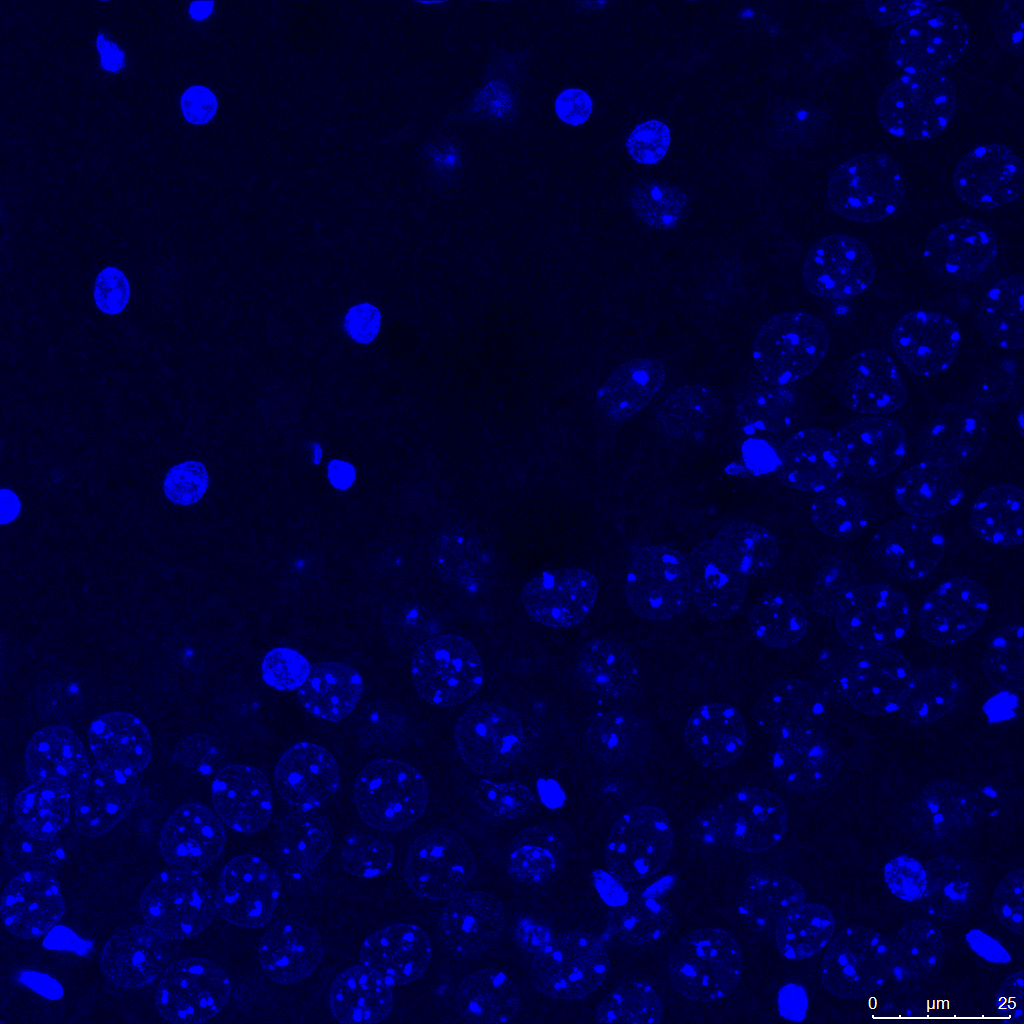

Supplement: Supplementary file 11 — Source data Fig. 5C-E [file 44319_2024_218_MOESM11_ESM.zip › Figure 5 C-D/5C/Ctrl/CA3/Post_Cont_Hip_5w_Hip_IMPDH2_555_CD68_488_Casp7_633_5.4.21_KF_5w_Hip_CA3_Processed001_ch00.tif]

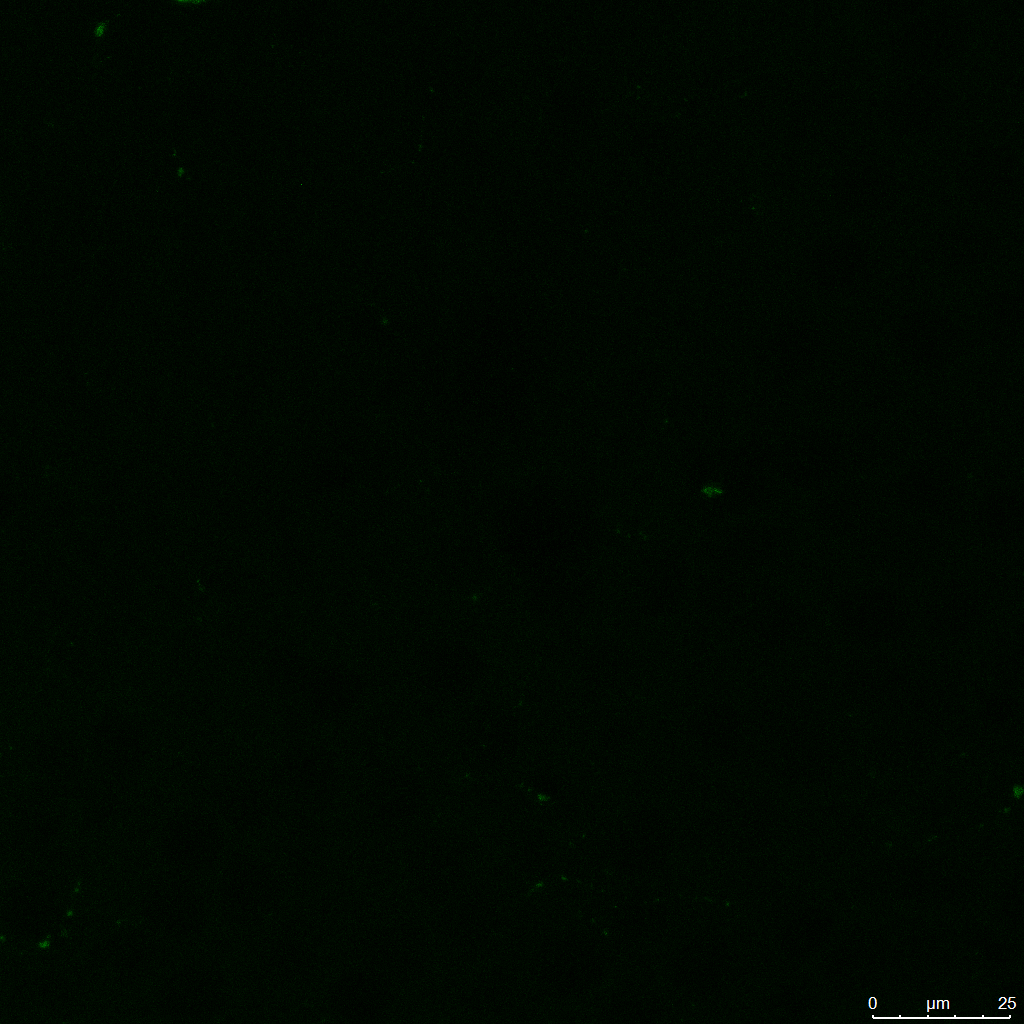

Supplement: Supplementary file 11 — Source data Fig. 5C-E [file 44319_2024_218_MOESM11_ESM.zip › Figure 5 C-D/5C/Ctrl/CA3/Post_Cont_Hip_5w_Hip_IMPDH2_555_CD68_488_Casp7_633_5.4.21_KF_5w_Hip_CA3_Processed001_ch01.tif]

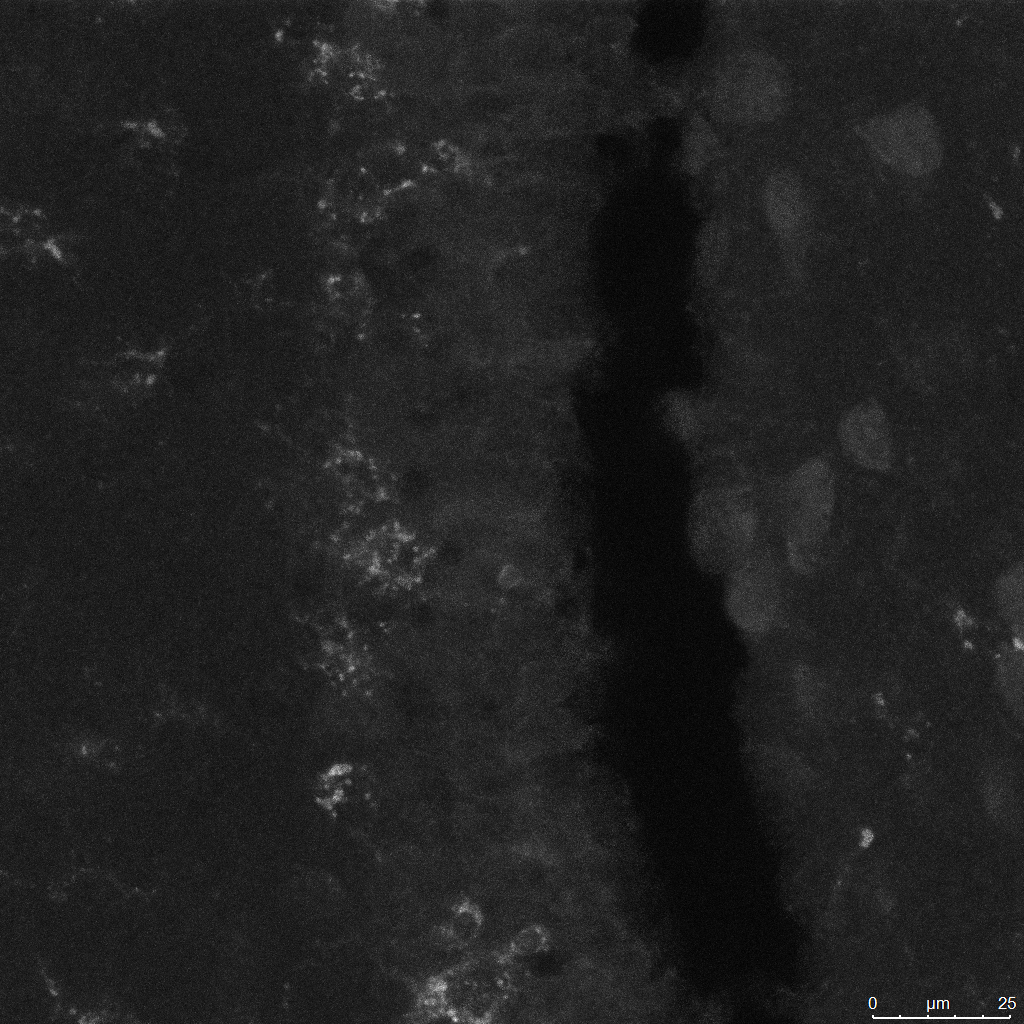

Supplement: Supplementary file 11 — Source data Fig. 5C-E [file 44319_2024_218_MOESM11_ESM.zip › Figure 5 C-D/5C/cdKO/DG/Post_CKO_Hip_5w_IMPDH2_555_CD68_488_Casp7_633_4.30.21_KFC_5w_Post_hip_DG_SB_reopt_Processed001_ch03.tif]

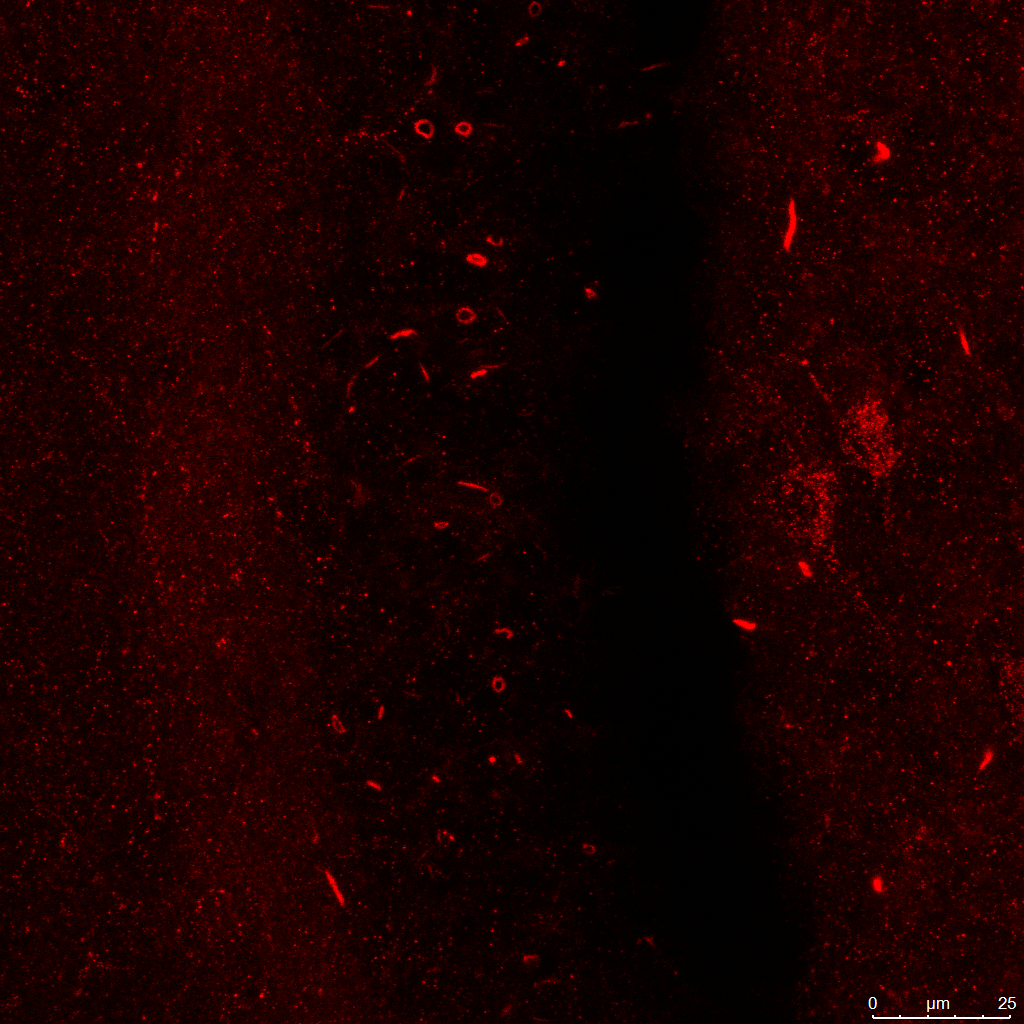

Supplement: Supplementary file 11 — Source data Fig. 5C-E [file 44319_2024_218_MOESM11_ESM.zip › Figure 5 C-D/5C/cdKO/DG/Post_CKO_Hip_5w_IMPDH2_555_CD68_488_Casp7_633_4.30.21_KFC_5w_Post_hip_DG_SB_reopt_Processed001_ch02.tif]

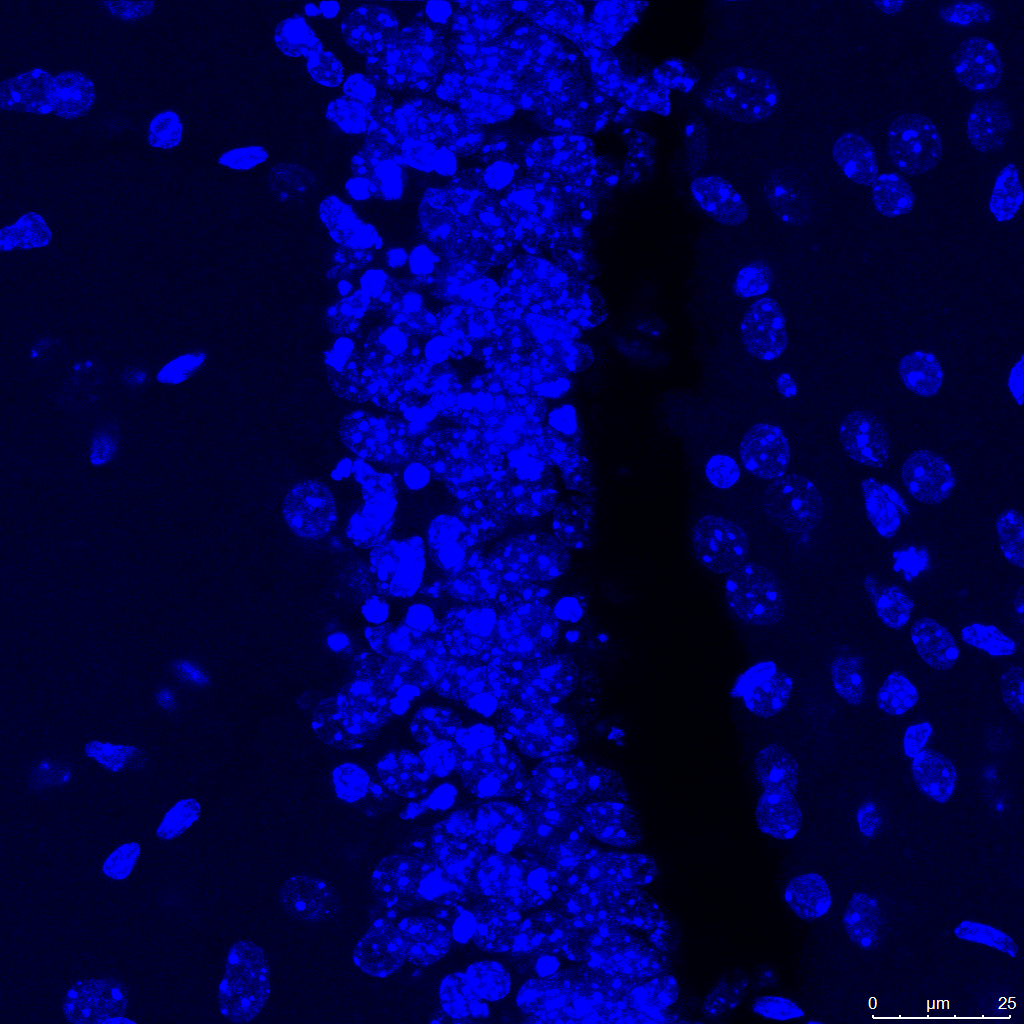

Supplement: Supplementary file 11 — Source data Fig. 5C-E [file 44319_2024_218_MOESM11_ESM.zip › Figure 5 C-D/5C/cdKO/DG/Post_CKO_Hip_5w_IMPDH2_555_CD68_488_Casp7_633_4.30.21_KFC_5w_Post_hip_DG_SB_reopt_Processed001_ch00.tif]

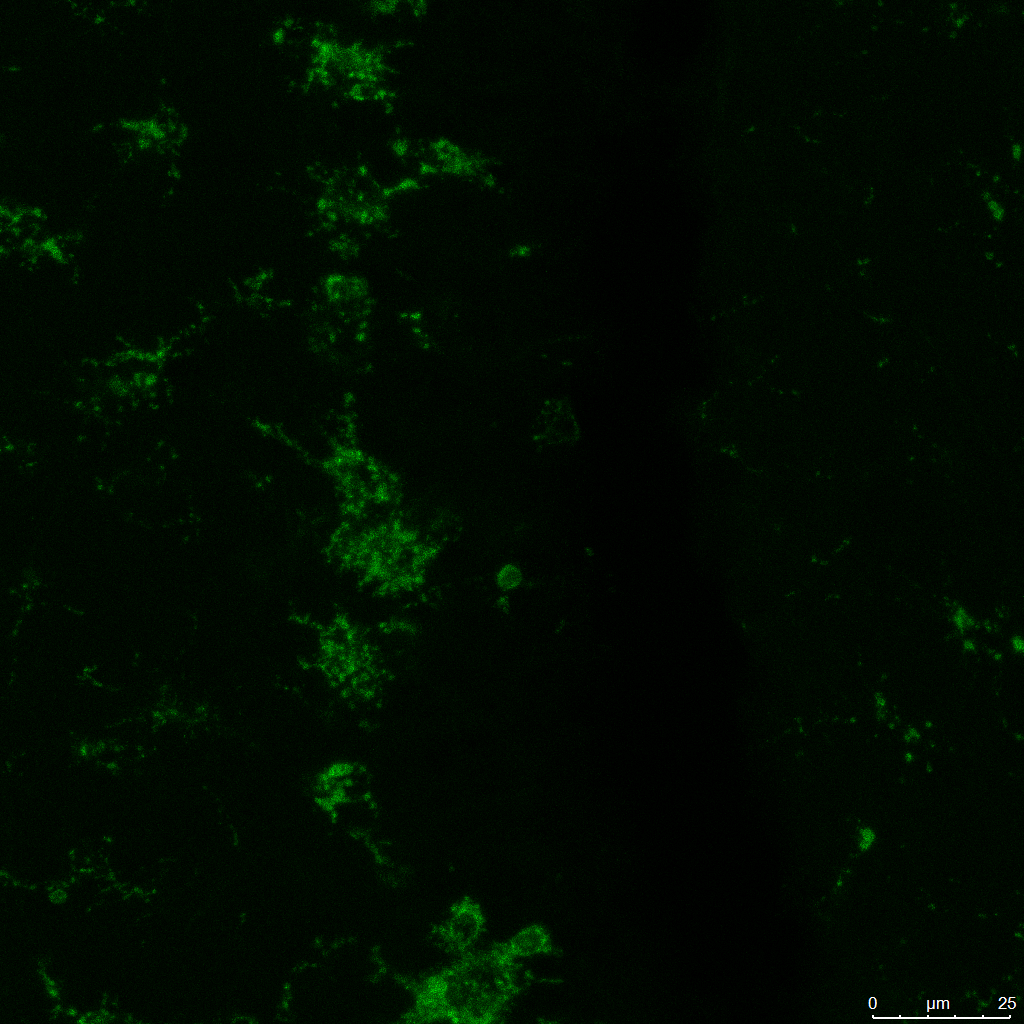

Supplement: Supplementary file 11 — Source data Fig. 5C-E [file 44319_2024_218_MOESM11_ESM.zip › Figure 5 C-D/5C/cdKO/DG/Post_CKO_Hip_5w_IMPDH2_555_CD68_488_Casp7_633_4.30.21_KFC_5w_Post_hip_DG_SB_reopt_Processed001_ch01.tif]

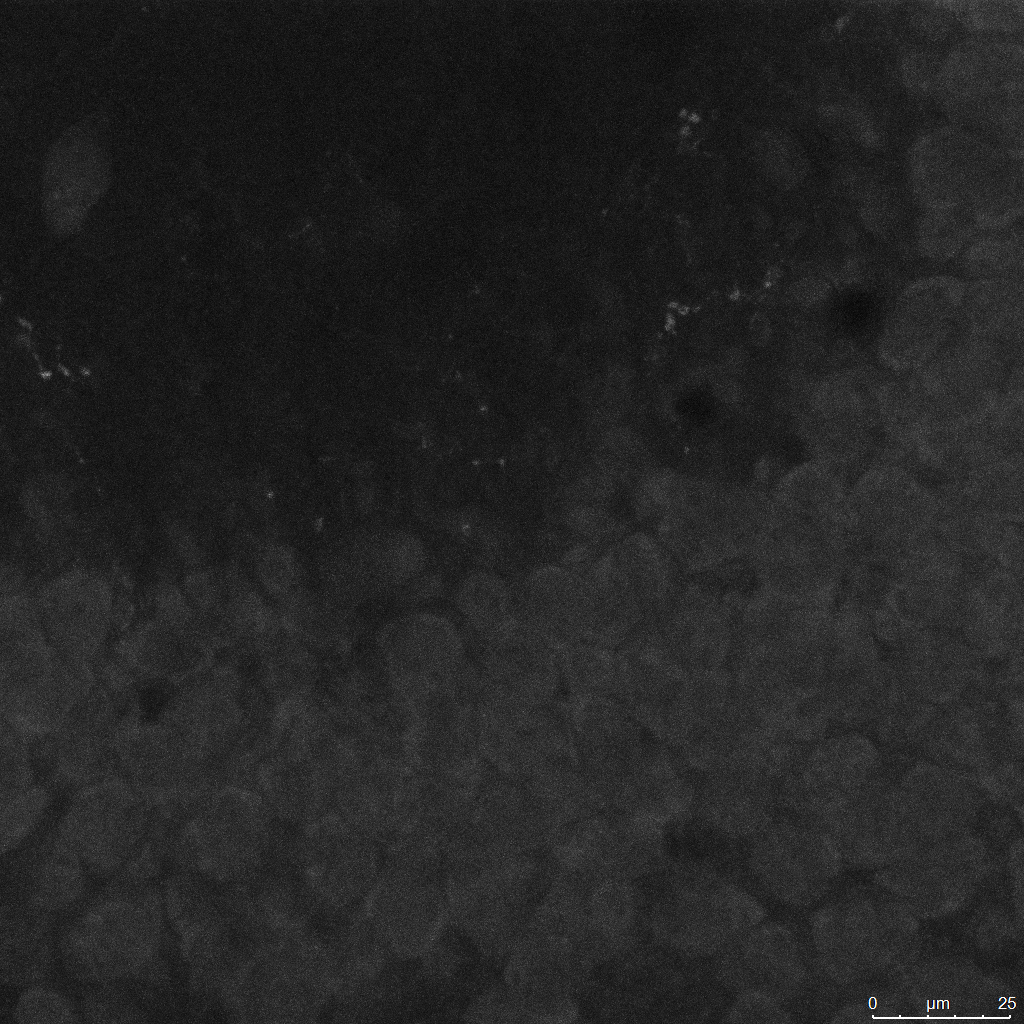

Supplement: Supplementary file 11 — Source data Fig. 5C-E [file 44319_2024_218_MOESM11_ESM.zip › Figure 5 C-D/5C/cdKO/CA3/Post_CKO_Hip_5w_IMPDH2_555_CD68_488_Casp7_633_4.30.21_KFC_5w_CA3_reopt_Processed001_ch03.tif]

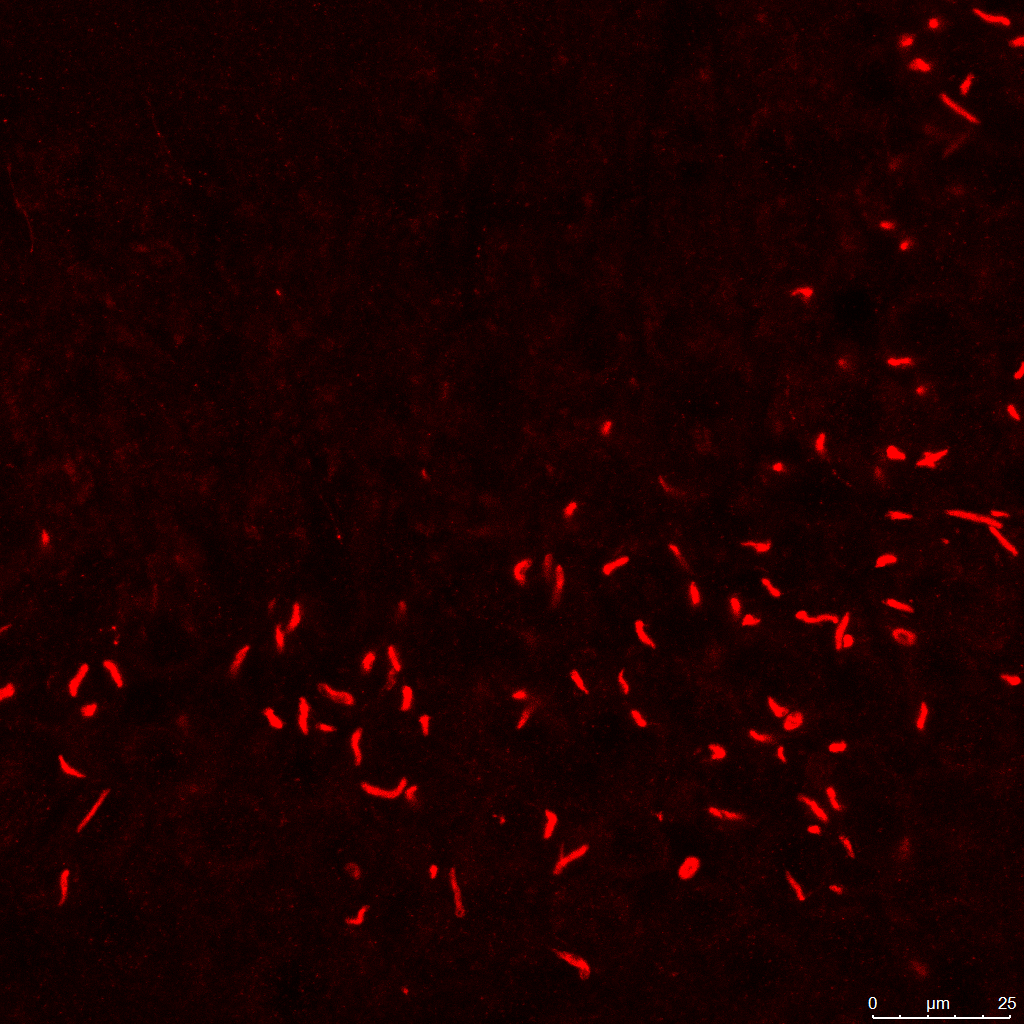

Supplement: Supplementary file 11 — Source data Fig. 5C-E [file 44319_2024_218_MOESM11_ESM.zip › Figure 5 C-D/5C/cdKO/CA3/Post_CKO_Hip_5w_IMPDH2_555_CD68_488_Casp7_633_4.30.21_KFC_5w_CA3_reopt_Processed001_ch02.tif]

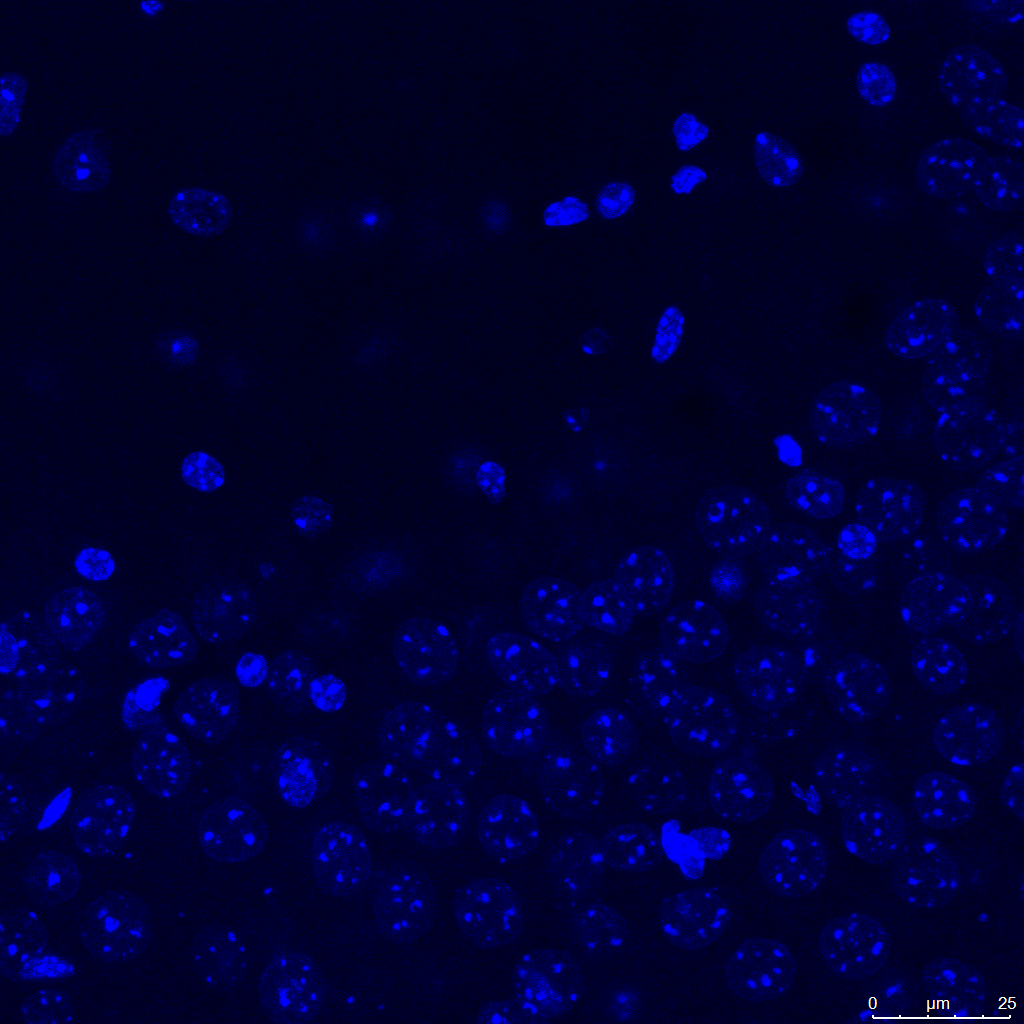

Supplement: Supplementary file 11 — Source data Fig. 5C-E [file 44319_2024_218_MOESM11_ESM.zip › Figure 5 C-D/5C/cdKO/CA3/Post_CKO_Hip_5w_IMPDH2_555_CD68_488_Casp7_633_4.30.21_KFC_5w_CA3_reopt_Processed001_ch00.tif]

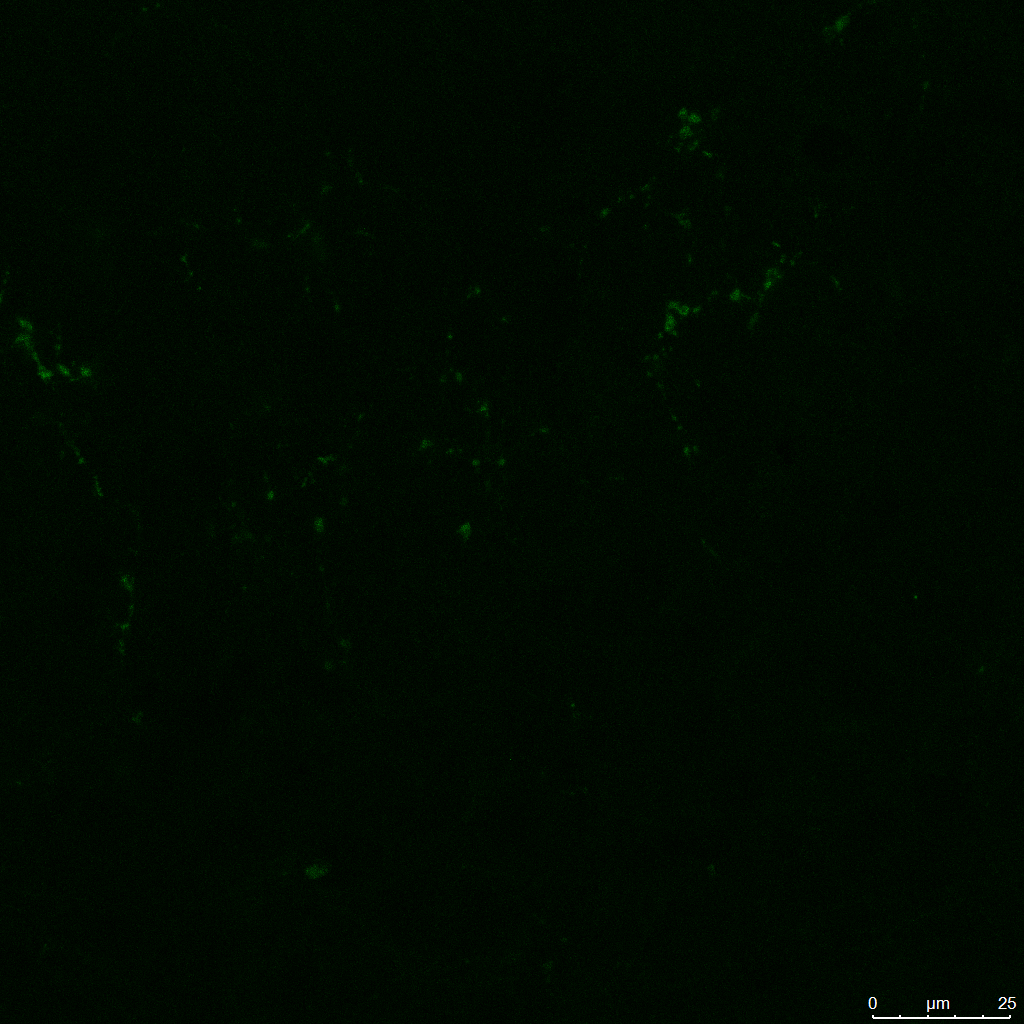

Supplement: Supplementary file 11 — Source data Fig. 5C-E [file 44319_2024_218_MOESM11_ESM.zip › Figure 5 C-D/5C/cdKO/CA3/Post_CKO_Hip_5w_IMPDH2_555_CD68_488_Casp7_633_4.30.21_KFC_5w_CA3_reopt_Processed001_ch01.tif]

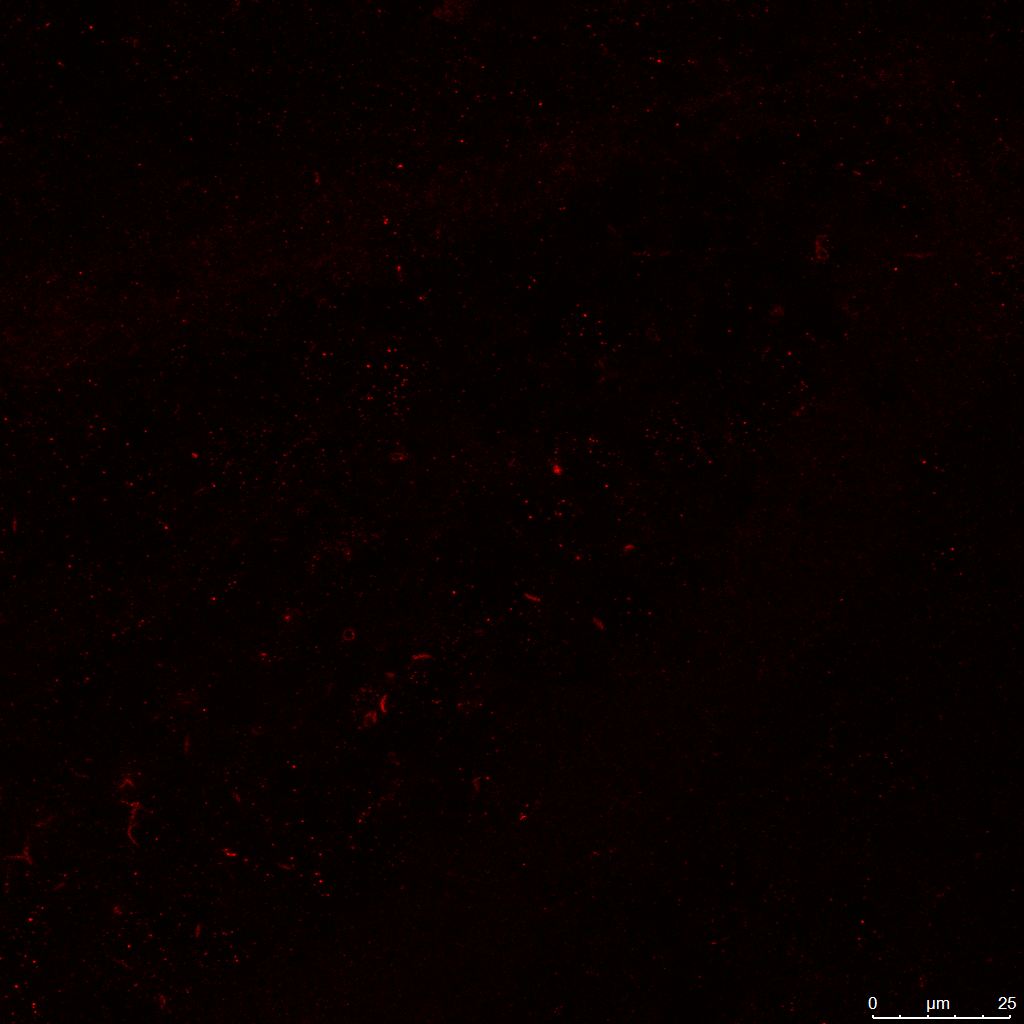

Supplement: Supplementary file 11 — Source data Fig. 5C-E [file 44319_2024_218_MOESM11_ESM.zip › Figure 5 C-D/5D/cdKO 5w/DG/IMPDH2_RR_Acc_63X_5w_CKO_Rep_IMPDH2_555_NeuN_633_GFAP_488_1.5.20_917_KFC_ant_hip_DG_Crest_Processed001_ch02.tif]

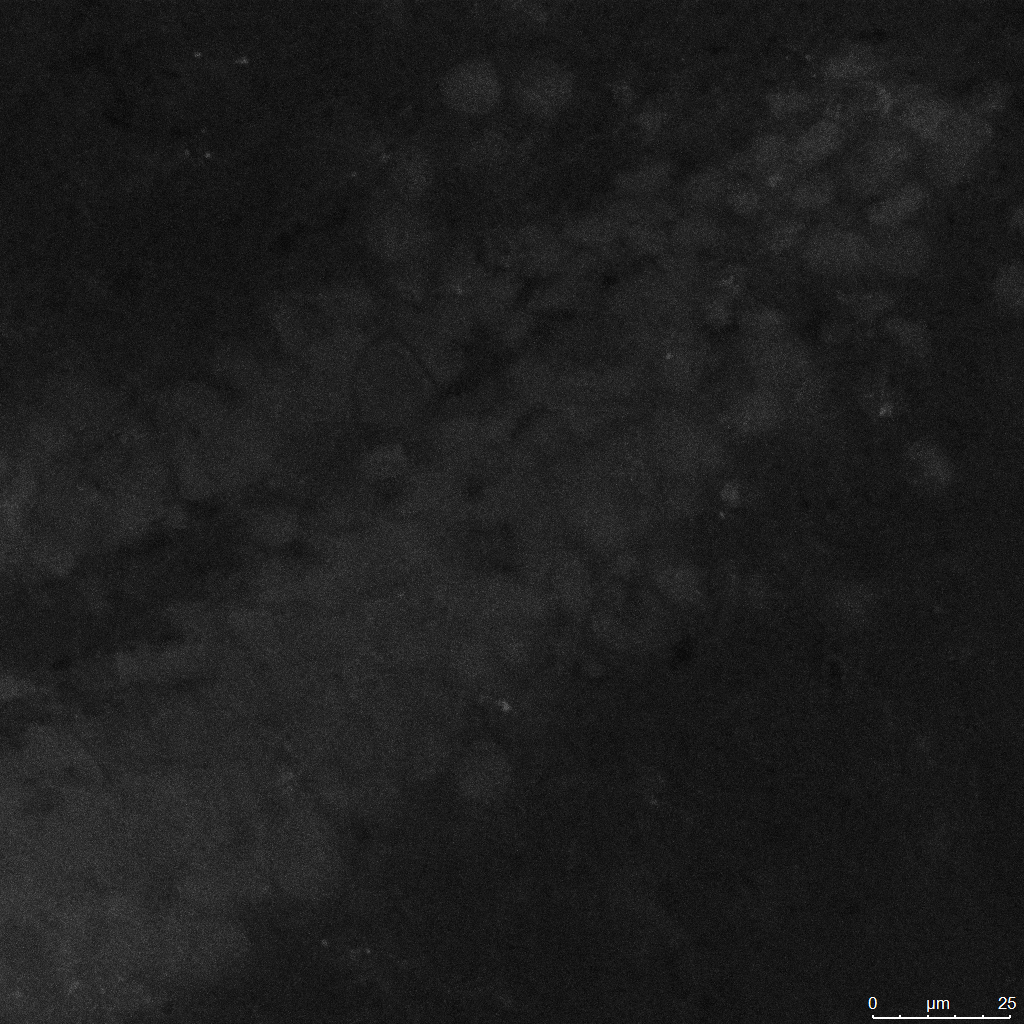

Supplement: Supplementary file 11 — Source data Fig. 5C-E [file 44319_2024_218_MOESM11_ESM.zip › Figure 5 C-D/5D/cdKO 5w/DG/IMPDH2_RR_Acc_63X_5w_CKO_Rep_IMPDH2_555_NeuN_633_GFAP_488_1.5.20_917_KFC_ant_hip_DG_Crest_Processed001_ch03.tif]
