# Supplementary figures and images for: IMPDH2 filaments protect from neurodegeneration in AMPD2 deficiency (part 2 of 2)
Source: EMBO Rep. 2024 Jul 29;25(9):16. doi: 10.1038/s44319-024-00218-2 (PMC11387764; doi:10.1038/s44319-024-00218-2)

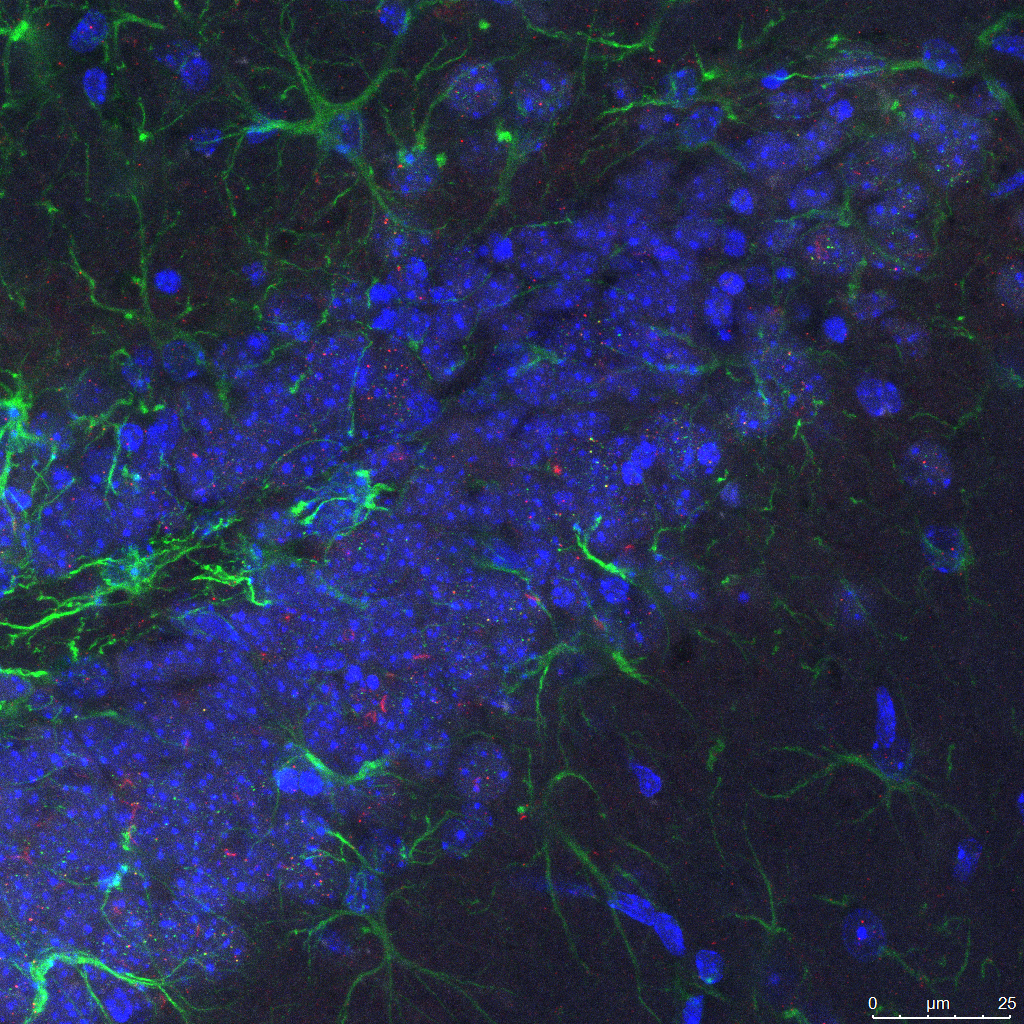

Supplement: Supplementary file 11 — Source data Fig. 5C-E [file 44319_2024_218_MOESM11_ESM.zip › Figure 5 C-D/5D/cdKO 5w/DG/IMPDH2_RR_Acc_63X_5w_CKO_Rep_IMPDH2_555_NeuN_633_GFAP_488_1.5.20_917_KFC_ant_hip_DG_Crest_Processed001.tif]

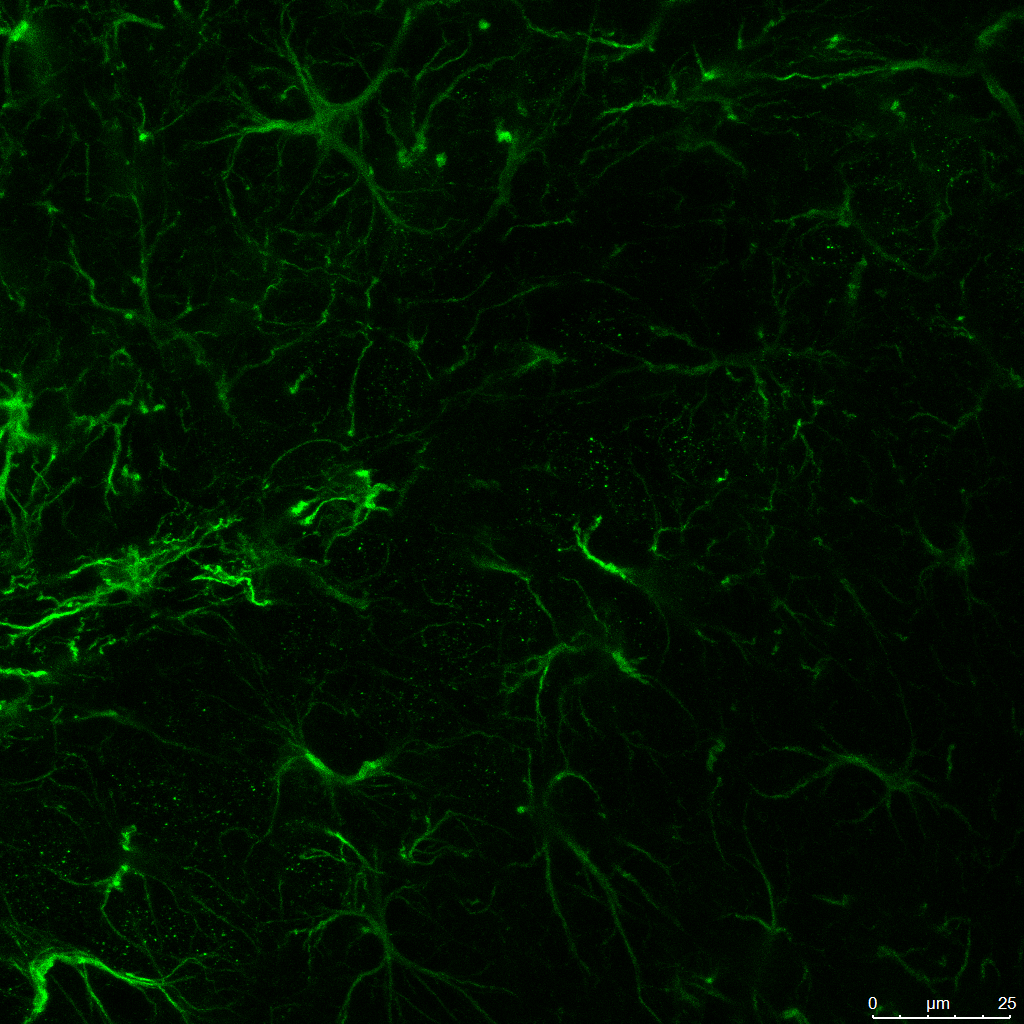

Supplement: Supplementary file 11 — Source data Fig. 5C-E [file 44319_2024_218_MOESM11_ESM.zip › Figure 5 C-D/5D/cdKO 5w/DG/IMPDH2_RR_Acc_63X_5w_CKO_Rep_IMPDH2_555_NeuN_633_GFAP_488_1.5.20_917_KFC_ant_hip_DG_Crest_Processed001_ch01.tif]

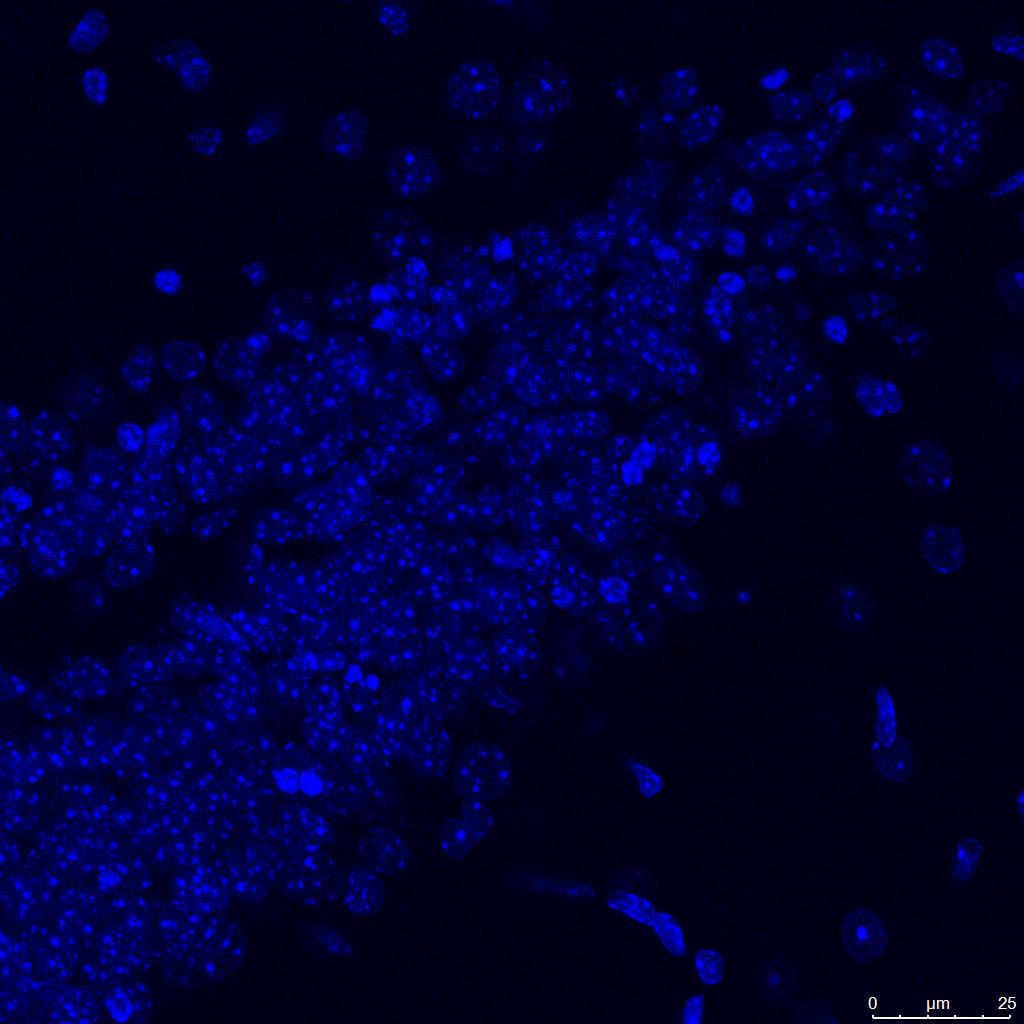

Supplement: Supplementary file 11 — Source data Fig. 5C-E [file 44319_2024_218_MOESM11_ESM.zip › Figure 5 C-D/5D/cdKO 5w/DG/IMPDH2_RR_Acc_63X_5w_CKO_Rep_IMPDH2_555_NeuN_633_GFAP_488_1.5.20_917_KFC_ant_hip_DG_Crest_Processed001_ch00.tif]

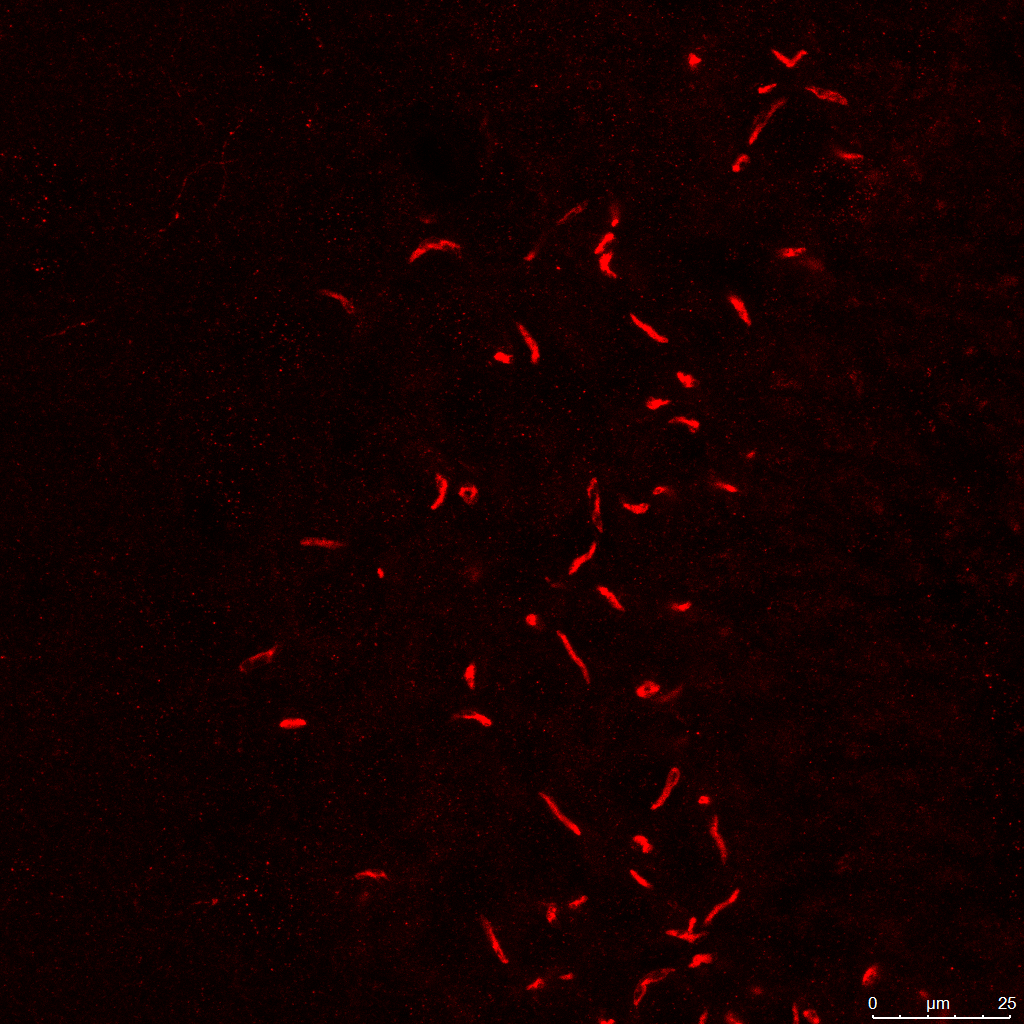

Supplement: Supplementary file 11 — Source data Fig. 5C-E [file 44319_2024_218_MOESM11_ESM.zip › Figure 5 C-D/5D/cdKO 5w/CA3/IMPDH2_RR_Acc_63X_5w_CKO_Rep_IMPDH2_555_NeuN_633_GFAP_488_1.5.20_917_KFC_ant_hip_CA3_Processed001_ch02.tif]

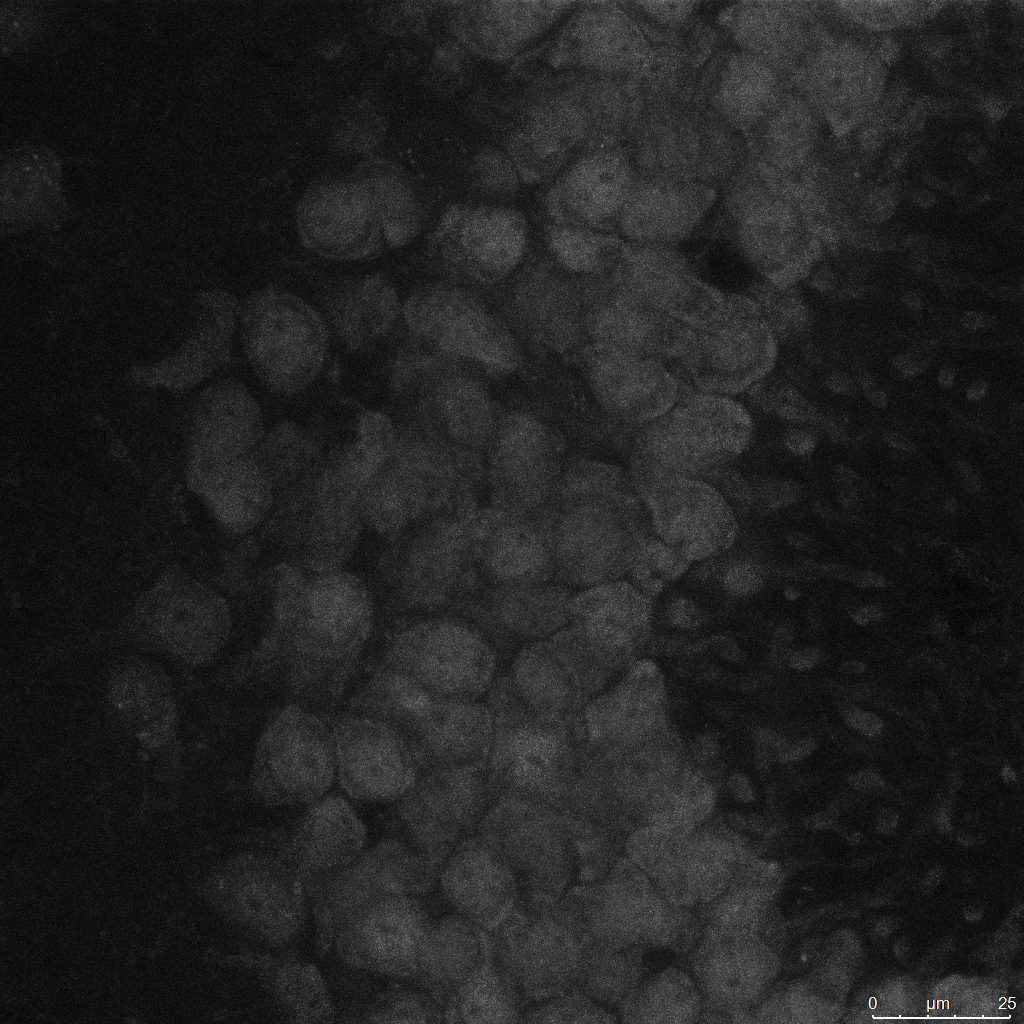

Supplement: Supplementary file 11 — Source data Fig. 5C-E [file 44319_2024_218_MOESM11_ESM.zip › Figure 5 C-D/5D/cdKO 5w/CA3/IMPDH2_RR_Acc_63X_5w_CKO_Rep_IMPDH2_555_NeuN_633_GFAP_488_1.5.20_917_KFC_ant_hip_CA3_Processed001_ch03.tif]

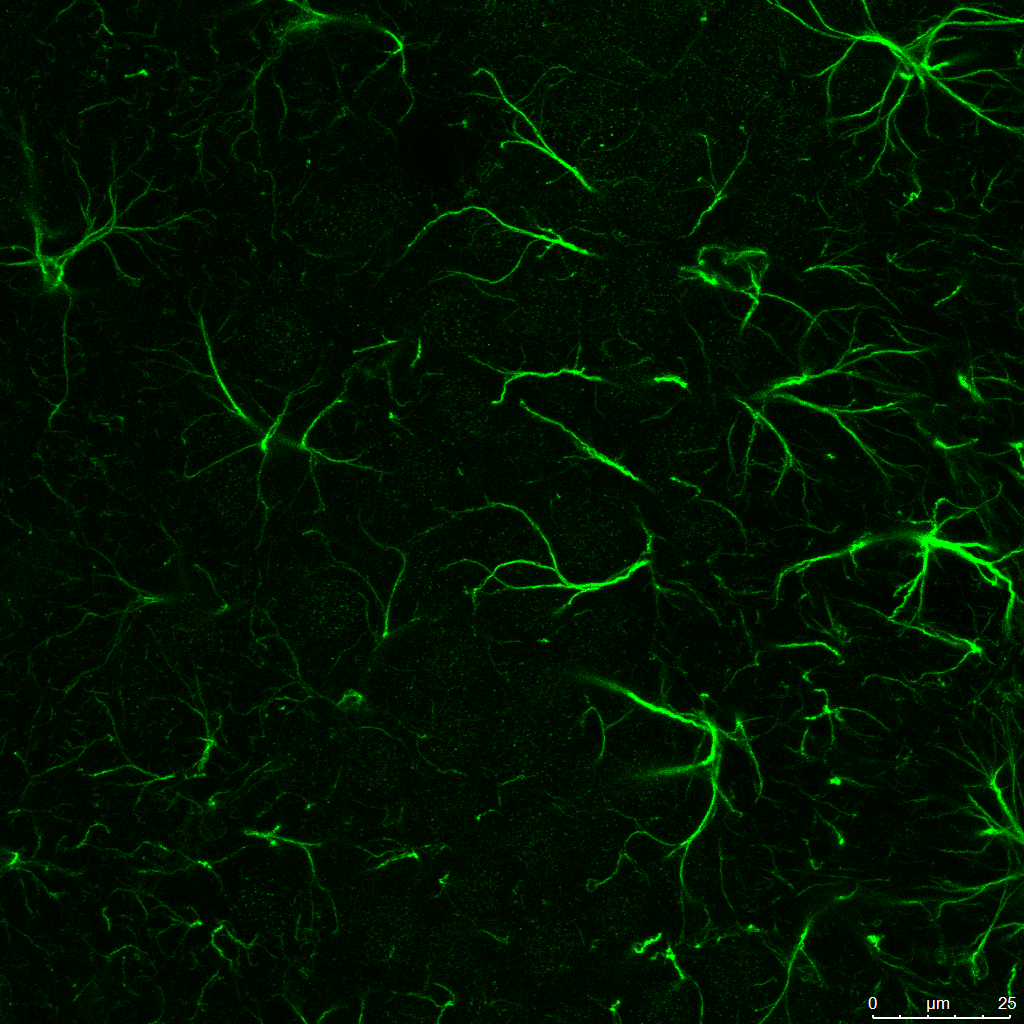

Supplement: Supplementary file 11 — Source data Fig. 5C-E [file 44319_2024_218_MOESM11_ESM.zip › Figure 5 C-D/5D/cdKO 5w/CA3/IMPDH2_RR_Acc_63X_5w_CKO_Rep_IMPDH2_555_NeuN_633_GFAP_488_1.5.20_917_KFC_ant_hip_CA3_Processed001_ch01.tif]

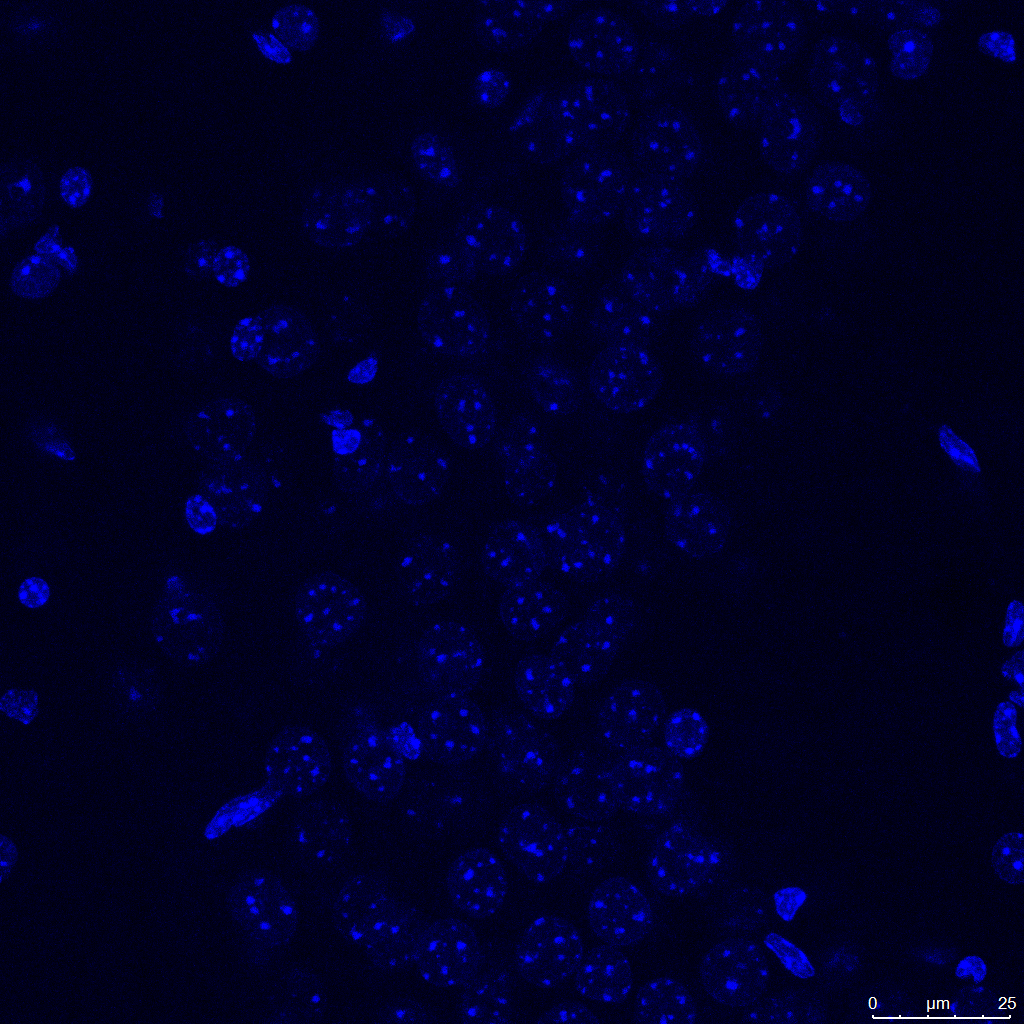

Supplement: Supplementary file 11 — Source data Fig. 5C-E [file 44319_2024_218_MOESM11_ESM.zip › Figure 5 C-D/5D/cdKO 5w/CA3/IMPDH2_RR_Acc_63X_5w_CKO_Rep_IMPDH2_555_NeuN_633_GFAP_488_1.5.20_917_KFC_ant_hip_CA3_Processed001_ch00.tif]

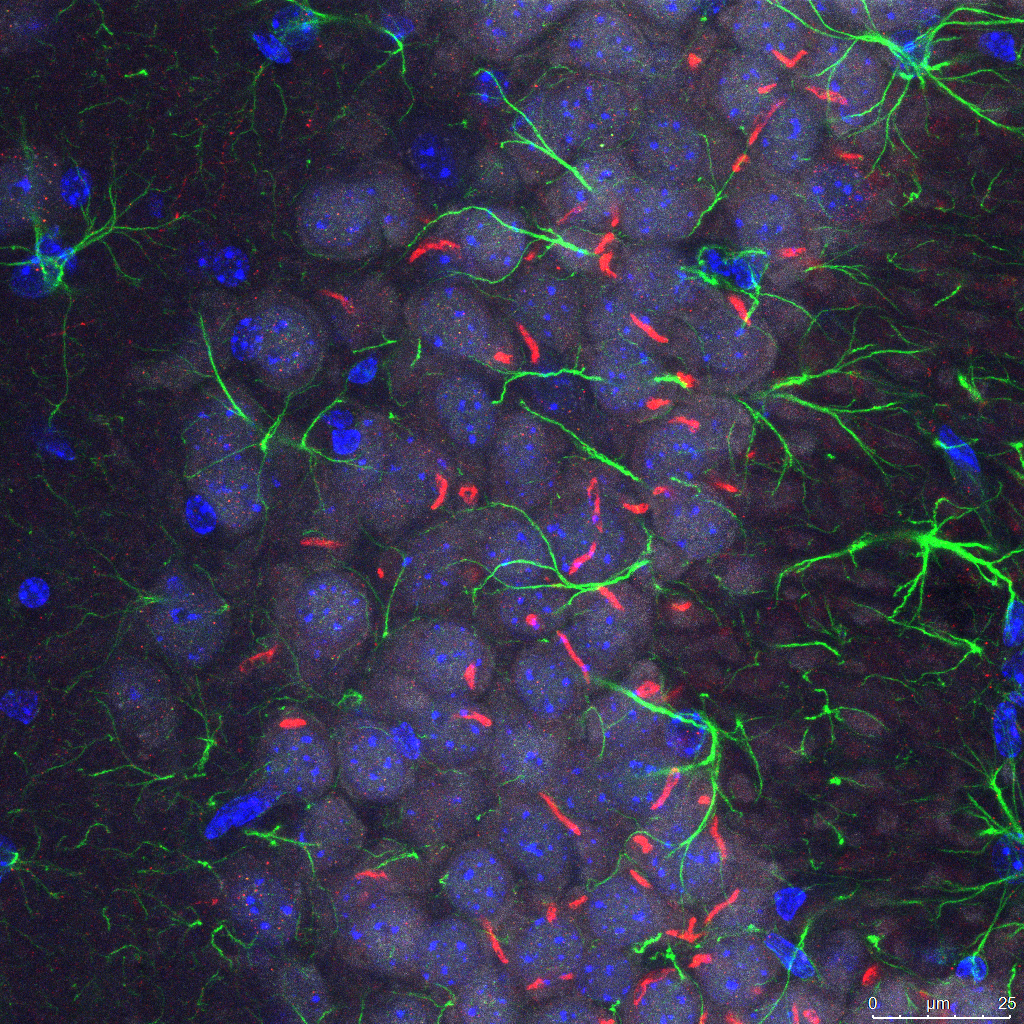

Supplement: Supplementary file 11 — Source data Fig. 5C-E [file 44319_2024_218_MOESM11_ESM.zip › Figure 5 C-D/5D/cdKO 5w/CA3/IMPDH2_RR_Acc_63X_5w_CKO_Rep_IMPDH2_555_NeuN_633_GFAP_488_1.5.20_917_KFC_ant_hip_CA3_Processed001.tif]

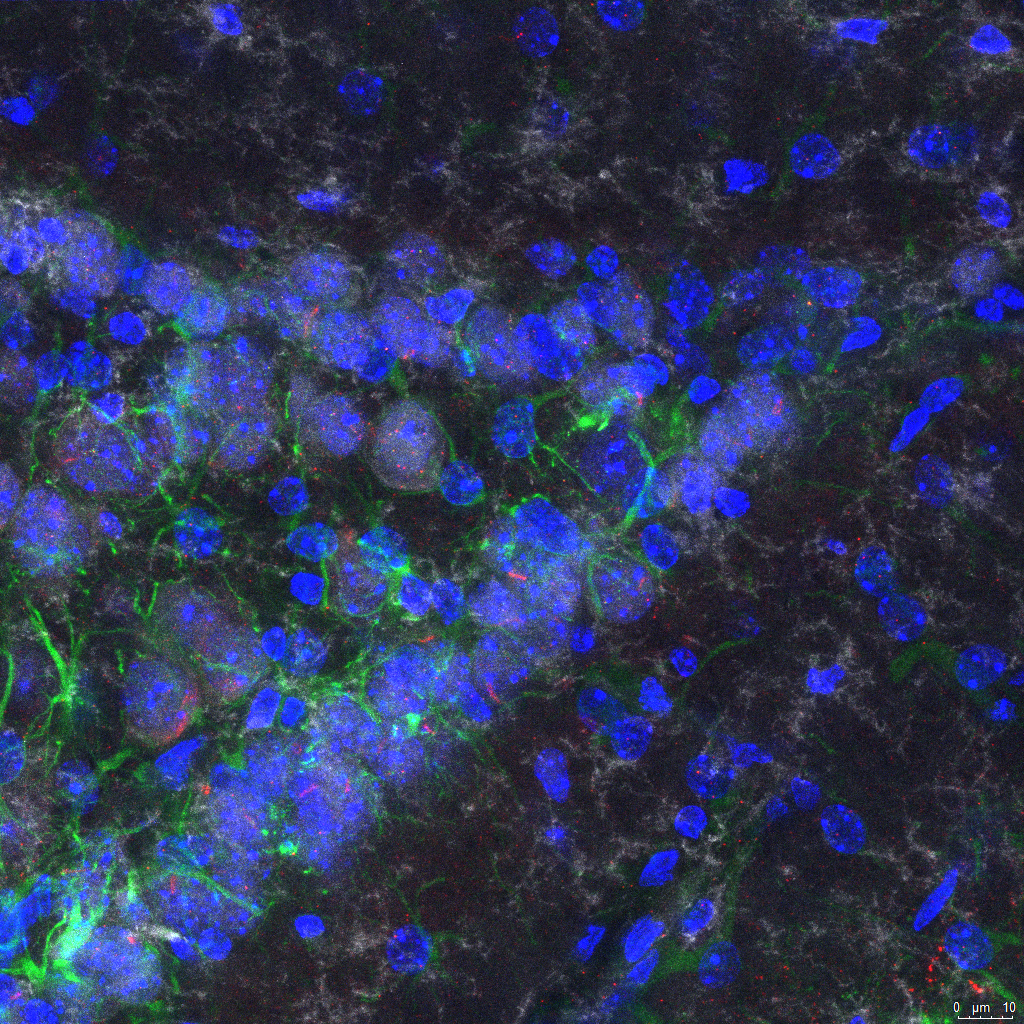

Supplement: Supplementary file 11 — Source data Fig. 5C-E [file 44319_2024_218_MOESM11_ESM.zip › Figure 5 C-D/5D/cdKO 8w/DG/Hip_IMPDH2_RR_Accumulation_CKO_n=1_63X_2mo_12.14.20_880_KFC_ant_hip_DG_crest_rep_Processed001.tif]

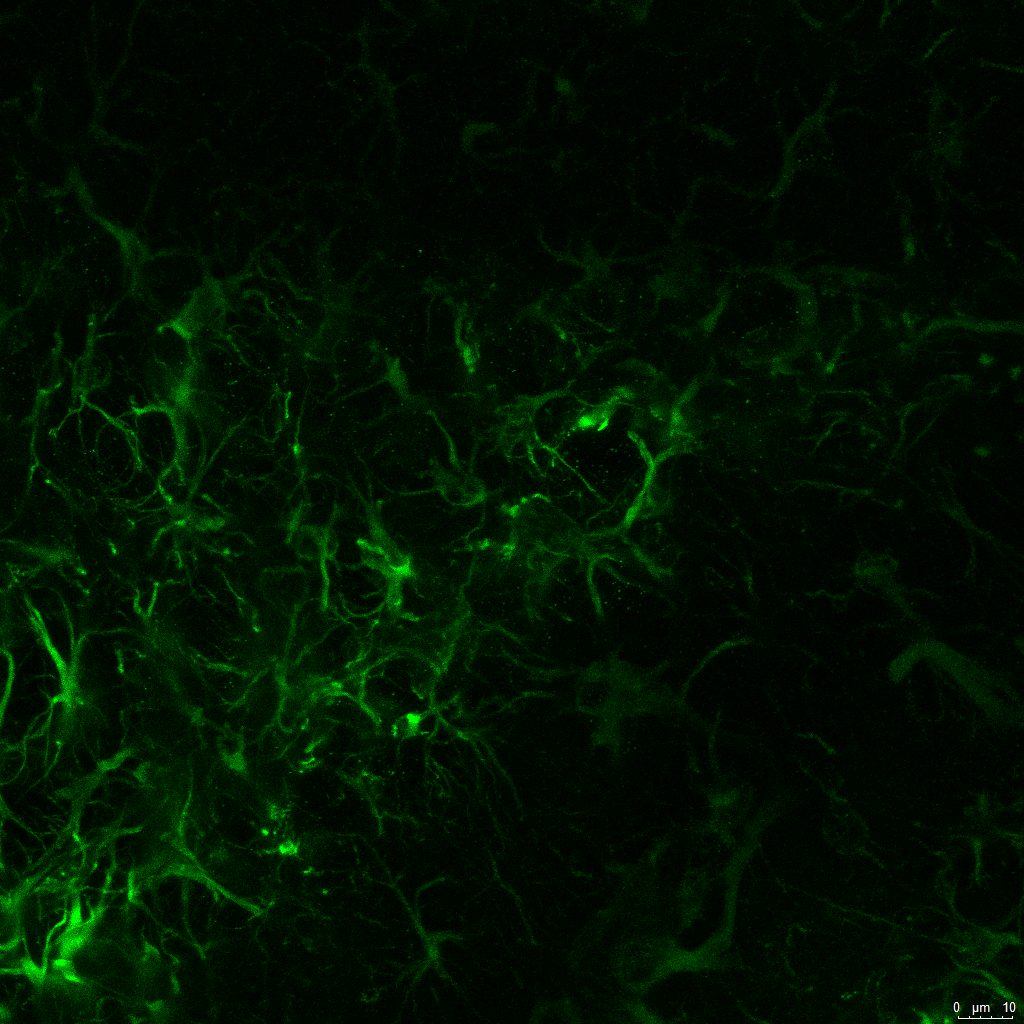

Supplement: Supplementary file 11 — Source data Fig. 5C-E [file 44319_2024_218_MOESM11_ESM.zip › Figure 5 C-D/5D/cdKO 8w/DG/Hip_IMPDH2_RR_Accumulation_CKO_n=1_63X_2mo_12.14.20_880_KFC_ant_hip_DG_crest_rep_Processed001_ch01.tif]

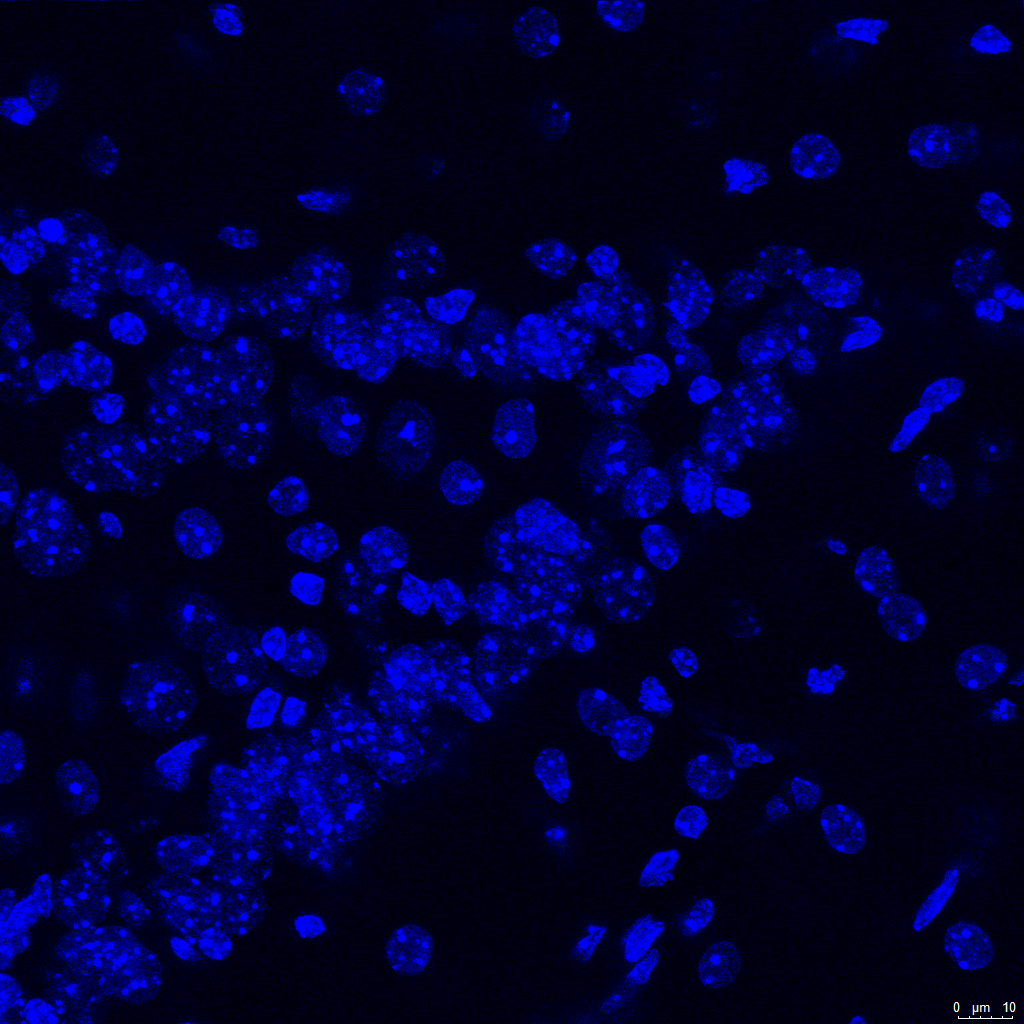

Supplement: Supplementary file 11 — Source data Fig. 5C-E [file 44319_2024_218_MOESM11_ESM.zip › Figure 5 C-D/5D/cdKO 8w/DG/Hip_IMPDH2_RR_Accumulation_CKO_n=1_63X_2mo_12.14.20_880_KFC_ant_hip_DG_crest_rep_Processed001_ch00.tif]

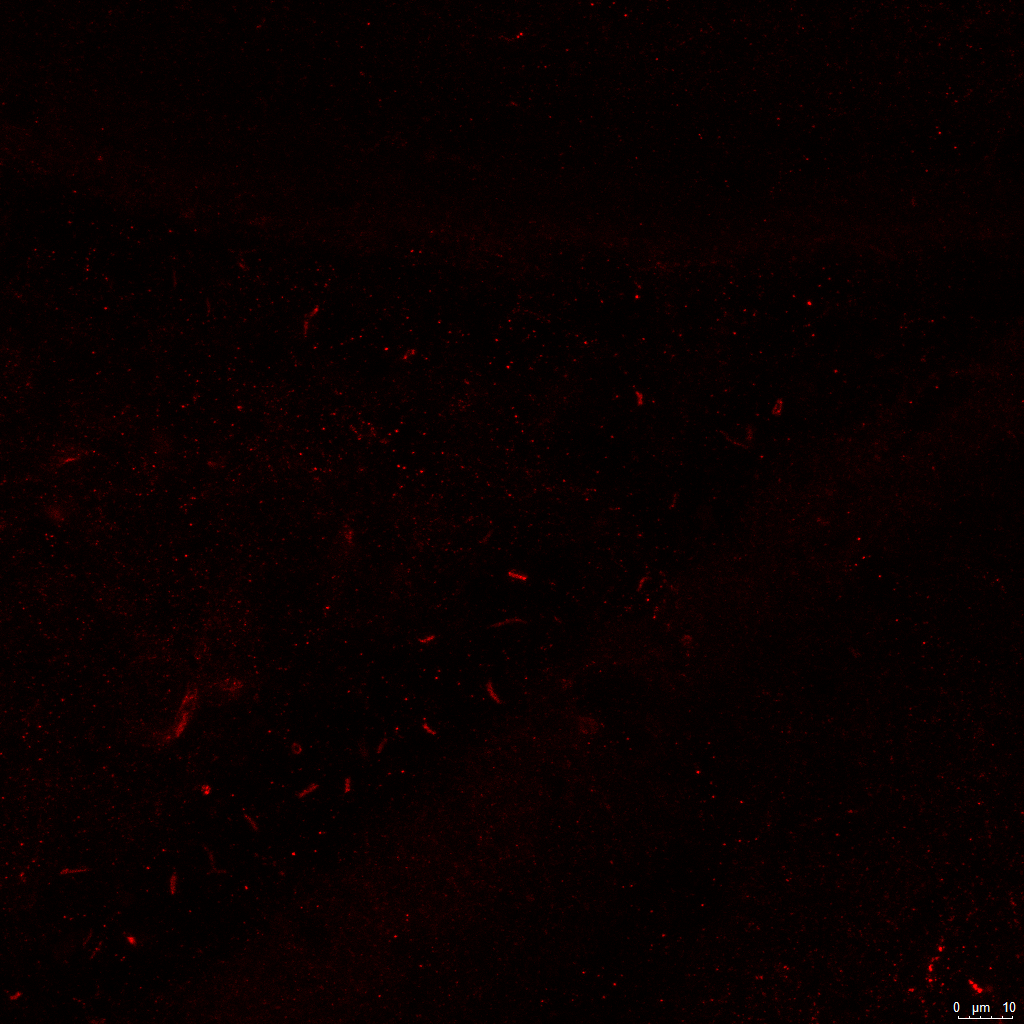

Supplement: Supplementary file 11 — Source data Fig. 5C-E [file 44319_2024_218_MOESM11_ESM.zip › Figure 5 C-D/5D/cdKO 8w/DG/Hip_IMPDH2_RR_Accumulation_CKO_n=1_63X_2mo_12.14.20_880_KFC_ant_hip_DG_crest_rep_Processed001_ch02.tif]

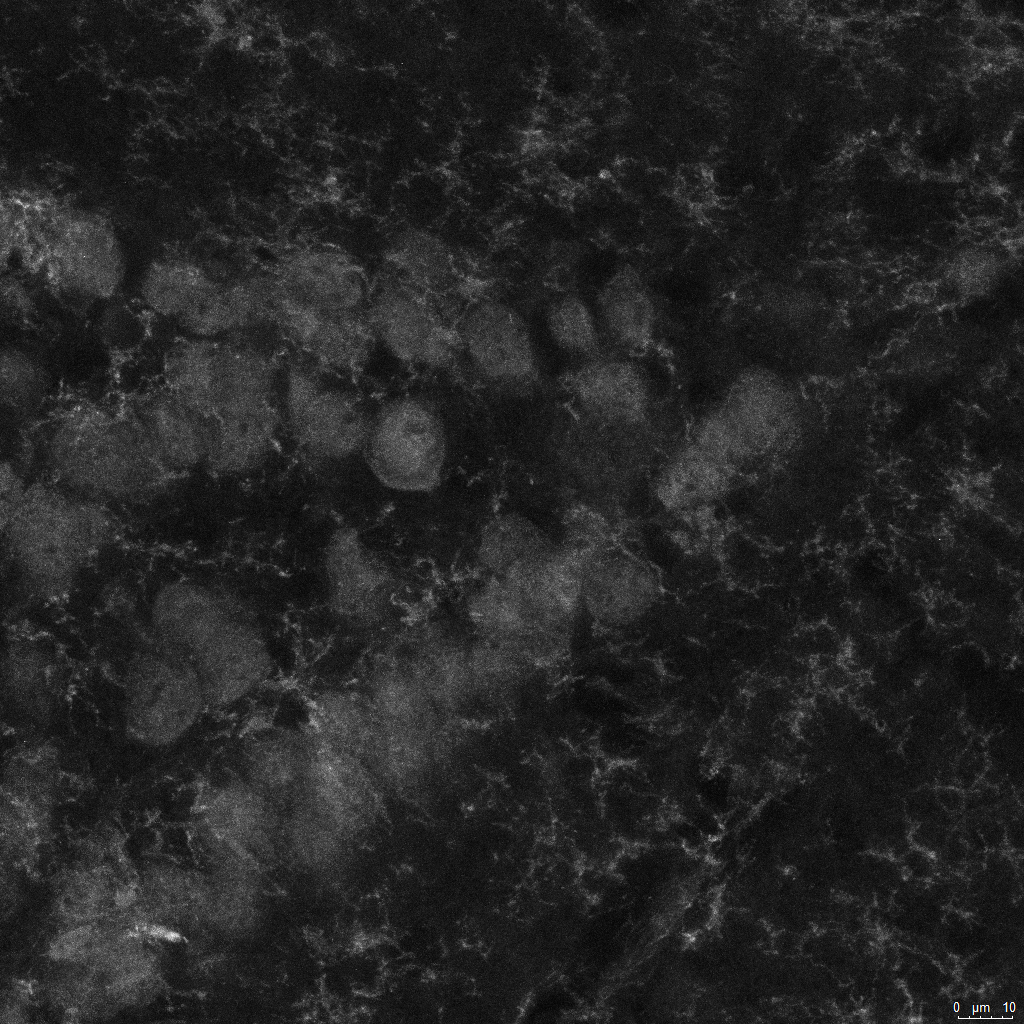

Supplement: Supplementary file 11 — Source data Fig. 5C-E [file 44319_2024_218_MOESM11_ESM.zip › Figure 5 C-D/5D/cdKO 8w/DG/Hip_IMPDH2_RR_Accumulation_CKO_n=1_63X_2mo_12.14.20_880_KFC_ant_hip_DG_crest_rep_Processed001_ch03.tif]

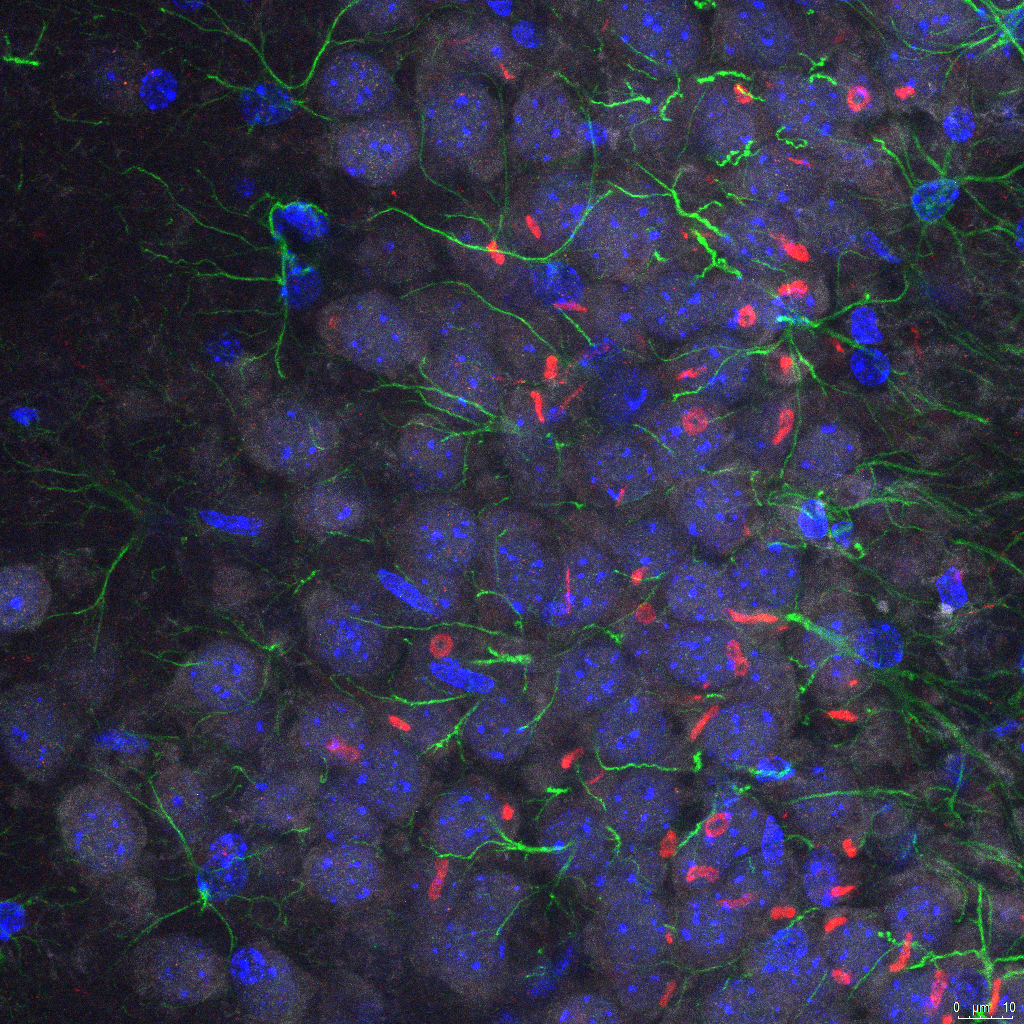

Supplement: Supplementary file 11 — Source data Fig. 5C-E [file 44319_2024_218_MOESM11_ESM.zip › Figure 5 C-D/5D/cdKO 8w/CA3/Hip_IMPDH2_RR_Accumulation_CKO_n=1_63X_2mo_12.14.20_880_KFC_ant_hip_CA3_rep_Processed001.tif]

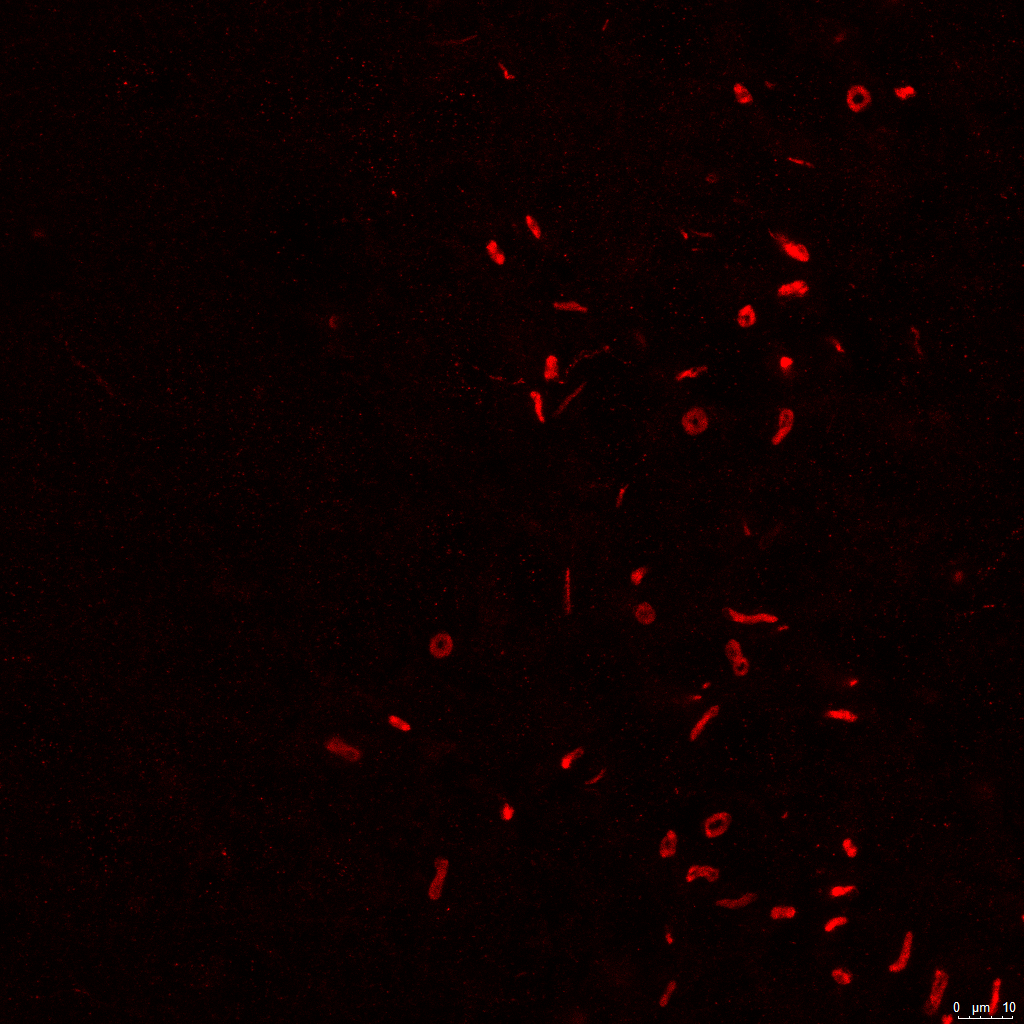

Supplement: Supplementary file 11 — Source data Fig. 5C-E [file 44319_2024_218_MOESM11_ESM.zip › Figure 5 C-D/5D/cdKO 8w/CA3/Hip_IMPDH2_RR_Accumulation_CKO_n=1_63X_2mo_12.14.20_880_KFC_ant_hip_CA3_rep_Processed001_ch02.tif]

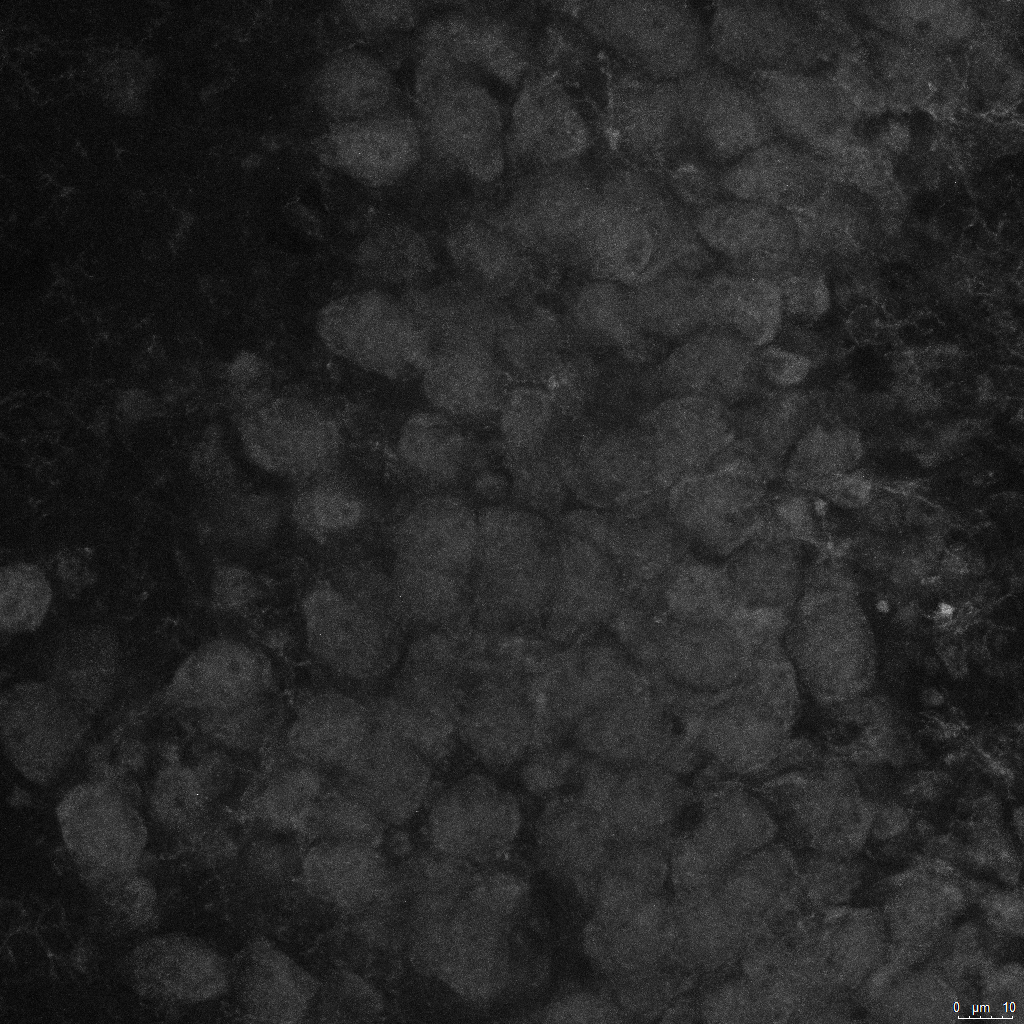

Supplement: Supplementary file 11 — Source data Fig. 5C-E [file 44319_2024_218_MOESM11_ESM.zip › Figure 5 C-D/5D/cdKO 8w/CA3/Hip_IMPDH2_RR_Accumulation_CKO_n=1_63X_2mo_12.14.20_880_KFC_ant_hip_CA3_rep_Processed001_ch03.tif]

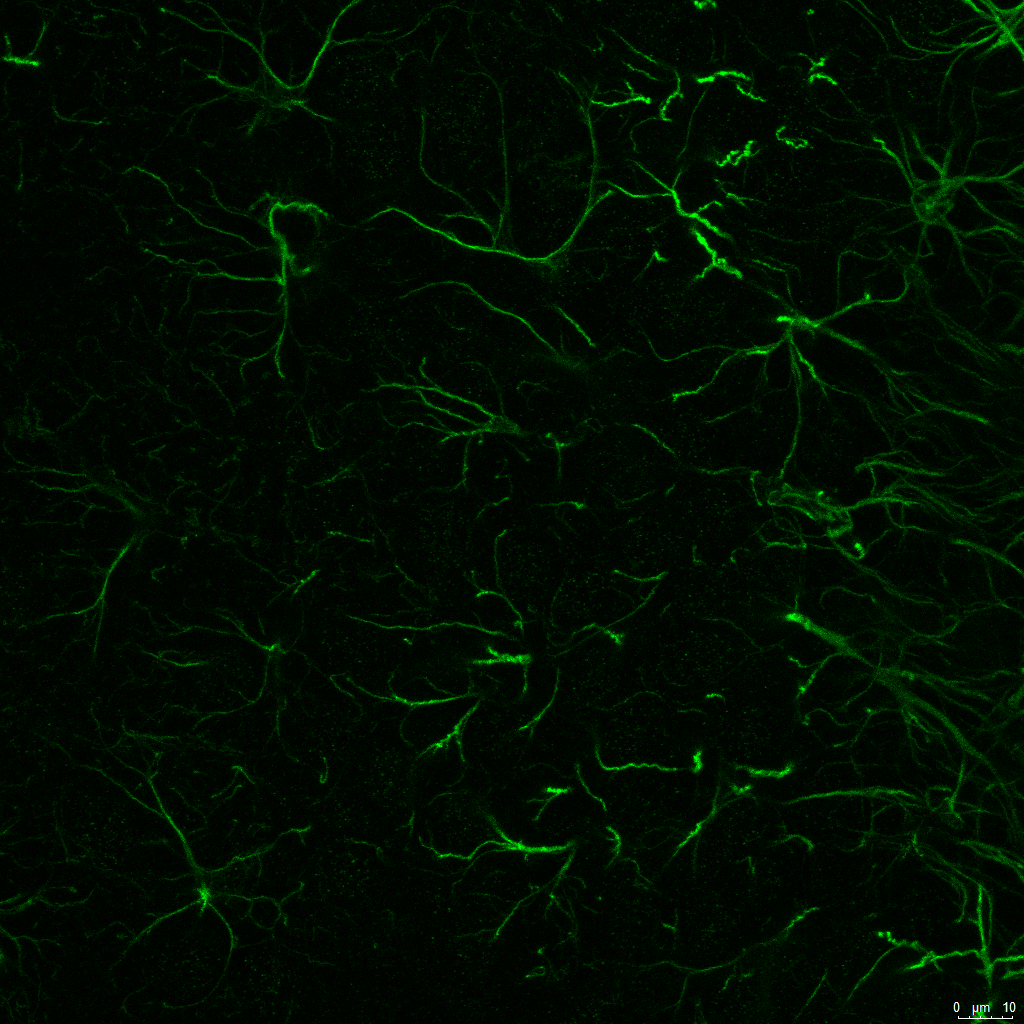

Supplement: Supplementary file 11 — Source data Fig. 5C-E [file 44319_2024_218_MOESM11_ESM.zip › Figure 5 C-D/5D/cdKO 8w/CA3/Hip_IMPDH2_RR_Accumulation_CKO_n=1_63X_2mo_12.14.20_880_KFC_ant_hip_CA3_rep_Processed001_ch01.tif]

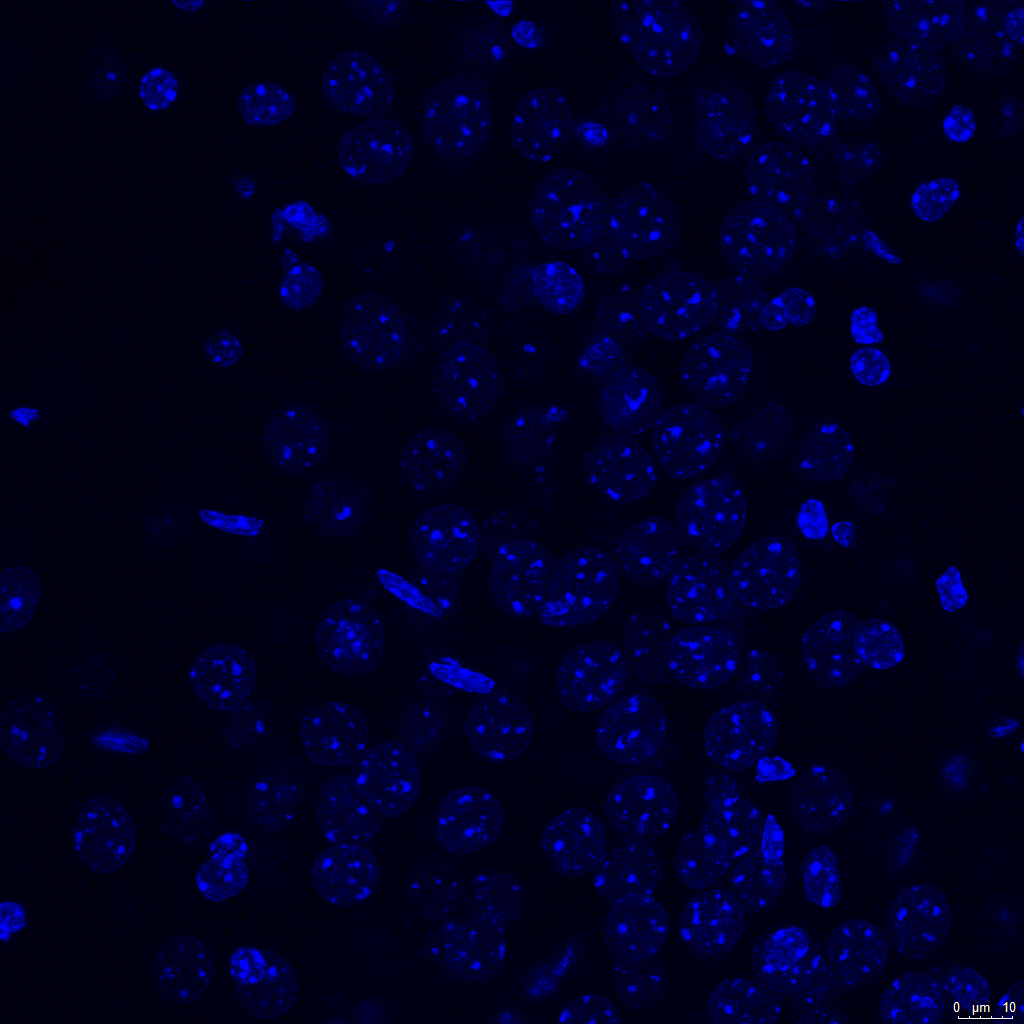

Supplement: Supplementary file 11 — Source data Fig. 5C-E [file 44319_2024_218_MOESM11_ESM.zip › Figure 5 C-D/5D/cdKO 8w/CA3/Hip_IMPDH2_RR_Accumulation_CKO_n=1_63X_2mo_12.14.20_880_KFC_ant_hip_CA3_rep_Processed001_ch00.tif]

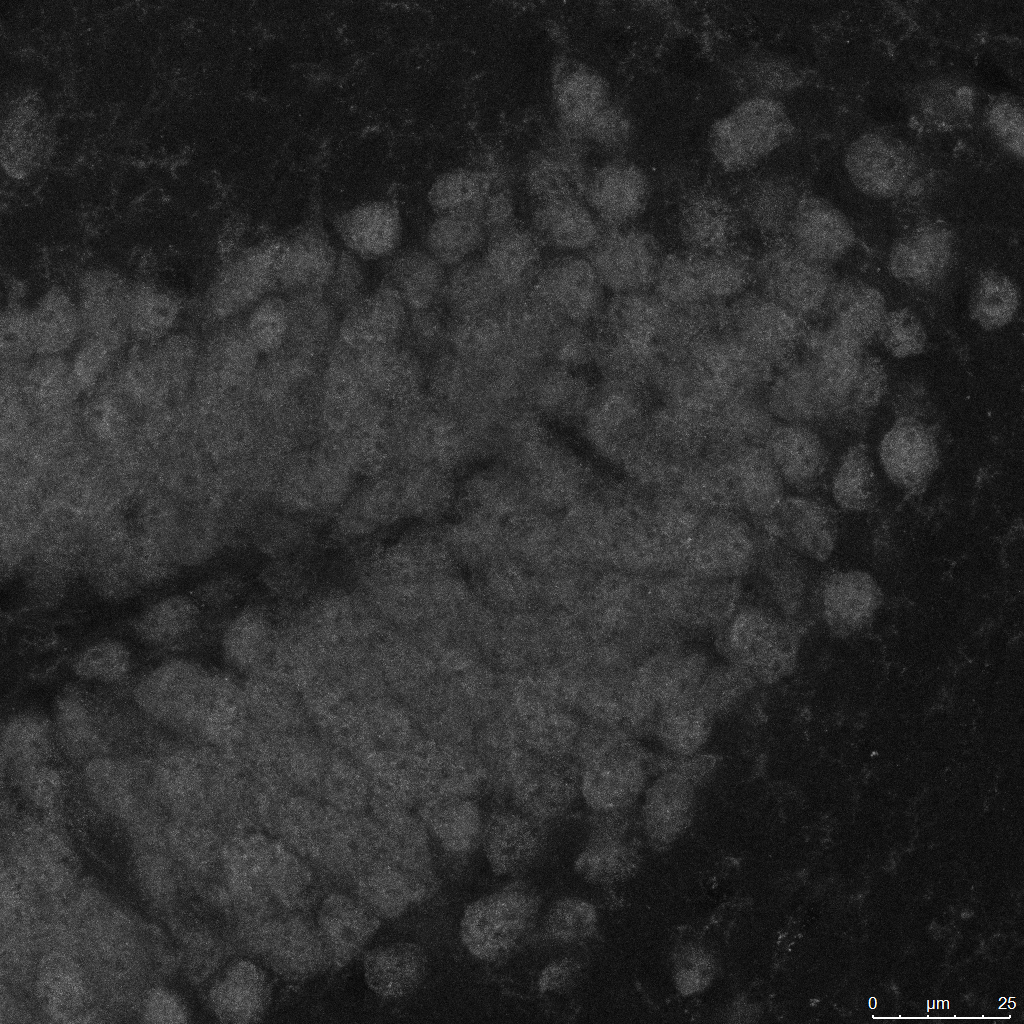

Supplement: Supplementary file 11 — Source data Fig. 5C-E [file 44319_2024_218_MOESM11_ESM.zip › Figure 5 C-D/5D/Ctrl 5w/DG/IMPDH2_RR_Acc_63X_5w_Control_Rep_IMPDH2_555_NeuN_633_GFAP_488_1.5.20_915_Cont_ant_hip_DG_Crest_Processed001_ch03.tif]

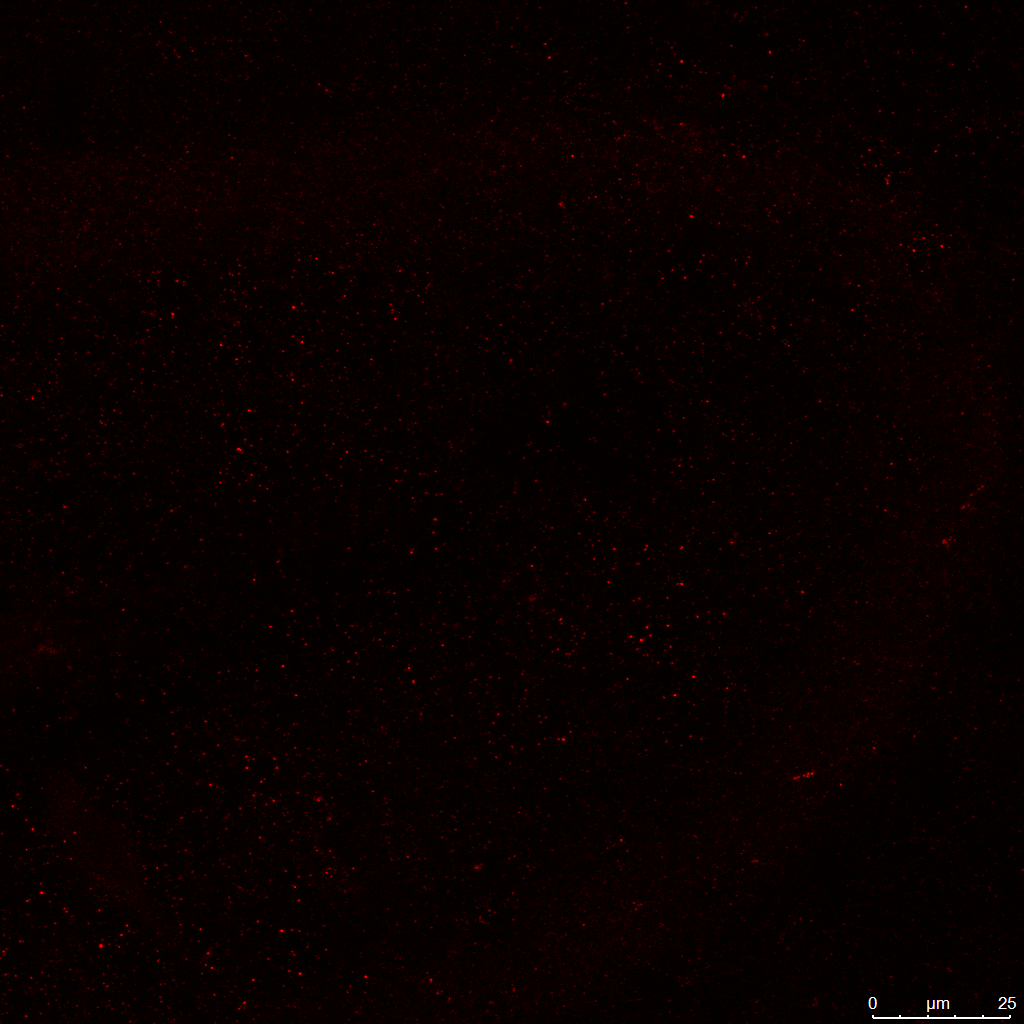

Supplement: Supplementary file 11 — Source data Fig. 5C-E [file 44319_2024_218_MOESM11_ESM.zip › Figure 5 C-D/5D/Ctrl 5w/DG/IMPDH2_RR_Acc_63X_5w_Control_Rep_IMPDH2_555_NeuN_633_GFAP_488_1.5.20_915_Cont_ant_hip_DG_Crest_Processed001_ch02.tif]

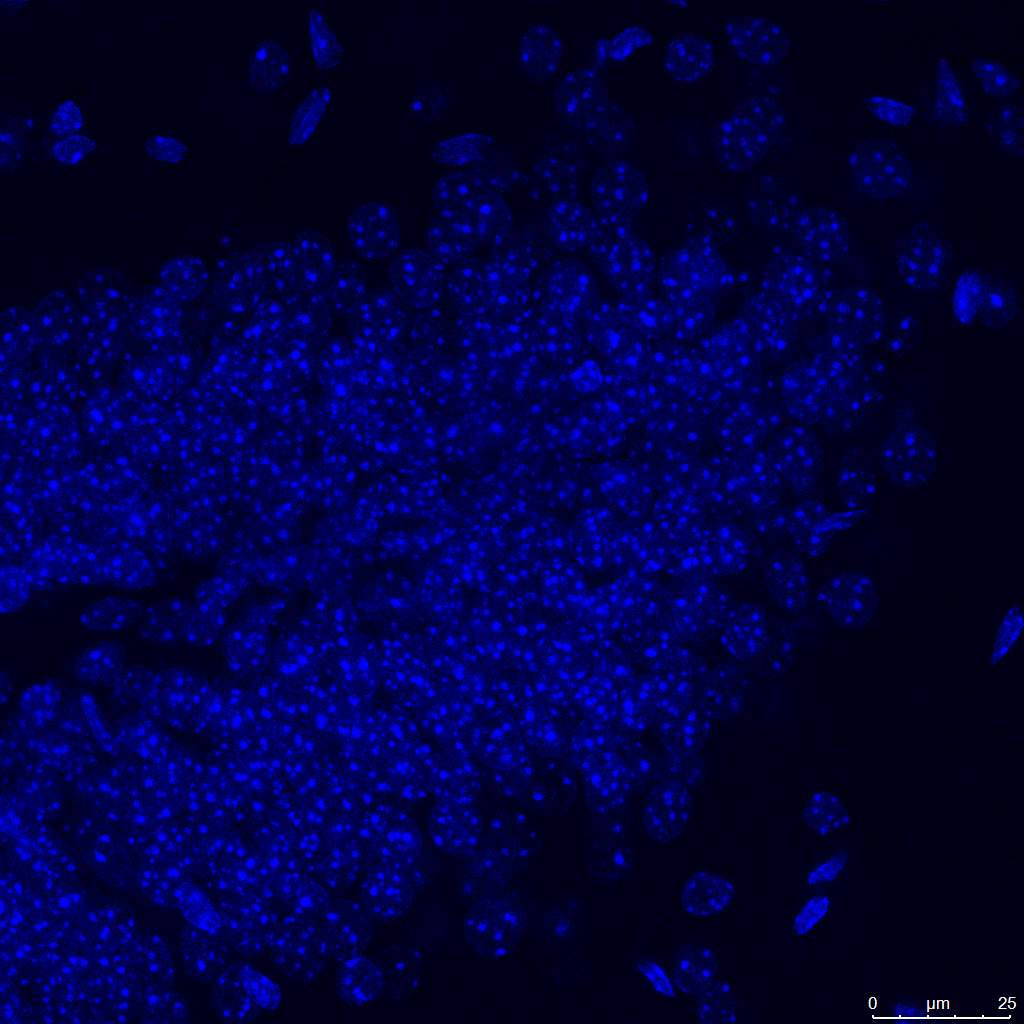

Supplement: Supplementary file 11 — Source data Fig. 5C-E [file 44319_2024_218_MOESM11_ESM.zip › Figure 5 C-D/5D/Ctrl 5w/DG/IMPDH2_RR_Acc_63X_5w_Control_Rep_IMPDH2_555_NeuN_633_GFAP_488_1.5.20_915_Cont_ant_hip_DG_Crest_Processed001_ch00.tif]

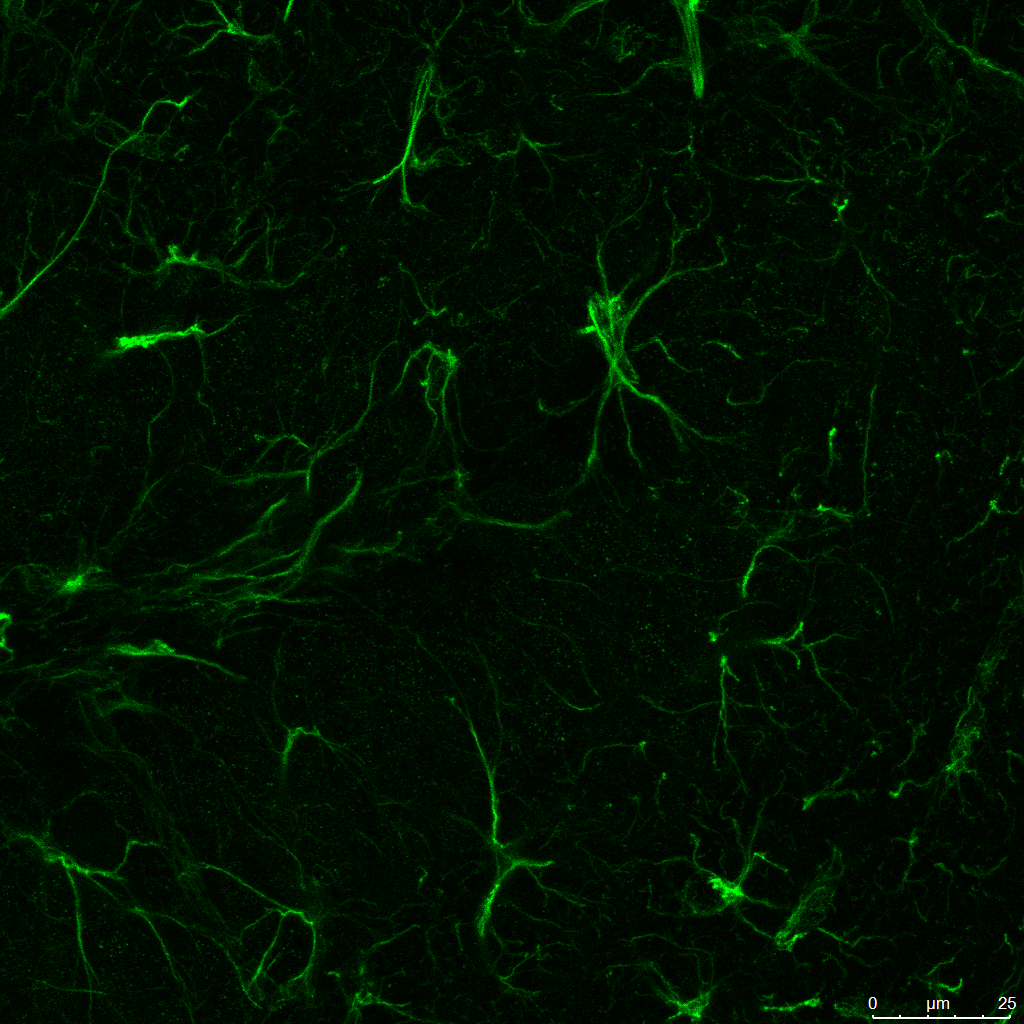

Supplement: Supplementary file 11 — Source data Fig. 5C-E [file 44319_2024_218_MOESM11_ESM.zip › Figure 5 C-D/5D/Ctrl 5w/DG/IMPDH2_RR_Acc_63X_5w_Control_Rep_IMPDH2_555_NeuN_633_GFAP_488_1.5.20_915_Cont_ant_hip_DG_Crest_Processed001_ch01.tif]

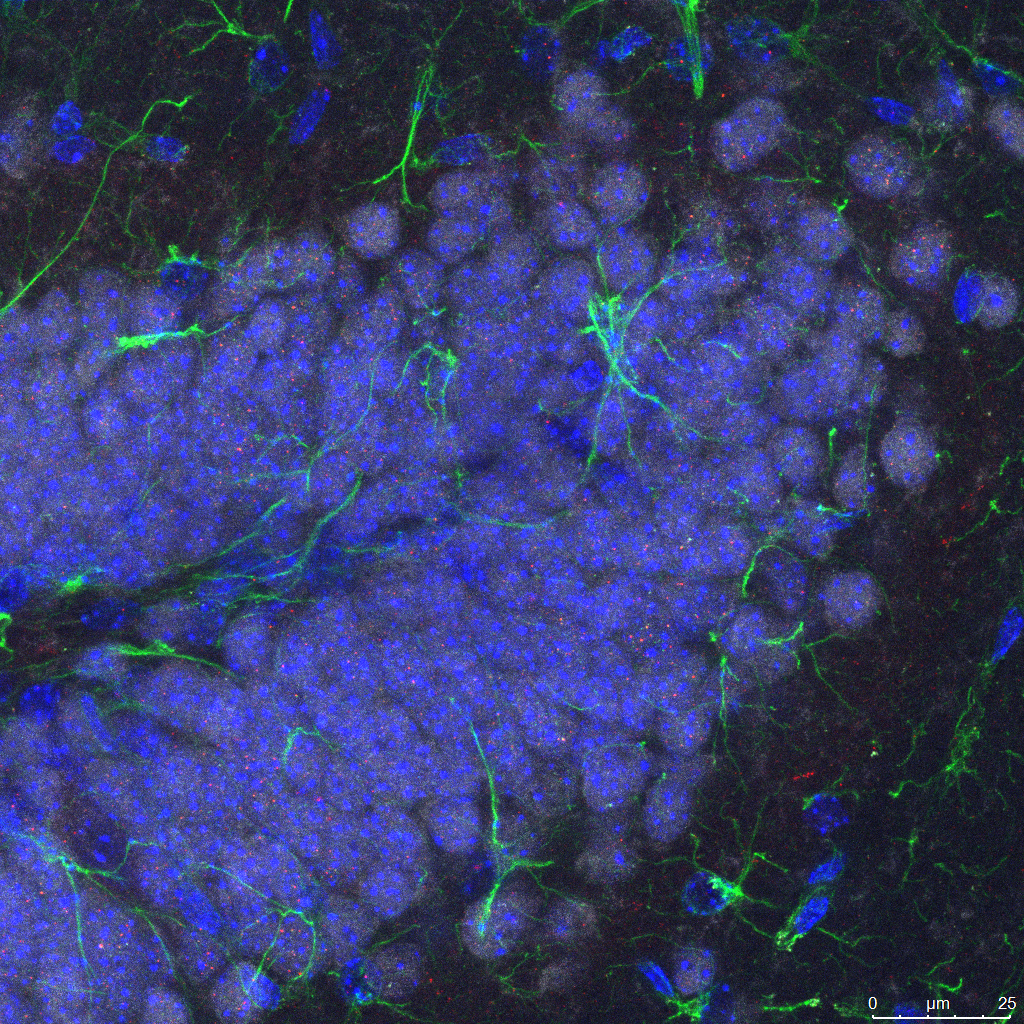

Supplement: Supplementary file 11 — Source data Fig. 5C-E [file 44319_2024_218_MOESM11_ESM.zip › Figure 5 C-D/5D/Ctrl 5w/DG/IMPDH2_RR_Acc_63X_5w_Control_Rep_IMPDH2_555_NeuN_633_GFAP_488_1.5.20_915_Cont_ant_hip_DG_Crest_Processed001.tif]

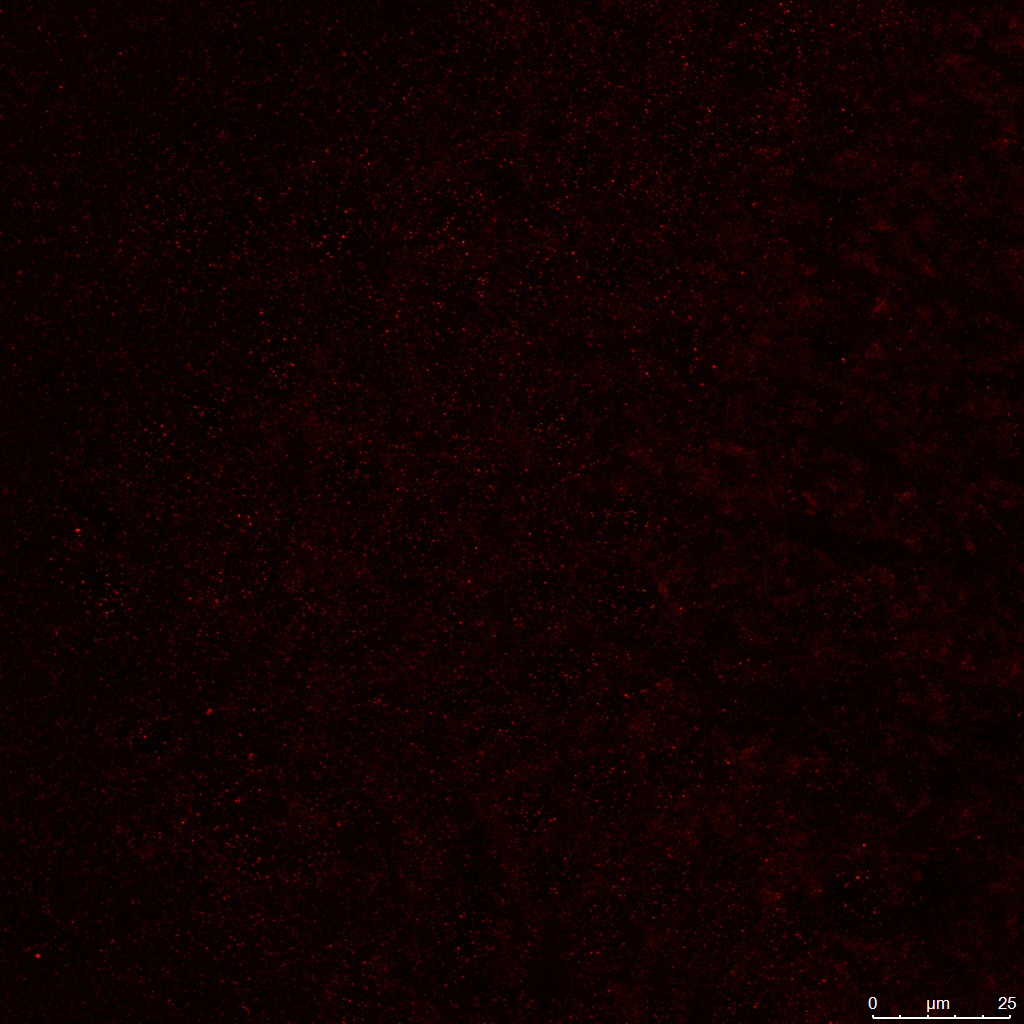

Supplement: Supplementary file 11 — Source data Fig. 5C-E [file 44319_2024_218_MOESM11_ESM.zip › Figure 5 C-D/5D/Ctrl 5w/CA3/IMPDH2_RR_Acc_63X_5w_Control_Rep_IMPDH2_555_NeuN_633_GFAP_488_1.5.20_915_Cont_ant_hip_CA3_Processed001_ch02.tif]

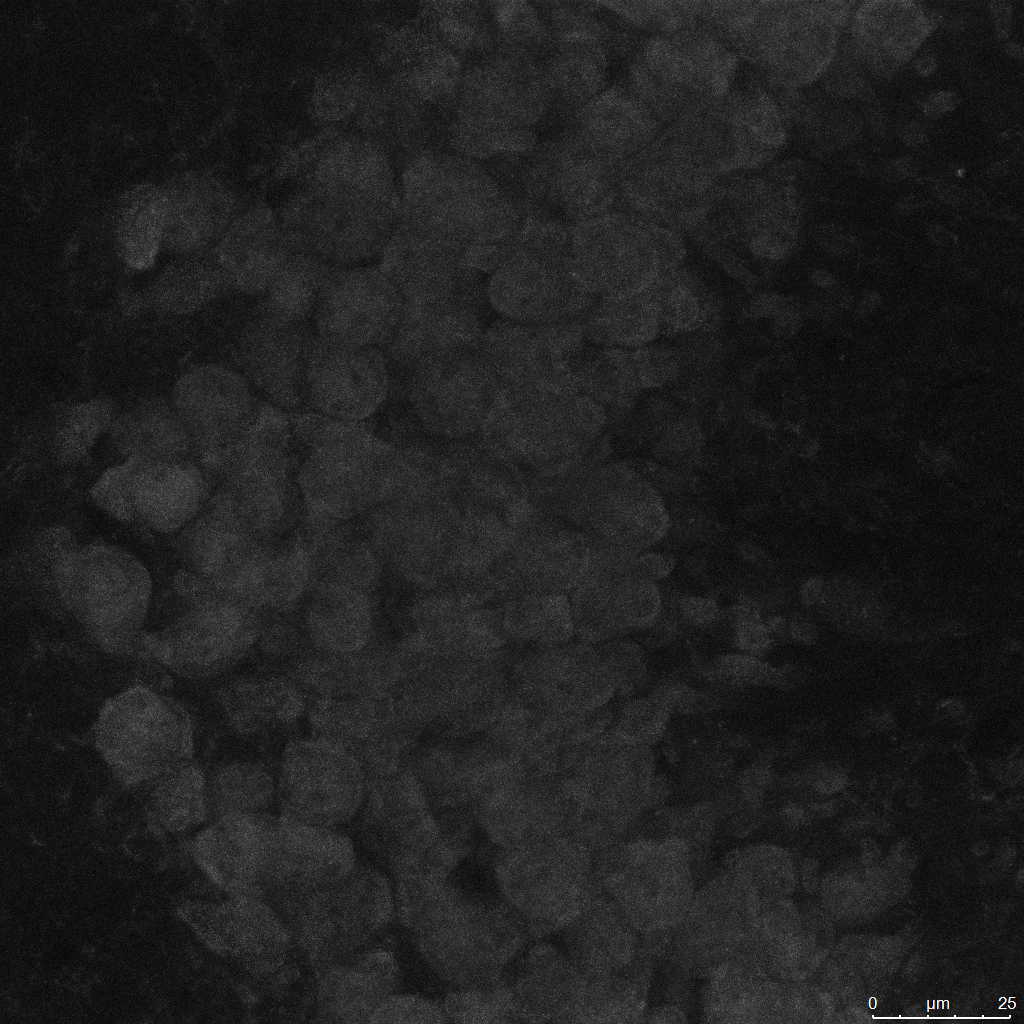

Supplement: Supplementary file 11 — Source data Fig. 5C-E [file 44319_2024_218_MOESM11_ESM.zip › Figure 5 C-D/5D/Ctrl 5w/CA3/IMPDH2_RR_Acc_63X_5w_Control_Rep_IMPDH2_555_NeuN_633_GFAP_488_1.5.20_915_Cont_ant_hip_CA3_Processed001_ch03.tif]

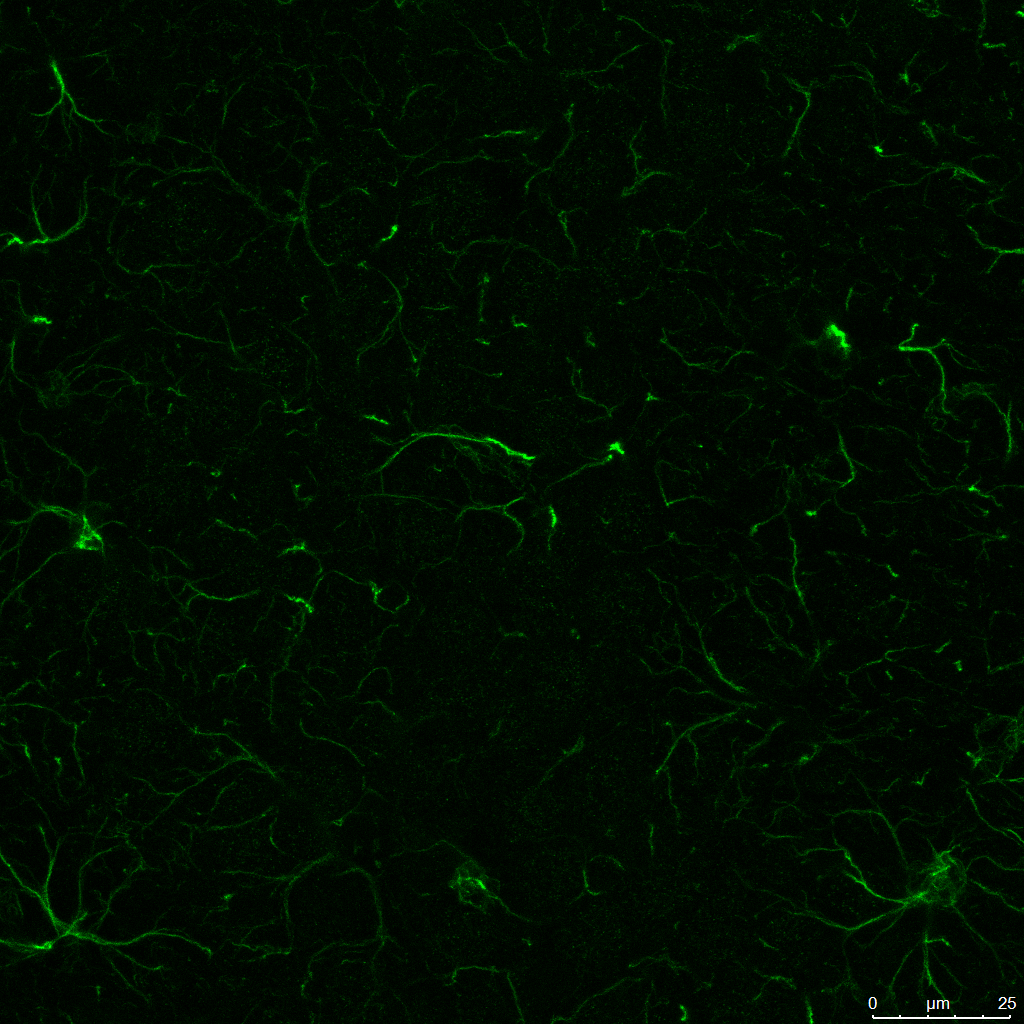

Supplement: Supplementary file 11 — Source data Fig. 5C-E [file 44319_2024_218_MOESM11_ESM.zip › Figure 5 C-D/5D/Ctrl 5w/CA3/IMPDH2_RR_Acc_63X_5w_Control_Rep_IMPDH2_555_NeuN_633_GFAP_488_1.5.20_915_Cont_ant_hip_CA3_Processed001_ch01.tif]

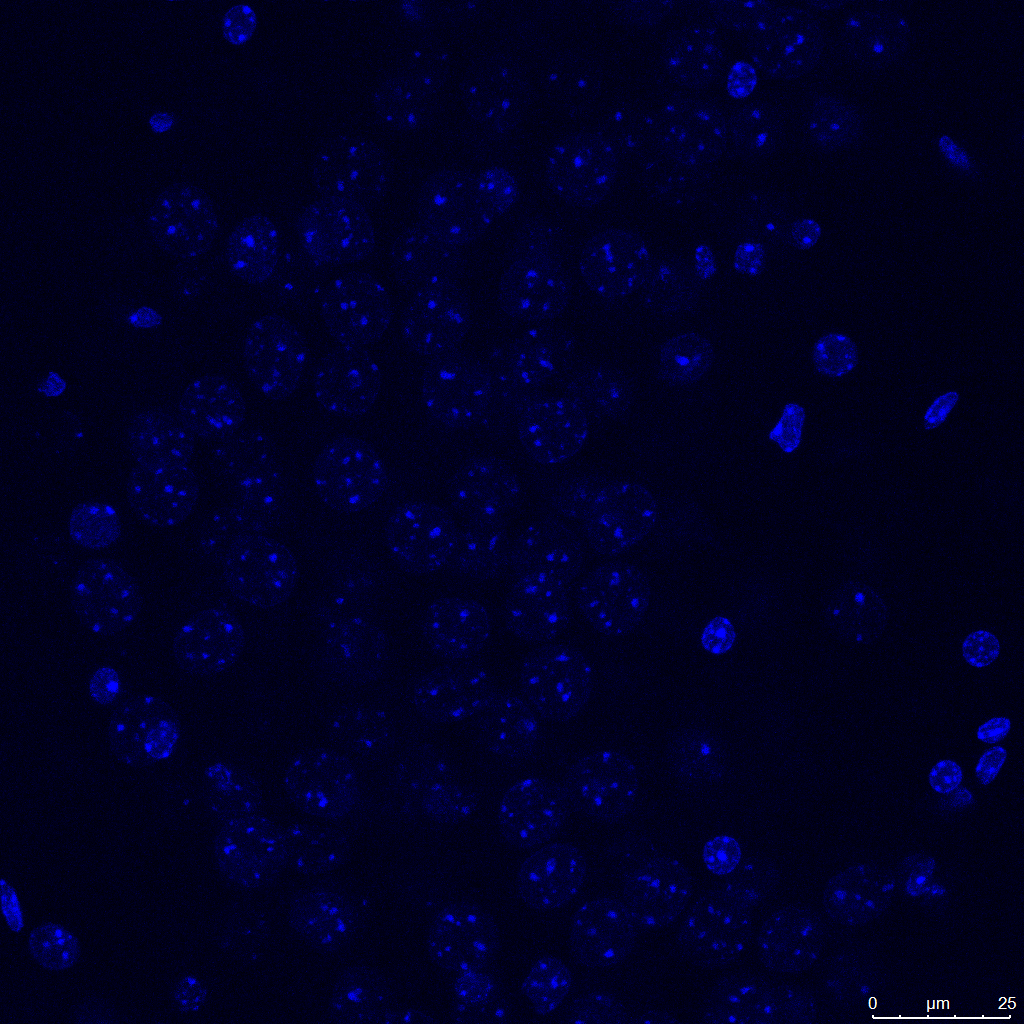

Supplement: Supplementary file 11 — Source data Fig. 5C-E [file 44319_2024_218_MOESM11_ESM.zip › Figure 5 C-D/5D/Ctrl 5w/CA3/IMPDH2_RR_Acc_63X_5w_Control_Rep_IMPDH2_555_NeuN_633_GFAP_488_1.5.20_915_Cont_ant_hip_CA3_Processed001_ch00.tif]

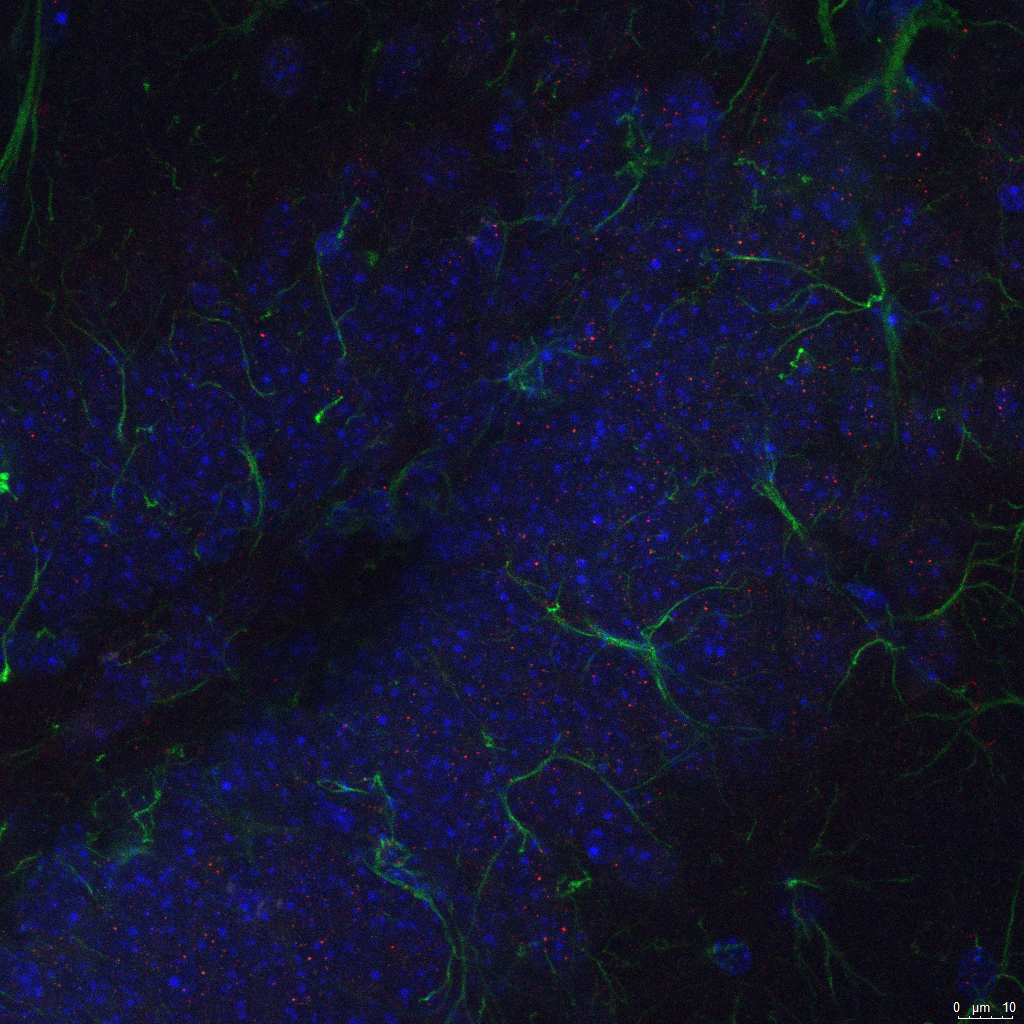

Supplement: Supplementary file 11 — Source data Fig. 5C-E [file 44319_2024_218_MOESM11_ESM.zip › Figure 5 C-D/5D/Ctrl 8w/DG/Hip_IMPDH2_RR_Accumulation_Control_n=1_poor_staining_63X_2mo_12.14.20_885_HF_ant_hip_DG_crest_rep_Processed001.tif]

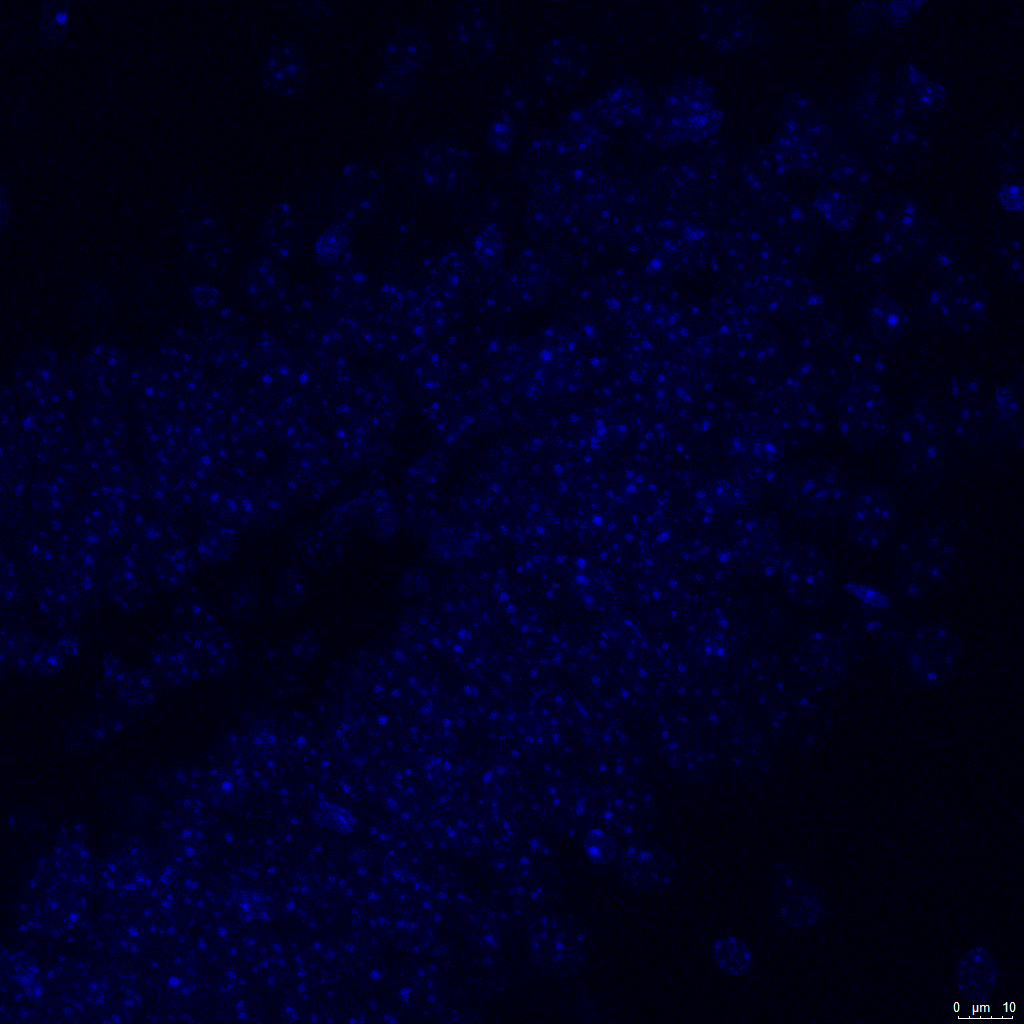

Supplement: Supplementary file 11 — Source data Fig. 5C-E [file 44319_2024_218_MOESM11_ESM.zip › Figure 5 C-D/5D/Ctrl 8w/DG/Hip_IMPDH2_RR_Accumulation_Control_n=1_poor_staining_63X_2mo_12.14.20_885_HF_ant_hip_DG_crest_rep_Processed001_ch00.tif]

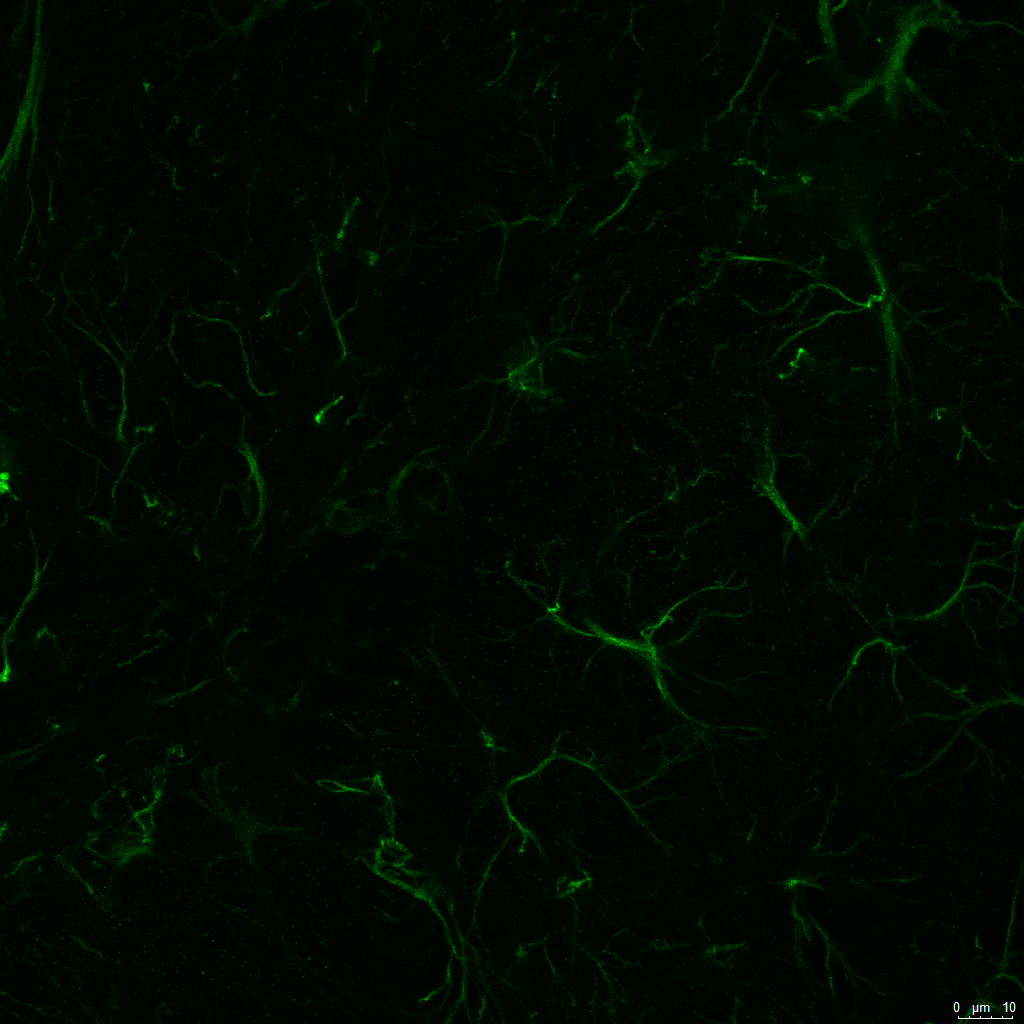

Supplement: Supplementary file 11 — Source data Fig. 5C-E [file 44319_2024_218_MOESM11_ESM.zip › Figure 5 C-D/5D/Ctrl 8w/DG/Hip_IMPDH2_RR_Accumulation_Control_n=1_poor_staining_63X_2mo_12.14.20_885_HF_ant_hip_DG_crest_rep_Processed001_ch01.tif]

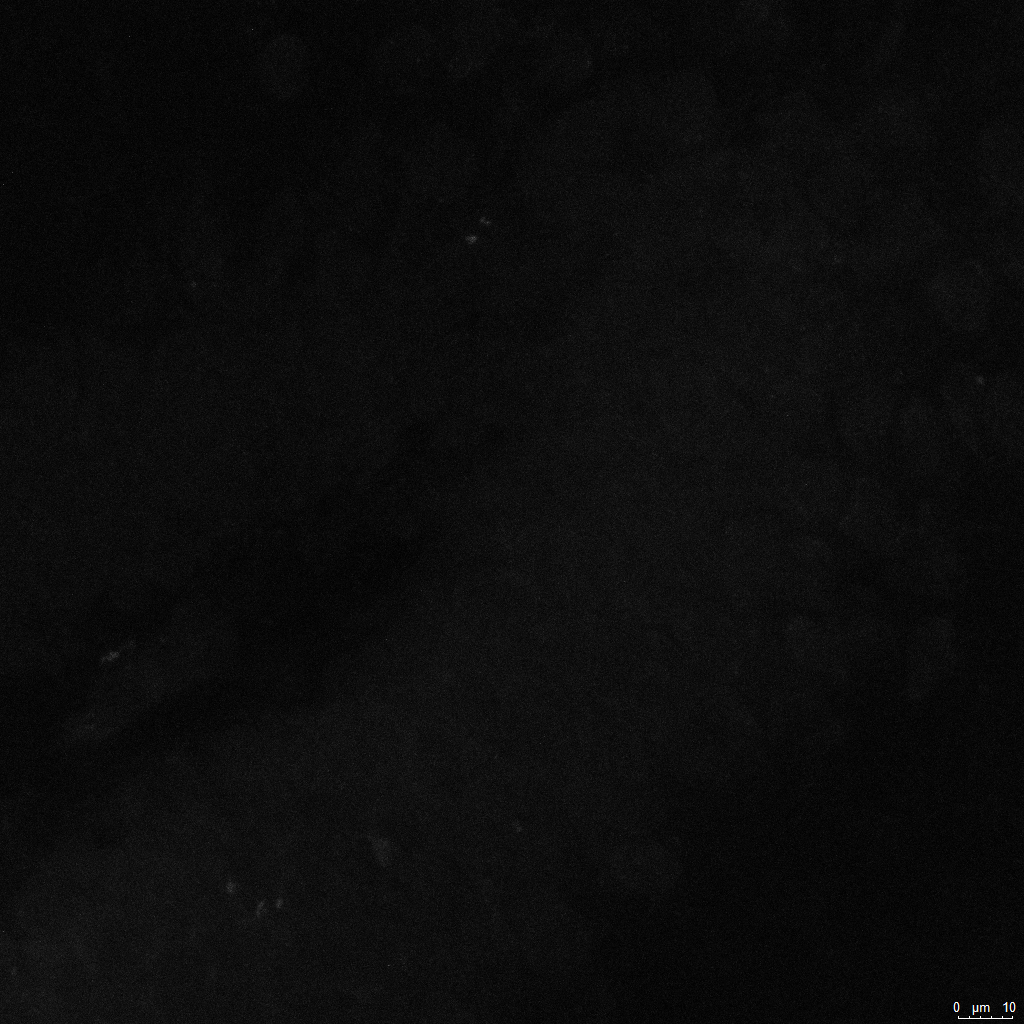

Supplement: Supplementary file 11 — Source data Fig. 5C-E [file 44319_2024_218_MOESM11_ESM.zip › Figure 5 C-D/5D/Ctrl 8w/DG/Hip_IMPDH2_RR_Accumulation_Control_n=1_poor_staining_63X_2mo_12.14.20_885_HF_ant_hip_DG_crest_rep_Processed001_ch03.tif]

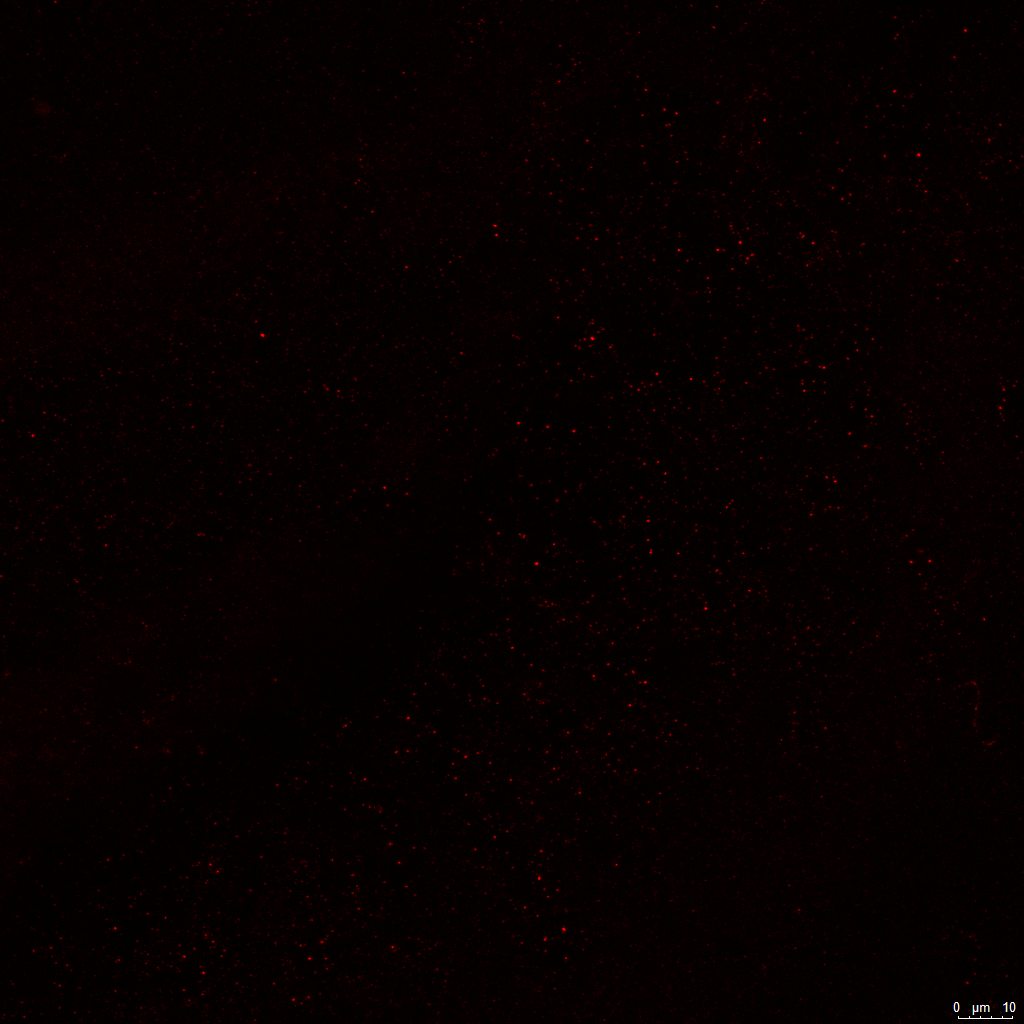

Supplement: Supplementary file 11 — Source data Fig. 5C-E [file 44319_2024_218_MOESM11_ESM.zip › Figure 5 C-D/5D/Ctrl 8w/DG/Hip_IMPDH2_RR_Accumulation_Control_n=1_poor_staining_63X_2mo_12.14.20_885_HF_ant_hip_DG_crest_rep_Processed001_ch02.tif]

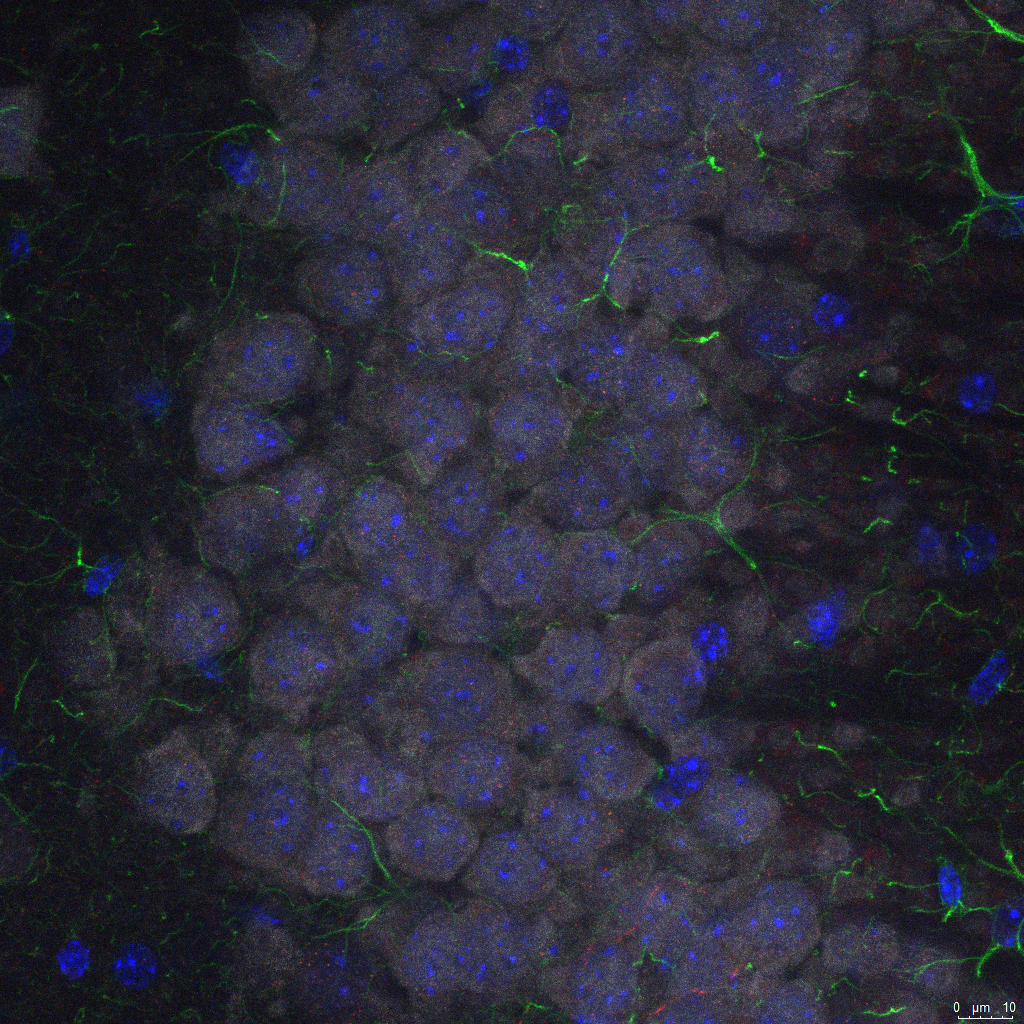

Supplement: Supplementary file 11 — Source data Fig. 5C-E [file 44319_2024_218_MOESM11_ESM.zip › Figure 5 C-D/5D/Ctrl 8w/CA3/Hip_IMPDH2_RR_Accumulation_Control_n=1_poor_staining_63X_2mo_12.14.20_885_HF_ant_hip_CA3_rep_Processed001.tif]

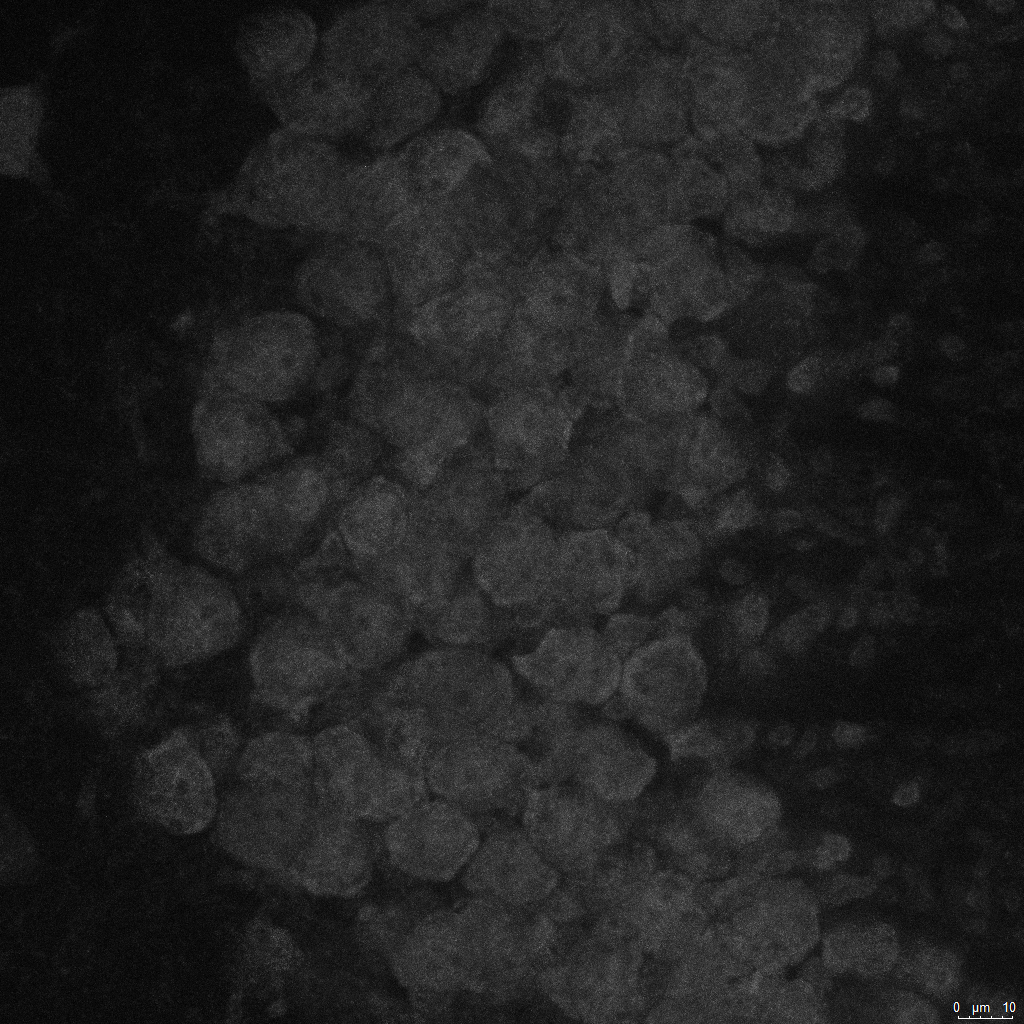

Supplement: Supplementary file 11 — Source data Fig. 5C-E [file 44319_2024_218_MOESM11_ESM.zip › Figure 5 C-D/5D/Ctrl 8w/CA3/Hip_IMPDH2_RR_Accumulation_Control_n=1_poor_staining_63X_2mo_12.14.20_885_HF_ant_hip_CA3_rep_Processed001_ch03.tif]

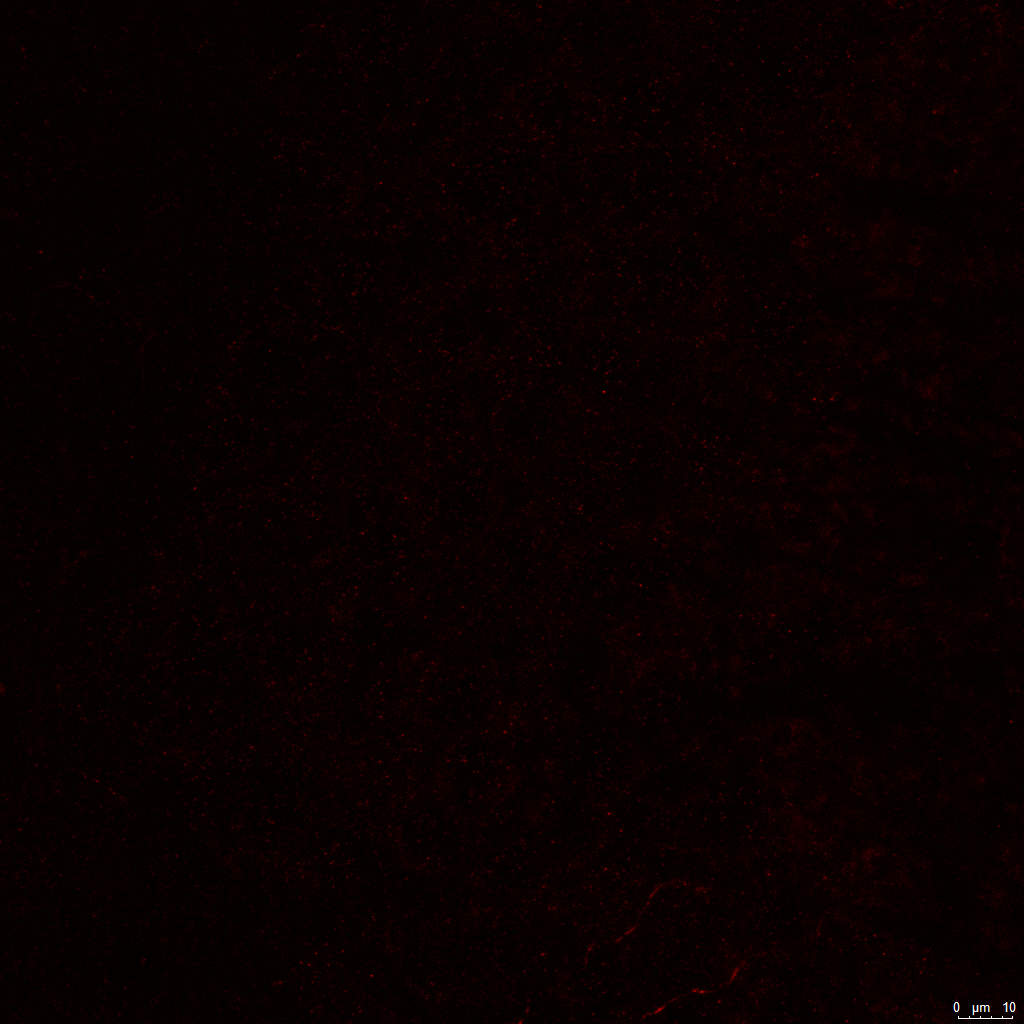

Supplement: Supplementary file 11 — Source data Fig. 5C-E [file 44319_2024_218_MOESM11_ESM.zip › Figure 5 C-D/5D/Ctrl 8w/CA3/Hip_IMPDH2_RR_Accumulation_Control_n=1_poor_staining_63X_2mo_12.14.20_885_HF_ant_hip_CA3_rep_Processed001_ch02.tif]

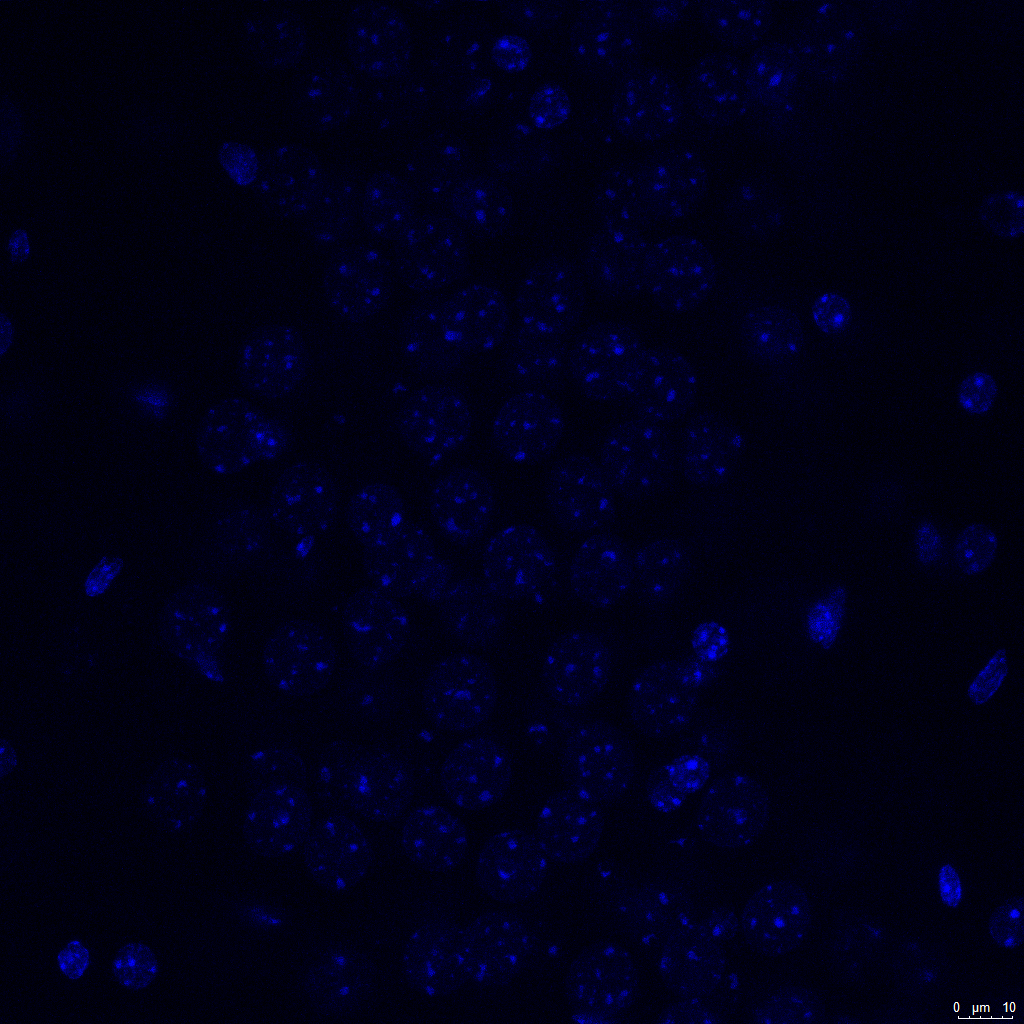

Supplement: Supplementary file 11 — Source data Fig. 5C-E [file 44319_2024_218_MOESM11_ESM.zip › Figure 5 C-D/5D/Ctrl 8w/CA3/Hip_IMPDH2_RR_Accumulation_Control_n=1_poor_staining_63X_2mo_12.14.20_885_HF_ant_hip_CA3_rep_Processed001_ch00.tif]

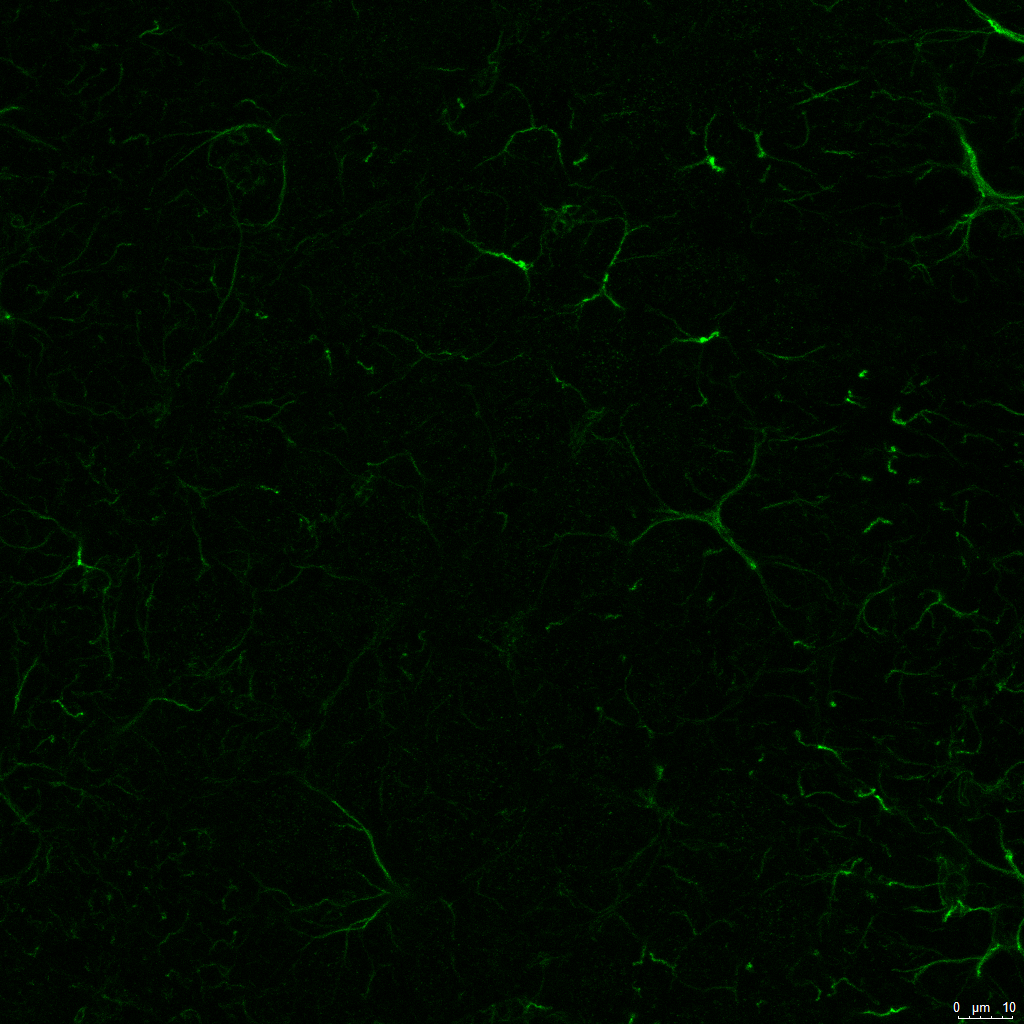

Supplement: Supplementary file 11 — Source data Fig. 5C-E [file 44319_2024_218_MOESM11_ESM.zip › Figure 5 C-D/5D/Ctrl 8w/CA3/Hip_IMPDH2_RR_Accumulation_Control_n=1_poor_staining_63X_2mo_12.14.20_885_HF_ant_hip_CA3_rep_Processed001_ch01.tif]

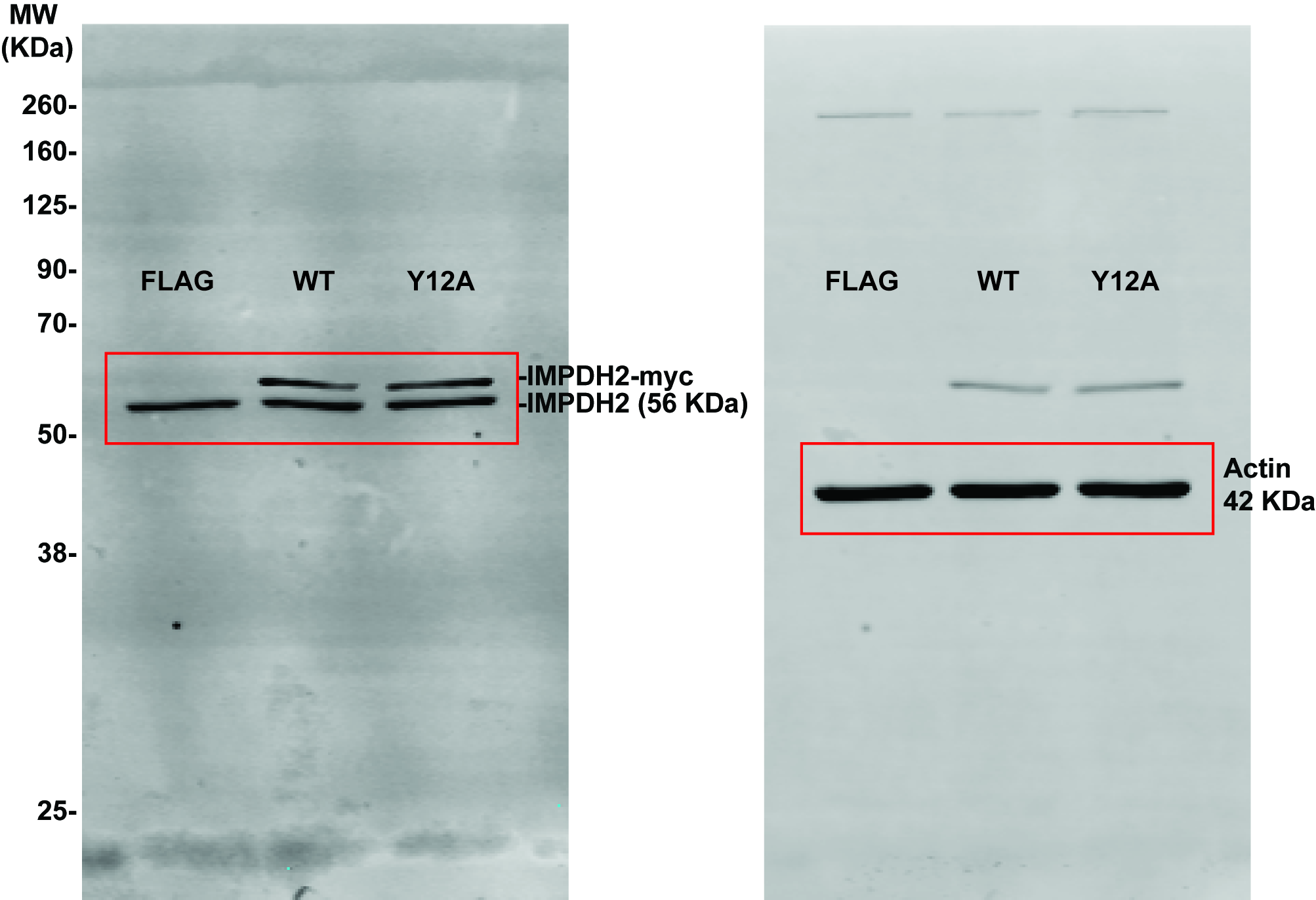

Supplement: Supplementary file 12 — Source data Fig. 6 [file 44319_2024_218_MOESM12_ESM.zip › Figure 6/6B/WB 6B.tif]

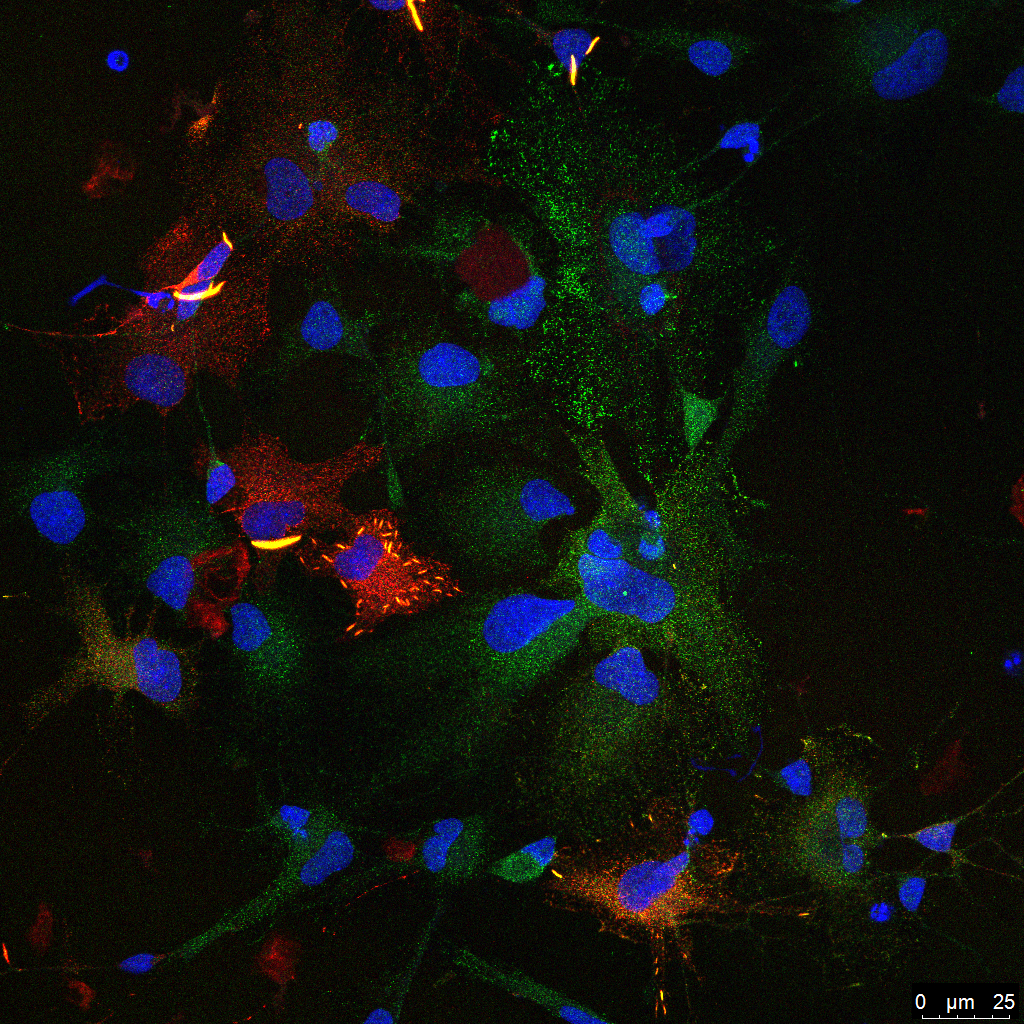

Supplement: Supplementary file 12 — Source data Fig. 6 [file 44319_2024_218_MOESM12_ESM.zip › Figure 6/6C/WT, Transduced_41 NPC, WT507, Dox 50 ng-ml 24h, -ADO, IMPDH2-488, cMyc-555 40x, Z-Stack.tif]

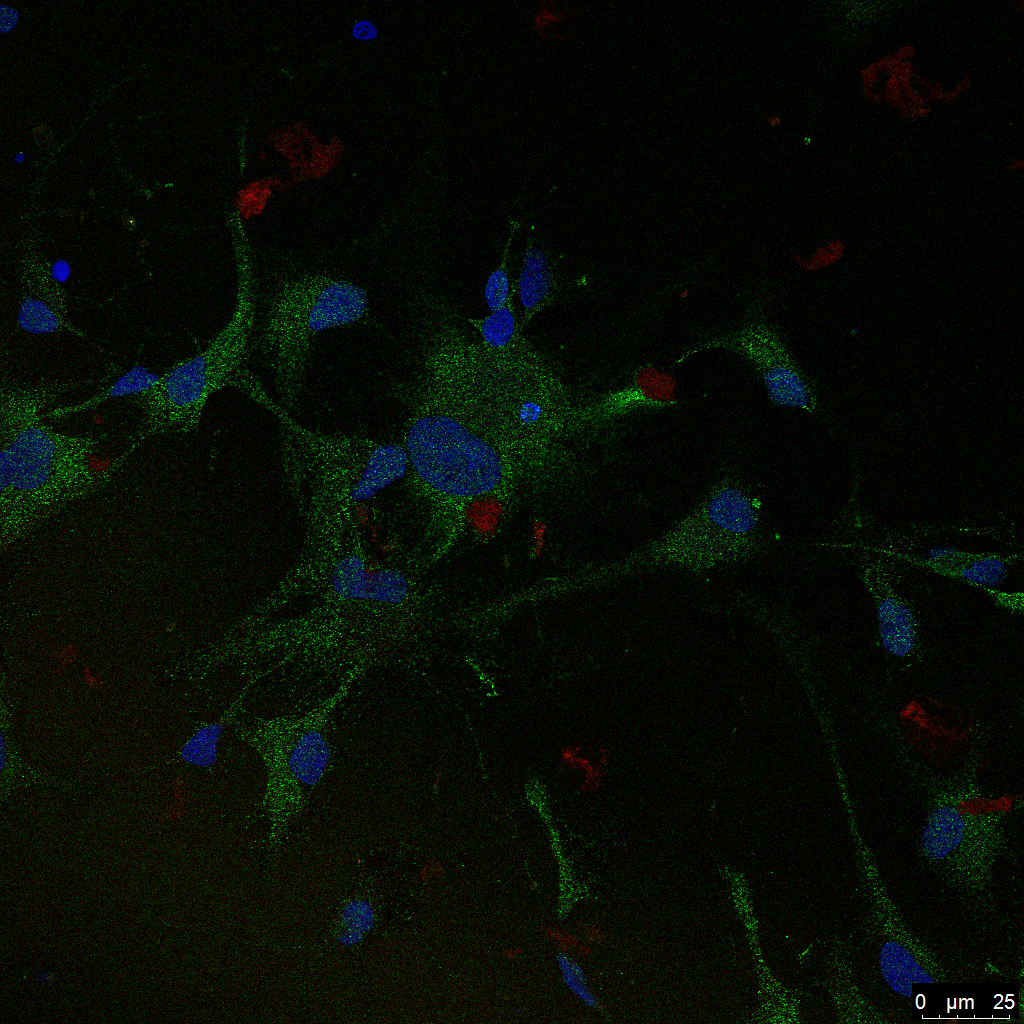

Supplement: Supplementary file 12 — Source data Fig. 6 [file 44319_2024_218_MOESM12_ESM.zip › Figure 6/6C/FLAG, Transduced 41NPC_41 NPC, FLAG, Dox 50 ng-ml 24h, -ADO, IMPDH2-488, cMyc-555 40x.tif]

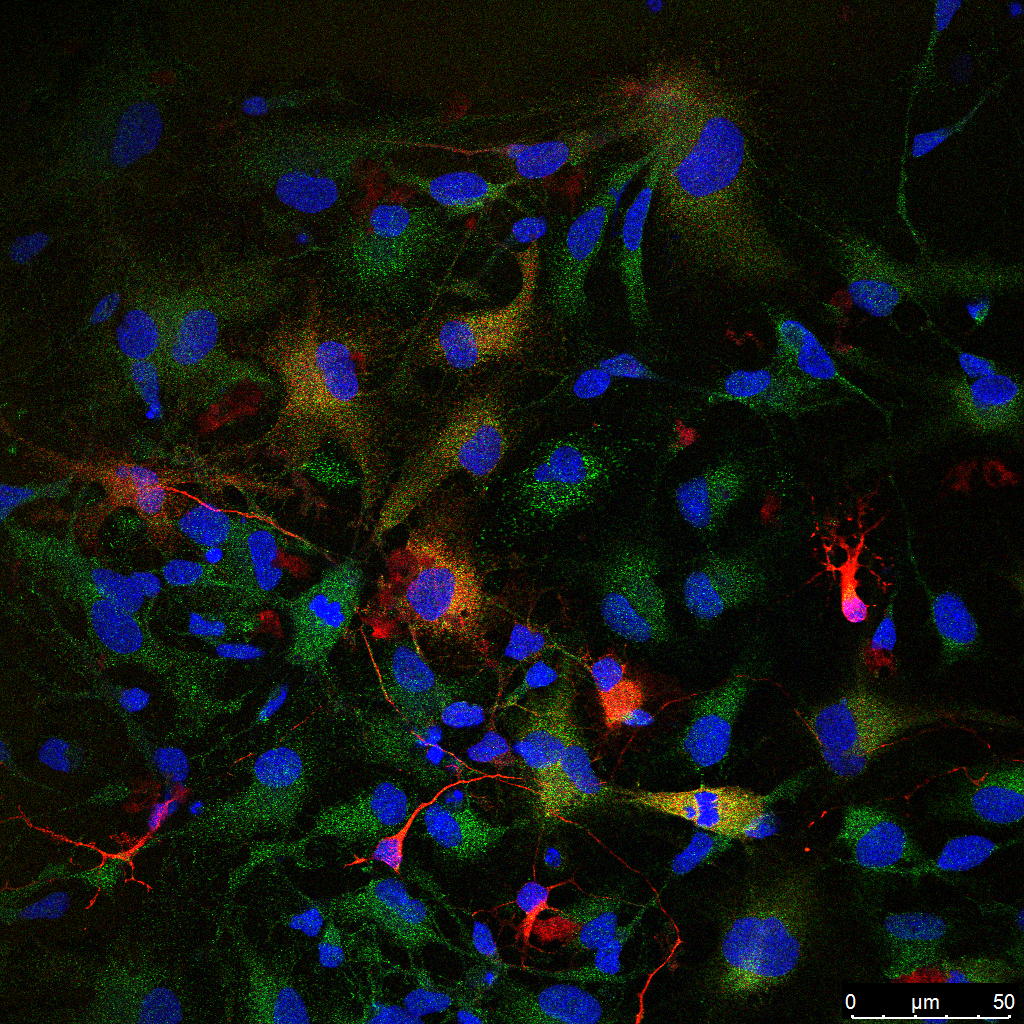

Supplement: Supplementary file 12 — Source data Fig. 6 [file 44319_2024_218_MOESM12_ESM.zip › Figure 6/6C/Y12A, Transduced 41NPC_41 NPC, Mut543, Dox 50 ng-ml 24h, -ADO, IMPDH2-488, cMyc-555 40x ZF090-II.tif]

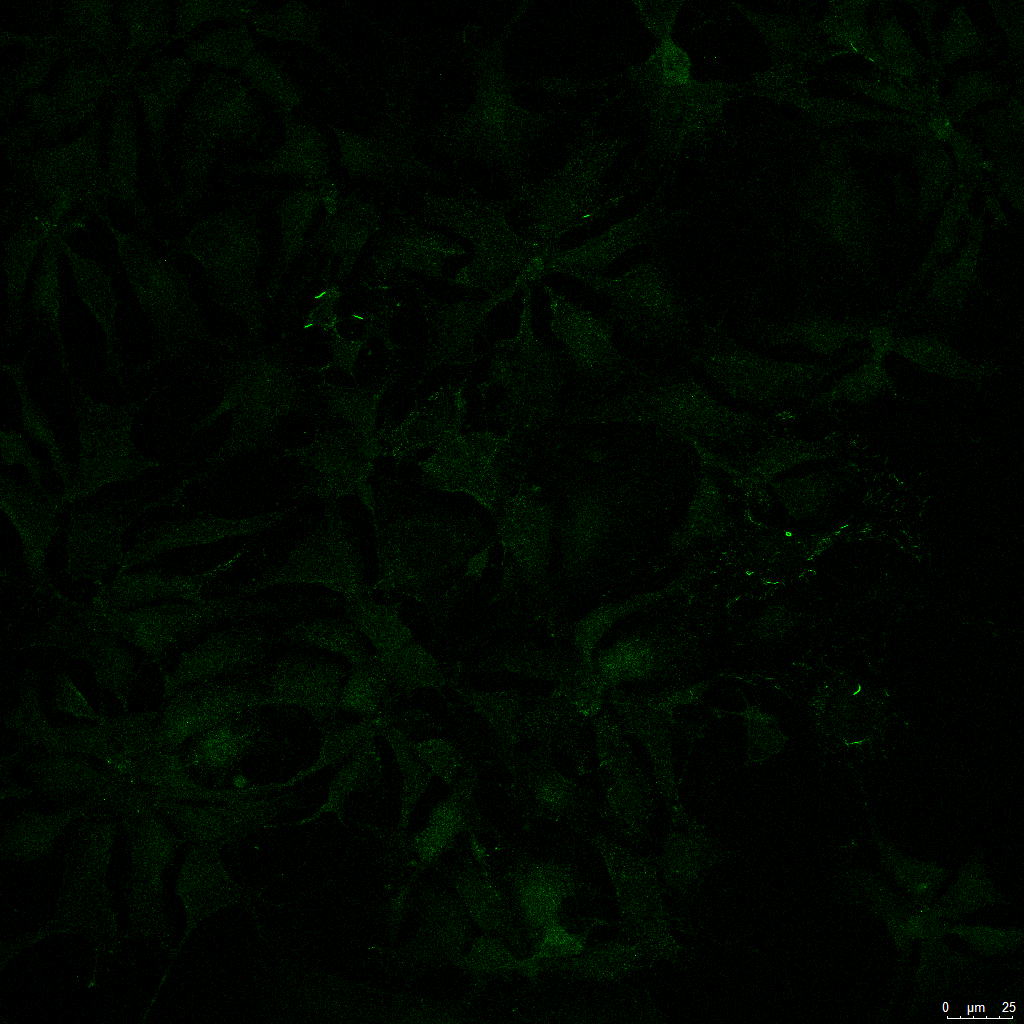

Supplement: Supplementary file 12 — Source data Fig. 6 [file 44319_2024_218_MOESM12_ESM.zip › Figure 6/6A/PCH9 NPCs/41,2 NPC, Ki67_41-2 NPC, ADO 10um 0h, IMPDH2-488, Ki67-555, Nestin-633, ZS 40x ZF75-II_ch01.tif]

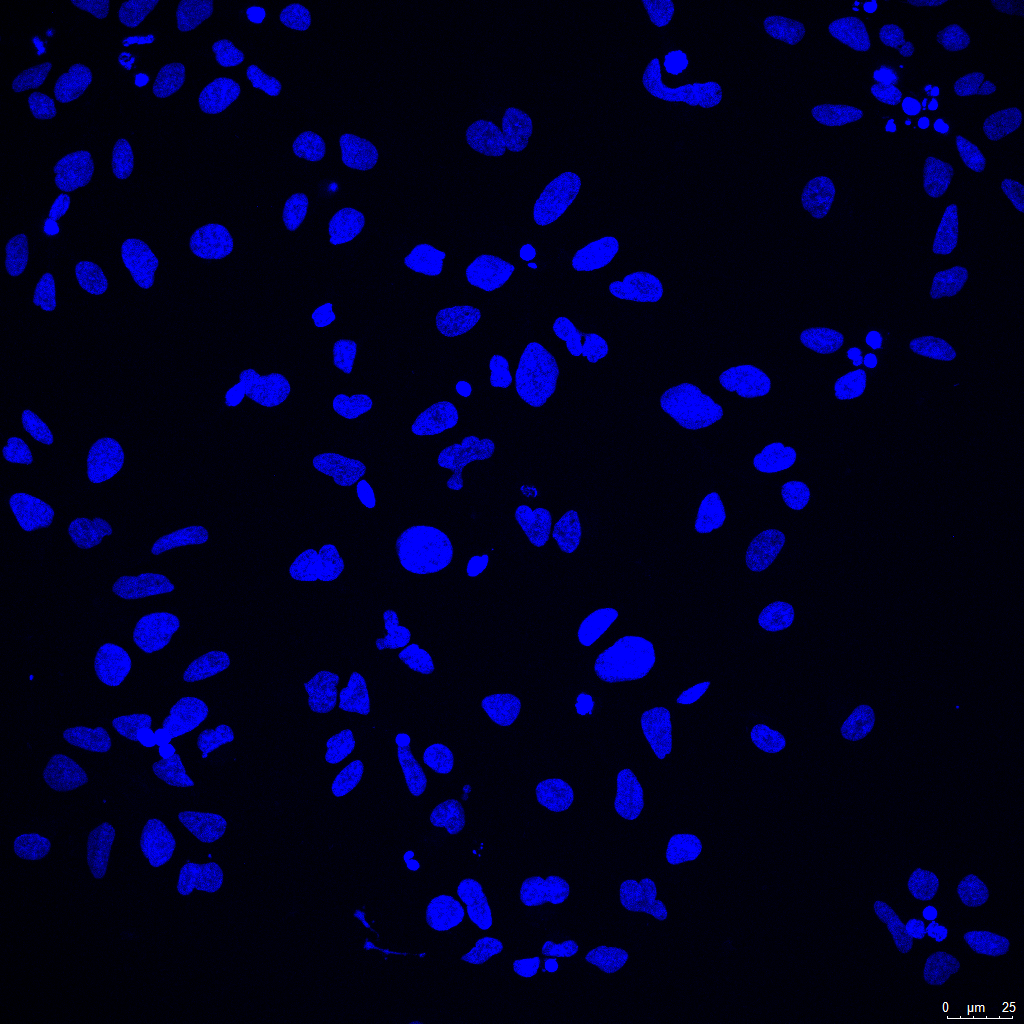

Supplement: Supplementary file 12 — Source data Fig. 6 [file 44319_2024_218_MOESM12_ESM.zip › Figure 6/6A/PCH9 NPCs/41,2 NPC, Ki67_41-2 NPC, ADO 10um 0h, IMPDH2-488, Ki67-555, Nestin-633, ZS 40x ZF75-II_ch00.tif]

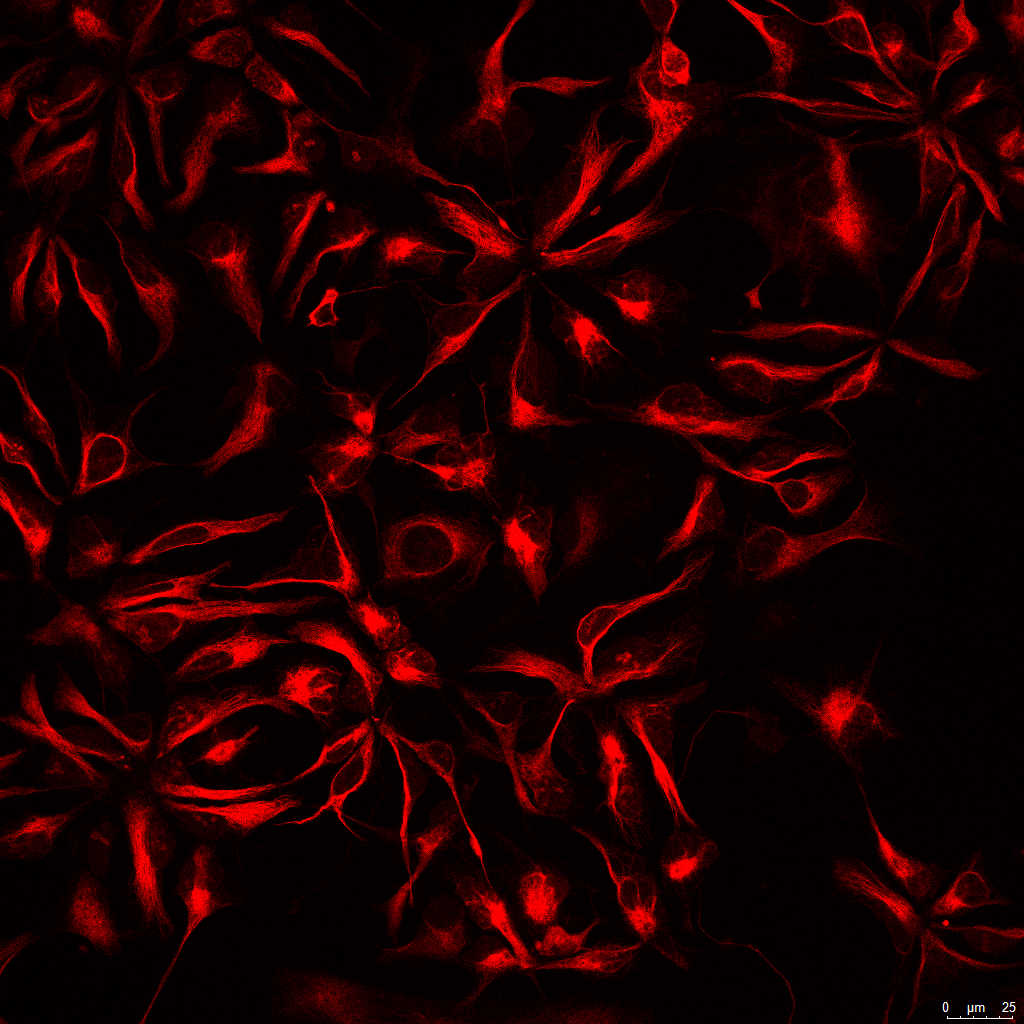

Supplement: Supplementary file 12 — Source data Fig. 6 [file 44319_2024_218_MOESM12_ESM.zip › Figure 6/6A/PCH9 NPCs/41,2 NPC, Ki67_41-2 NPC, ADO 10um 0h, IMPDH2-488, Ki67-555, Nestin-633, ZS 40x ZF75-II_ch03.tif]

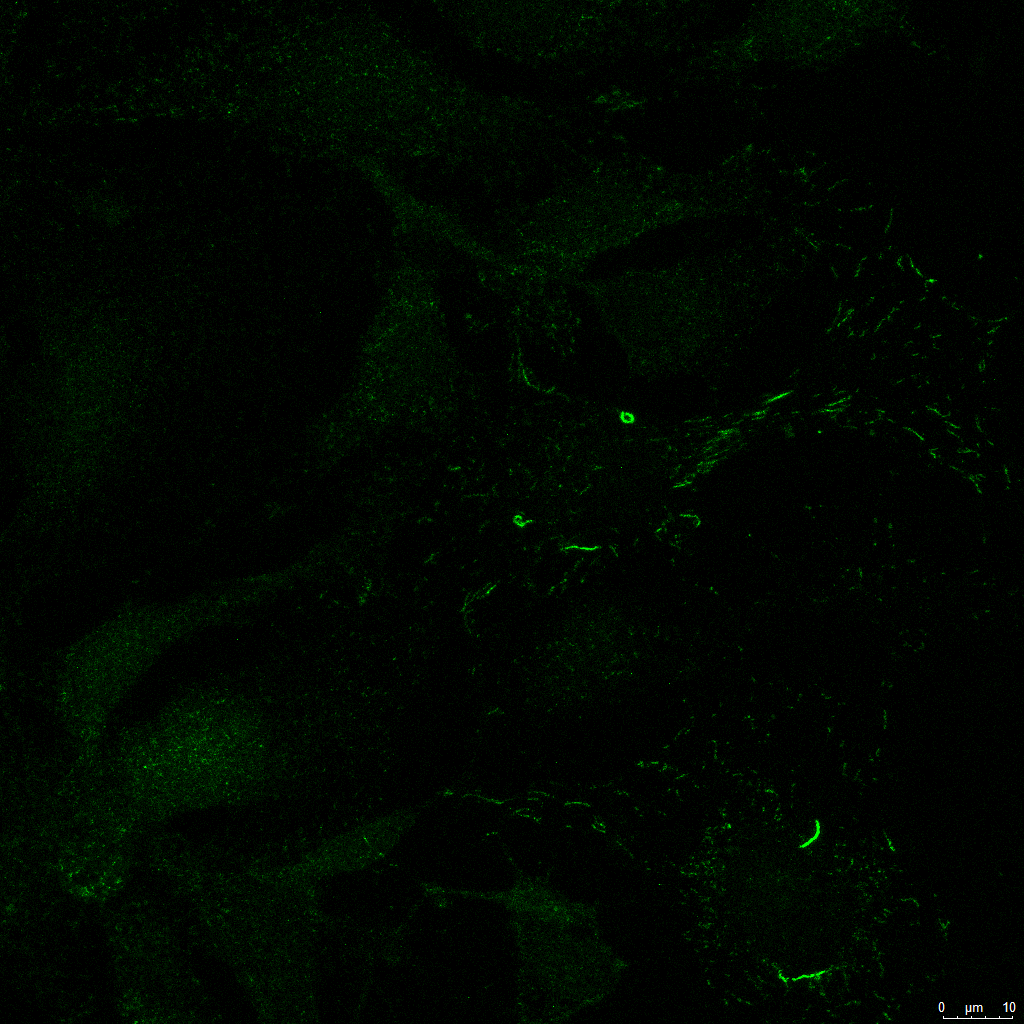

Supplement: Supplementary file 12 — Source data Fig. 6 [file 44319_2024_218_MOESM12_ESM.zip › Figure 6/6A/PCH9 NPCs/41,2 NPC, Ki67_41-2 NPC, ADO 10um 0h, IMPDH2-488, Ki67-555, Nestin-633, ZS 40x ZF2-II_ch01.tif]

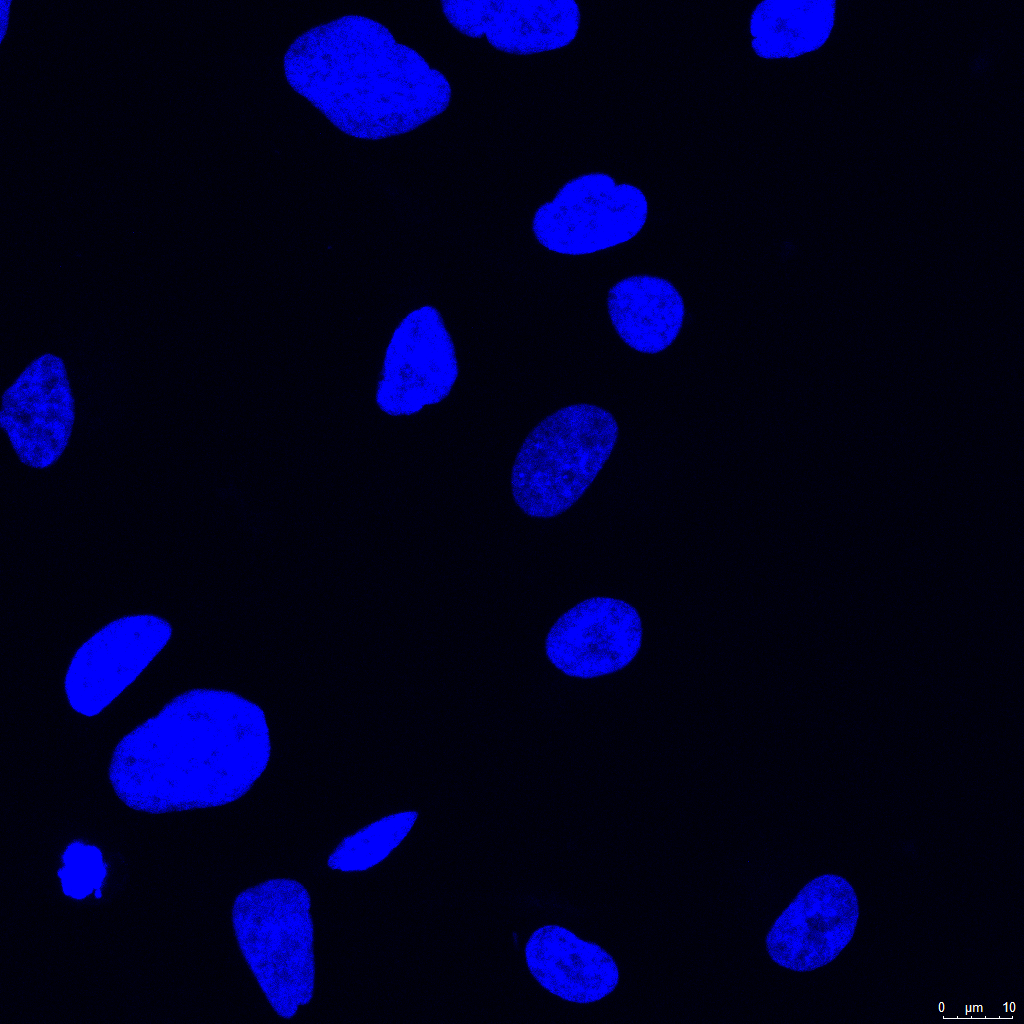

Supplement: Supplementary file 12 — Source data Fig. 6 [file 44319_2024_218_MOESM12_ESM.zip › Figure 6/6A/PCH9 NPCs/41,2 NPC, Ki67_41-2 NPC, ADO 10um 0h, IMPDH2-488, Ki67-555, Nestin-633, ZS 40x ZF2-II_ch00.tif]

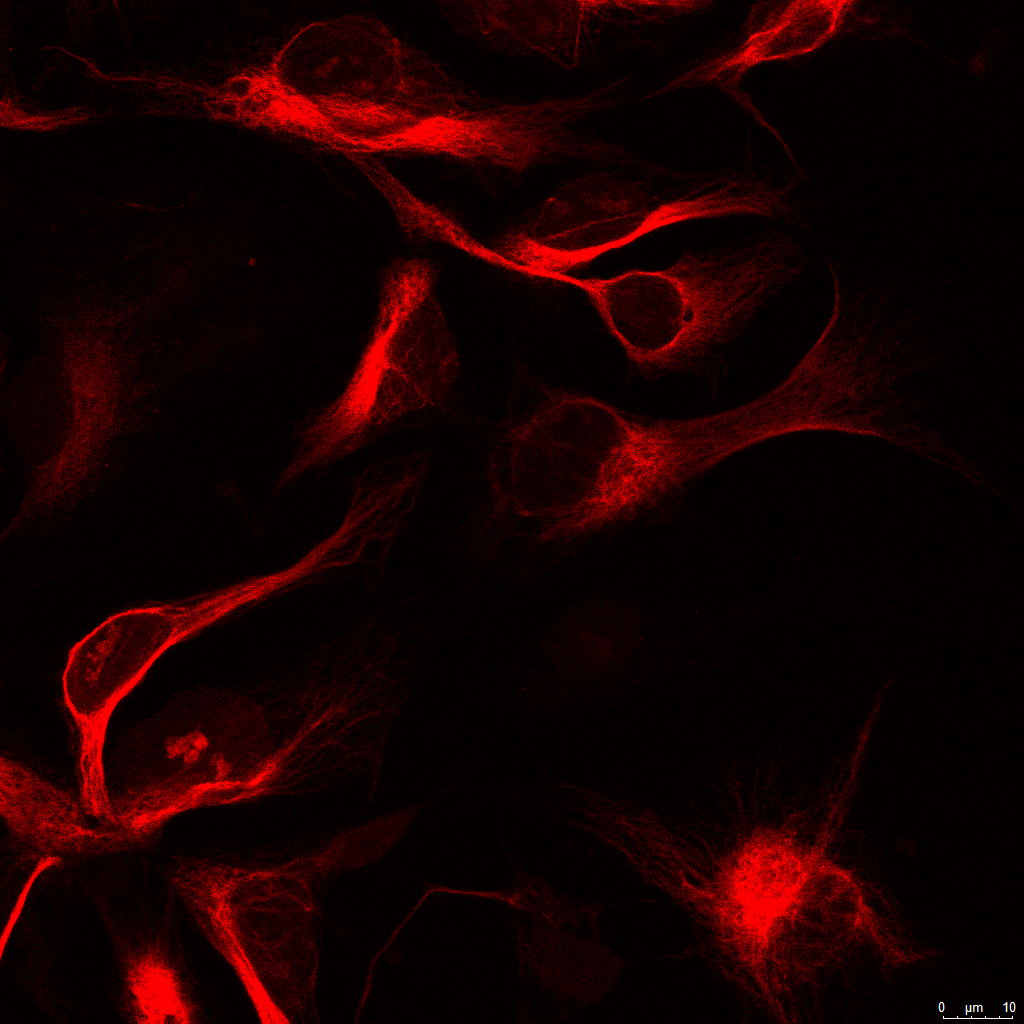

Supplement: Supplementary file 12 — Source data Fig. 6 [file 44319_2024_218_MOESM12_ESM.zip › Figure 6/6A/PCH9 NPCs/41,2 NPC, Ki67_41-2 NPC, ADO 10um 0h, IMPDH2-488, Ki67-555, Nestin-633, ZS 40x ZF2-II_ch03.tif]

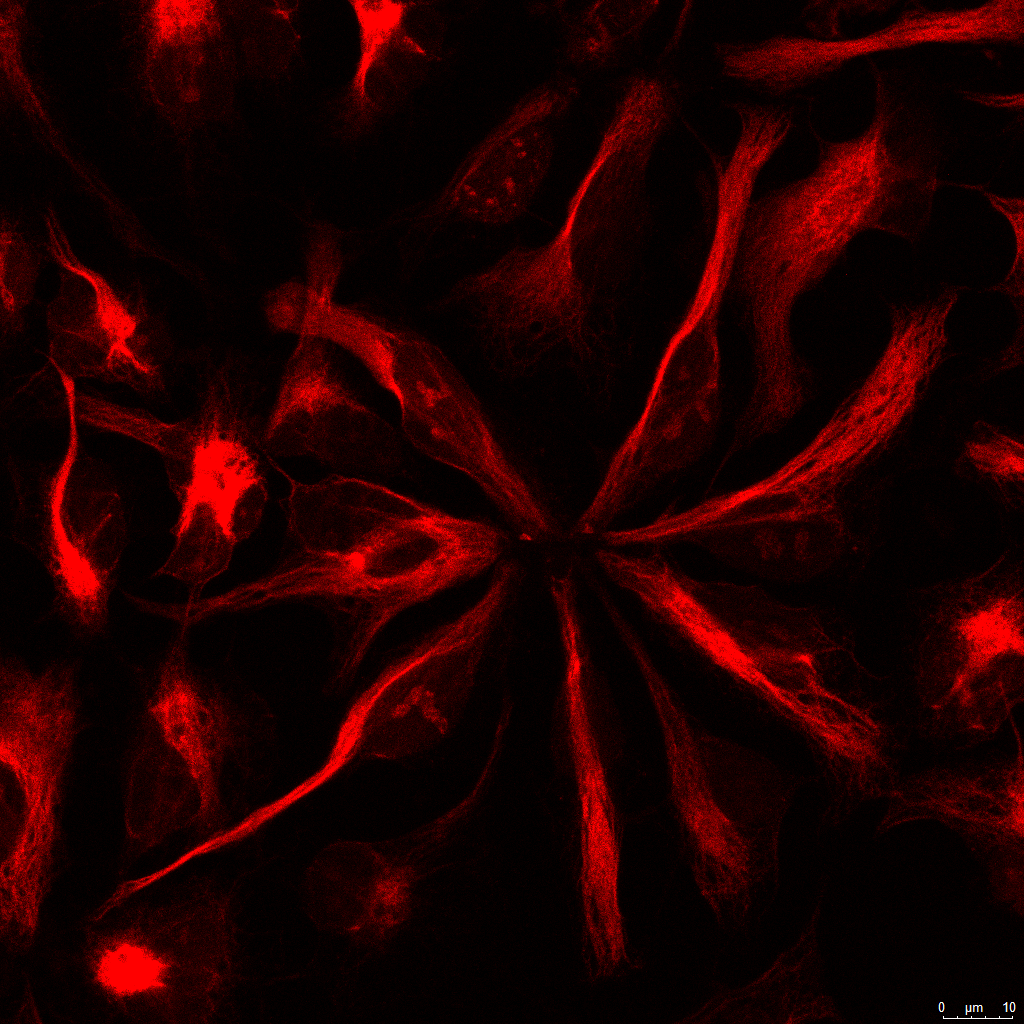

Supplement: Supplementary file 12 — Source data Fig. 6 [file 44319_2024_218_MOESM12_ESM.zip › Figure 6/6A/Control NPCs/39 NPC, Ki67_39 NPC, ADO 10um 0h, IMPDH2-488, Ki67-555, Nestin-633, ZS 40x ZF2-VII_ch03.tif]

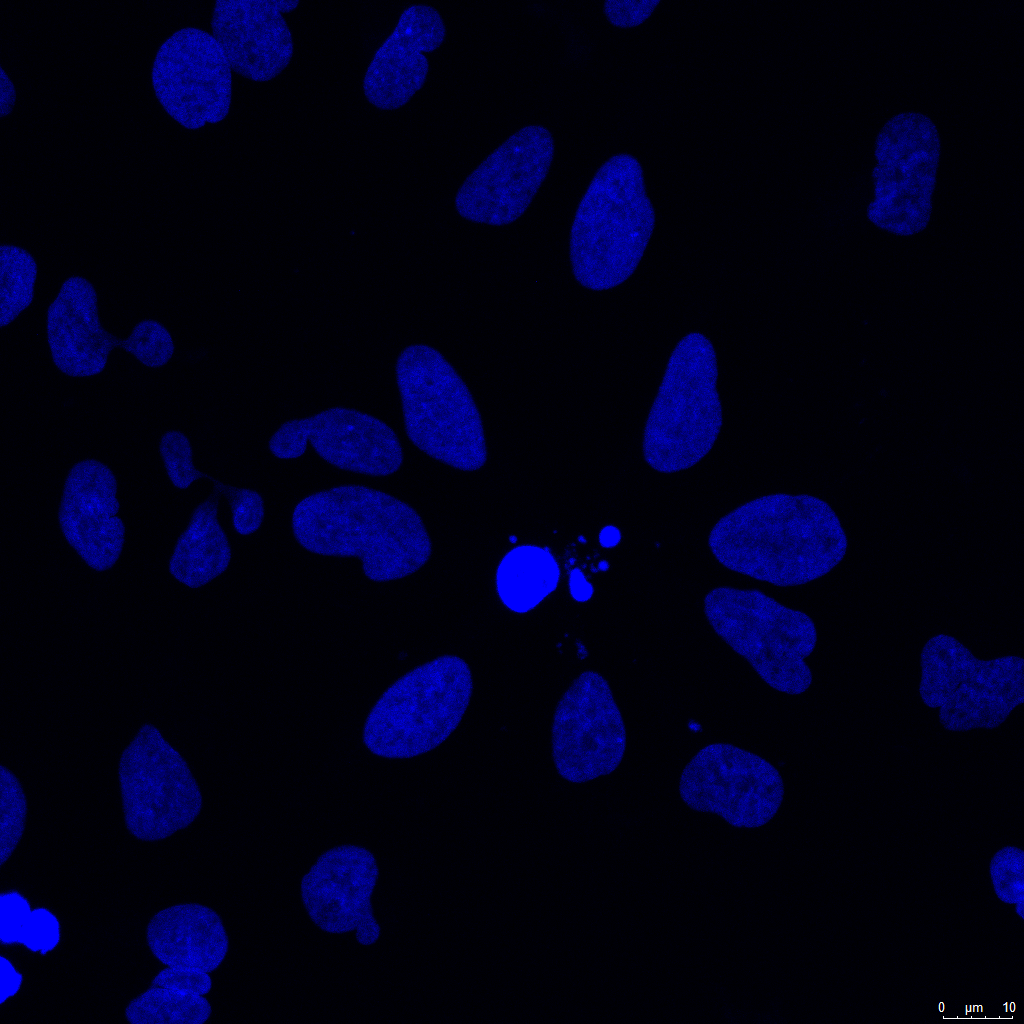

Supplement: Supplementary file 12 — Source data Fig. 6 [file 44319_2024_218_MOESM12_ESM.zip › Figure 6/6A/Control NPCs/39 NPC, Ki67_39 NPC, ADO 10um 0h, IMPDH2-488, Ki67-555, Nestin-633, ZS 40x ZF2-VII_ch00.tif]

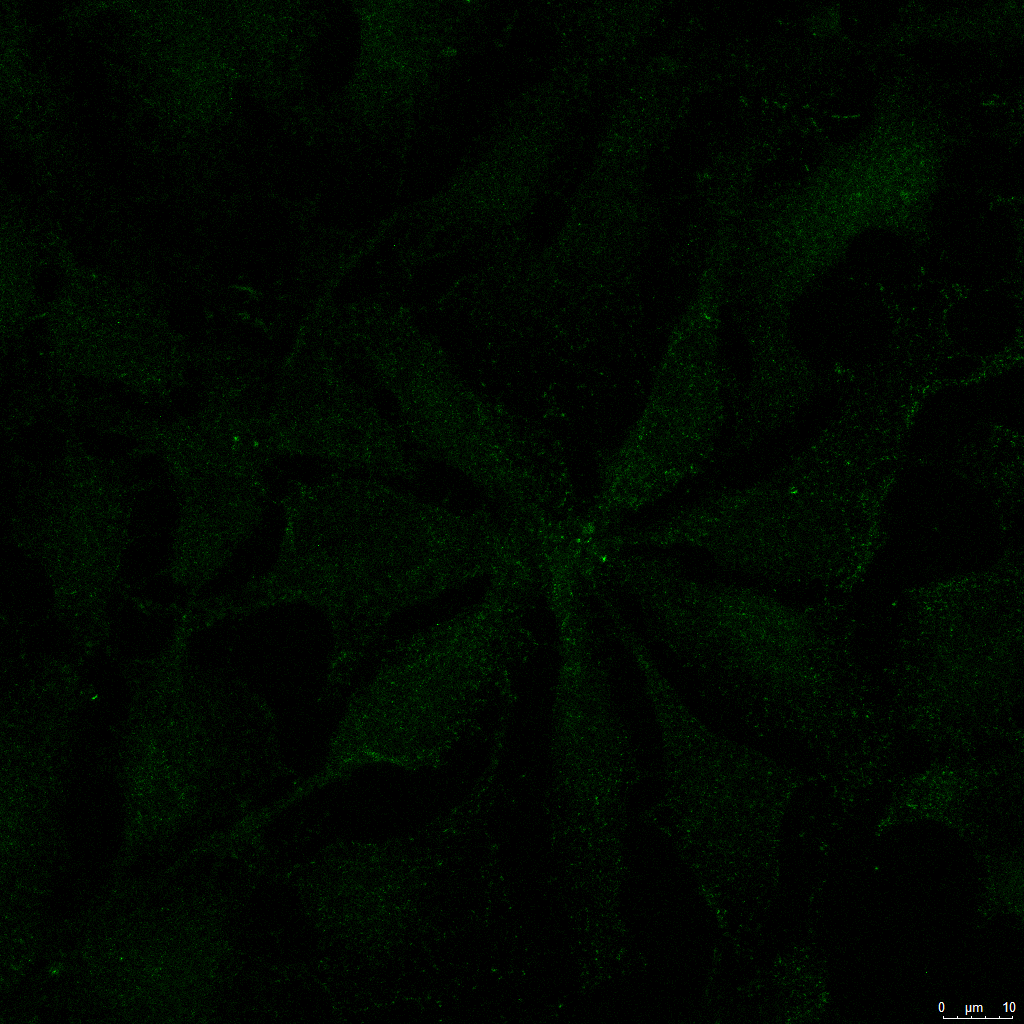

Supplement: Supplementary file 12 — Source data Fig. 6 [file 44319_2024_218_MOESM12_ESM.zip › Figure 6/6A/Control NPCs/39 NPC, Ki67_39 NPC, ADO 10um 0h, IMPDH2-488, Ki67-555, Nestin-633, ZS 40x ZF2-VII_ch01.tif]

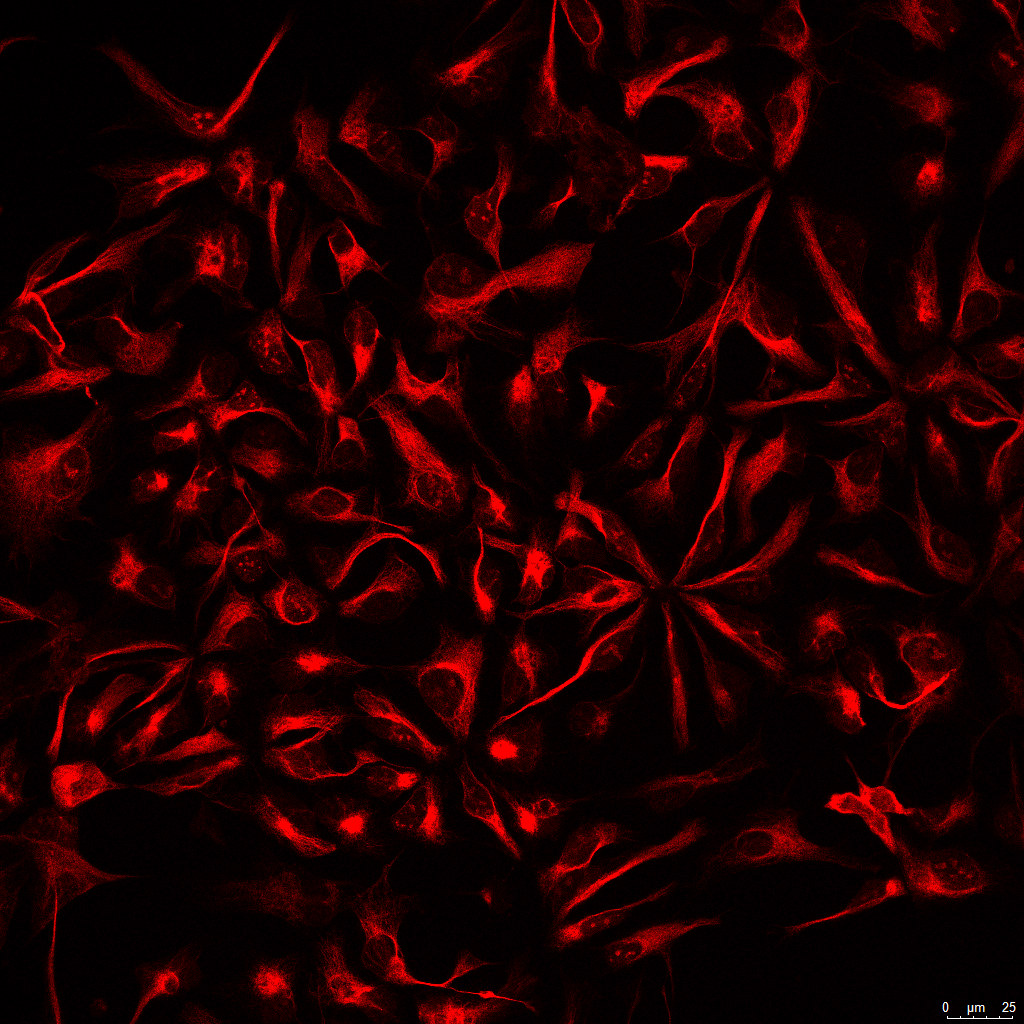

Supplement: Supplementary file 12 — Source data Fig. 6 [file 44319_2024_218_MOESM12_ESM.zip › Figure 6/6A/Control NPCs/39 NPC, Ki67_39 NPC, ADO 10um 0h, IMPDH2-488, Ki67-555, Nestin-633, ZS 40x ZF075-VII_ch03.tif]

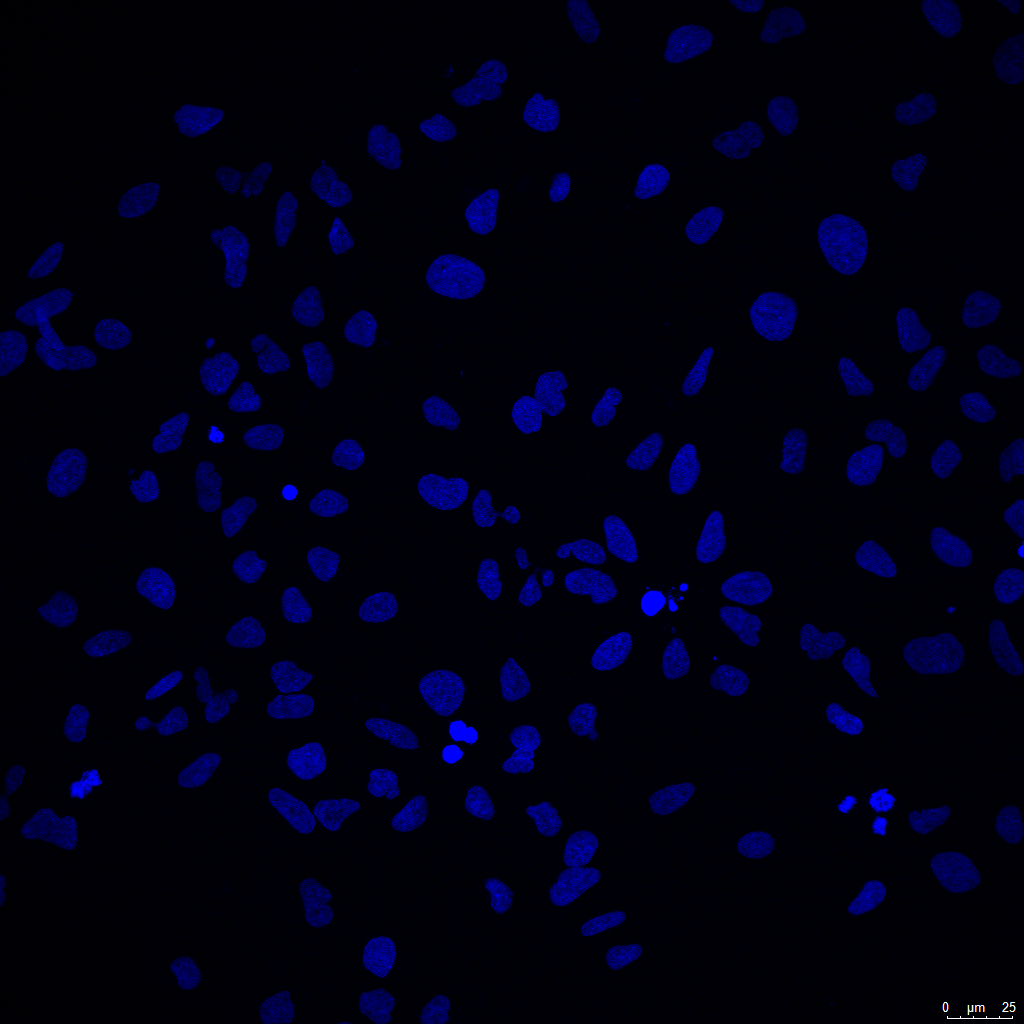

Supplement: Supplementary file 12 — Source data Fig. 6 [file 44319_2024_218_MOESM12_ESM.zip › Figure 6/6A/Control NPCs/39 NPC, Ki67_39 NPC, ADO 10um 0h, IMPDH2-488, Ki67-555, Nestin-633, ZS 40x ZF075-VII_ch00.tif]

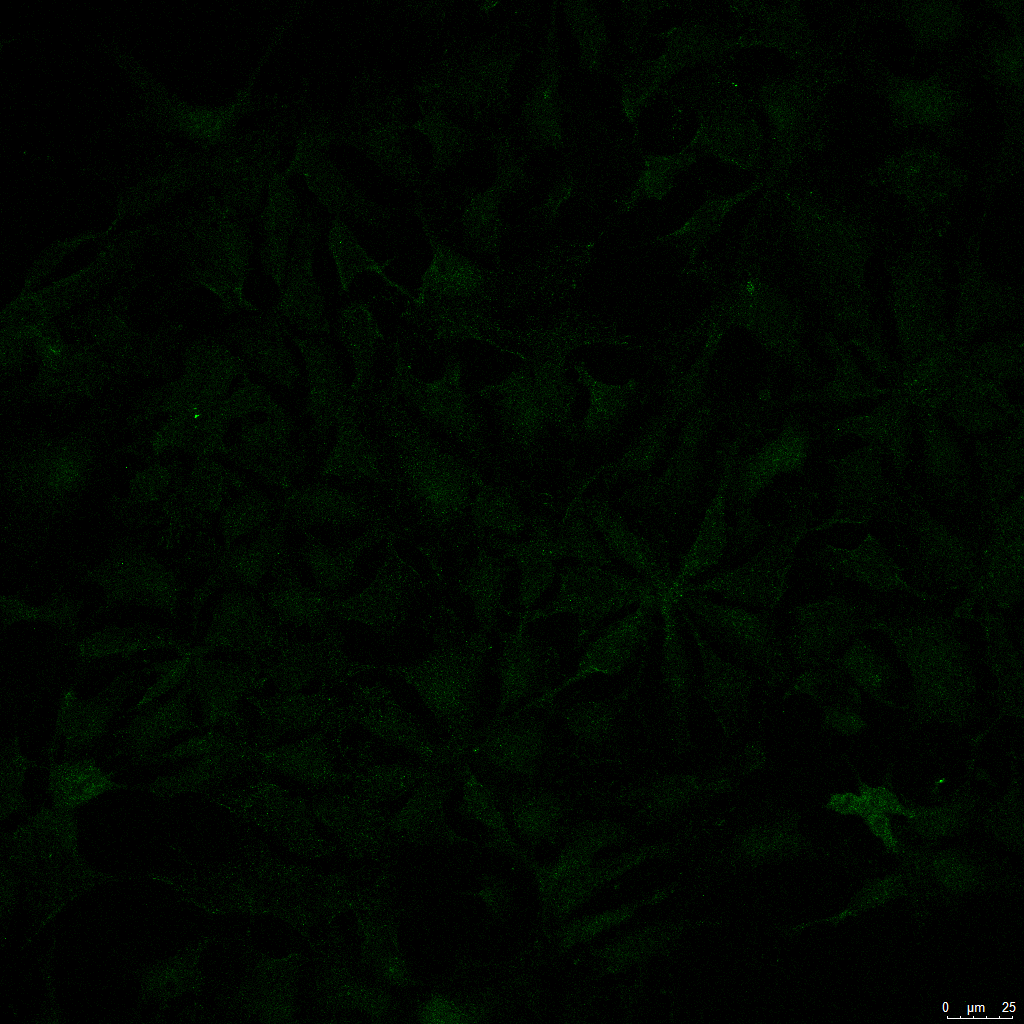

Supplement: Supplementary file 12 — Source data Fig. 6 [file 44319_2024_218_MOESM12_ESM.zip › Figure 6/6A/Control NPCs/39 NPC, Ki67_39 NPC, ADO 10um 0h, IMPDH2-488, Ki67-555, Nestin-633, ZS 40x ZF075-VII_ch01.tif]

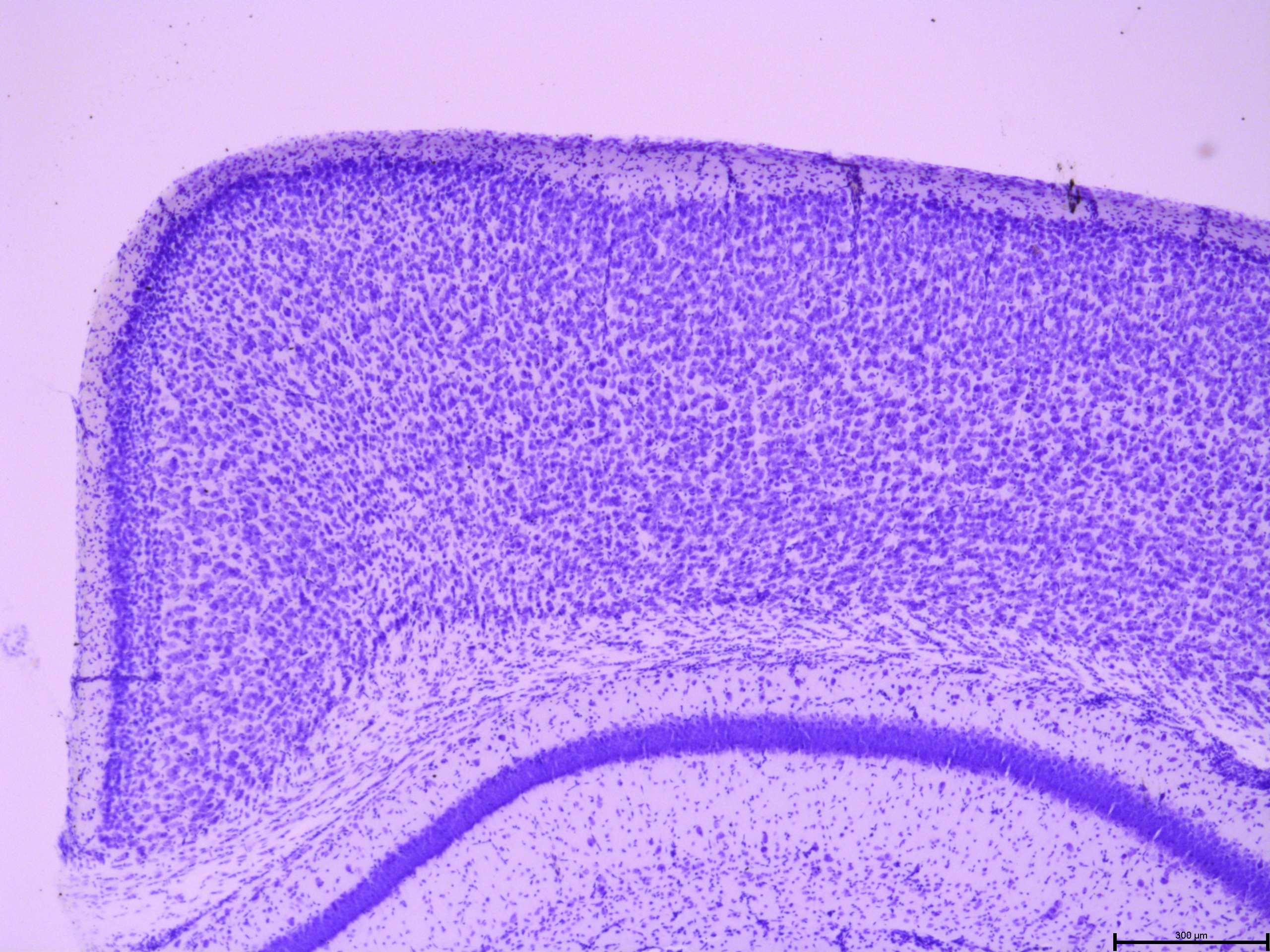

Supplement: Supplementary file 13 — Figure EV1 Source Data [file 44319_2024_218_MOESM13_ESM.zip › Figure EV1/Ctrl/609 Het, Nissl_609 Het, Ctx 5x-III_ch00.tif]

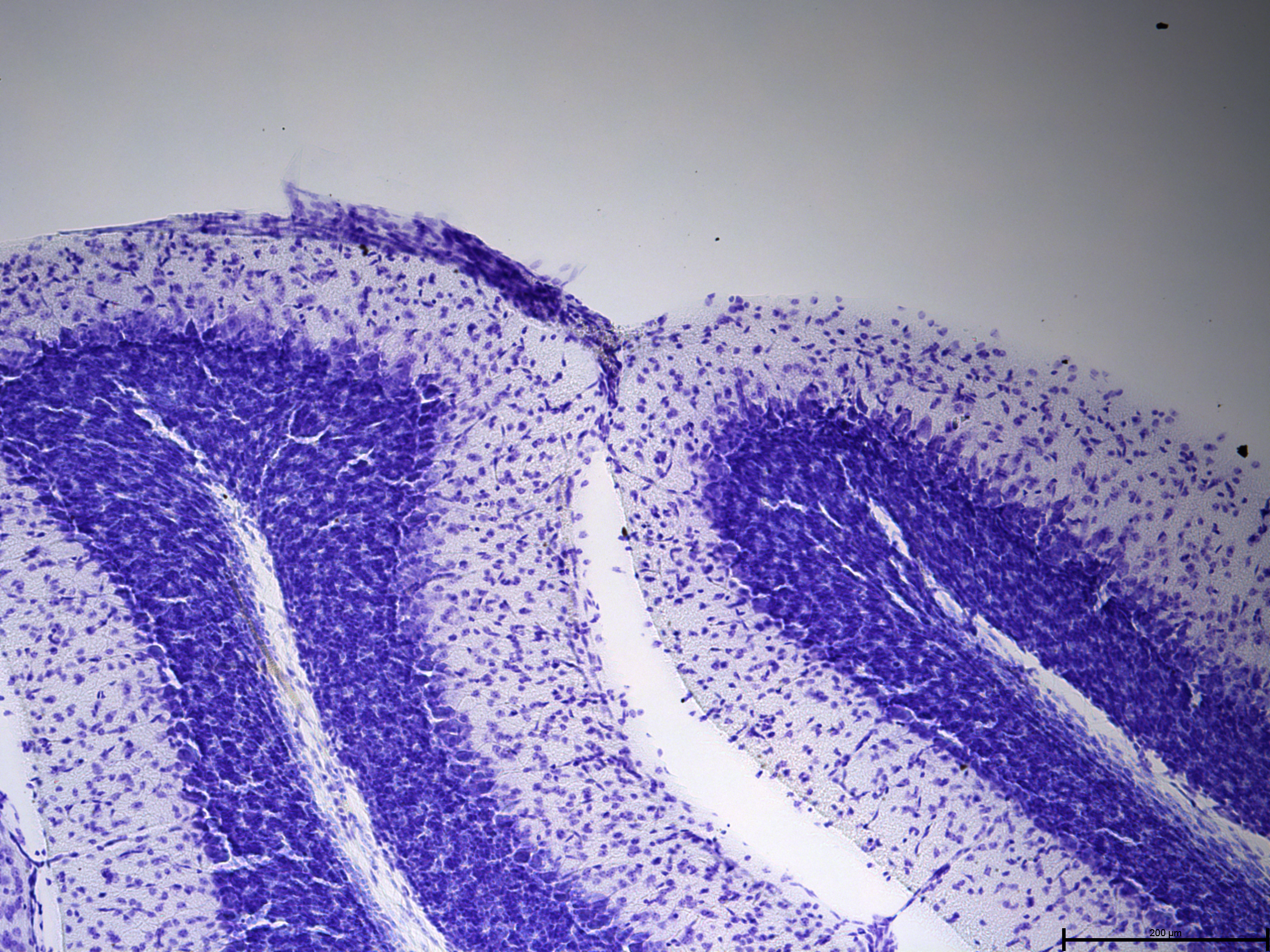

Supplement: Supplementary file 13 — Figure EV1 Source Data [file 44319_2024_218_MOESM13_ESM.zip › Figure EV1/Ctrl/A609Het, Cb_A609Het, S1, L4-5 10X-3_ch00.tif]

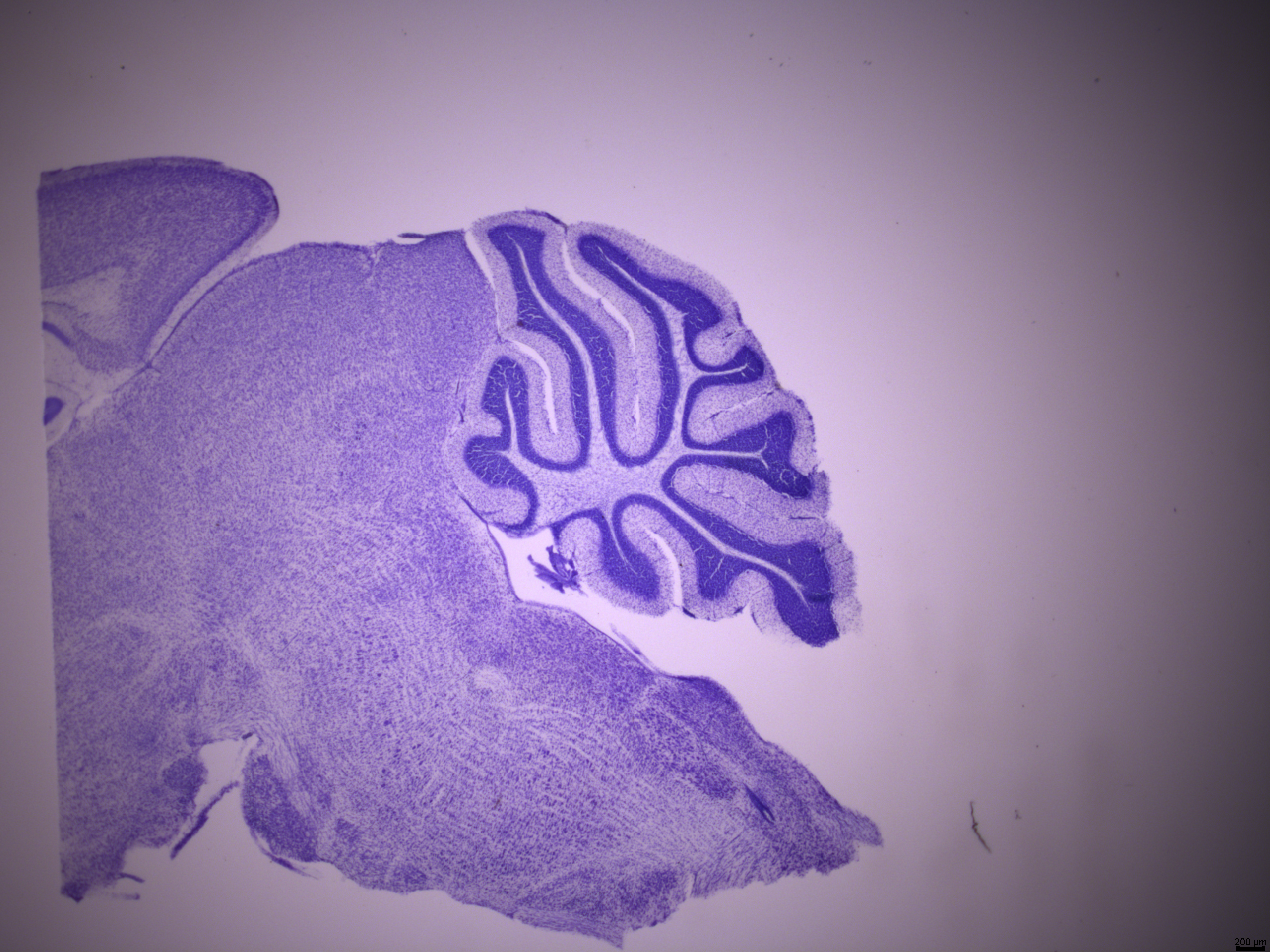

Supplement: Supplementary file 13 — Figure EV1 Source Data [file 44319_2024_218_MOESM13_ESM.zip › Figure EV1/Ctrl/A609Het, Cb_A609Het, S1, 1,25X_ch00.tif]

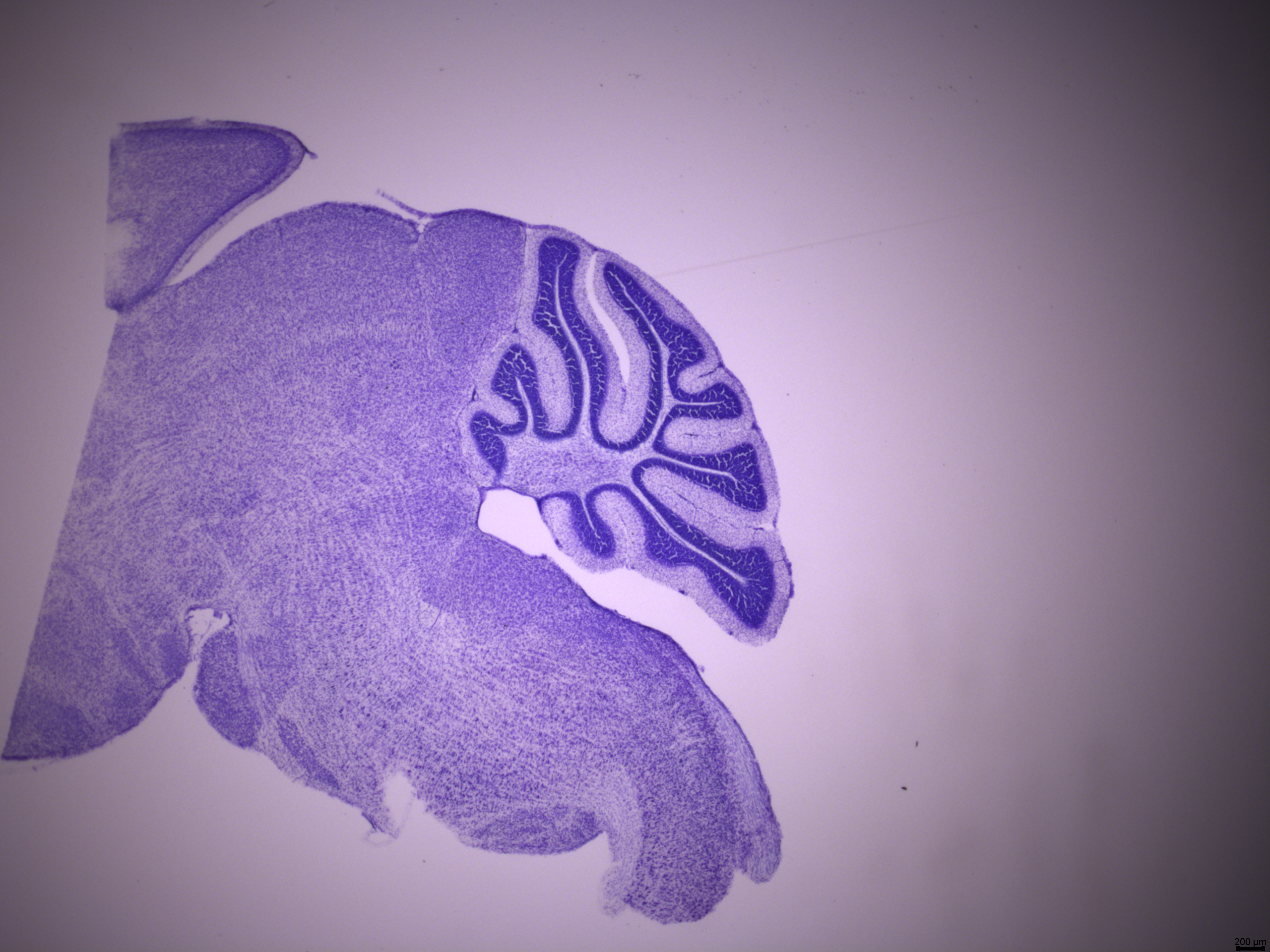

Supplement: Supplementary file 13 — Figure EV1 Source Data [file 44319_2024_218_MOESM13_ESM.zip › Figure EV1/dKO/A614dKO, Cb_A614dKO, S2, L10 1,25-2_ch00.tif]

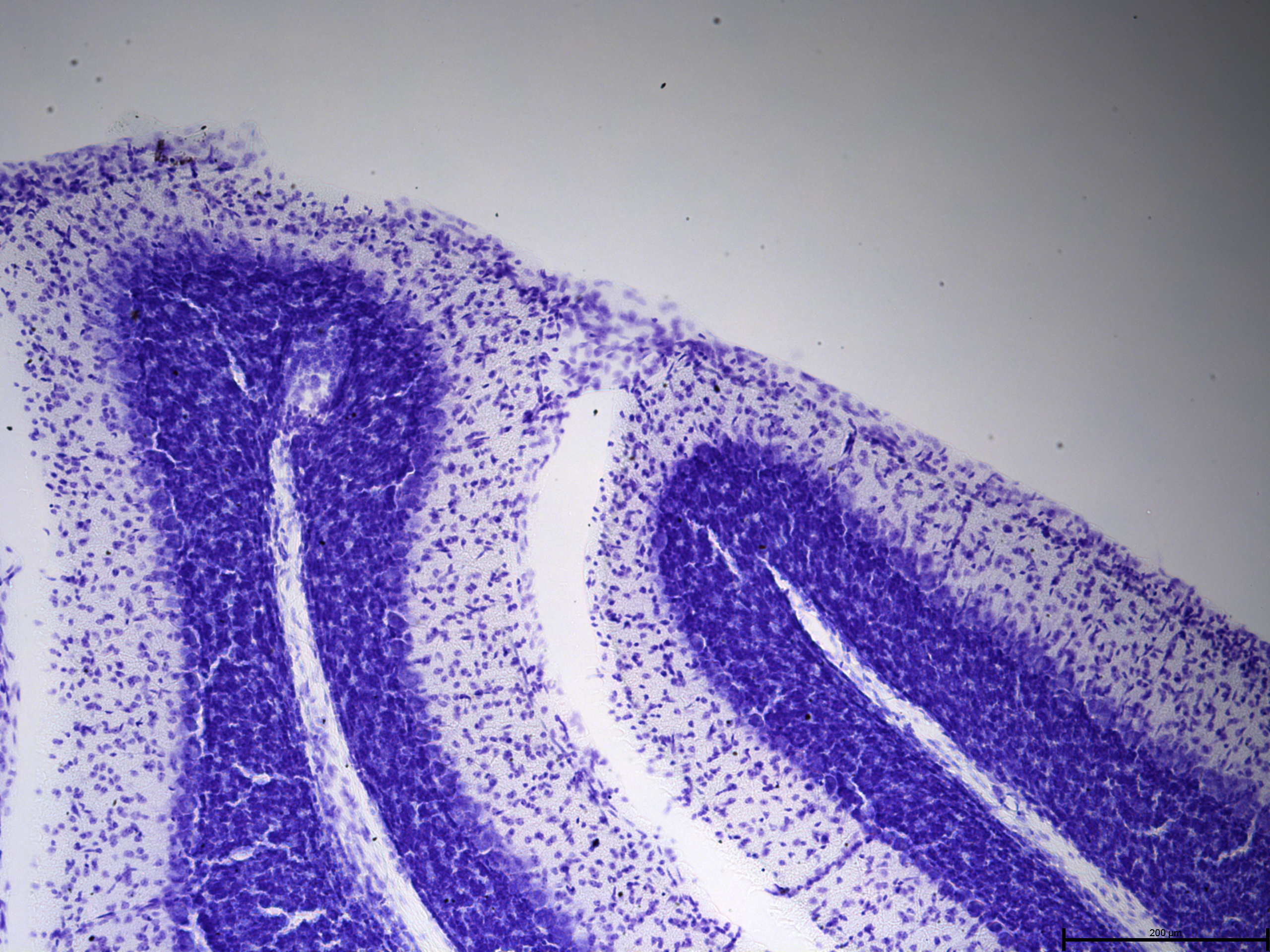

Supplement: Supplementary file 13 — Figure EV1 Source Data [file 44319_2024_218_MOESM13_ESM.zip › Figure EV1/dKO/A614dKO, Cb_A614dKO, S2, L4-5 10X-2_ch00.tif]

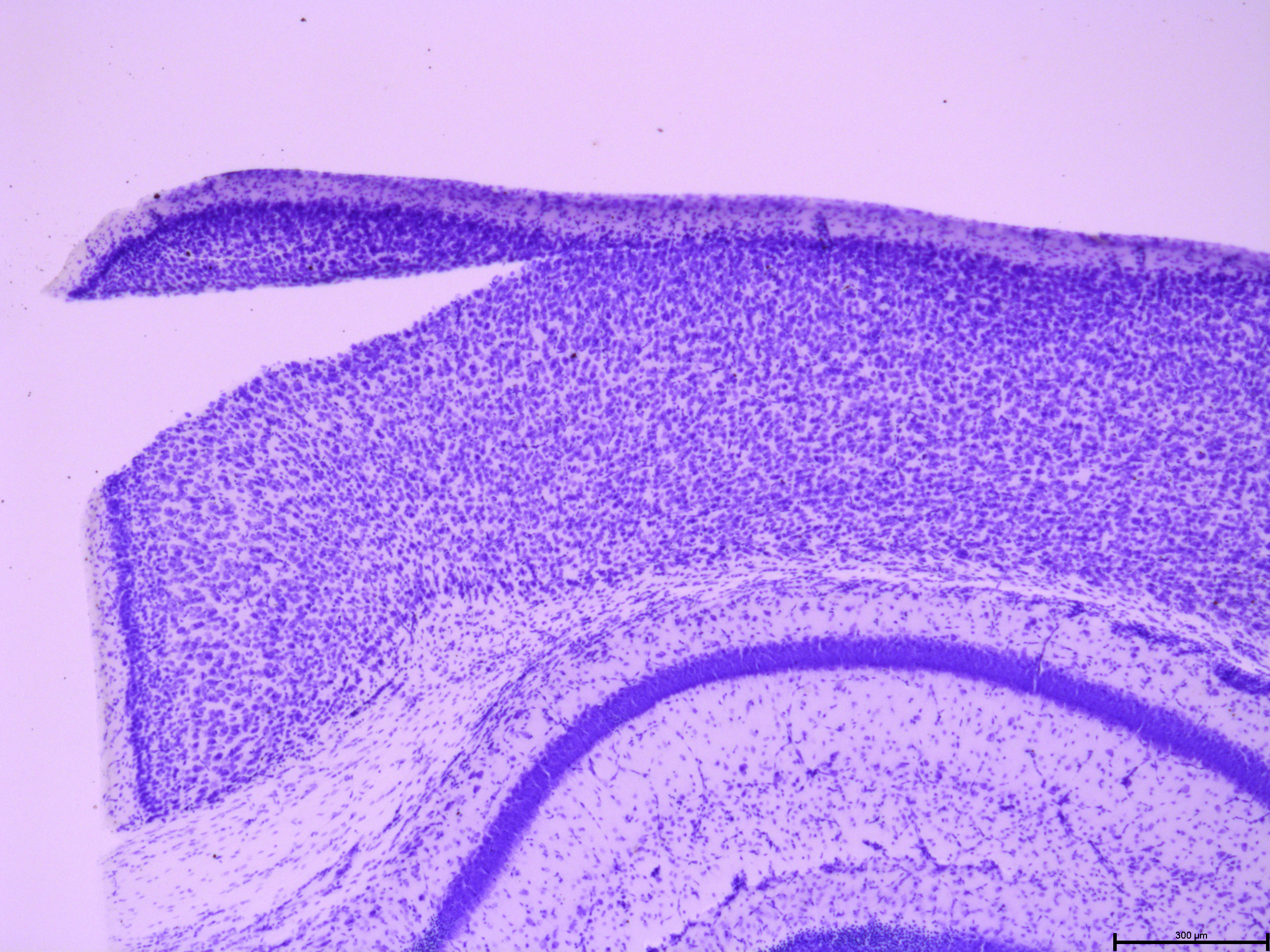

Supplement: Supplementary file 13 — Figure EV1 Source Data [file 44319_2024_218_MOESM13_ESM.zip › Figure EV1/dKO/Nissl_614dKO, Ctx 5x_ch00.tif]

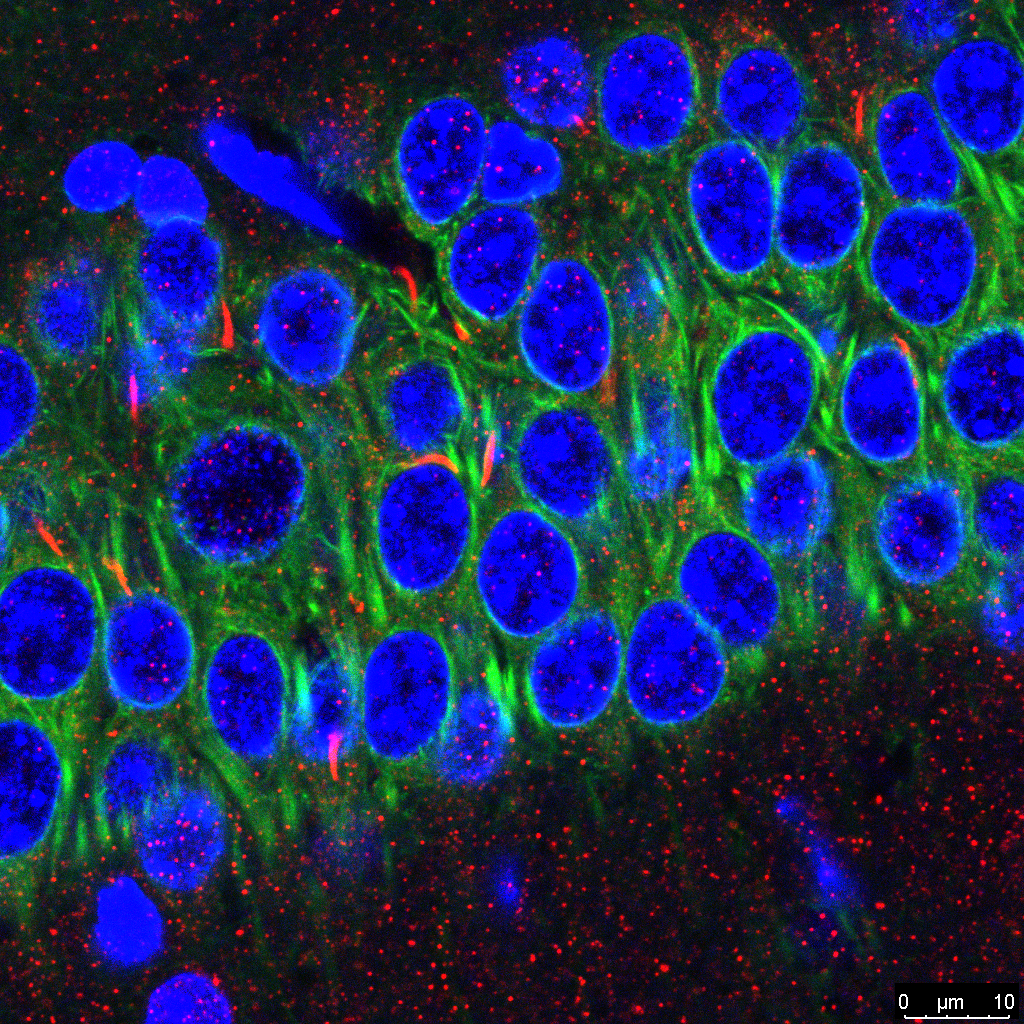

Supplement: Supplementary file 14 — Figure EV2 Source Data [file 44319_2024_218_MOESM14_ESM.zip › Figure EV2/2D/475p15 dKO IMPDH, Ace tub_480p15 dKO, IMPDH2 555, Ace tub 488, CA1 40x ZF3.tif]

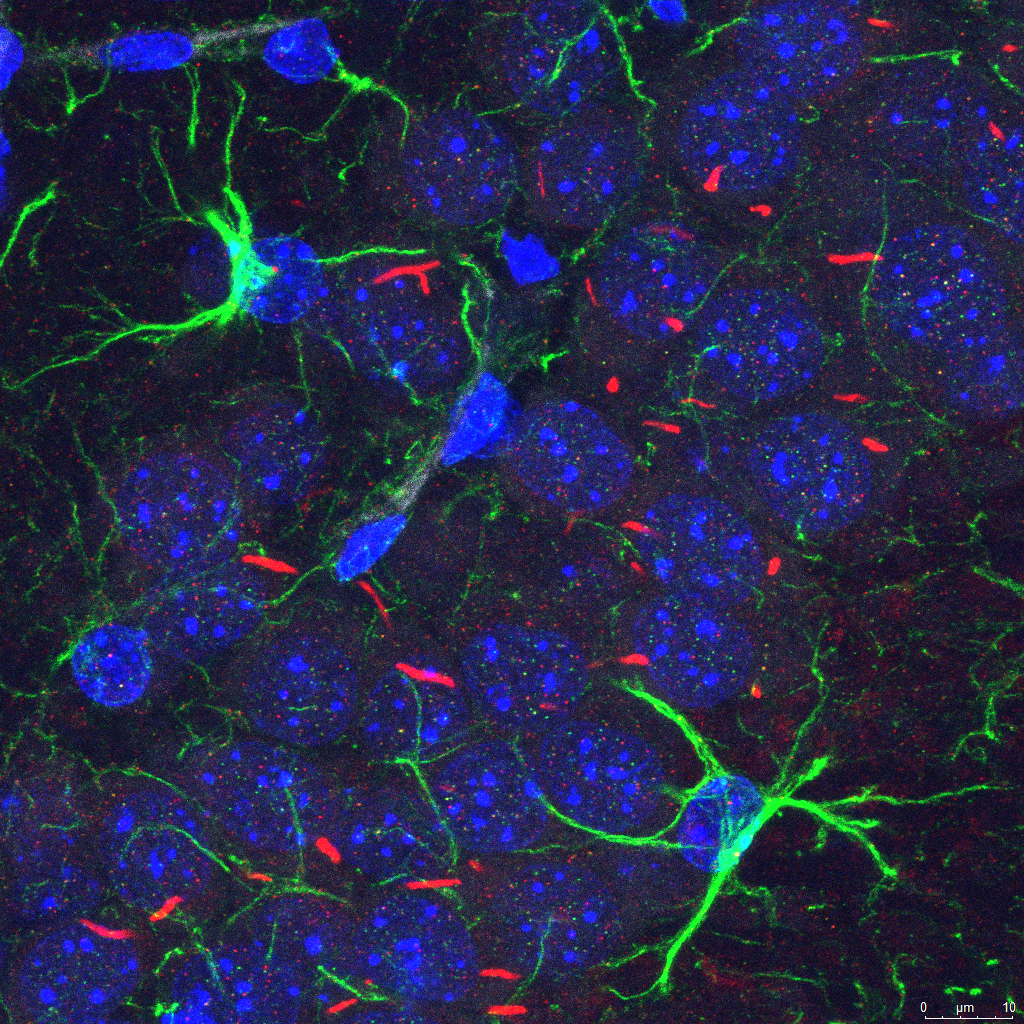

Supplement: Supplementary file 15 — Figure EV3 Source Data [file 44319_2024_218_MOESM15_ESM.zip › Figure EV3/3A/A162p19dKO_A162p19dKO, S3 CA2-3, GFAP488-G, IMPDH555-R, NeuN633-W, ZS 40xZF2,5-I.tif]

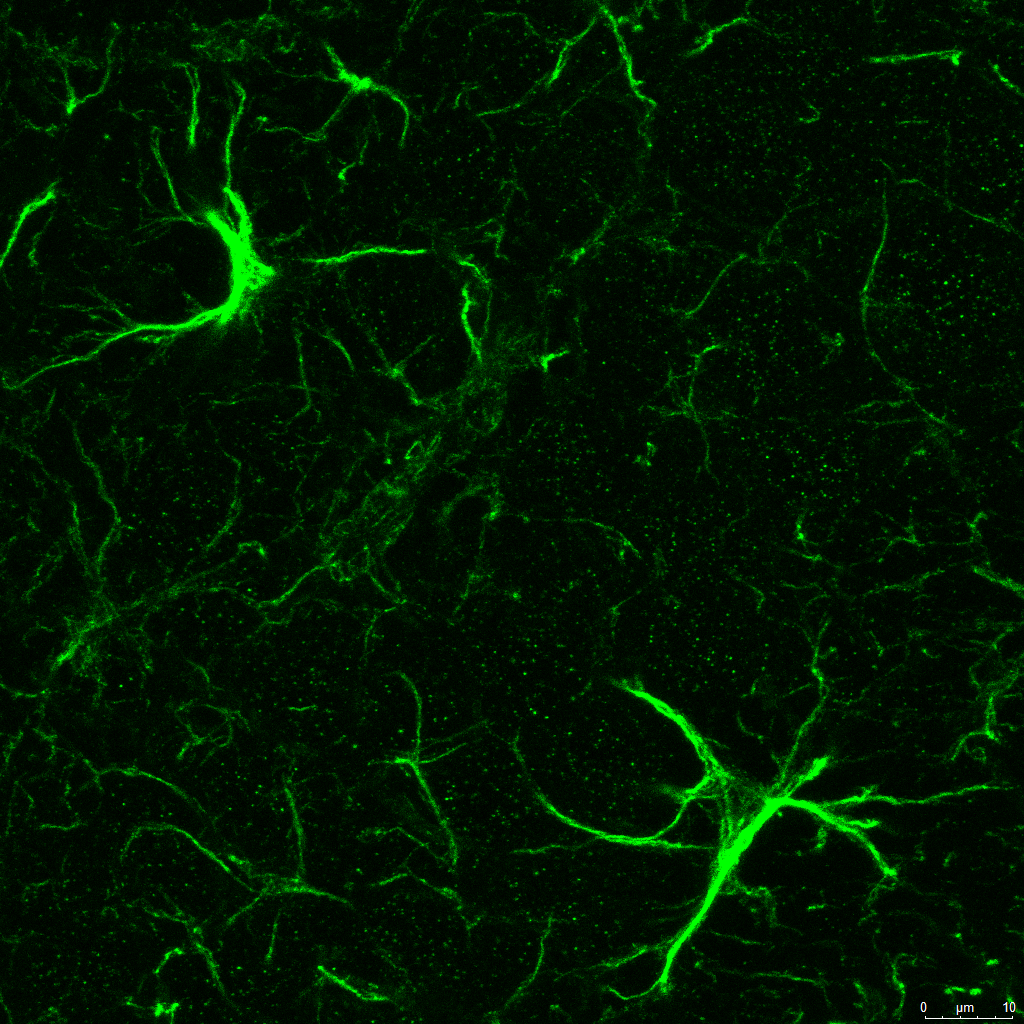

Supplement: Supplementary file 15 — Figure EV3 Source Data [file 44319_2024_218_MOESM15_ESM.zip › Figure EV3/3A/A162p19dKO_A162p19dKO, S3 CA2-3, GFAP488-G, IMPDH555-R, NeuN633-W, ZS 40xZF2,5-I_ch01.tif]

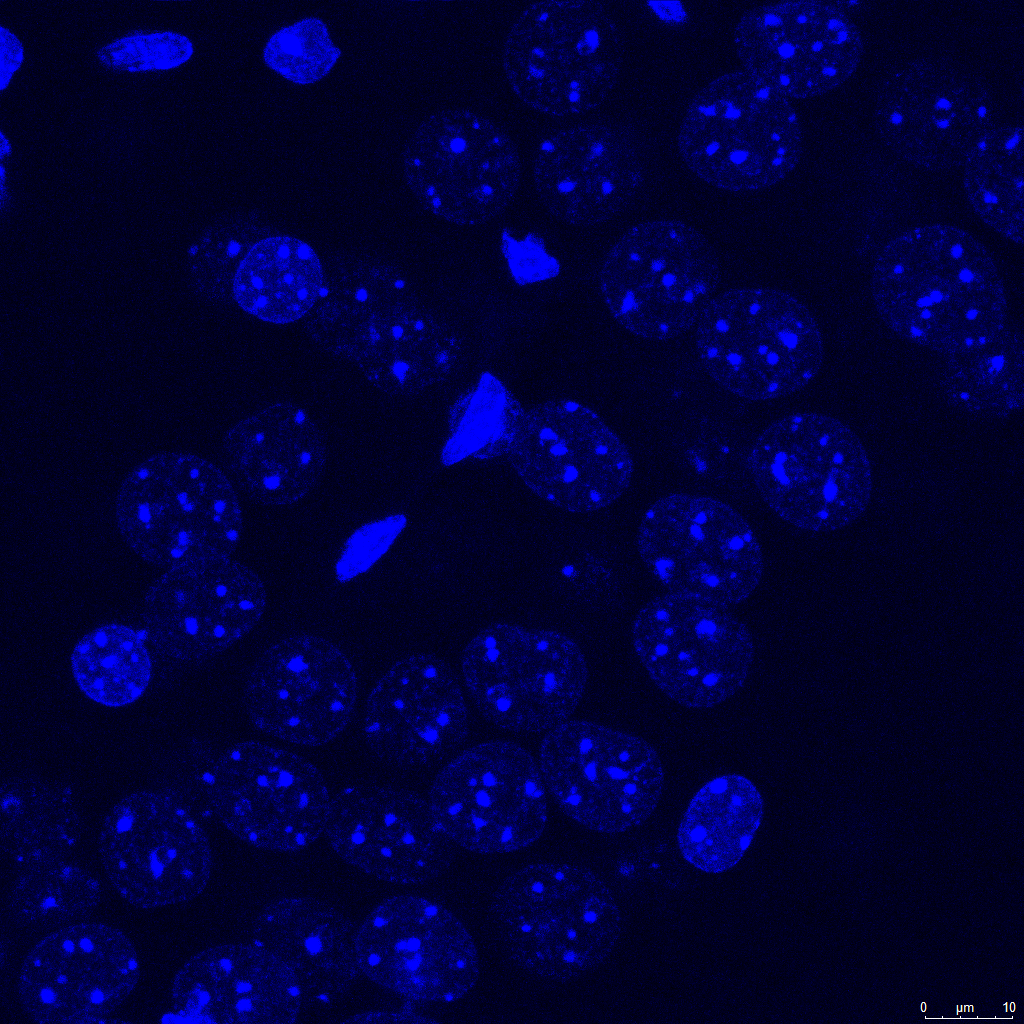

Supplement: Supplementary file 15 — Figure EV3 Source Data [file 44319_2024_218_MOESM15_ESM.zip › Figure EV3/3A/A162p19dKO_A162p19dKO, S3 CA2-3, GFAP488-G, IMPDH555-R, NeuN633-W, ZS 40xZF2,5-I_ch00.tif]

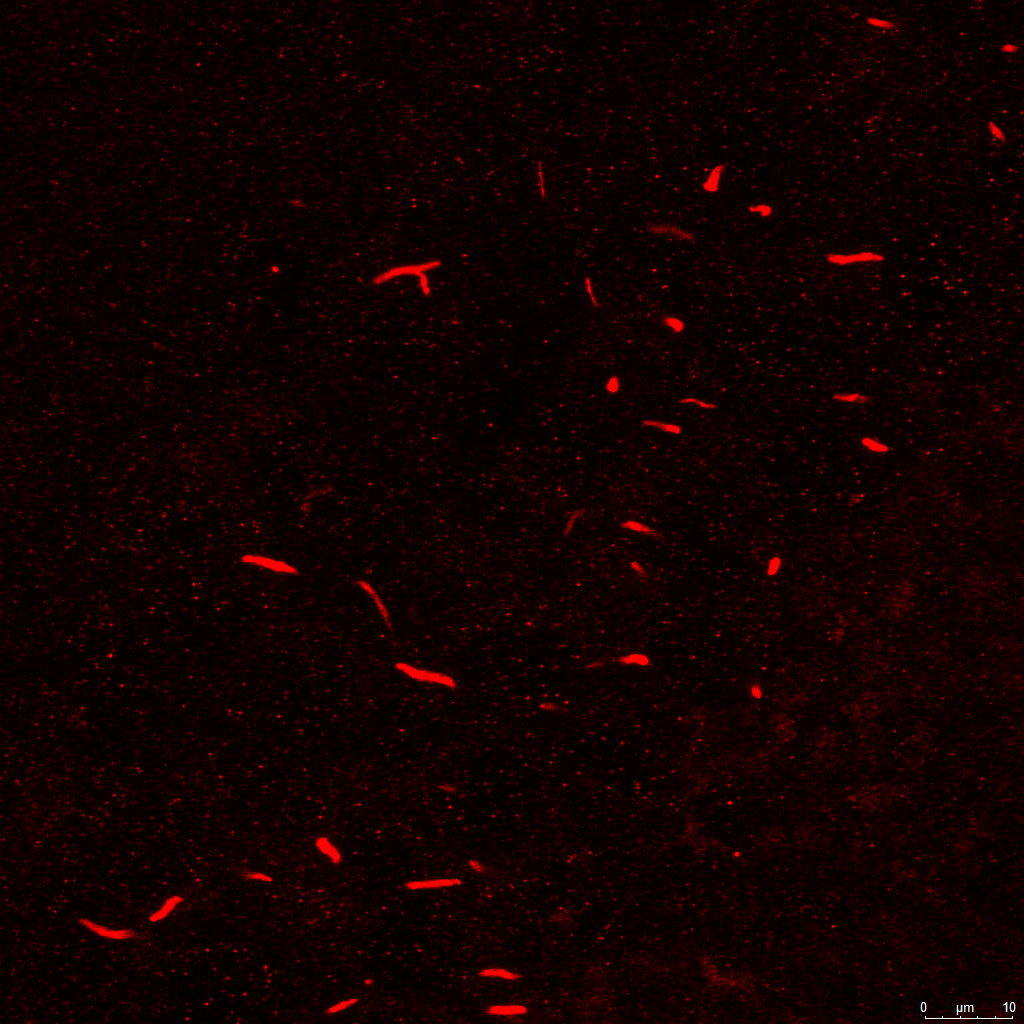

Supplement: Supplementary file 15 — Figure EV3 Source Data [file 44319_2024_218_MOESM15_ESM.zip › Figure EV3/3A/A162p19dKO_A162p19dKO, S3 CA2-3, GFAP488-G, IMPDH555-R, NeuN633-W, ZS 40xZF2,5-I_ch02.tif]

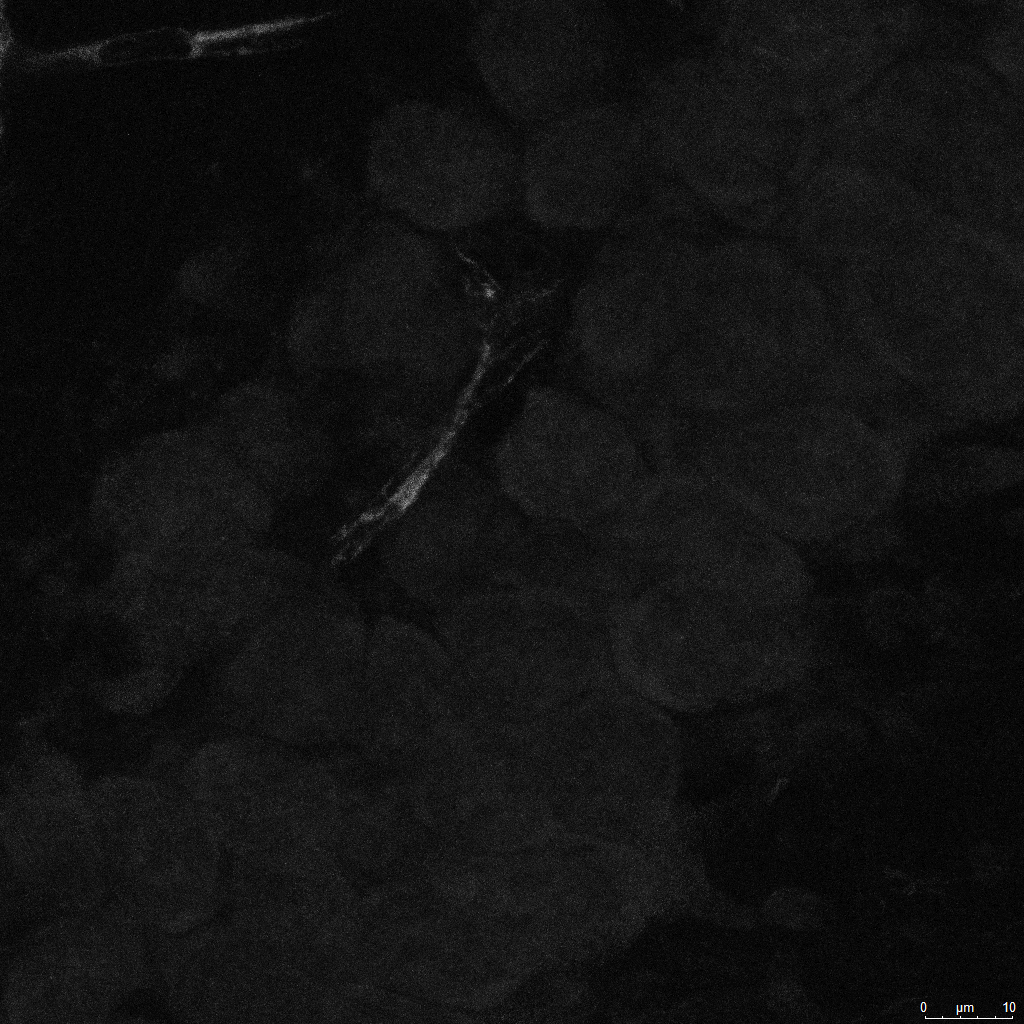

Supplement: Supplementary file 15 — Figure EV3 Source Data [file 44319_2024_218_MOESM15_ESM.zip › Figure EV3/3A/A162p19dKO_A162p19dKO, S3 CA2-3, GFAP488-G, IMPDH555-R, NeuN633-W, ZS 40xZF2,5-I_ch03.tif]

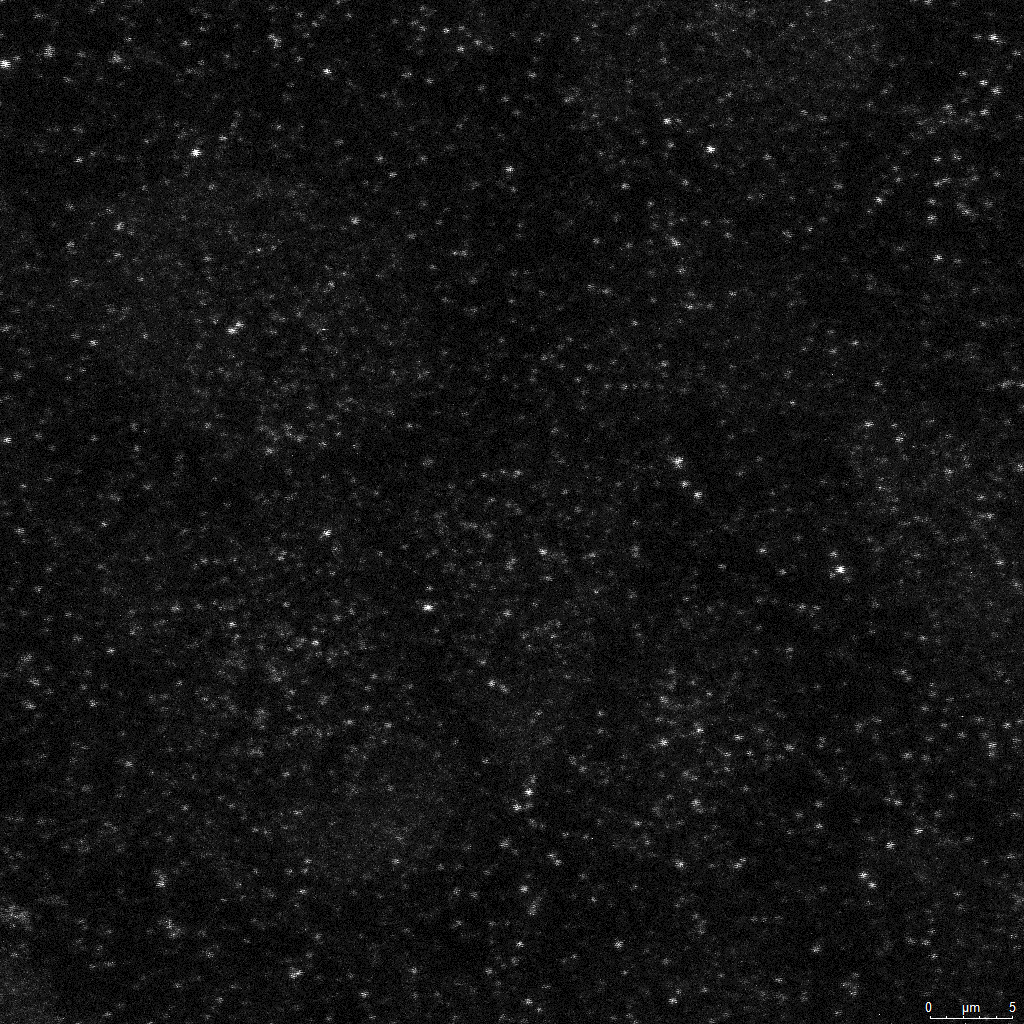

Supplement: Supplementary file 15 — Figure EV3 Source Data [file 44319_2024_218_MOESM15_ESM.zip › Figure EV3/3B/p10/dKO 898_dKO p10 898, Ctx, IMPDH2 488W, NEUN 568G, ZS 63X-2C_ch01.tif]

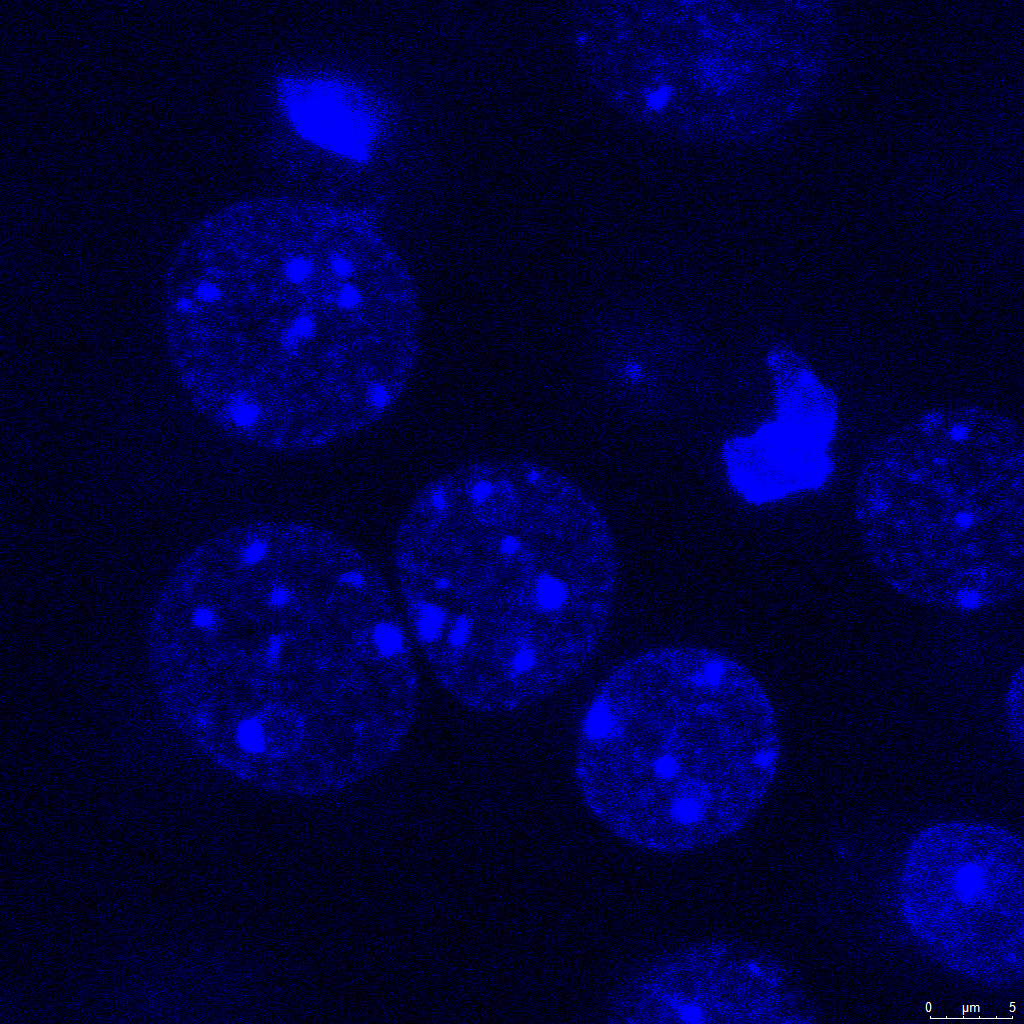

Supplement: Supplementary file 15 — Figure EV3 Source Data [file 44319_2024_218_MOESM15_ESM.zip › Figure EV3/3B/p10/dKO 898_dKO p10 898, Ctx, IMPDH2 488W, NEUN 568G, ZS 63X-2C_ch00.tif]

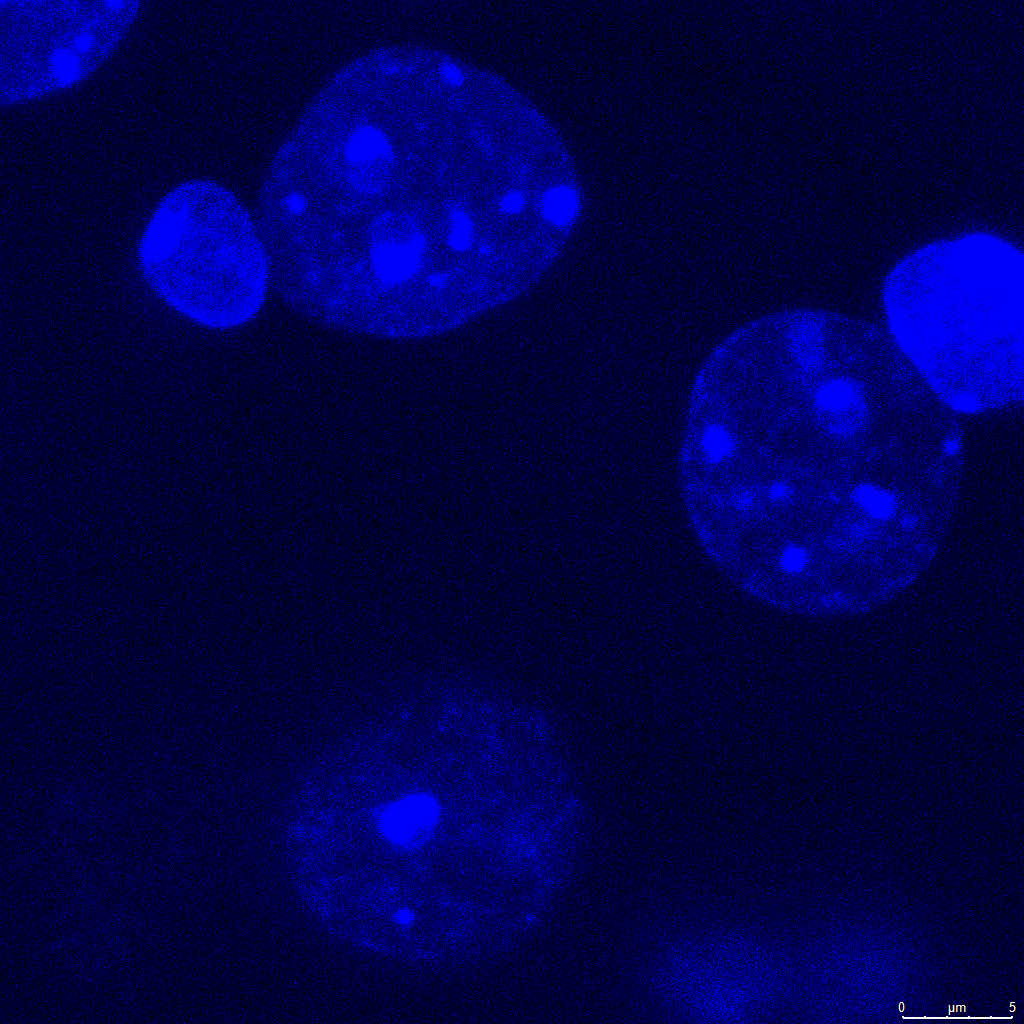

Supplement: Supplementary file 15 — Figure EV3 Source Data [file 44319_2024_218_MOESM15_ESM.zip › Figure EV3/3B/p20/p20 dKO 900_dKO p20 900, Ctx, IMPDH2 488W, NEUN 568G, ZS 63X-5C_ch00.tif]

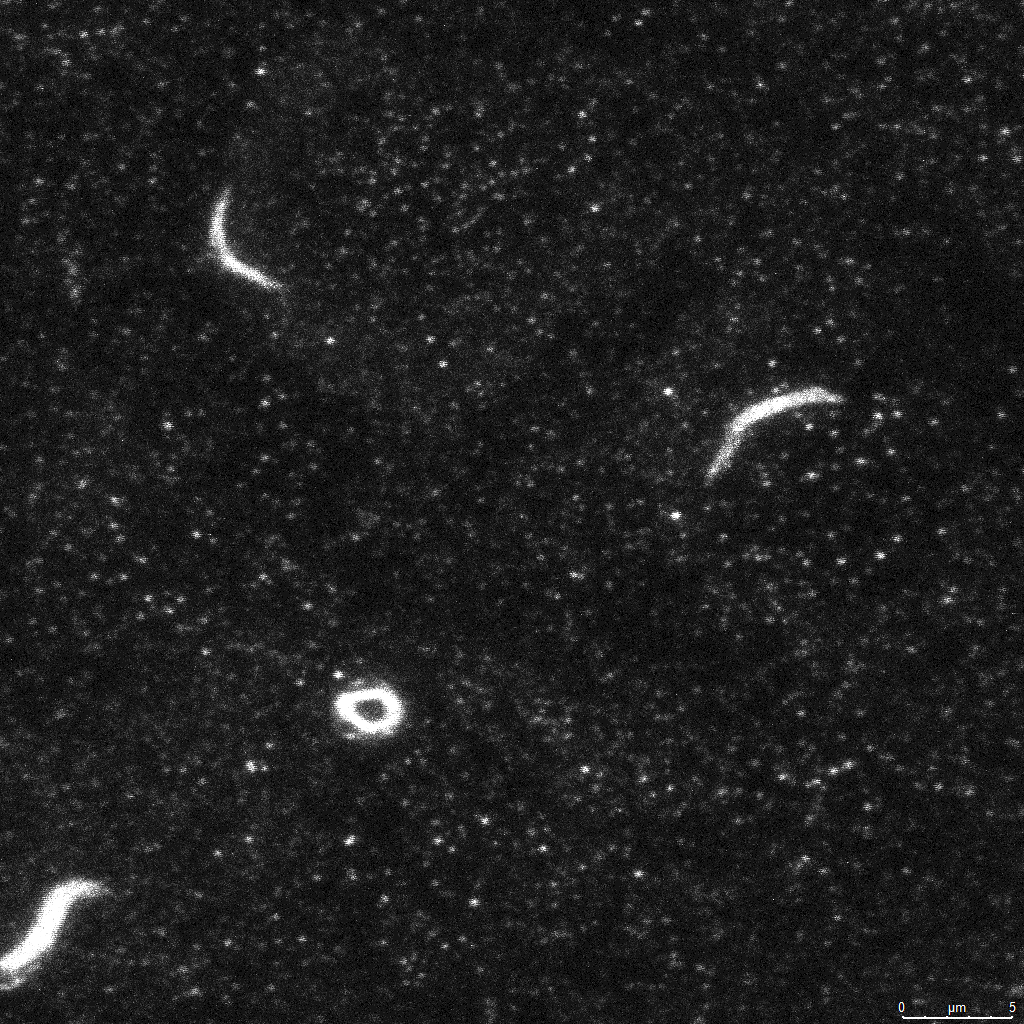

Supplement: Supplementary file 15 — Figure EV3 Source Data [file 44319_2024_218_MOESM15_ESM.zip › Figure EV3/3B/p20/p20 dKO 900_dKO p20 900, Ctx, IMPDH2 488W, NEUN 568G, ZS 63X-5C_ch01.tif]

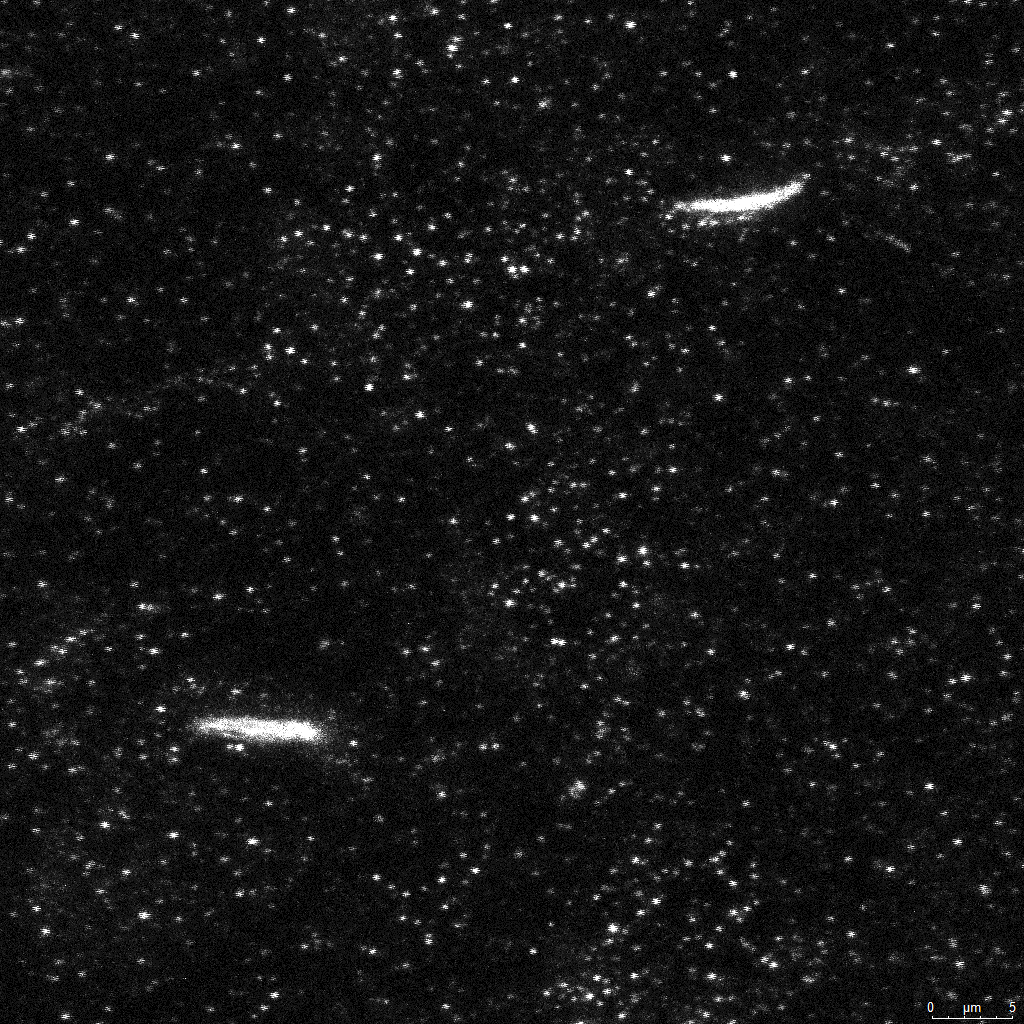

Supplement: Supplementary file 15 — Figure EV3 Source Data [file 44319_2024_218_MOESM15_ESM.zip › Figure EV3/3B/p15/dKO 896_dKO p15 896, Ctx, IMPDH2 488W, NEUN 568G, ZS 63X-1C_ch01.tif]

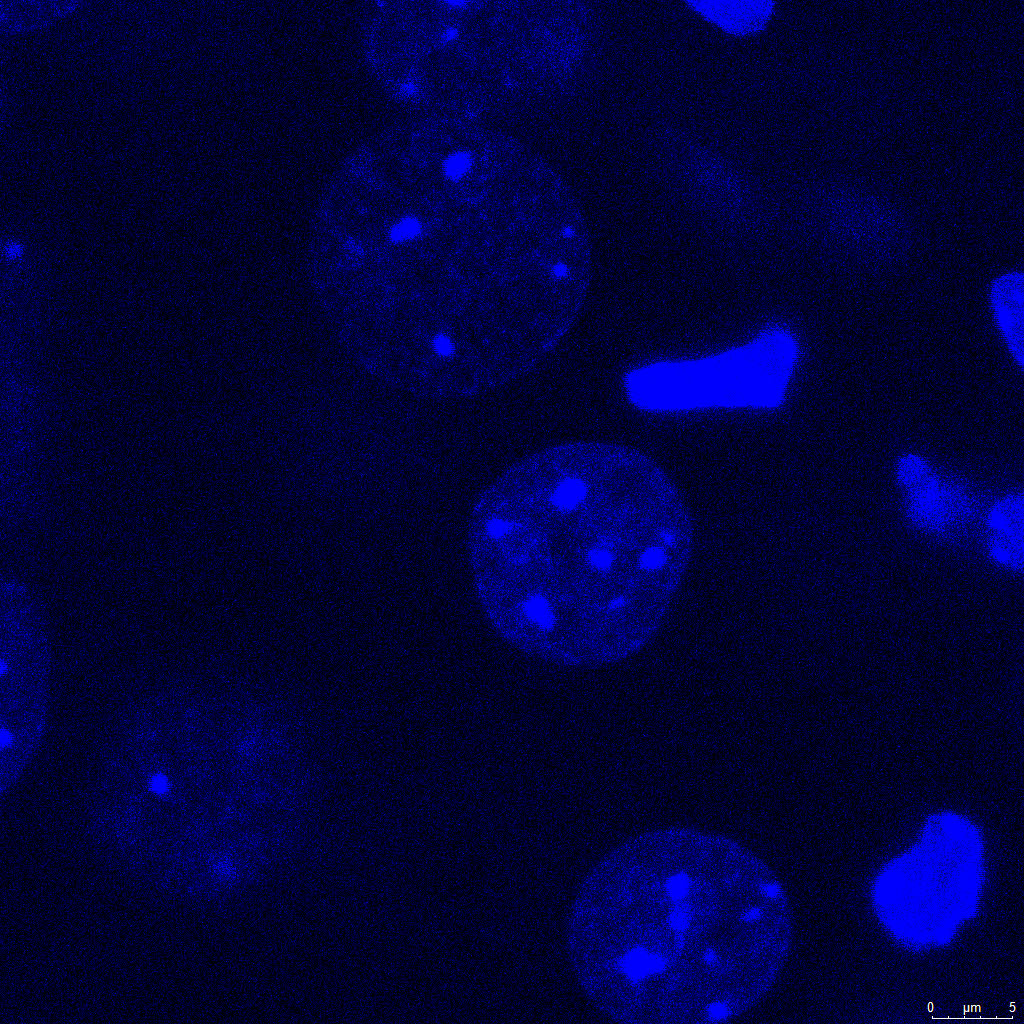

Supplement: Supplementary file 15 — Figure EV3 Source Data [file 44319_2024_218_MOESM15_ESM.zip › Figure EV3/3B/p15/dKO 896_dKO p15 896, Ctx, IMPDH2 488W, NEUN 568G, ZS 63X-1C_ch00.tif]

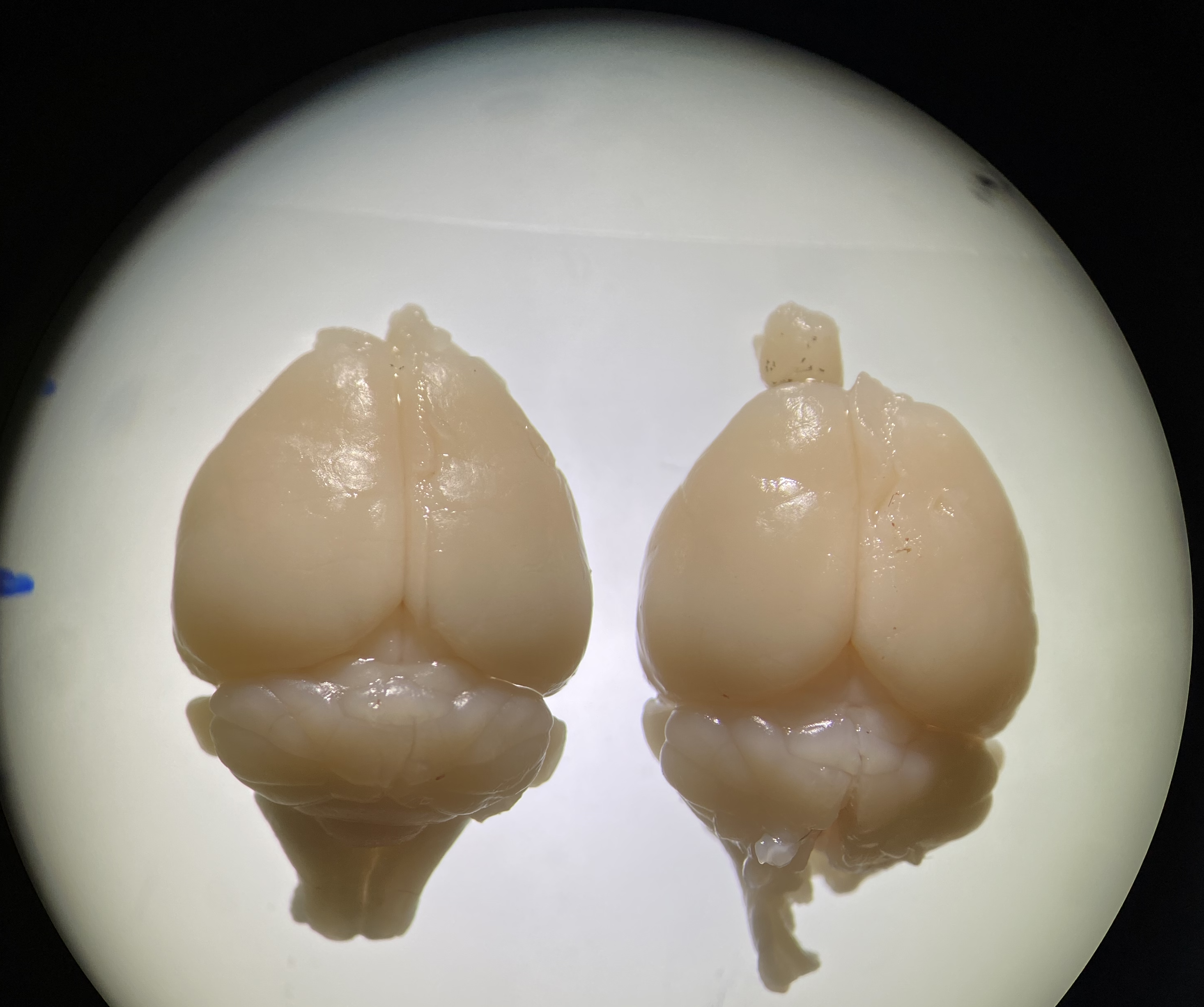

Supplement: Supplementary file 16 — Figure EV4 Source Data [file 44319_2024_218_MOESM16_ESM.zip › Figure EV4/4E/24w-1.tiff]

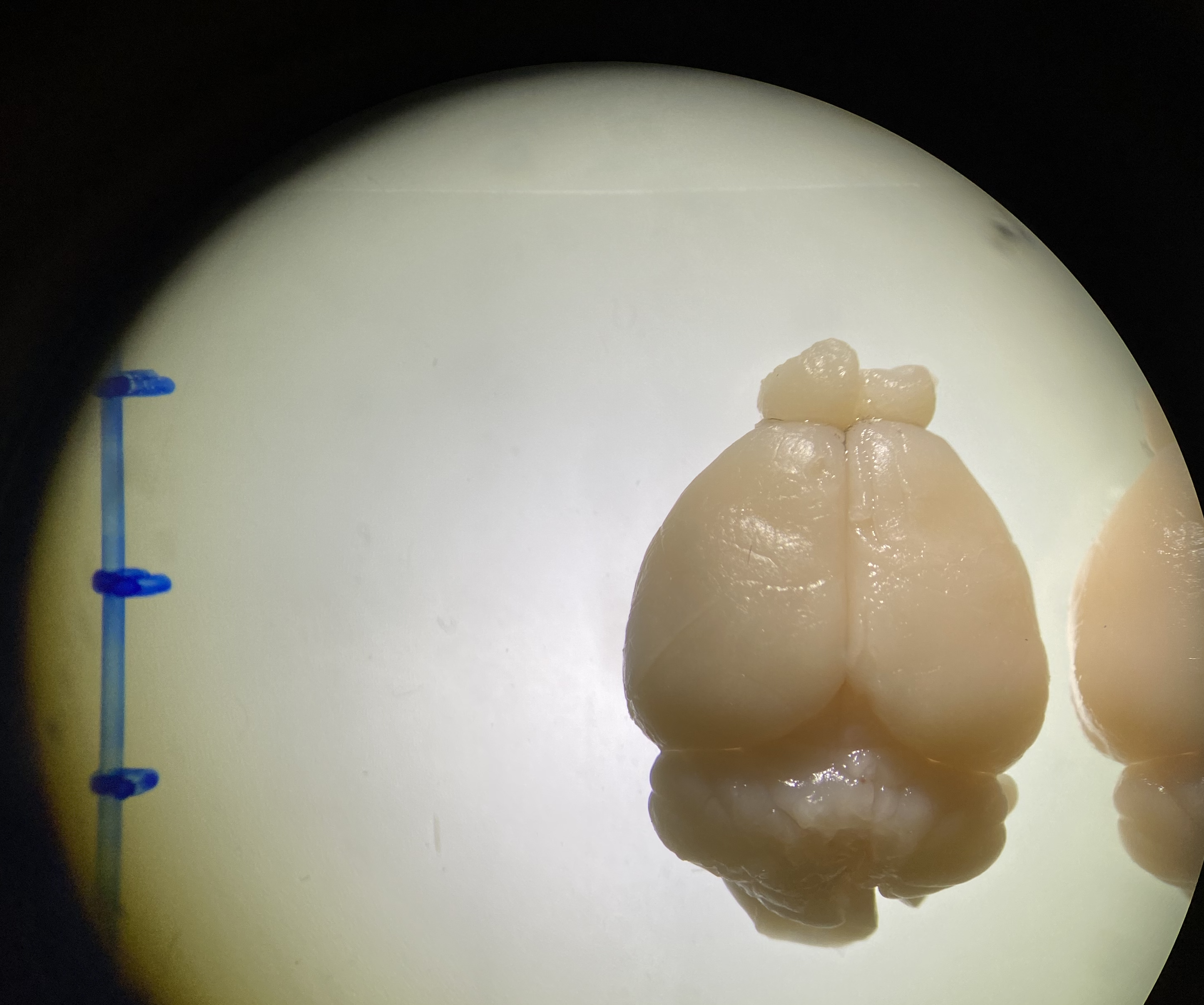

Supplement: Supplementary file 16 — Figure EV4 Source Data [file 44319_2024_218_MOESM16_ESM.zip › Figure EV4/4E/8w-2.tiff]

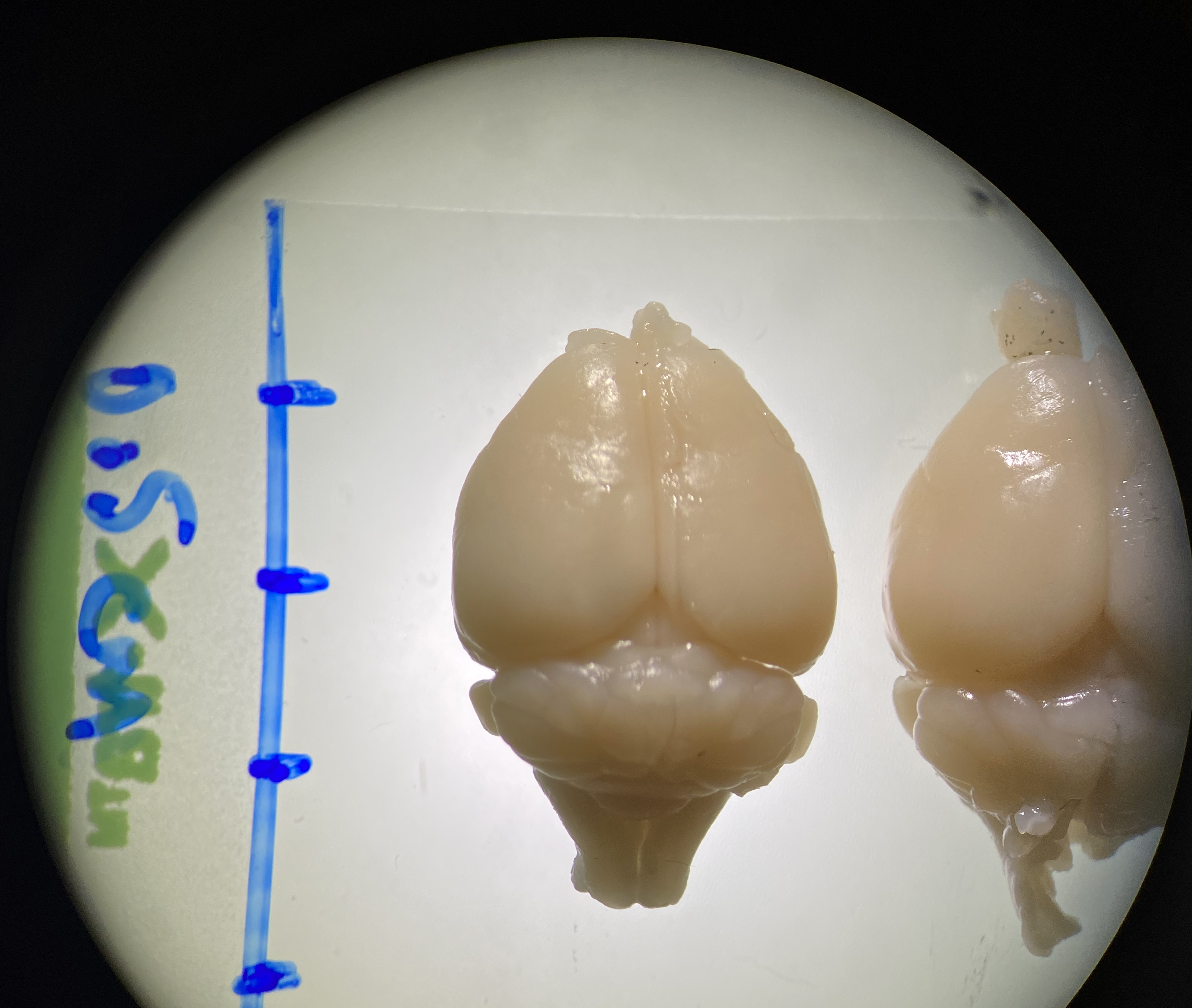

Supplement: Supplementary file 16 — Figure EV4 Source Data [file 44319_2024_218_MOESM16_ESM.zip › Figure EV4/4E/24w-2.tiff]

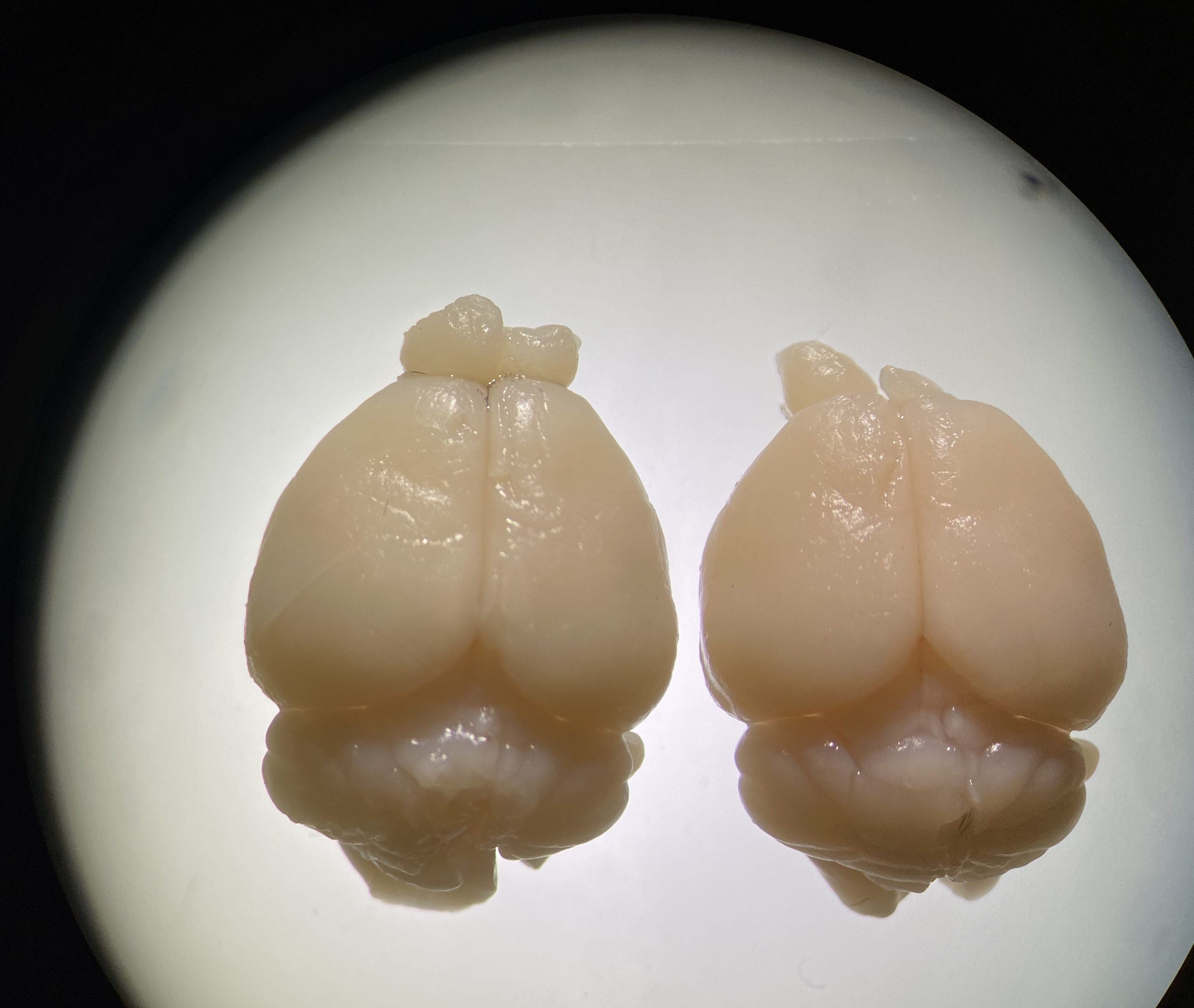

Supplement: Supplementary file 16 — Figure EV4 Source Data [file 44319_2024_218_MOESM16_ESM.zip › Figure EV4/4E/8w-1.png]

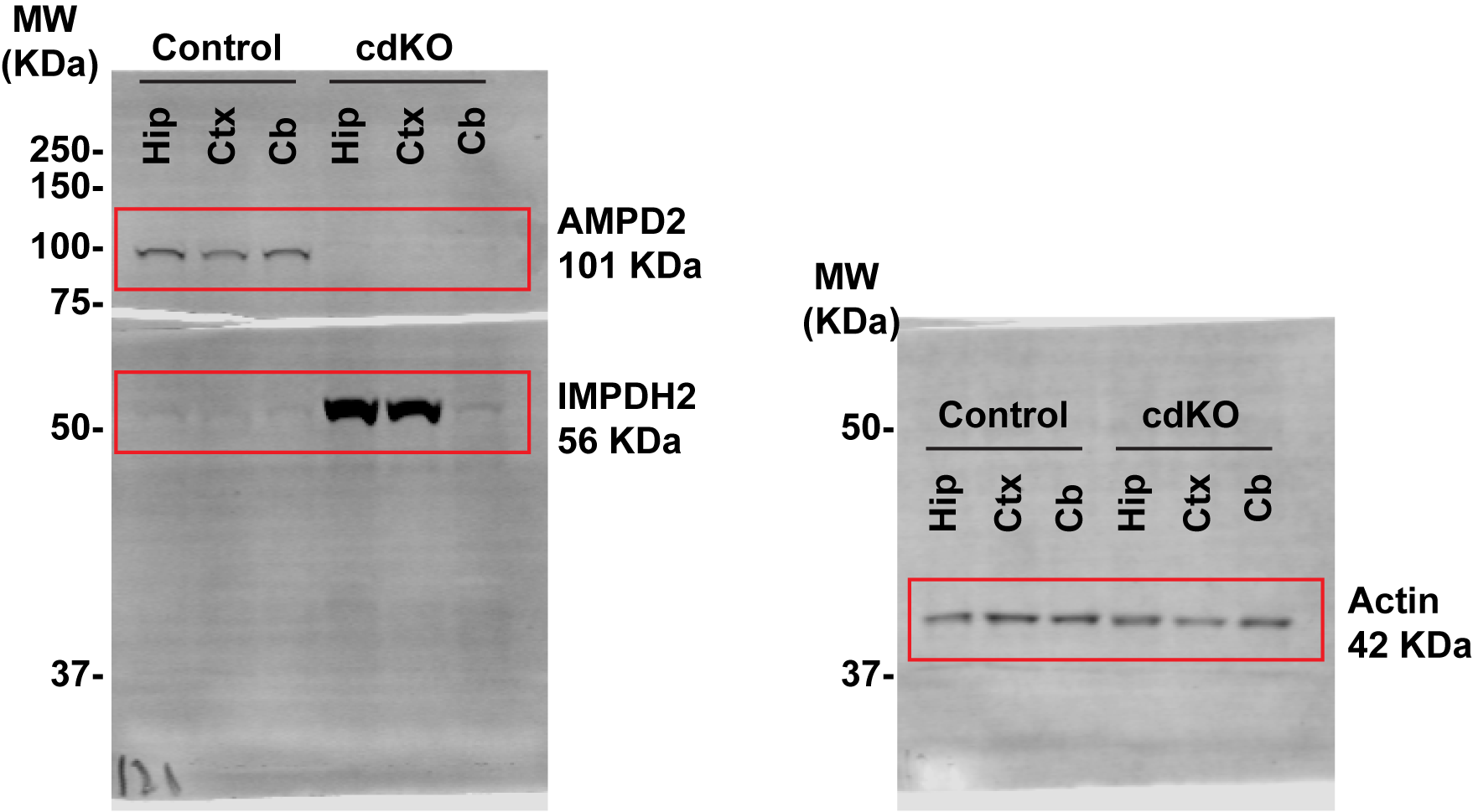

Supplement: Supplementary file 16 — Figure EV4 Source Data [file 44319_2024_218_MOESM16_ESM.zip › Figure EV4/4B/WB EV4B.tif]

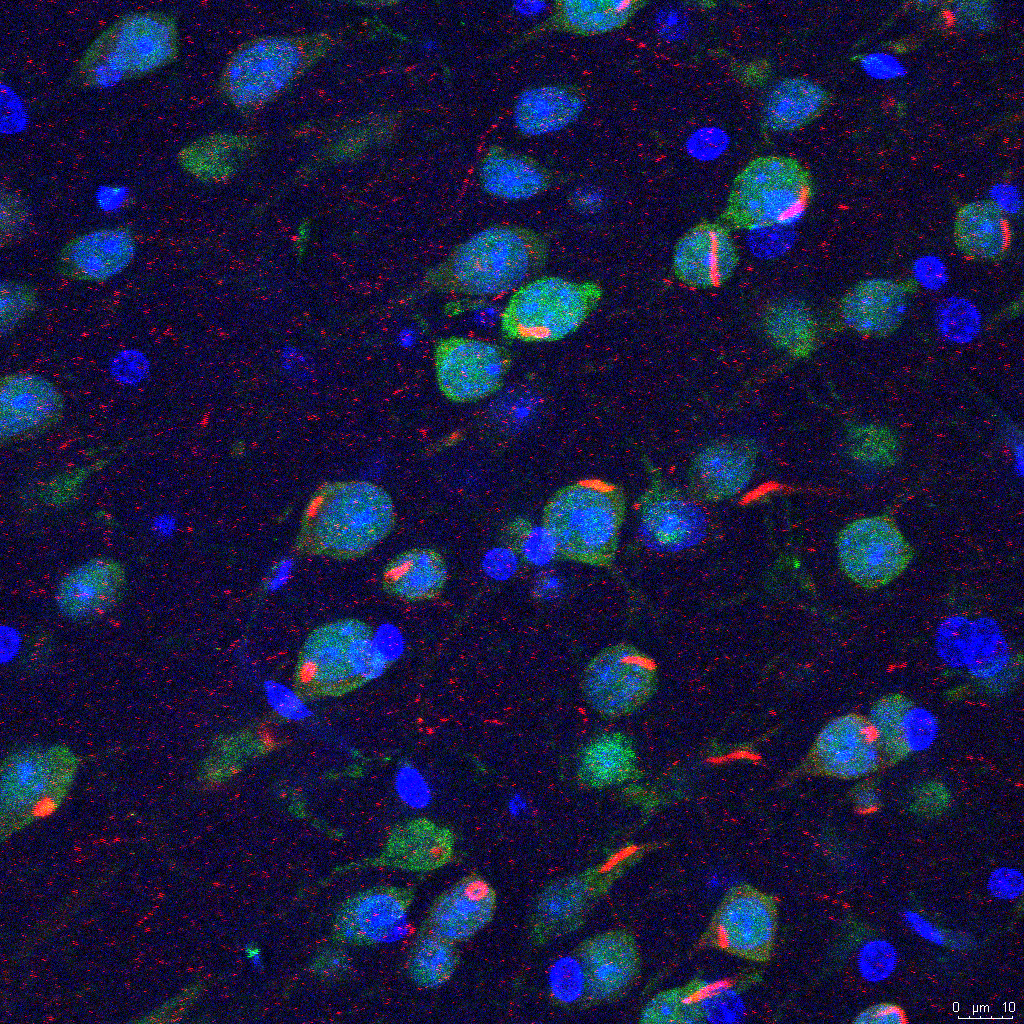

Supplement: Supplementary file 16 — Figure EV4 Source Data [file 44319_2024_218_MOESM16_ESM.zip › Figure EV4/4H/cdKO/cdKO 367_cdKO 6mo 367, Ctx, NeuN 488G, IMPDH2 555R, ZS 63x-1A.tif]

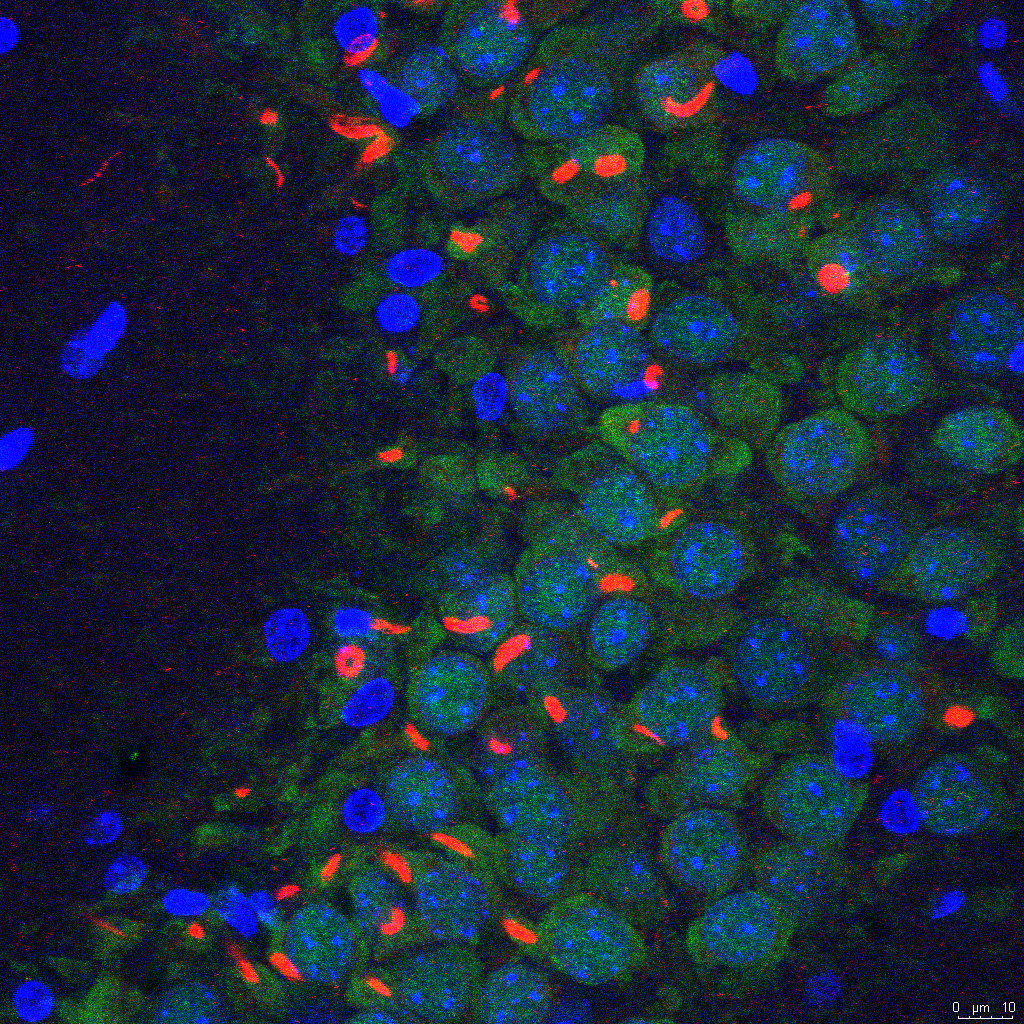

Supplement: Supplementary file 16 — Figure EV4 Source Data [file 44319_2024_218_MOESM16_ESM.zip › Figure EV4/4H/cdKO/cdKO 367_cdKO 6mo 367, CA3, NeuN 488G, IMPDH2 555R, ZS 63x-1D.tif]

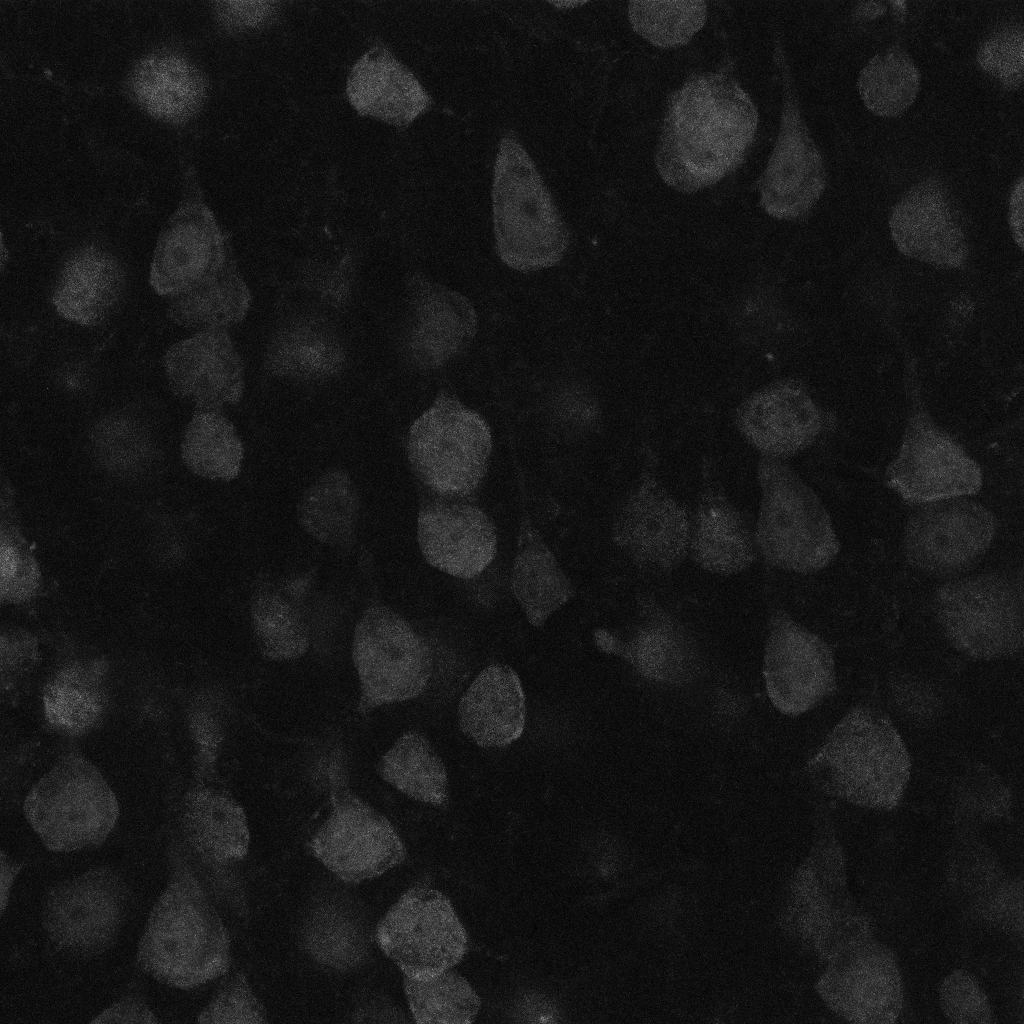

Supplement: Supplementary file 16 — Figure EV4 Source Data [file 44319_2024_218_MOESM16_ESM.zip › Figure EV4/4I/cdKO 5w/C4-IMPDH2_RR_Acc_63X_5w_CKO_Rep_IMPDH2_555_NeuN_633_GFAP_488_1.5.20.lif - 917_KFC_ant_hip_SS_Ctx_L5_Processed001_2.tif]

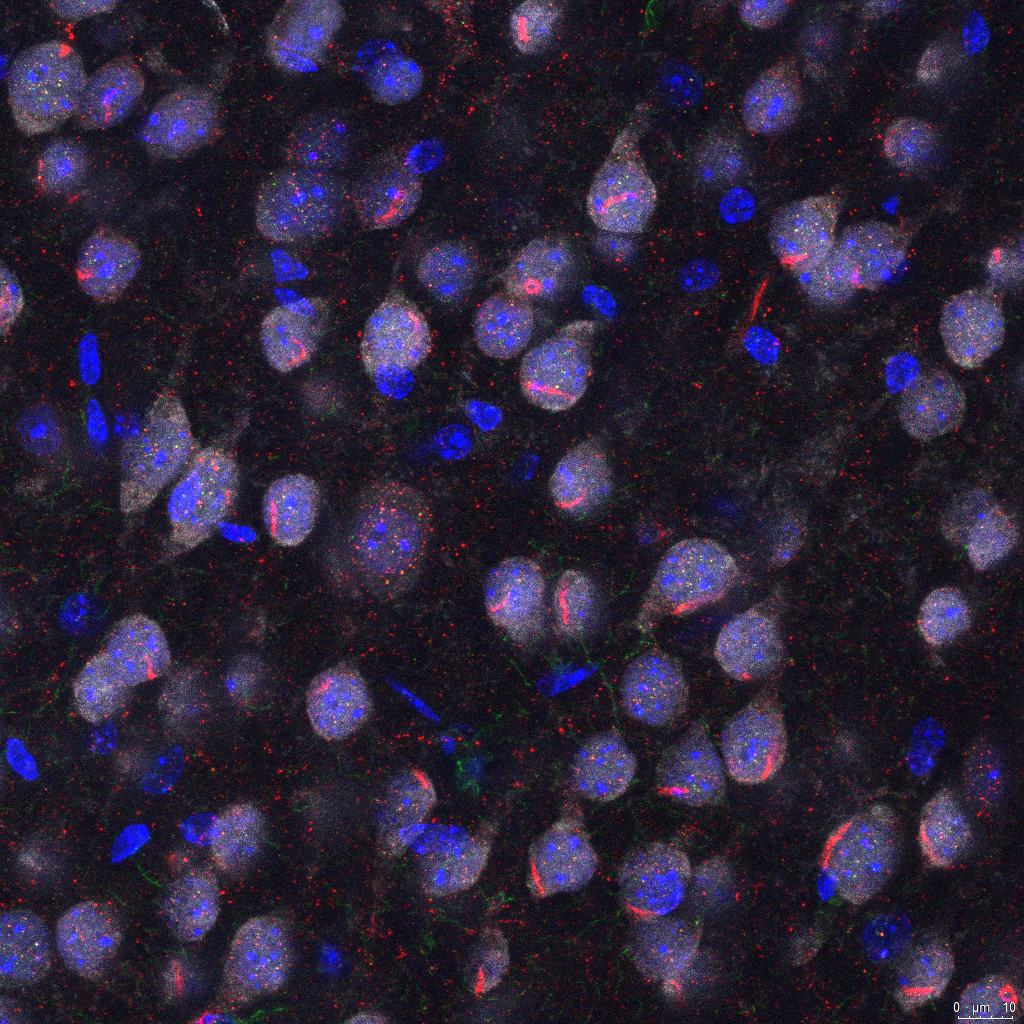

Supplement: Supplementary file 16 — Figure EV4 Source Data [file 44319_2024_218_MOESM16_ESM.zip › Figure EV4/4I/cdKO 8w/Hip_IMPDH2_RR_Accumulation_CKO_n=1_63X_2mo_12.14.20_880_KFC_ant_hip_MCtx_L5_rep_opt_Processed001.tif]

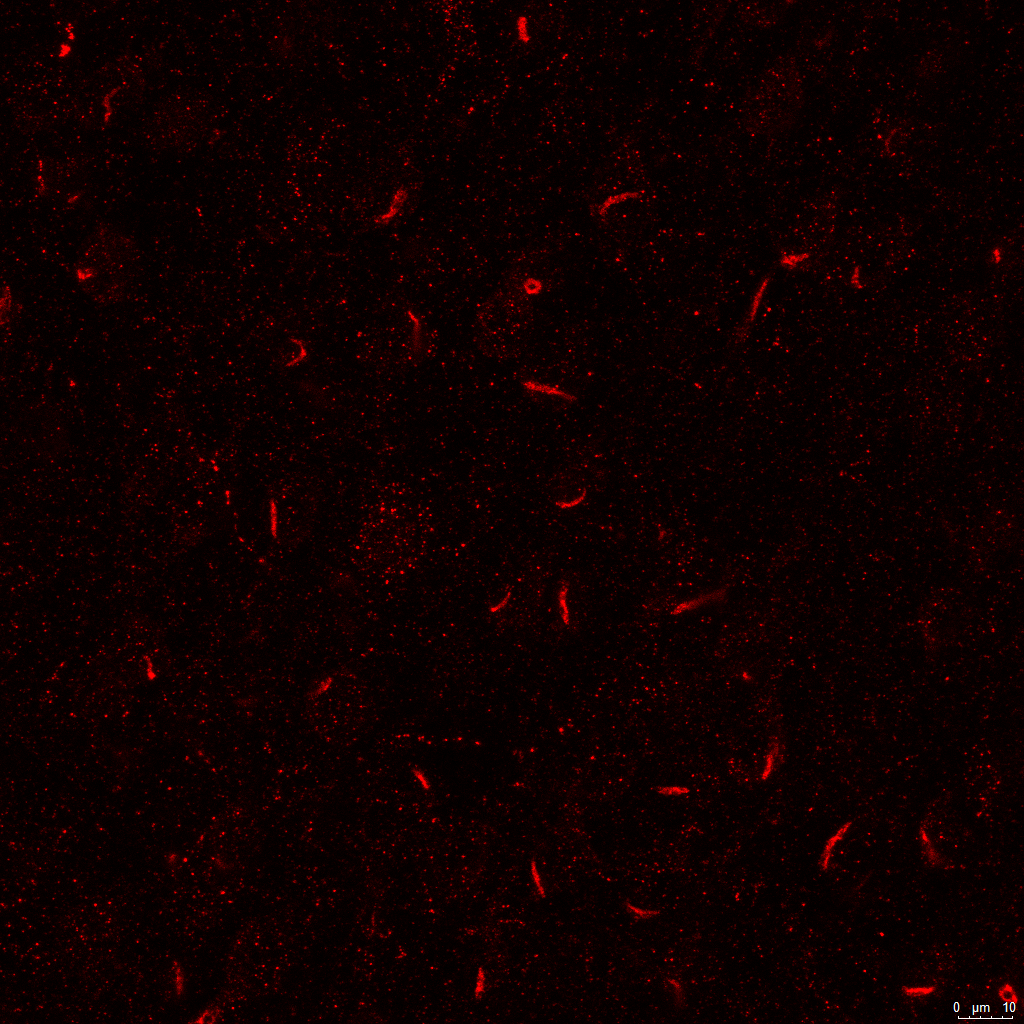

Supplement: Supplementary file 16 — Figure EV4 Source Data [file 44319_2024_218_MOESM16_ESM.zip › Figure EV4/4I/cdKO 8w/Hip_IMPDH2_RR_Accumulation_CKO_n=1_63X_2mo_12.14.20_880_KFC_ant_hip_MCtx_L5_rep_opt_Processed001_ch02.tif]

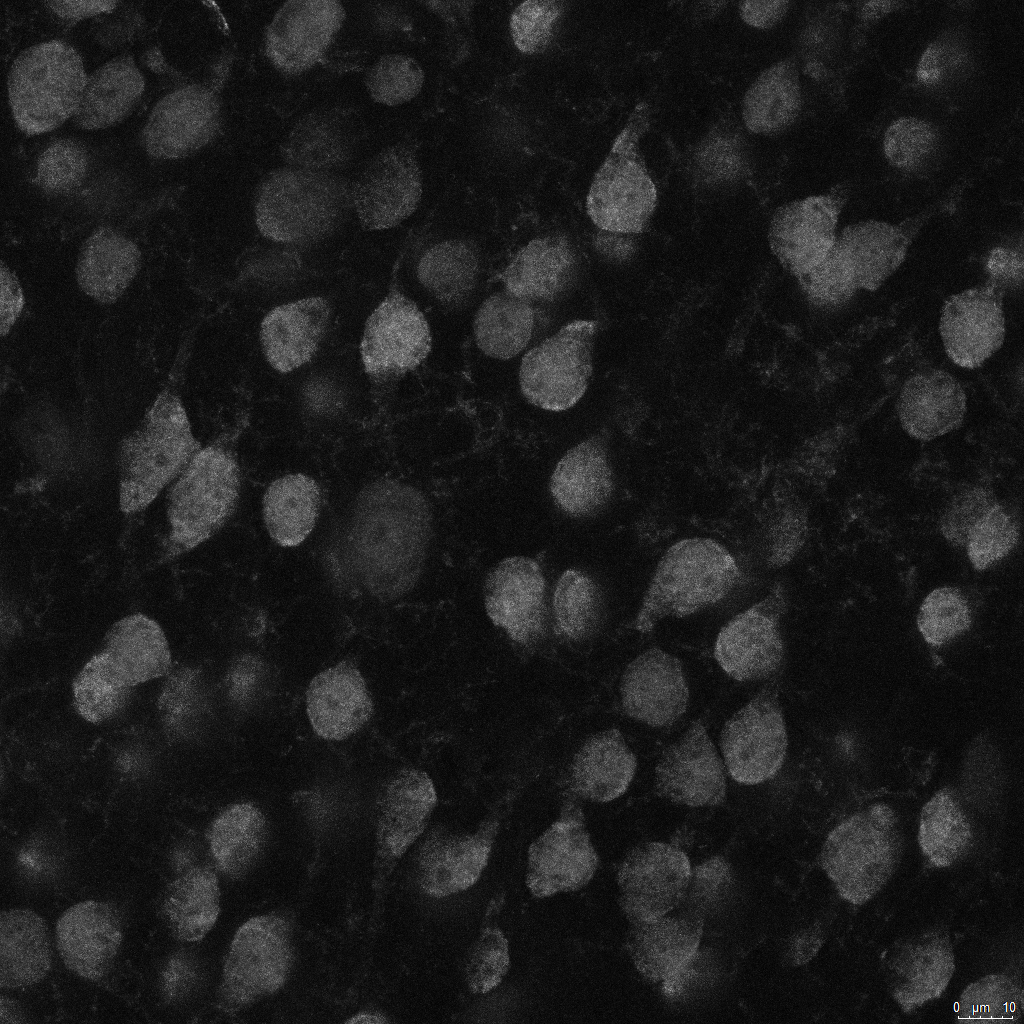

Supplement: Supplementary file 16 — Figure EV4 Source Data [file 44319_2024_218_MOESM16_ESM.zip › Figure EV4/4I/cdKO 8w/Hip_IMPDH2_RR_Accumulation_CKO_n=1_63X_2mo_12.14.20_880_KFC_ant_hip_MCtx_L5_rep_opt_Processed001_ch03.tif]

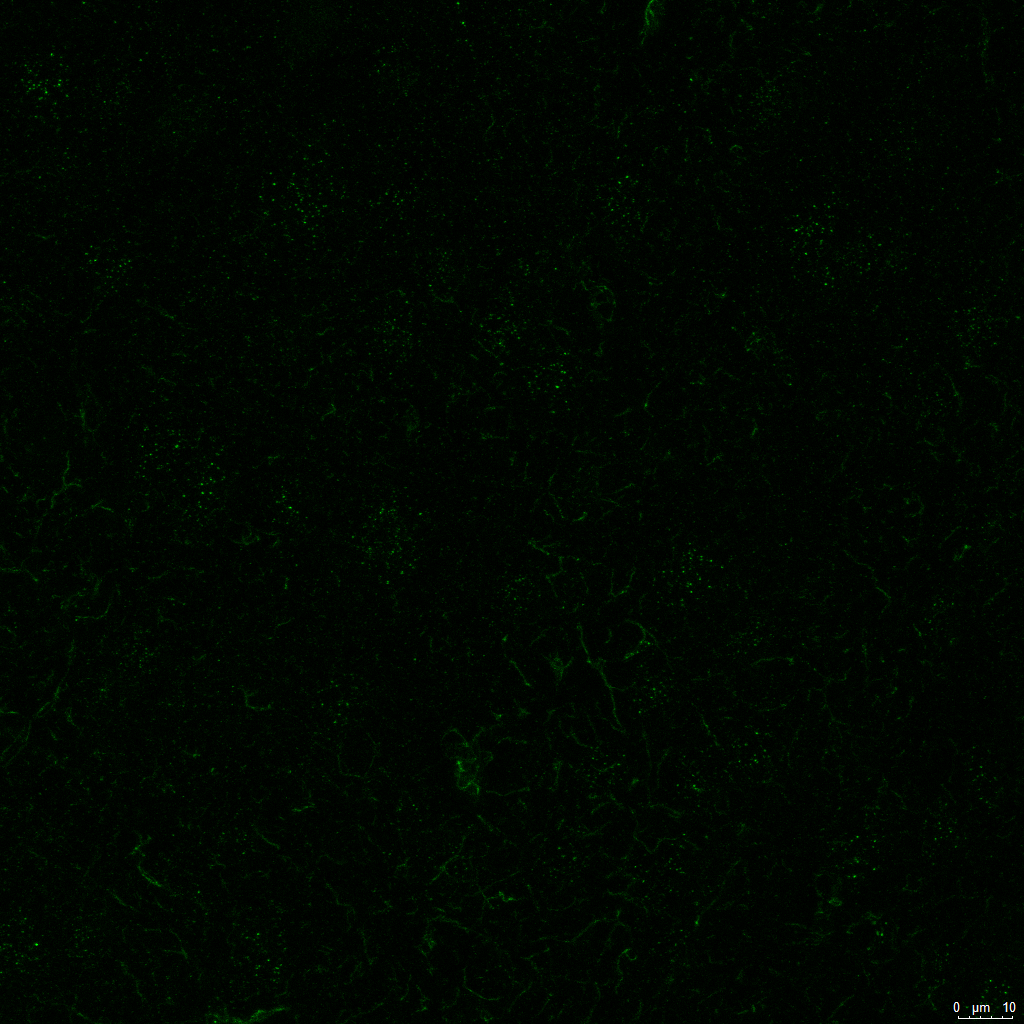

Supplement: Supplementary file 16 — Figure EV4 Source Data [file 44319_2024_218_MOESM16_ESM.zip › Figure EV4/4I/cdKO 8w/Hip_IMPDH2_RR_Accumulation_CKO_n=1_63X_2mo_12.14.20_880_KFC_ant_hip_MCtx_L5_rep_opt_Processed001_ch01.tif]

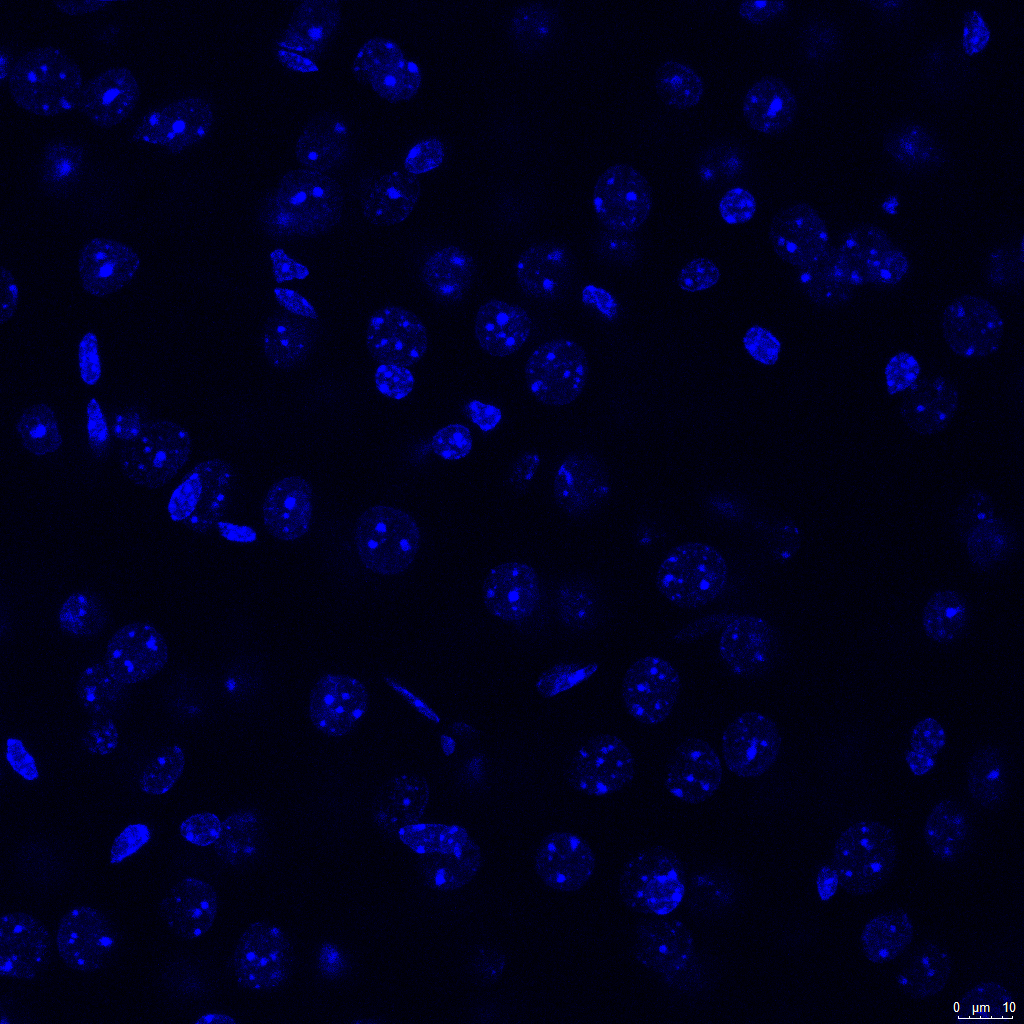

Supplement: Supplementary file 16 — Figure EV4 Source Data [file 44319_2024_218_MOESM16_ESM.zip › Figure EV4/4I/cdKO 8w/Hip_IMPDH2_RR_Accumulation_CKO_n=1_63X_2mo_12.14.20_880_KFC_ant_hip_MCtx_L5_rep_opt_Processed001_ch00.tif]

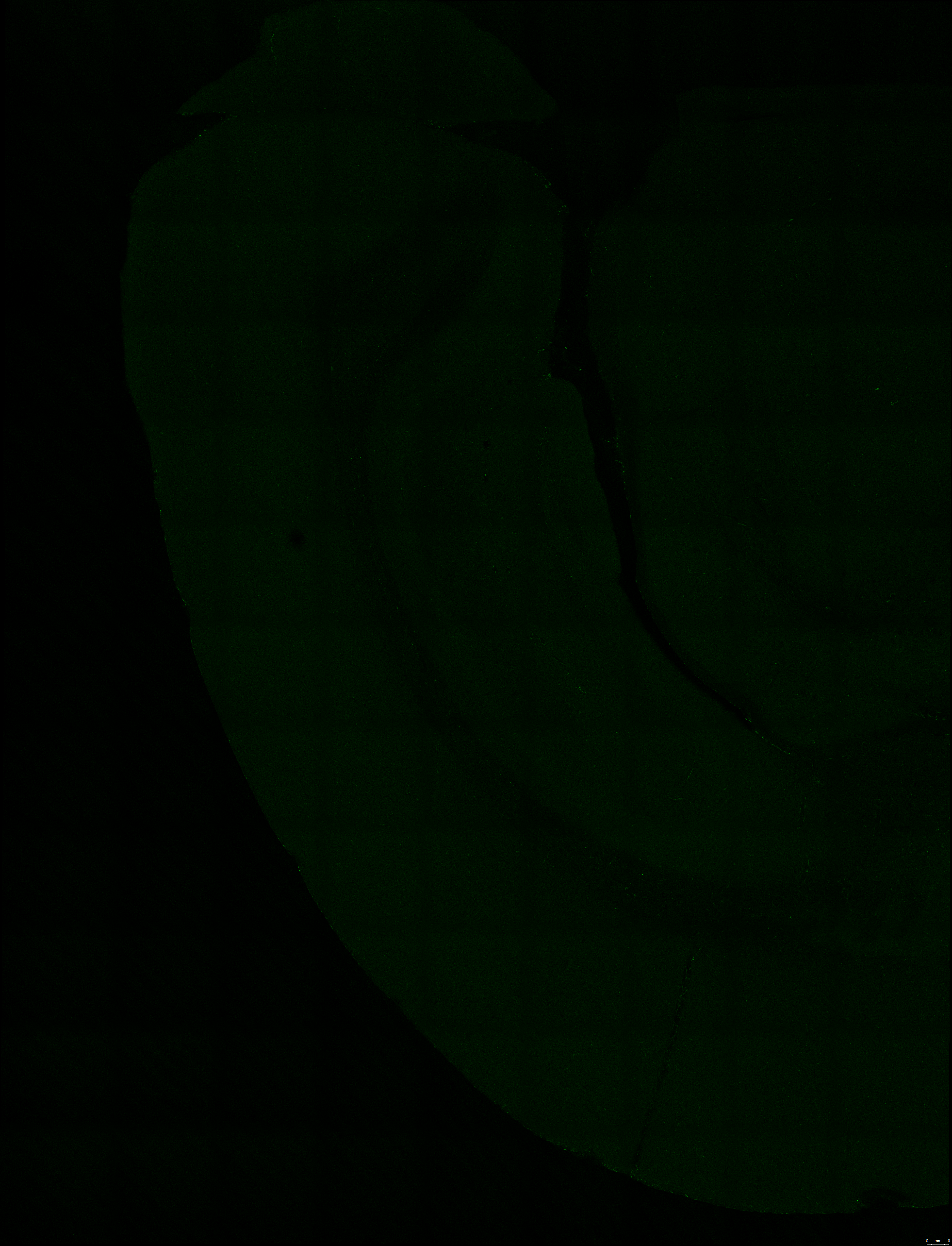

Supplement: Supplementary file 16 — Figure EV4 Source Data [file 44319_2024_218_MOESM16_ESM.zip › Figure EV4/4G/Ctrl 8w/Post_Hip_2mo_Cont_TileScan_001_Merging_Processed001_ch01.tif]

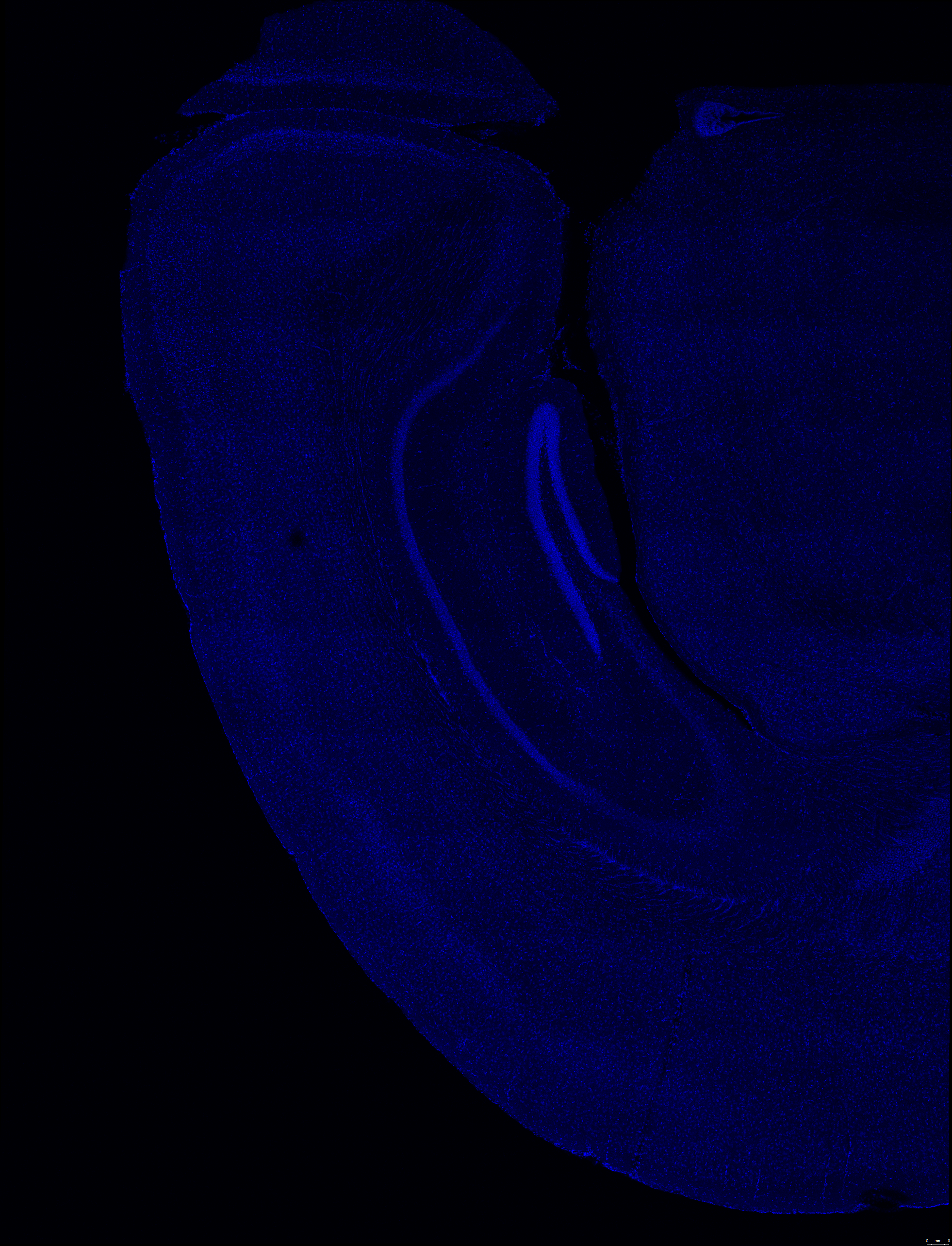

Supplement: Supplementary file 16 — Figure EV4 Source Data [file 44319_2024_218_MOESM16_ESM.zip › Figure EV4/4G/Ctrl 8w/Post_Hip_2mo_Cont_TileScan_001_Merging_Processed001_ch00.tif]

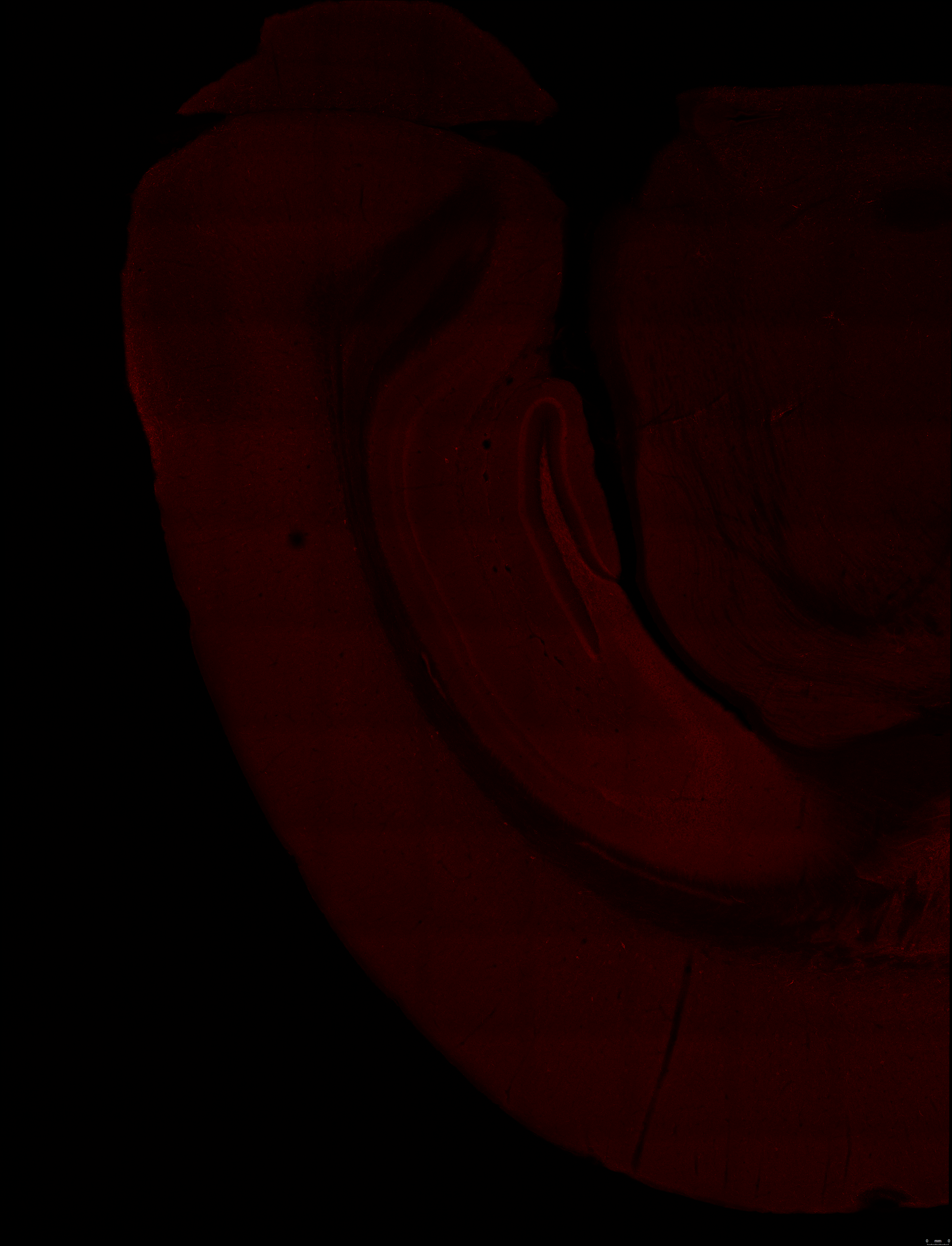

Supplement: Supplementary file 16 — Figure EV4 Source Data [file 44319_2024_218_MOESM16_ESM.zip › Figure EV4/4G/Ctrl 8w/Post_Hip_2mo_Cont_TileScan_001_Merging_Processed001_ch02.tif]

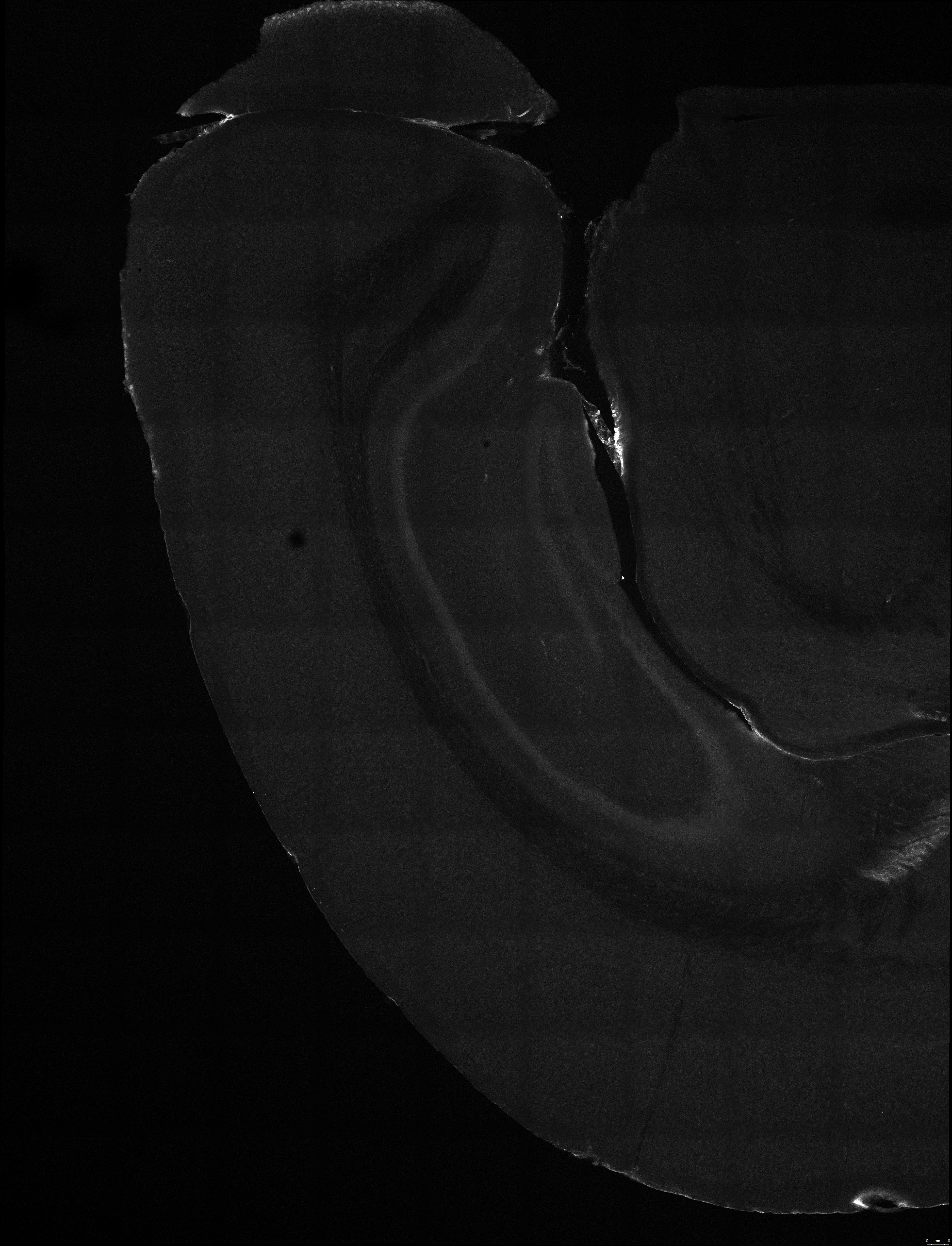

Supplement: Supplementary file 16 — Figure EV4 Source Data [file 44319_2024_218_MOESM16_ESM.zip › Figure EV4/4G/Ctrl 8w/Post_Hip_2mo_Cont_TileScan_001_Merging_Processed001_ch03.tif]

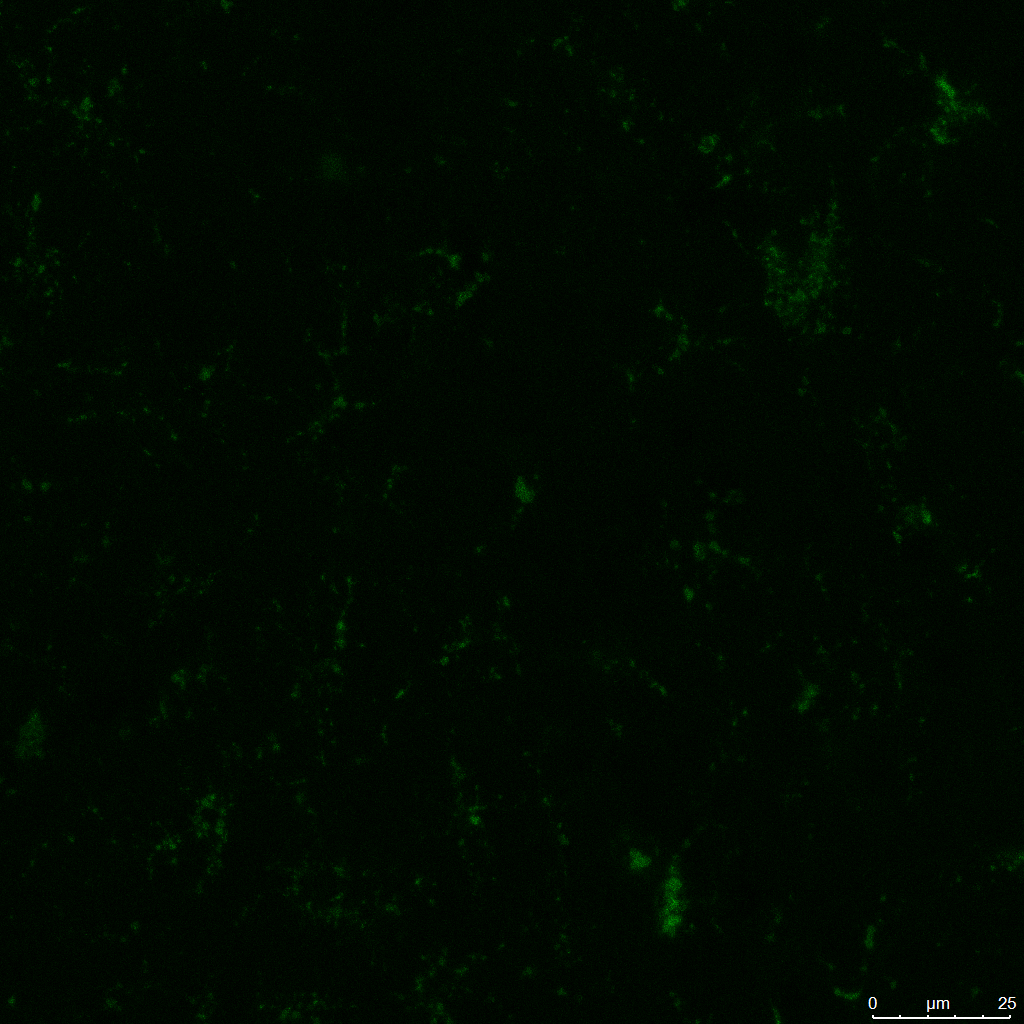

Supplement: Supplementary file 16 — Figure EV4 Source Data [file 44319_2024_218_MOESM16_ESM.zip › Figure EV4/4G/cdKO 8w/DG/2mo_CKO_post_IMPDH2_555_CD68_488_Casp7_633_5.3.21_KFC_post_Hip_DG_SB_Processed001_ch01.tif]

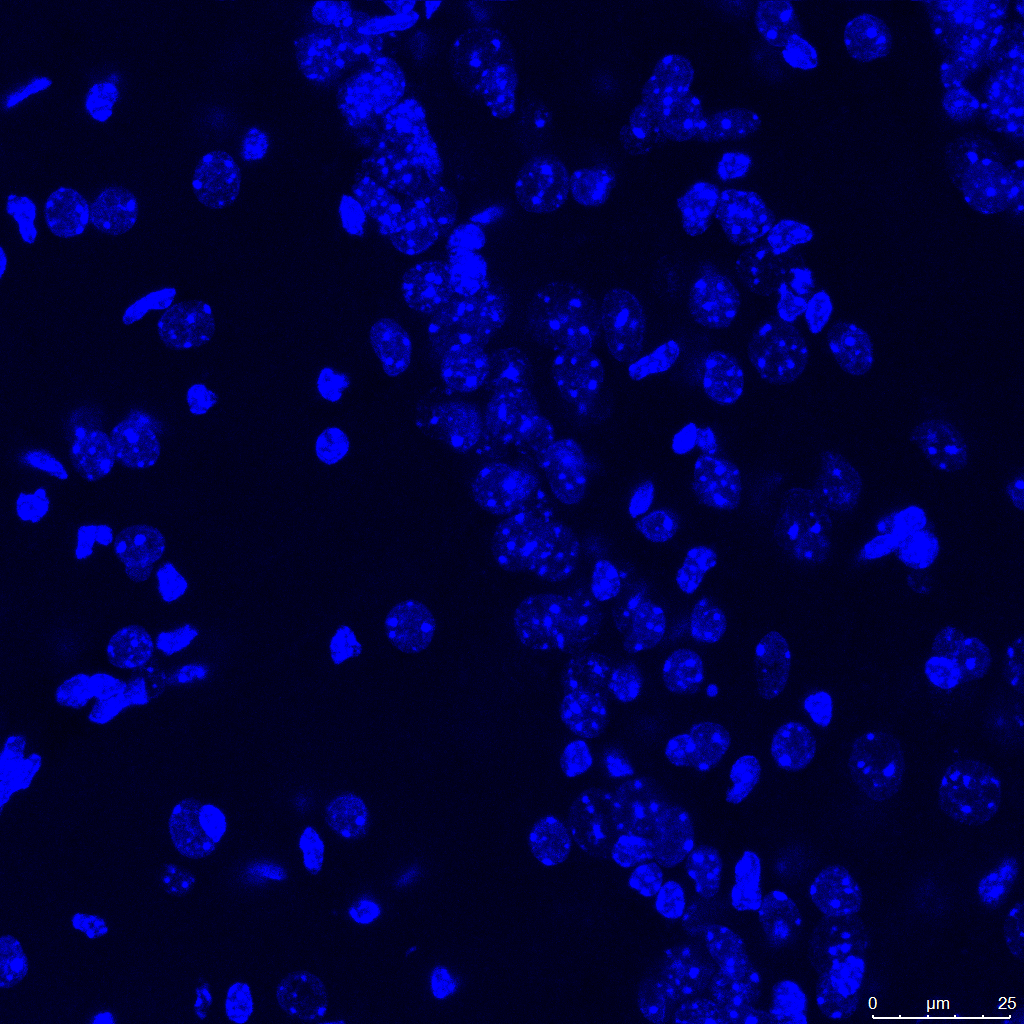

Supplement: Supplementary file 16 — Figure EV4 Source Data [file 44319_2024_218_MOESM16_ESM.zip › Figure EV4/4G/cdKO 8w/DG/2mo_CKO_post_IMPDH2_555_CD68_488_Casp7_633_5.3.21_KFC_post_Hip_DG_SB_Processed001_ch00.tif]

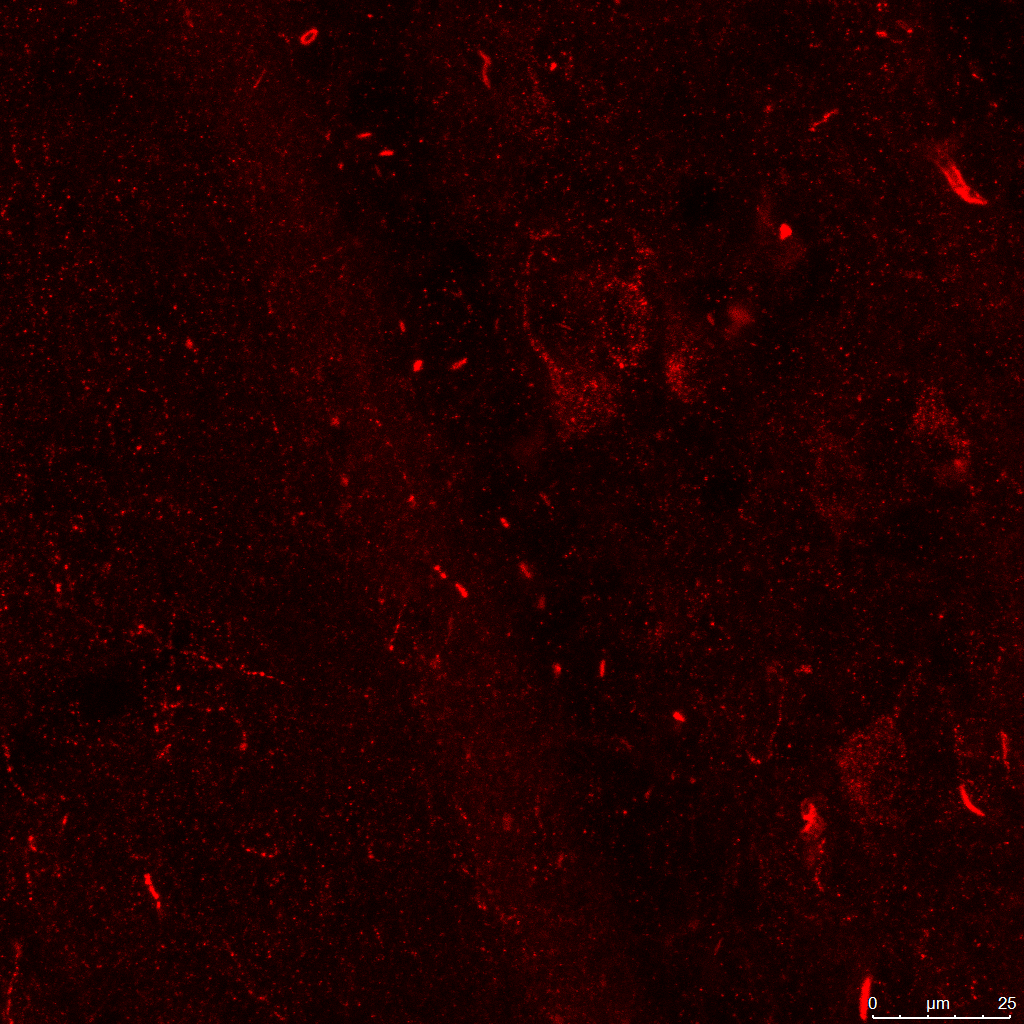

Supplement: Supplementary file 16 — Figure EV4 Source Data [file 44319_2024_218_MOESM16_ESM.zip › Figure EV4/4G/cdKO 8w/DG/2mo_CKO_post_IMPDH2_555_CD68_488_Casp7_633_5.3.21_KFC_post_Hip_DG_SB_Processed001_ch02.tif]

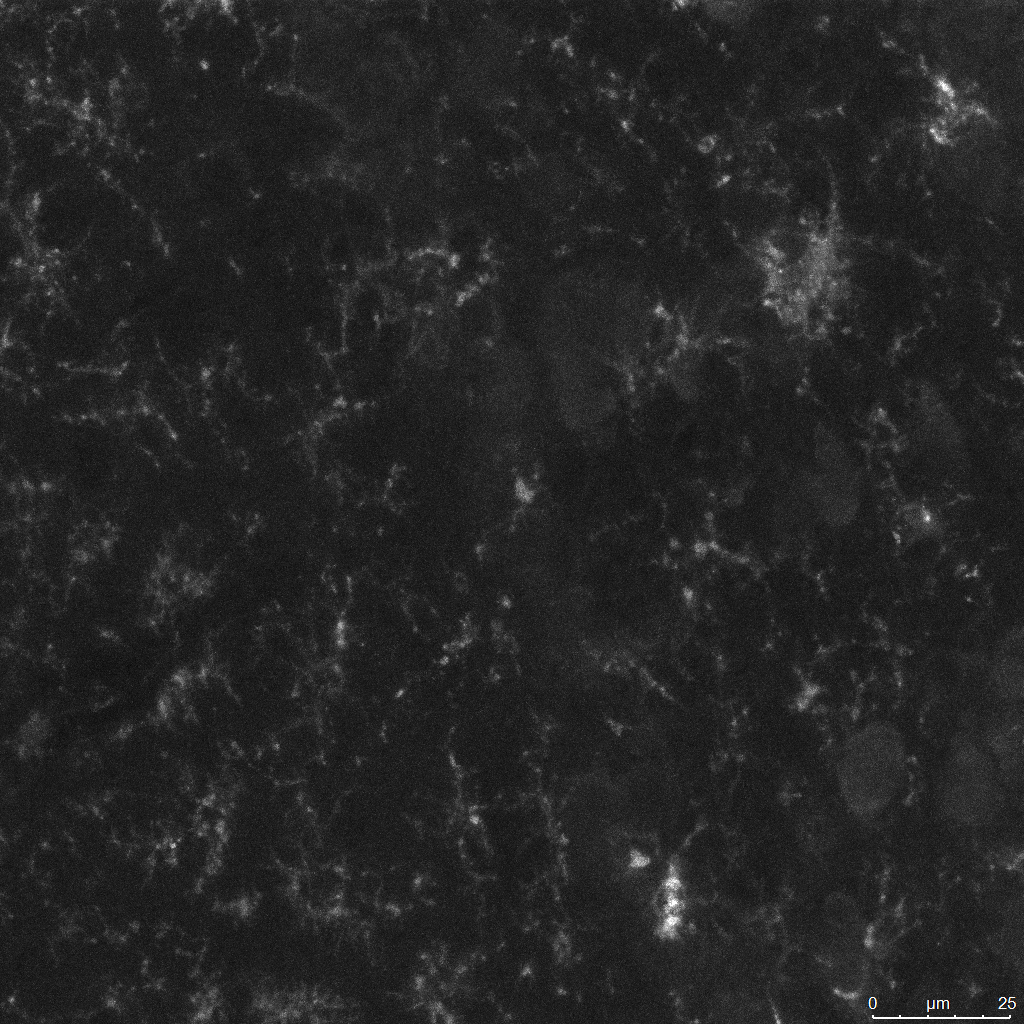

Supplement: Supplementary file 16 — Figure EV4 Source Data [file 44319_2024_218_MOESM16_ESM.zip › Figure EV4/4G/cdKO 8w/DG/2mo_CKO_post_IMPDH2_555_CD68_488_Casp7_633_5.3.21_KFC_post_Hip_DG_SB_Processed001_ch03.tif]

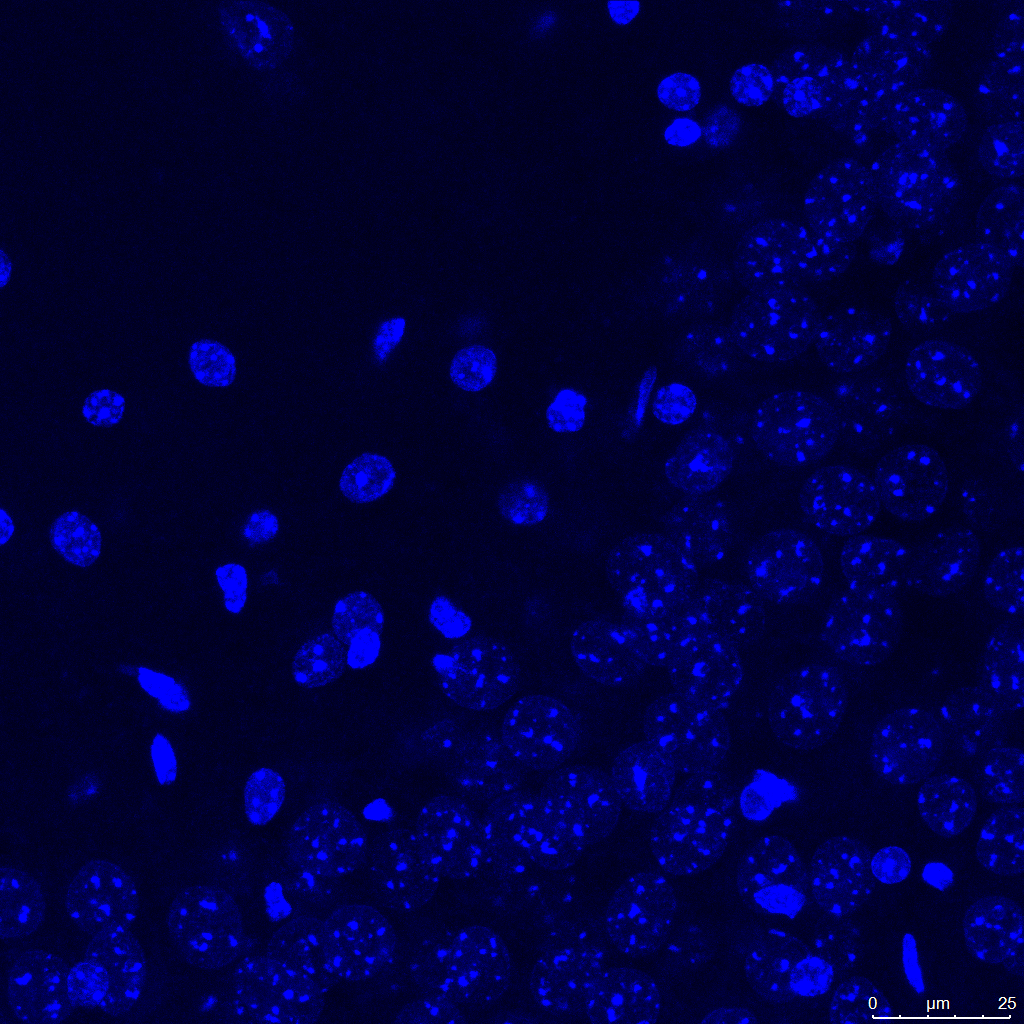

Supplement: Supplementary file 16 — Figure EV4 Source Data [file 44319_2024_218_MOESM16_ESM.zip › Figure EV4/4G/cdKO 8w/CA3/2mo_CKO_post_IMPDH2_555_CD68_488_Casp7_633_5.3.21_KFC_post_Hip_CA3_Processed001_ch00.tif]

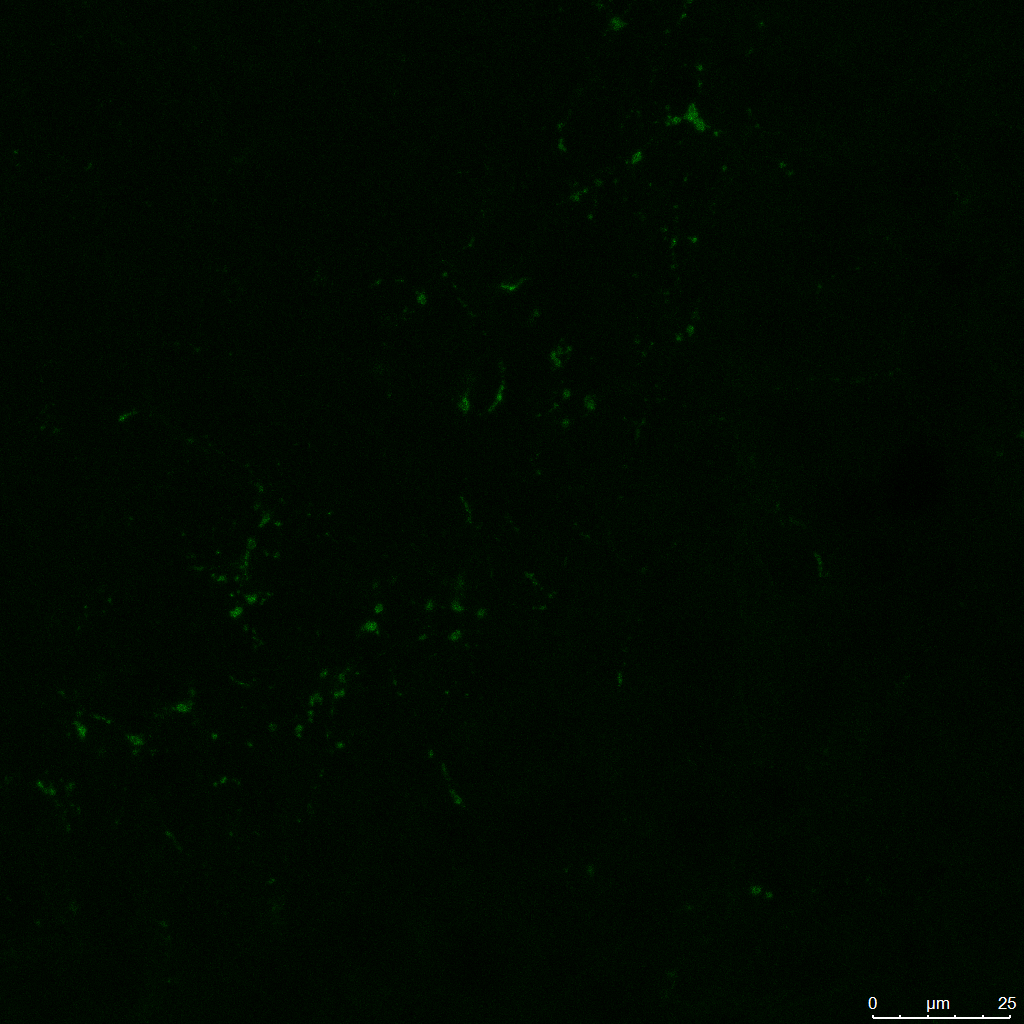

Supplement: Supplementary file 16 — Figure EV4 Source Data [file 44319_2024_218_MOESM16_ESM.zip › Figure EV4/4G/cdKO 8w/CA3/2mo_CKO_post_IMPDH2_555_CD68_488_Casp7_633_5.3.21_KFC_post_Hip_CA3_Processed001_ch01.tif]

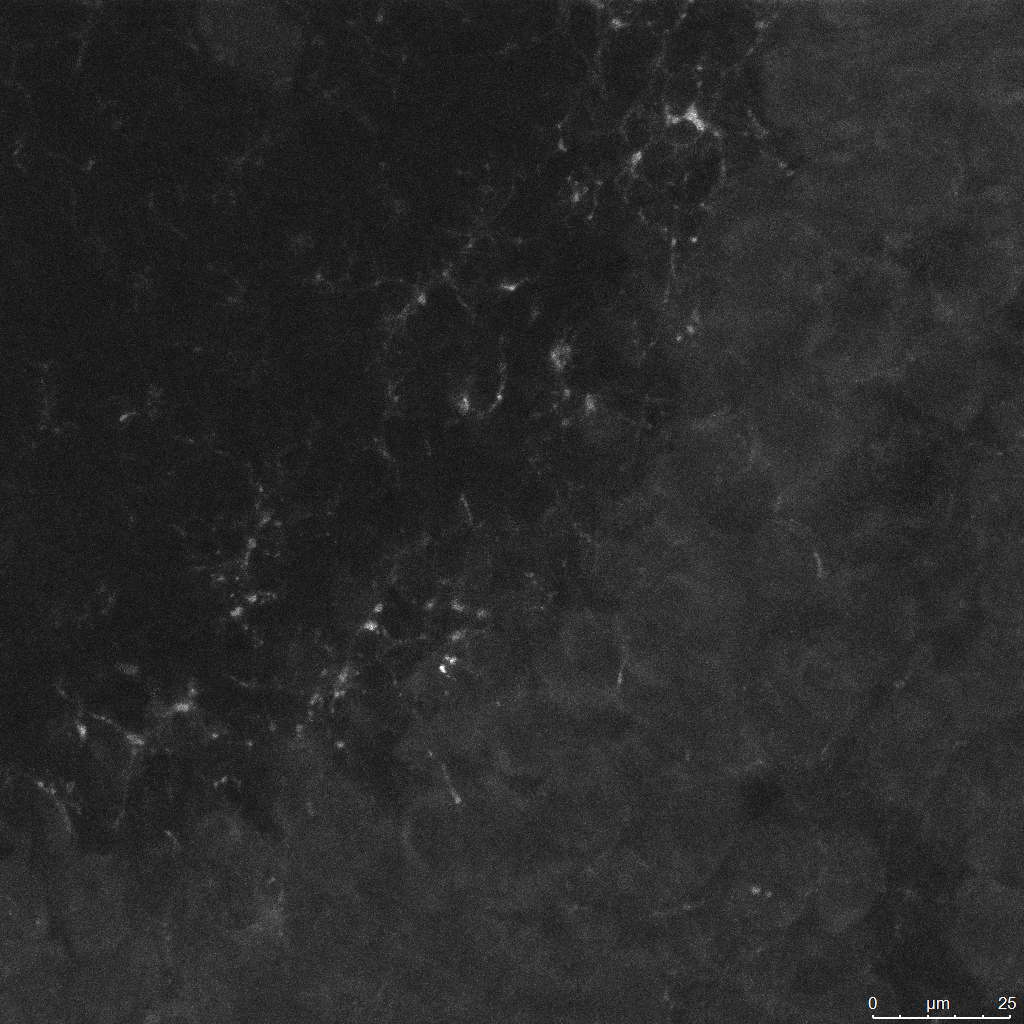

Supplement: Supplementary file 16 — Figure EV4 Source Data [file 44319_2024_218_MOESM16_ESM.zip › Figure EV4/4G/cdKO 8w/CA3/2mo_CKO_post_IMPDH2_555_CD68_488_Casp7_633_5.3.21_KFC_post_Hip_CA3_Processed001_ch03.tif]

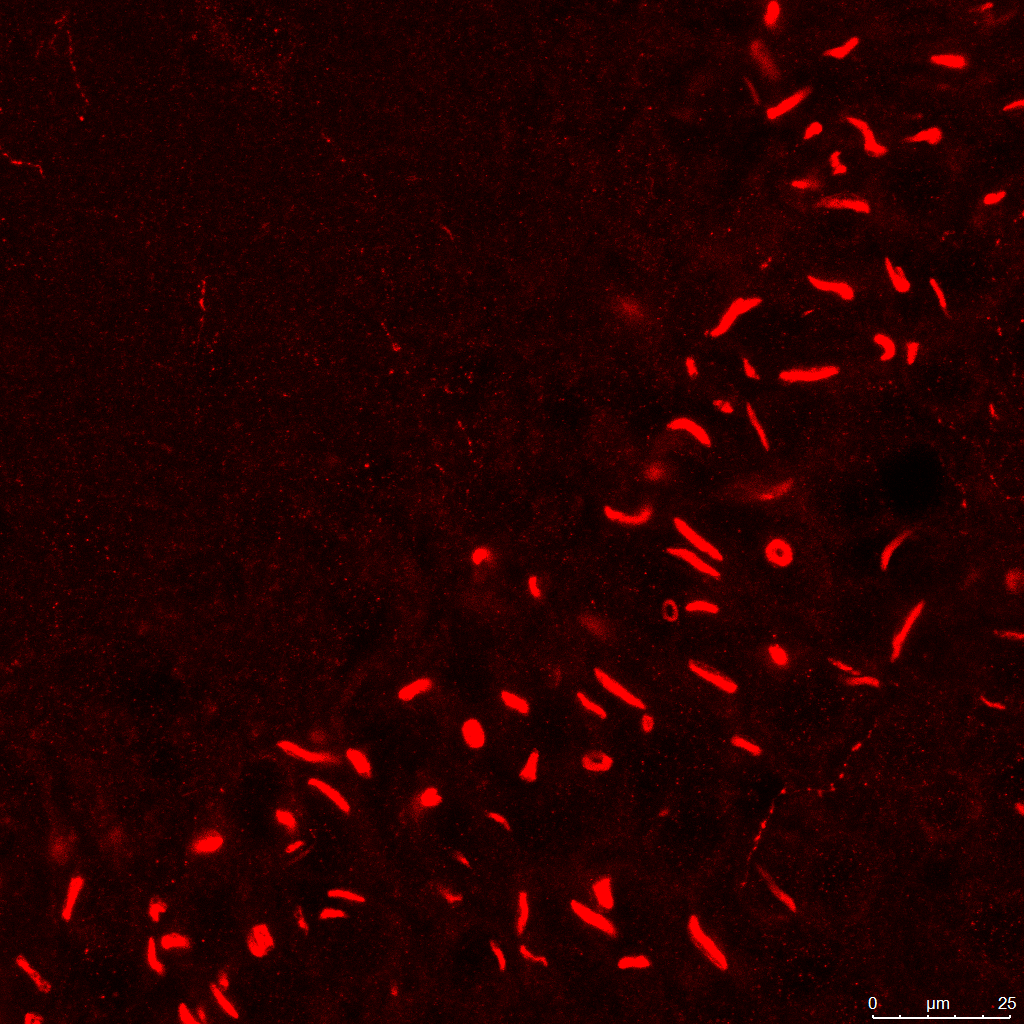

Supplement: Supplementary file 16 — Figure EV4 Source Data [file 44319_2024_218_MOESM16_ESM.zip › Figure EV4/4G/cdKO 8w/CA3/2mo_CKO_post_IMPDH2_555_CD68_488_Casp7_633_5.3.21_KFC_post_Hip_CA3_Processed001_ch02.tif]

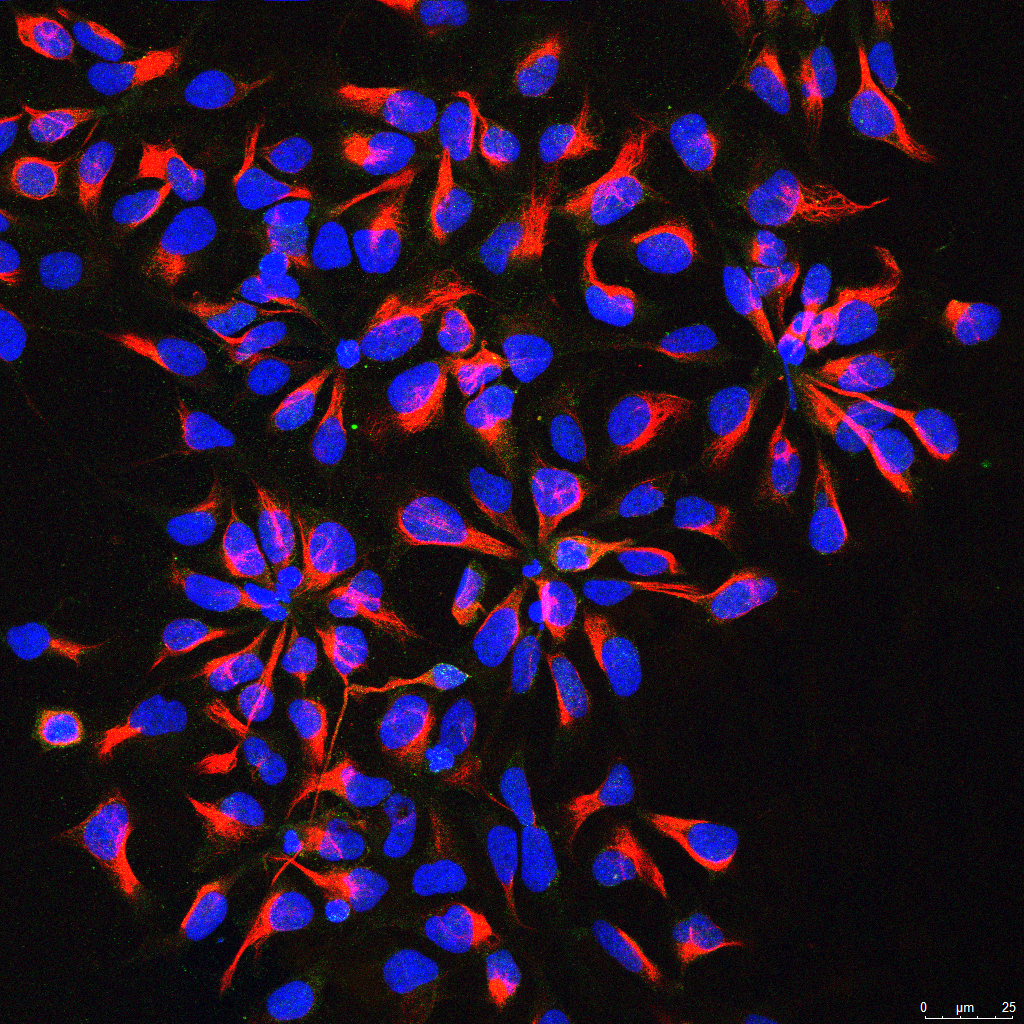

Supplement: Supplementary file 17 — Figure EV5 Source Data [file 44319_2024_218_MOESM17_ESM.zip › Figure EV5/5A/PCH9 NPCs, Pax6-488G, Nestin-555R, ZS 40xZF1-IIIB.tif]

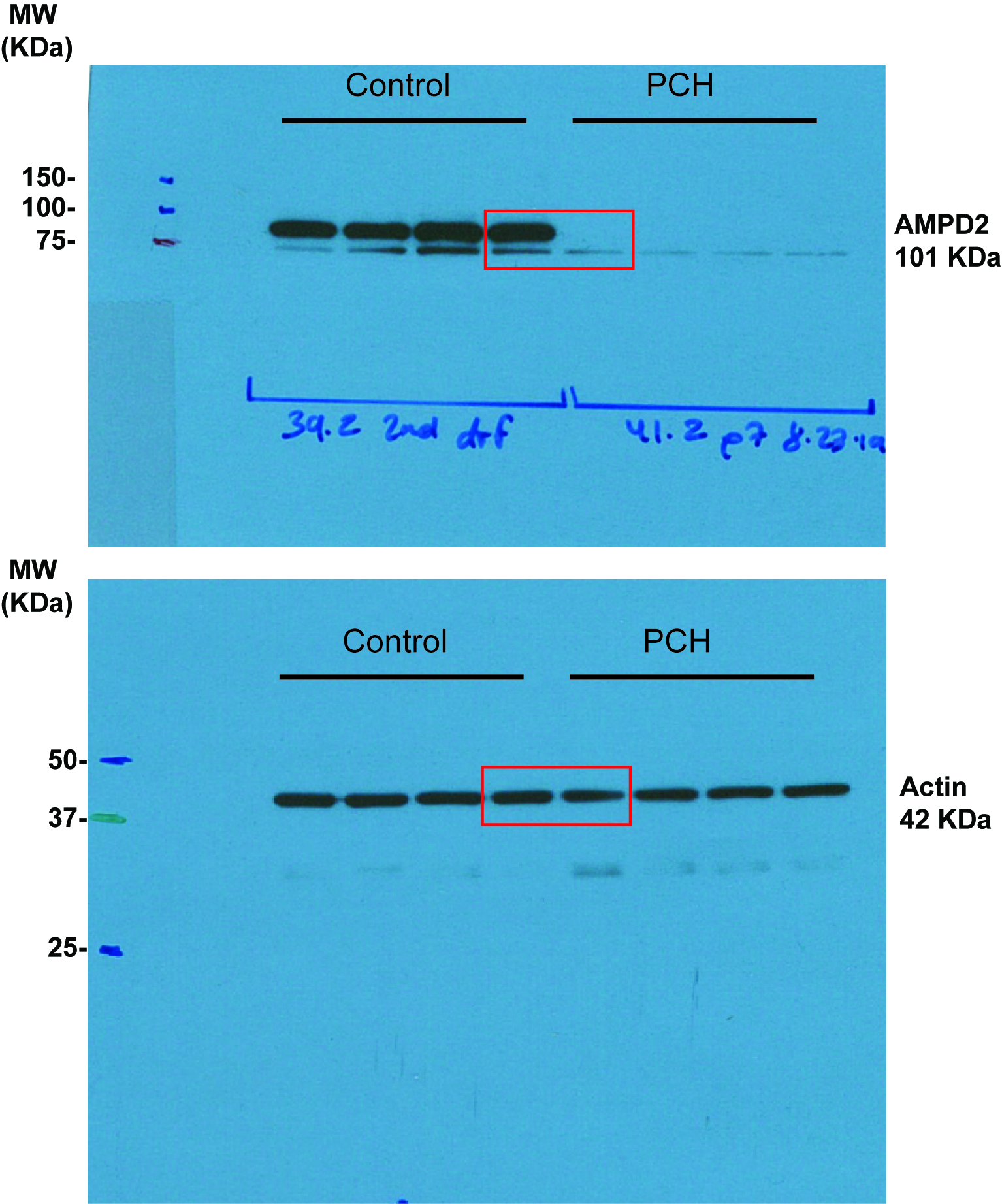

Supplement: Supplementary file 17 — Figure EV5 Source Data [file 44319_2024_218_MOESM17_ESM.zip › Figure EV5/5A/WB EV5A.tif]

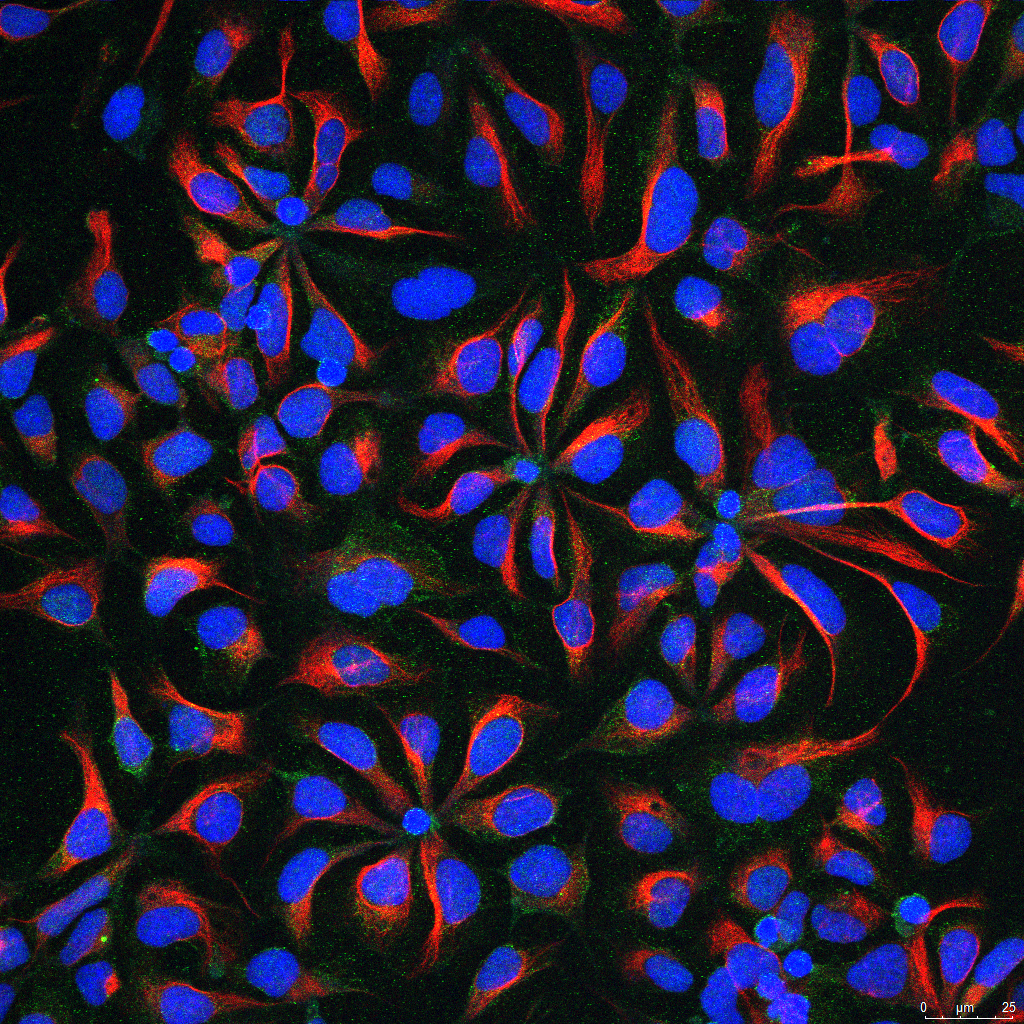

Supplement: Supplementary file 17 — Figure EV5 Source Data [file 44319_2024_218_MOESM17_ESM.zip › Figure EV5/5A/Ctrl NPCs, Pax6-488G, Nestin-555R, ZS 40xZF1-VIIC.tif]
