# Supplementary material for: IMPDH2 filaments protect from neurodegeneration in AMPD2 deficiency
Source: EMBO Rep. 2024 Jul 29;25(9):16. doi: 10.1038/s44319-024-00218-2 (PMC11387764; doi:10.1038/s44319-024-00218-2)
Supplement: Supplementary file 18 — Expanded View Figures [file 44319_2024_218_MOESM18_ESM.pdf]

Expanded View Figures

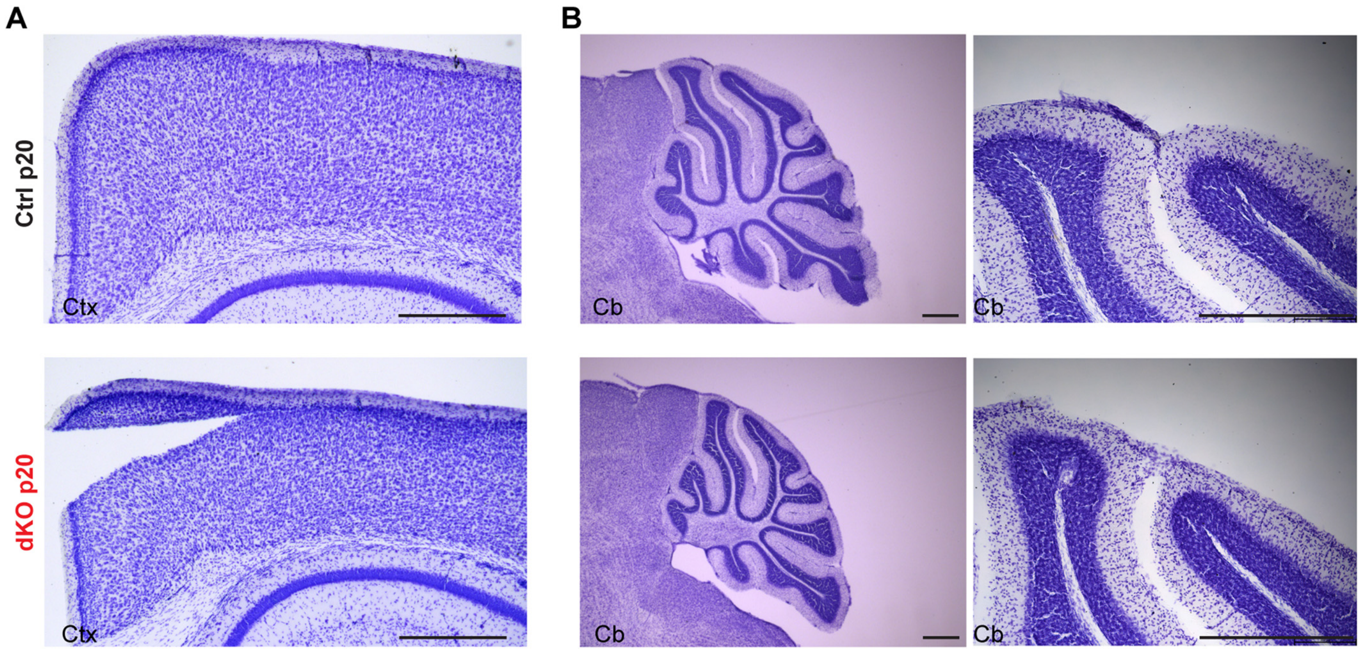

Figure EV1. No signs of overt neurodegeneration in cortex and cerebellum of Ampd2 and Ampd3 double knockout (dKO) mice.

(**A**, **B**) Representative Nissl staining of the cerebral cortex (**A**) and cerebellum (**B**) of control (Ctrl) and double knockout (dKO) mice brain vibratome sections at postnatal day 20 (p20) showing intact tissue, with no structural abnormalities or signs of neurodegeneration. Cerebral cortex images in (**A**) are magnifications of the whole brain coronal section images shown in Fig. 1E. Ctx = Cortex and Cb = Cerebellum. Scale bars, 500 µm. Source data are available online for this figure.

 © The Author(s)

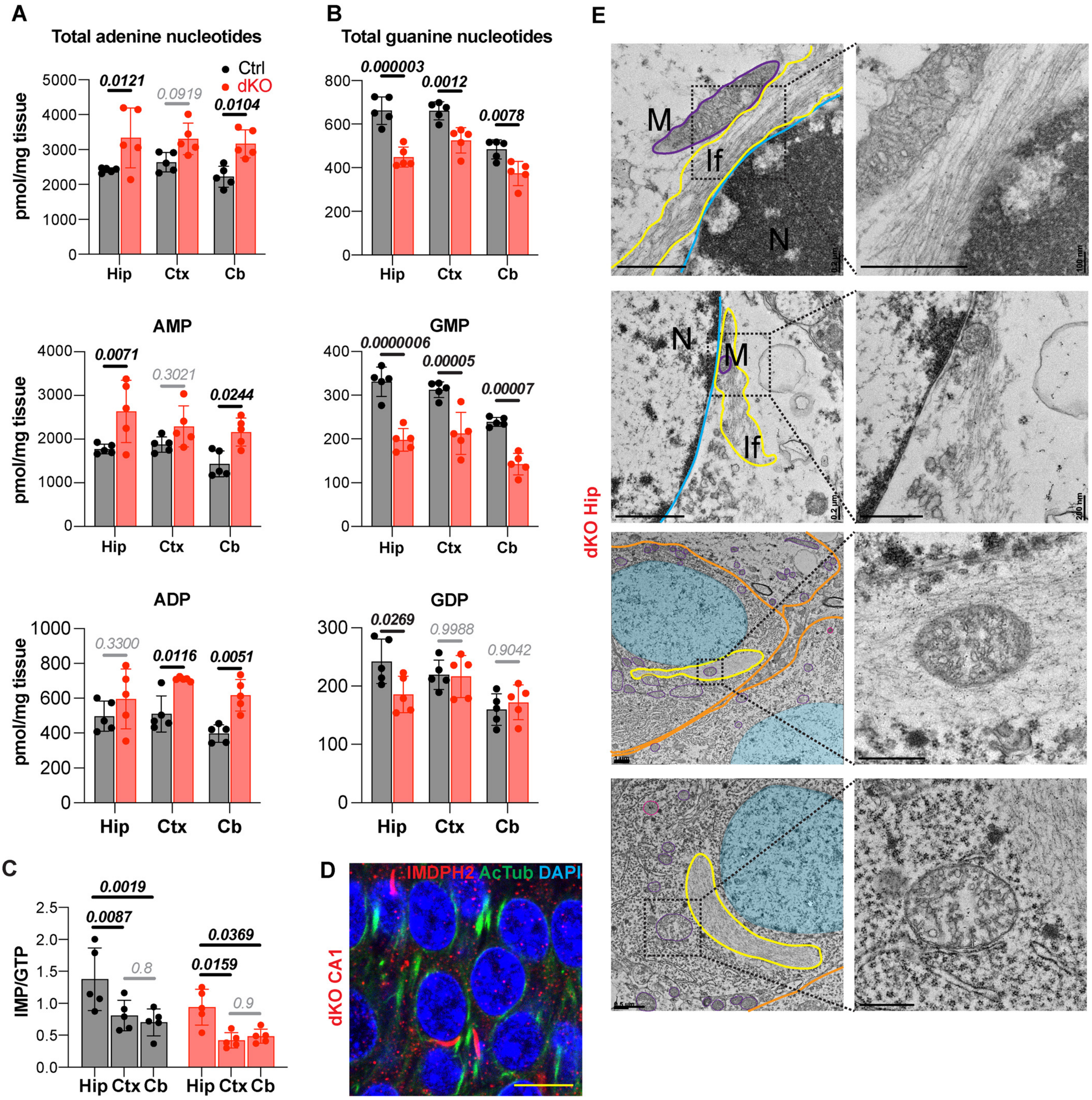

Figure EV2. Purine nucleotide imbalance and IMPDH2 filament assembly in dKO mice brain regions.

(**A, B**) Graphs showing adenine (**A**) and guanine (**B**) nucleotides levels in Hippocampus (Hip), Cortex (Ctx) and Cerebellum (Cb) of control (Ctrl) and double knockout (dKO) mice at postnatal day 20 (p20). Graph shows mean ± SD of $n = 5$ mice per genotype. Significance was calculated using two-way ANOVA with Sidak's post hoc analysis for multiple comparison. (**C**) Graph showing that the ratio between IMP and GTP levels is larger in control and dKO mice hippocampus (Hip) compared with cortex (Ctx) and cerebellum (Cb). Graphs depict mean ± SD of $n = 5$ mice per genotype. Significance was calculated with two-way ANOVA and Sidak's post hoc analysis for multiple comparison. (**D**) Representative immunostaining of IMPDH2 and Acetylated tubulin (primary cilia marker) in dKO mouse at p20. Scale bar, 10 μm. (**E**) Representative transmission electron microscopy images show IMPDH2 filaments (IF) (traced in yellow) within hippocampal neurons. Magnifications (right panels) show IMPDH2 filaments close to mitochondria (M) (traced in purple) and nucleus (N) (blue). Cells are delimited with orange traces. Scale bars, 1 μm (left); Scale bars, 0.5 μm (right). Source data are available online for this figure.

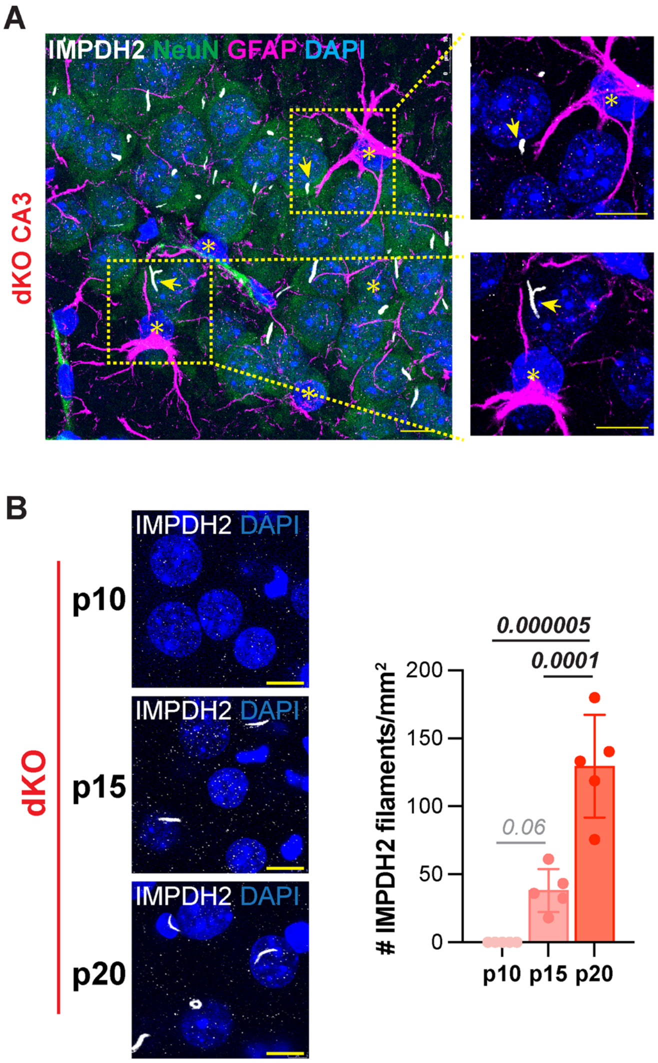

Figure EV3. IMPDH2 filaments progressively accumulate in dKO neurons.

(**A**) Representative IMPDH2, NEUN (neuronal marker), and GFAP (astrocyte marker) immunostainings showing localization of IMPDH2 filaments predominantly in NEUN+ neurons of dKO mice CA3 region. Yellow arrows point to IMPDH2 filaments in neurons and yellow asterisks to astrocytes. Scale bars, 10 µm. (**B**) Representative IMPDH2 immunostainings of dKO cortex (Ctx) at postnatal days 10, 15, and 20, showing age-dependent progressive accumulation of IMPDH2 filaments. Scale bars, 10 µm. Graph shows mean ± SD of IMPDH2 filament density at p10, p15, and p20 dKO mice. $n = 5$ mice per genotype. Significance was calculated using one-way ANOVA with Tukey's post hoc analysis for multiple comparison. Source data are available online for this figure.

 © The Author(s)

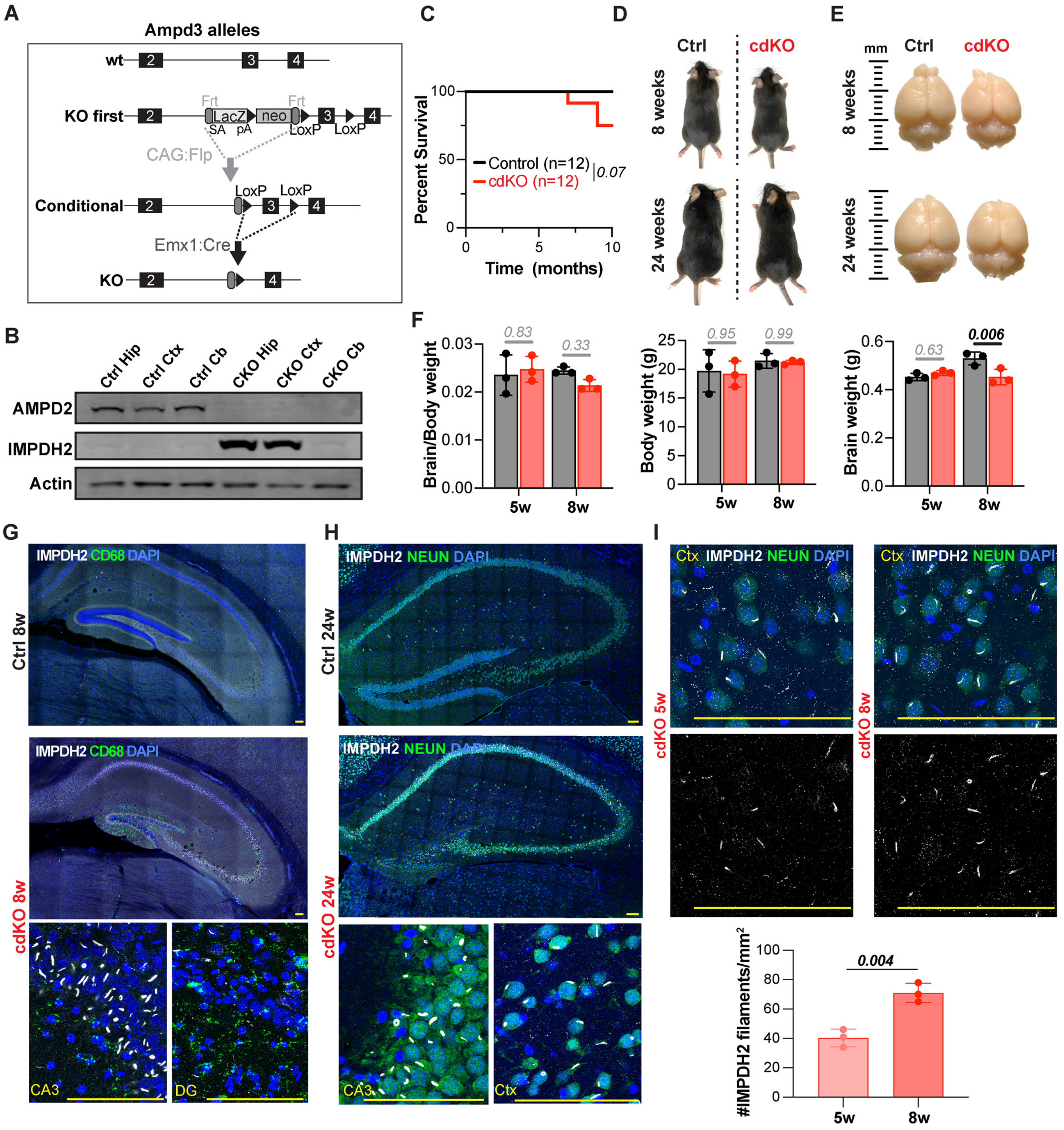
A
Ampd3 alleles
wt
KO first
Frt
LacZ
neo
SA
pA
LoxP
CAG:Flp
Conditional
Emx1:Cre
KO
B
Ctrl Hip
Ctrl Ctx
Ctrl Cb
CKO Hip
CKO Ctx
CKO Cb
AMPD2
IMPDH2
Actin
C
Percent Survival
Control (n=12)
cdKO (n=12)
0.07
Time (months)
D
Ctrl
cdKO
8 weeks
24 weeks
E
mm
F
Brain/Body weight
Body weight (g)
Brain weight (g)
0.83
0.33
0.95
0.99
0.63
0.006
5w
8w
G
IMPDH2 CD68 DAPI
Ctrl 8w
cdKO 8w
CA3
DG
H
IMPDH2 NEUN DAPI
Ctrl 24w
cdKO 24w
Ctx
I
Ctx IMPDH2 NEUN DAPI
cdKO 5w
cdKO 8w
#IMPDH2 filaments/mm²
0.004

Figure EV4. Forebrain deletion of *Ampd2* and *Ampd3* leads to mild loss of brain weight, microglia accumulation in hippocampal DG, and progressive IMPDH2 filament accumulation in CA1-3 and cortex.

(**A**) Schematic diagram showing Cre-mediated excision of floxed Ampd3 exon 3 to generate the conditional double knockout (cdKO) mouse for *Ampd2* and *Ampd3*. (**B**) Representative Western blot showing absence of AMPD2 and upregulation of IMPDH2 in cdKO compared with control (Ctrl) mice (5w). (**C**) Kaplan Meier survival curve showing no differences between $n = 12$ control and $n = 12$ cdKO mice analyzed. Significance was calculated using Log-rank (Mantel-Cox) test. (**D**) Representative image of Ctrl and cdKO mice at 8 and 24 weeks. (**E**) Brain images from Ctrl and cdKO mice at 8 and 24 weeks. (**F**) Brain weight, body weight, and their ratio at 5 and 8 weeks of age. Graph shows mean ± SD of $n = 3$ mice per genotype. Significance was calculated using two-way ANOVA with Sidak's post hoc analysis for multiple comparison. (**G**) Representative immunostainings of IMPDH2 and CD68 in control and cdKO mice at 8 weeks. Bottom panels show higher magnifications of cdKO CA3 with IMPDH2 filaments and low microglia density, and cdKO DG with no IMPDH2 filaments and high microglia density. (**H**) Representative immunostainings of IMPDH2 and NEUN (neuronal marker) in control and cdKO mice at 24 weeks showing nearly undetectable DG and intact CA1-3. Bottom panels are magnifications of CA3 and Ctx showing dense IMPDH2 filaments. (**I**) Representative images of cortex (Ctx) in cdKO mice at 5 (left) and 8 weeks (right) of age showing age-dependent progressive IMPDH2 filament accumulation. Bar graph shows mean ± SD of IMPDH2 filament density in $n = 3$ cdKO mice at 5w and 8w. Significance was calculated using unpaired t-test analysis. Scale bars, 100 μm. Source data are available online for this figure.

 © The Author(s)

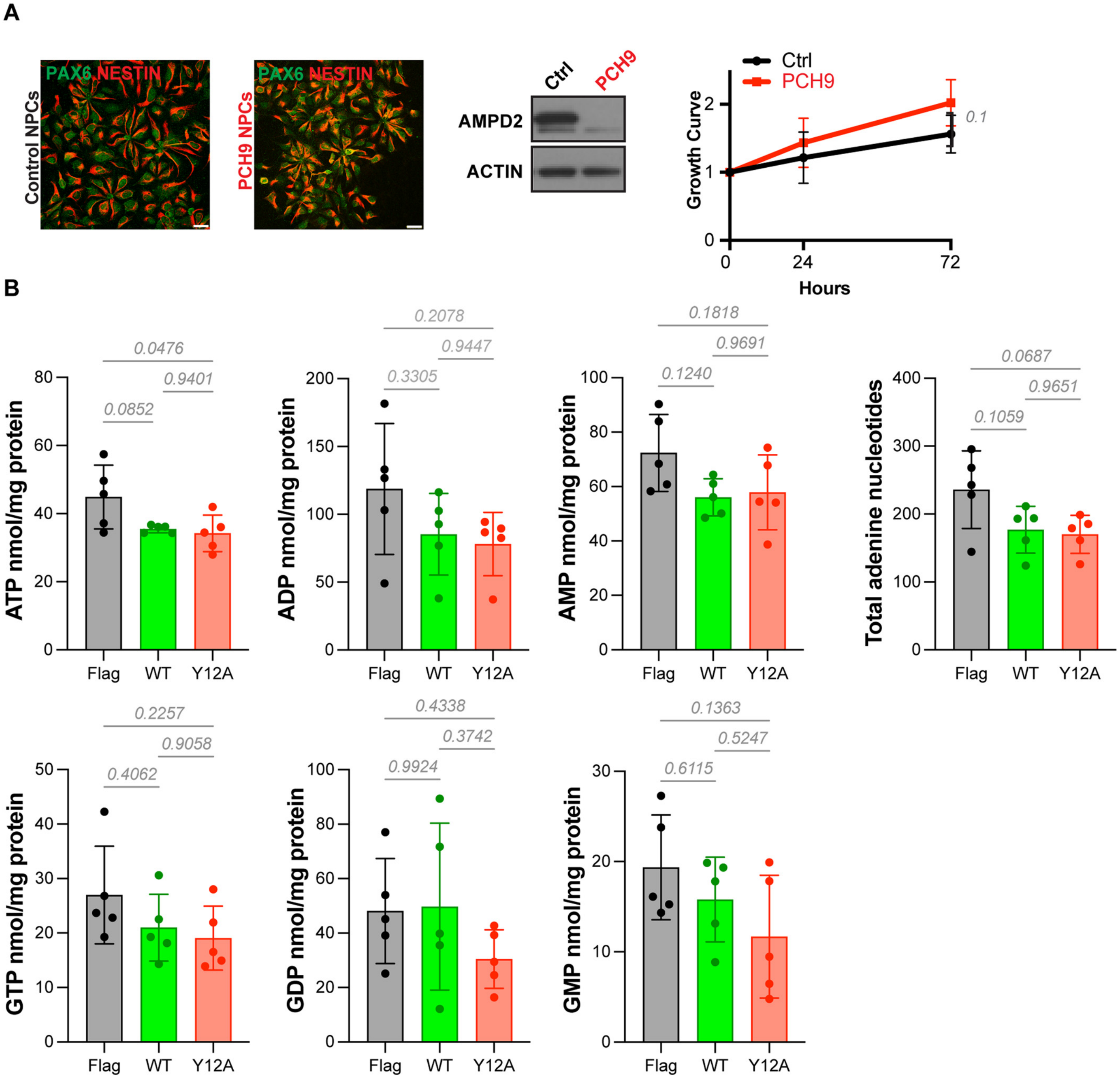

Figure EV5. Similar purine nucleotide levels upon IMPDH2-WT or IMPDH2-p.Y12A transduction in PCH9 NPCs.

(**A**) Representative images of PCH9 patient and unaffected control NPCs immunostained with anti-PAX6 and anti-NESTIN NPC markers. Western Blot analysis showing lack of AMPD2 protein in PCH9 NPCs compared to controls. Graph shows growth curves of control (Ctrl) and PCH9 NPCs over a 72 h period. Graph shows mean ± SD of 4 independent cultures. Statistical difference was calculated comparing slops of simple linear regression. (**B**) Adenine and Guanine nucleotides levels in PCH9 NPCs expressing FLAG, IMPDH2-WT, and IMPDH2-p.Y12A. Graph shows mean ± SD of $n = 5$ samples per group. Significance was calculated with one-way ANOVA with Tukey's post hoc analysis for multiple comparison. Scale bars, 25 µm. Source data are available online for this figure.

© The Author(s)
